# Widespread effects of catecholamines on growth of human gut bacteria

**DOI:** 10.1038/s41522-026-00948-2

**Published:** 2026-03-20

**Authors:** Michael Daniels, Dinely Wijayagunasekera, David Berry

**Affiliations:** Division of Microbial Ecology, Department of Microbiology and Ecosystem Science, Center for Microbiology and Environmental Systems Science, University of Vienna, Vienna, Austria

**Keywords:** Microbiology, Physiology

## Abstract

The interactions between hosts and their microbiomes are driven in part by chemical communication, which influences immune responses, metabolism, and microbial community structure. Neuroendocrine signals are central to this bidirectional communication, forming the basis of microbial endocrinology. Although host-derived hormones, including catecholamines, are known to affect microbial physiology, much of the existing literature focuses on a limited number of model organisms or complex in vivo systems, where disentangling direct microbial responses from host-mediated effects is challenging. As a result, systematic comparative analyses of direct bacterial responses under controlled conditions remain scarce. Here, we performed a systematic in vitro screen under anaerobic conditions to assess catecholamine effects on the growth dynamics of phylogenetically diverse human gut bacteria. Catecholamines altered multiple growth parameters in a species-specific manner, with effects detectable at nanogram concentrations. Multivariate analyses, including principal component analysis and non-metric multidimensional scaling, revealed lineage-associated response patterns across taxa. Although derived from monoculture experiments, these intrinsic responses provide a comparative framework for understanding how direct hormone–microbe interactions may contribute to microbiome dynamics under host stress. Overall, this study provides a quantitative cross-species dataset to inform future systems-level investigations in microbial endocrinology.

## Introduction

The interaction between host and symbiotic microorganisms represents one of the most influential aspects of communication within ecosystems^1^. A particularly important example of such interactions occurs in the animal gut, which harbors both commensal and pathogenic microorganisms and serves as a key site for host–microbe communication. The human gastrointestinal (GI) tract in particular constitutes a highly complex and dynamic microbial ecosystem composed of trillions of bacteria^2^. These microorganisms play crucial roles in food metabolism and nutrient acquisition^3^, the stimulation of the immune system^4^, and contribute to the exclusion of enteric pathogens^5^. The GI microbiota is therefore essential not only for host immunity and metabolism but also for overall human health and well-being^3,6,7^. Increasing evidence suggests that host–microbe interactions extend beyond the immune and metabolic pathways to include communication with the endocrine system^8,9^, thereby giving rise to the emerging field of microbial endocrinology.

Microbial endocrinology investigates the bidirectional communication between microbiota and its host neuroendocrine systems, encompassing both the production of neuroactive compounds by microorganisms and their capacity to sense and respond to host-derived neurochemicals^10,11^. It examines how neurochemicals, the concentrations of which can be altered by stress, affect bacterial physiology and growth^12^. Recent integrative frameworks have emphasized the broad physiological, ecological, and evolutionary implications of microbe–neurochemical interactions across host-associated ecosystems^13^. It has been shown that microorganisms respond to neuroendocrine molecules, such as neurotransmitters and hormones, that are canonically associated with mammalian signaling. These responses are mediated via receptors, suggesting that these molecules serve as signals for intercellular communication between host and microbe^14^. Hormones have also been shown to modulate bacterial gene expression, potentially leading to physiological consequences for the host^15^. Notably, studies have shown that pathogenic neurotoxins can alter murine neuroendocrine hormone levels, highlighting the reciprocal nature of host–microbe interactions within this framework^16^. Furthermore, the gut microbiome has been shown to modulate mammalian behavior in various ways. Studies in germ-free mice showed significant alterations in cognitive ability, memory, stress reaction, anxiety, and social interaction^17,18^. Similarly, in humans, emotional states and disorders, such as stress-related irritable bowel syndrome, have been associated with gut microbiota composition and function^19–22^.

A wide range of host-derived neuroactive compounds have been implicated in host–microbiota communication. These include serotonin, histamine, acetylcholine, γ-aminobutyric acid (GABA), and various neuropeptides, many of which are produced or metabolized within the gastrointestinal tract and can directly influence bacterial physiology and behavior^8–11^. Among the key hormones involved in these processes are catecholamines, which play major roles in regulating memory and stress responses. The gastrointestinal tract itself represents a major source of endogenous dopamine production in humans, accounting for a substantial proportion of total body dopamine synthesis, independent of sympathetic innervation^23^.

In the present study, we focus on catecholamines as a well-characterized and physiologically relevant class of stress-associated neurochemicals with established links to bacterial growth, virulence, and dysbiosis, allowing for a controlled and comparative assessment of bacterial responsiveness to endocrine compounds. The catecholamines adrenaline, noradrenaline and dopamine as well as their precursor L-3,4-dihydroxyphenylalanine (DOPA) are a major group of neurotransmitters involved in regulating cognitive functions^24^, motivation^25^ as well as memory^26^. Hereafter, unless explicitly stated otherwise, the term “catecholamines” is used collectively to refer to adrenaline, noradrenaline, dopamine, and their immediate biosynthetic precursor DOPA. These compounds also mediate physiological adjustments that prepare the body for physical activity (the “fight-or-flight response”), including elevated heart rate and blood pressure as well as decreased metabolic activity^27^. Emerging evidence suggests that the composition and function of the gut microbiota can be influenced by catecholamines^28^.

Although extensive in vitro and in vivo studies have demonstrated that catecholamines can directly influence bacterial growth, virulence, and physiology^29^, much of the existing literature focuses on a limited number of model organisms or on complex in vivo systems, where disentangling direct microbial responses from host-mediated effects and community interactions remains challenging^30^. As a result, comparative analyses that systematically assess catecholamine responses across diverse gut bacterial taxa under standardized experimental conditions remain relatively scarce. Catecholamines have been shown to influence bacterial proliferation and pathogenicity and enhance bacterial adhesion to host tissues^31^. For instance, enterohemorrhagic *Escherichia coli* (EHEC) can detect host-derived catecholamines through quorum-sensing signaling pathways, inducing the expression of virulence genes^32^. Importantly, bacteria do not possess mammalian adrenergic receptors such as β₂-adrenergic receptors. Instead, catecholamine sensing in bacteria is mediated through distinct molecular mechanisms, including two-component signaling systems such as QseC/QseB, which have been characterized in *Escherichia coli* and related enteric taxa, as well as through indirect mechanisms involving iron acquisition and metabolic modulation. Several of the taxa examined in this study, including *Escherichia coli* and *Klebsiella pneumoniae*, are known to harbor such catecholamine-responsive systems^33,34^. Overall, catecholamines can affect a plethora of bacterial physiological traits, including growth, motility, biofilm formations, and pathogenic potential^15,31,35,36^.

While these findings have significantly advanced the field of microbial endocrinology, they also highlight key limitations. Specifically, distinguishing the direct, causative responses of individual gut microorganisms to host stress hormone release from secondary, community-level effects remains challenging. Moreover, confounding factors such as host diet, age, and health status, as well as complex interactions between microbial communities and the host immune system, introduce additional pleiotropic layers that complicate data interpretation. While several mechanisms by which catecholamines influence bacterial physiology have been identified, e.g., iron mobilization from host proteins^37^ and stimulation of horizontal gene transfer^38^, these insights are largely derived from a limited number of model organisms. A comprehensive understanding of how diverse gut bacterial species directly sense and respond to catecholamines, therefore, remains incomplete.

To address this gap, comparative, in vitro experiments under controlled conditions provide an opportunity to study bacterial responses to catecholamines in isolation, thereby eliminating the complexity inherent in microbiome systems. This reductionist approach enables the development of targeted experimental model systems that can later be integrated into broader frameworks to investigate community-level and host-microbiome dynamics. To enable a systematic comparison of catecholamine responsiveness across gut bacteria, we selected a panel of 29 bacterial species representative of the phylogenetic and functional diversity commonly observed in the adult human gut microbiota^39^. The collection spans multiple dominant gut-associated phyla and includes both commensal and potentially opportunistic taxa that are prevalent in healthy adult populations. The number of strains was chosen to balance breadth of taxonomic coverage with experimental tractability and is comparable to prior high-throughput phenotypic screening studies^40^.

To that end, in this study, we conducted a comprehensive in vitro screening of 29 phylogenetically diverse members of the human gut microbiome to assess their responses to each of the four catecholamines. We systematically characterized bacterial growth dynamics under catecholamine exposure to evaluate species-specific interaction patterns. By combining comparative analyses across diverse taxa with controlled experiential conditions, this study aims to establish a foundation for disentangling the direct effects of host neuroendocrine signals on gut bacteria. Our findings enlarge current perspectives on catecholamine–microbe interactions that can inform future systems-level approaches investigating how bacterial hormone interactions might contribute to community-level dynamics and host physiology.

## Results

### General effects of catecholamines on the growth of gut-associated bacterial species

Microbial propagation within the host is impacted by a plethora of external cues in various ways, ranging from nutrient uptake by polysaccharide degradation via growth stimulation by molecules from the gastrointestinal environment up to the level of full growth arrest caused by antimicrobial compounds. In order to understand the impact of catecholamines on individual bacterial species, we performed a screening of their effects on the growth dynamics of representative gut bacteria.

We first set out to investigate general changes in overall growth in the presence of catecholamines. To this end, we selected 29 bacterial strains found within the human gastrointestinal tract. The selected strains span the four major bacterial phyla commonly found in the human gut and encompass six different classes, 10 orders, and 12 families. They include both facultative and strict anaerobes, as well as abundant commensal species and opportunistic pathogens, allowing us to assess catecholamine effects across the ecological and phylogenetic spectrum of gut microbes.

To maximize sensitivity to potential catecholamine-induced effects, we initially assessed bacterial growth at high catecholamine concentrations (1 mg/mL). To allow for cross-compound comparison, concentrations are reported in mass-based units; corresponding molar concentrations are provided in the Methods. We decided on this approach for an initial, unbiased screen to ensure that any possible stimulatory or inhibitory responses could be detected. Additionally, the use of high catecholamine concentrations was intended to probe the upper bounds of bacterial growth responses under extreme or non-physiological conditions, rather than to directly model average luminal concentrations in the human gut.

We cultured all strains anaerobically in a diluted, nutrient-rich brain heart infusion (BHI) broth supplemented with hemin to support the growth of fastidious anaerobes^41^. Using diluted rich media with high levels of compounds of interest also allows us to investigate whether compounds can be utilized as a carbon source; the latter of which is not observable in full rich media at low compound concentrations^40^. Furthermore, L-cysteine was added as a reducing agent to inhibit the oxidation of the dihydroxybenzene ring system of catecholamines to toxic o-quinones and avoid ROS production upon autooxidation^42^. Using this approach, we incubated individual bacterial species with each of the four catecholamines and quantified total bacterial growth using the area under the growth curve (AUC), a parameter reflecting cumulative biomass formation over time^43,44^.

**Fig. 1:**
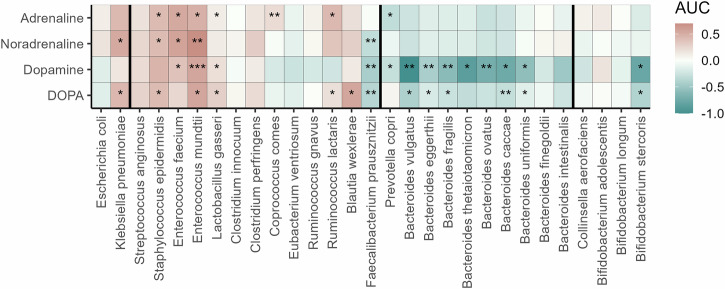
Catecholamines selectively influence overall growth estimated as the area under the curve (AUC) of gut bacteria. Heatmap of changes in overall growth (AUC) for 29 gut bacteria (*N* = 3) when exposed to different catecholamines (vertical axis). Colors represent relative differences in AUC of catecholamine exposure compared to growth in diluted media without catecholamine addition. Light colors indicate little to no change compared to the control. Blueish colors represent a reduced AUC. Red colors represent an increased total growth. Stars represent significant changes in growth between the control and respective catecholamine addition: *p < 0.05, **p < 0.01, ***p < 0.001 (two-sided *t*-test). Details about the underlying statistical analysis can be found in the Supplementary Table 1. Vertical lines indicate separation of the different taxonomic phyla left to right: *Pseudomonadota*, *Bacillota*, *Bacteroidota, Actinomycetota*.

Our results show that catecholamines exerted widespread and highly variable effects on bacterial growth (ANOVA, *p* < 0.0001; Fig. 1). Some species exhibited substantial growth increases in the presence of catecholamines, whereas others showed markedly reduced growth, including complete inhibition. For instance, *Faecalibacterium prausnitzii* displayed no detectable growth in the presence of dopamine. This suggests that high catecholamine concentrations might have toxic effects on certain bacteria. Other species, in contrast, displayed increased growth with catecholamines. *Enterococcus mundtii*, for example, showed up to 180% increased growth. Overall, these results indicate that catecholamines can profoundly influence bacterial growth, with responses varying across individual bacterial species (ANOVA, *p* < 0.0001) that might drive the differentiation of the gut microbiota.

Since our initial high concentrations do not reflect general catecholamine levels typically found in the human gut, we next wanted to investigate what concentrations of catecholamines induce effects on overall bacterial growth. As BHI is an animal-derived medium originally developed as a substitute for blood-based formulations with the intention to “…extract the growth factors or hormones…”^45^, we assumed that it might intrinsically contain catecholamines that could confound experimental outcomes. To test this, we quantified dopamine concentrations in BHI using a highly sensitive Enzyme-linked Immunosorbent Assay (ELISA).

**Fig. 2:**
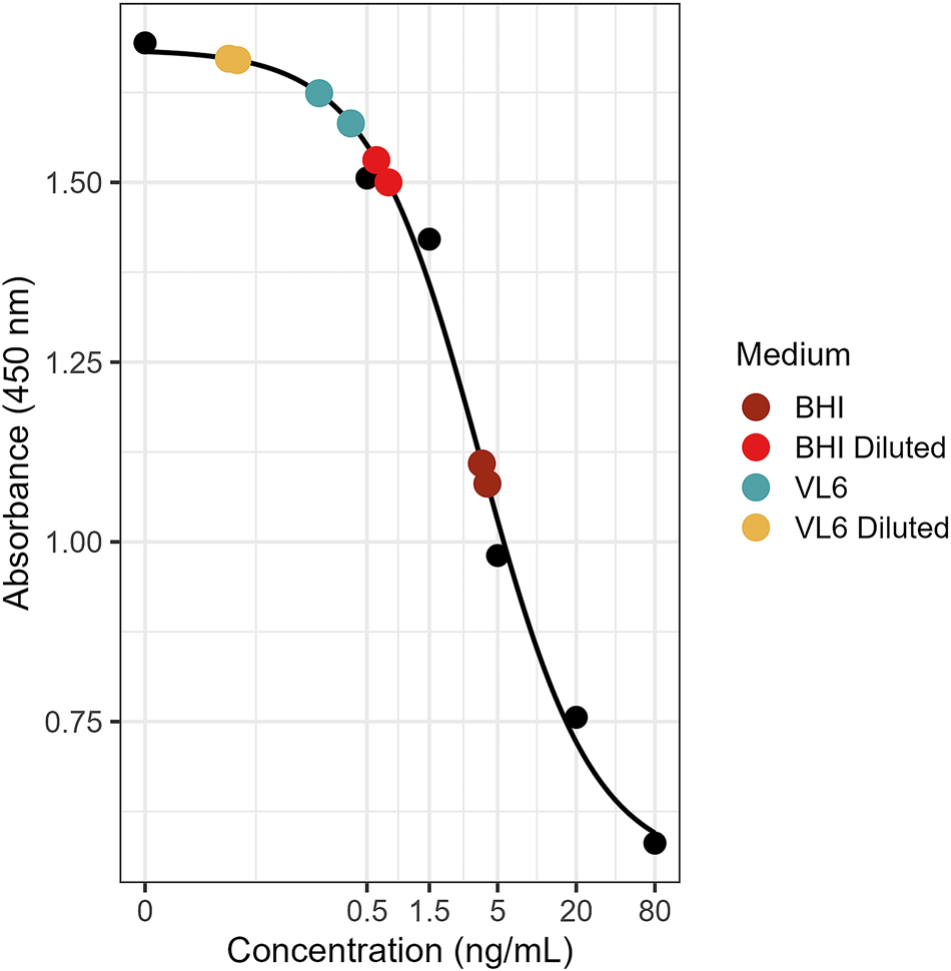
High sensitivity ELISA reveals the presence of dopamine in brain heart infusion (BHI). Results of Dopamine high sensitivity ELISA (ImmuSmol). Black points represent 450 nm absorbance measurements (*y*-axis) for the six standards plotted against their reported dopamine concentrations (*x*-axis). Black line represents a fitted standard curve (4-parameter logistic curve, Marquart-like). Colored points represent the measured absorbances (*y*-value) for tested media and the calculated estimated dopamine concentration (*x*-value) fitted against the standard curve.

Dopamine was detected at ~4 ng/mL in full BHI (Fig. 2). Dopamine concentrations were measured in full and diluted BHI and VL6 media (*N* = 2 for each condition). We therefore transitioned to a plant-based, animal-component-free medium (VL6) for subsequent experiments. ELISA measurements confirmed that dopamine levels in VL6 were below the assay’s detection limit. Quantification of dopamine in full and diluted BHI and VL6 media revealed residual dopamine concentrations of 3.79–4.18 ng/mL in full BHI and 0.59–0.73 ng/mL in diluted BHI. In contrast, VL6 exhibited apparent dopamine concentrations at or below the assay’s sensitivity threshold (0.22–0.38 and 0.05–0.04 ng/mL) and was therefore considered catecholamine-free. While the assay is designed for high sensitivity, low-level cross-reactivity with structurally related compounds cannot be fully excluded.

### Concentration-dependent and species-specific effects of catecholamines on bacterial growth

To minimize background contamination by endogenous catecholamines commonly present in animal-derived media, we conducted all subsequent experiments in a diluted, plant-based, animal-free medium (VL6 Formedia) supplemented with hemin to support the growth of more fastidious anaerobic species. As expected, this medium was not able to support the growth of all of the bacterial species tested in BHI, which was developed specifically to cultivate a wide variety of gut-derived strains. Nevertheless, VL6 still sustained the growth of a subset of our bacterial species spanning all four major phyla found inside the human gut. Twelve of those species were selected for subsequent experiments.

**Fig. 3:**
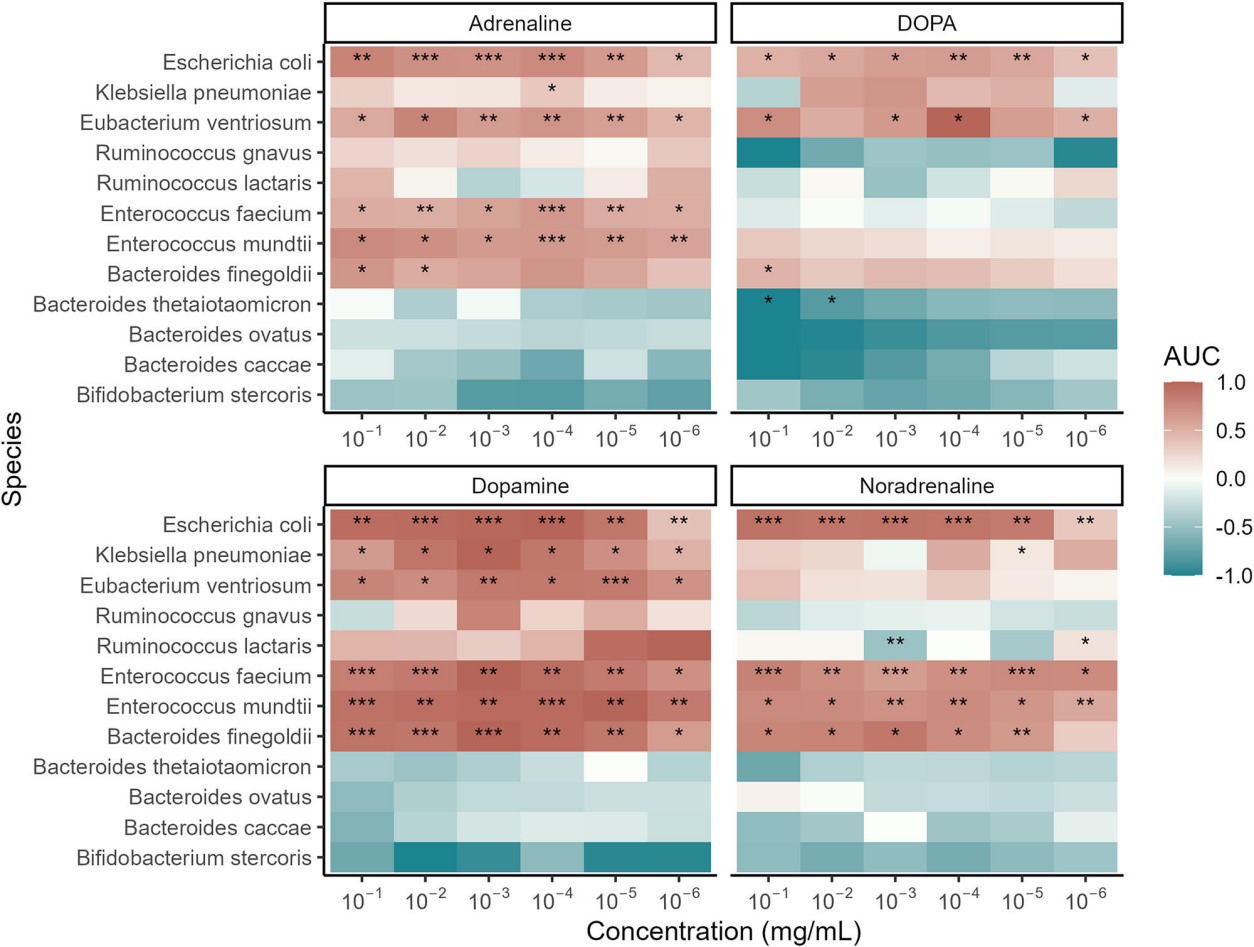
Catecholamine concentrations consistently affect overall bacterial growth. Heatmaps of paired *t*-test AUC differences in catecholamine metabolism across bacterial species. Heatmaps display the normalized differences in area under the curve (AUC) values across different concentrations (100 µg–1 ng/mL of catecholamine) for each bacterial species (*N* = 4). Panels represent the different catecholamines. Colors indicate the direction and magnitude of change, with red colors representing higher and blue colors lower AUC values compared to the control. Statistical significance was assessed using paired *t*-tests; significance levels are denoted as *p* < 0.05 (*), *p* < 0.01 (**), and *p* < 0.001 (***). The underlying statistical values can be found in the Supplementary Table 2.

Having established that high catecholamine concentrations can strongly influence the overall growth of diverse gut-associated bacteria, we next aimed to sensitivity of bacteria to catecholamines using a 10-fold titration experiment spanning six orders of magnitude (100 µg/mL–1 ng/mL). To this end, each strain was grown under strictly anaerobic conditions in the presence of one of four catecholamines. Control cultures without catecholamine addition served as the baseline. Growth was quantified by calculating the area under the growth curve (AUC), and normalized differences relative to controls were visualized in a heatmap to facilitate comparison across species and concentrations (Fig. 3).

We observed pronounced effects of catecholamines on bacterial growth (ANOVA, *p* < 0.0001; Fig. 3). While some taxa, such as *Enterococcus mundtii* and *Escherichia coli*, exhibited significant increases in total growth (AUC) in the presence of catecholamines across all concentrations, others, like *Bacteroides thetaiotaomicron* and *Bifidobacterium stercoris* tended to be suppressed in their growth when grown on the different catecholamines. These opposing trends indicate that, even at very low concentrations, catecholamines can influence overall bacterial growth in a species-specific manner rather than exerting a uniform stimulatory or inhibitory effect across taxa.

Certain species, particularly *E. coli* and *E. mundtii*, displayed statistically significant increased growth even at catecholamine concentrations as low as 1 ng/mL, which is roughly four times below the concentration measured in BHI medium. This remarkable sensitivity suggests that these bacteria might be finely attuned to host-derived stress signals and capable of responding to minute concentration changes in the host environment.

Taken together, these findings demonstrate that catecholamines can differentially influence bacterial growth in a species-specific fashion at very low concentrations. The observation that significant effects occur even at nanogram concentrations underscores the potential ecological relevance of host stress hormones in shaping gut microbial population dynamics.

### Multidimensional analysis reveals species-specific effects of catecholamines on growth dynamics

While total biomass accumulation, represented by the area under the growth curve (AUC), provides an integrative measure of bacterial proliferation, it captures only one aspect of microbial growth dynamics. Bacterial growth is a complex, multistage process involving distinct physiological phases, i.e., lag, exponential growth, and stationary phases. These phases are tightly linked to metabolism, changes in the environment through the consumption and production of metabolites, interspecies interactions, and ecological functions^46^. Changes in growth dynamic parameters associated with those individual phases can offer a deeper insight into how bacteria respond to external stimuli such as catecholamines.

**Fig. 4:**
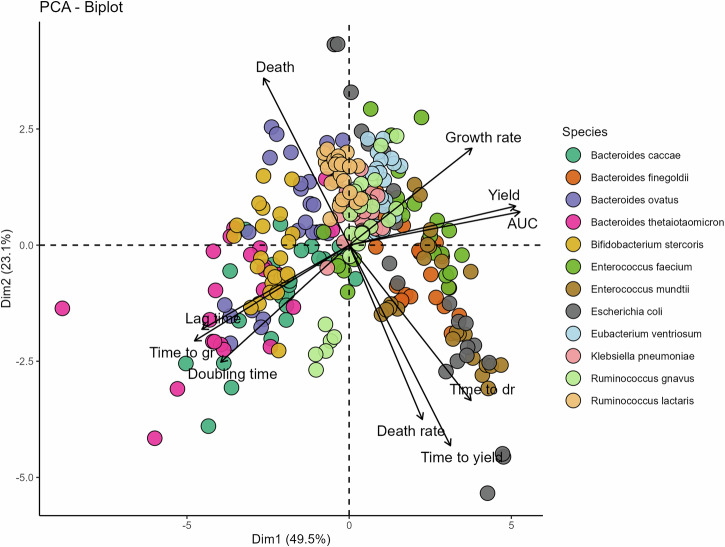
Differences in growth dynamics due to catecholamine exposure. Principal component analysis (PCA) plot of absolute differences in growth dynamics for 12 bacterial species exposed to catecholamines. Points represent the calculated differences (two-sided *t*-test) in growth parameters for all individual bacterial species when exposed to various concentrations of each of the four catecholamines compared to a control. Colors represent the 12 different species. Arrows indicate the contribution of each of the calculated growth dynamic parameters for the distribution across dimensions one and two of the PCA. For readability, “death rate” and “growth rate” have been abbreviated into “dr” and “gr”, respectively.

To gain a more holistic understanding of how catecholamines influence bacterial growth physiology, we extracted ten distinct growth parameters from our incubation experiments. In addition to AUC, these included carrying capacity (yield), growth rate, lag time, loss in biomass (death), death rate, doubling time, and the time points at which bacteria reached their maximum growth rate, maximum death rate, and carrying capacity (time to yield). This multidimensional dataset allowed us to examine not only whether catecholamines affected overall bacterial growth, but also how these effects manifested across different aspects of growth physiology. We applied a principal component analysis (PCA) to reduce the dimensionality of the dataset across individual species, type of catecholamine, compound concentration, and growth dynamic parameters, and visualize overall patterns in bacterial responses. The first two principal components together explained ~73% of the total variance in growth responses to catecholamine exposure. Dimension 1 (~50% of variance) was primarily associated with growth metrics reflecting biomass accumulation (AUC and carrying capacity). Dimension 2 (~23% of variance) was mainly driven by parameters characterizing the later parts of the growth curve, such as time to reach carrying capacity and loss in biomass in late stationary and death phases (Fig. 4).

The resulting PCA biplot shows that bacterial species clustered distinctly in this reduced-dimensional space, suggesting that species identity and specific growth dynamic responses to catecholamines are tightly coupled. A PERMANOVA analysis confirmed that species identity significantly influenced the distribution of strains across principal component space (*R*^2^ = 0.421, *p* < 0.001), whereas neither the identity of the catecholamine compound (*R*^2^ = 0.042) nor its concentration (*R*^2^ = 0.00061) explained a substantial proportion of the observed variation. Importantly, this indicates that across the dataset as a whole, species identity accounted for more variance than hormone identity, but does not rule out biologically meaningful catecholamine-specific effects in individual taxa or growth parameters. Notably, some species exhibited general inhibition by catecholamines across various growth dynamic parameters, with *Bacteroides thetaiotaomicron*, for example, displaying decreased carrying capacity and growth rate and increased lag time, while other species, such as *E. coli* or *E. mundtii*, showed the opposite effect. This indicates that catecholamines affect bacterial taxa in a multimodal fashion.

**Fig. 5:**
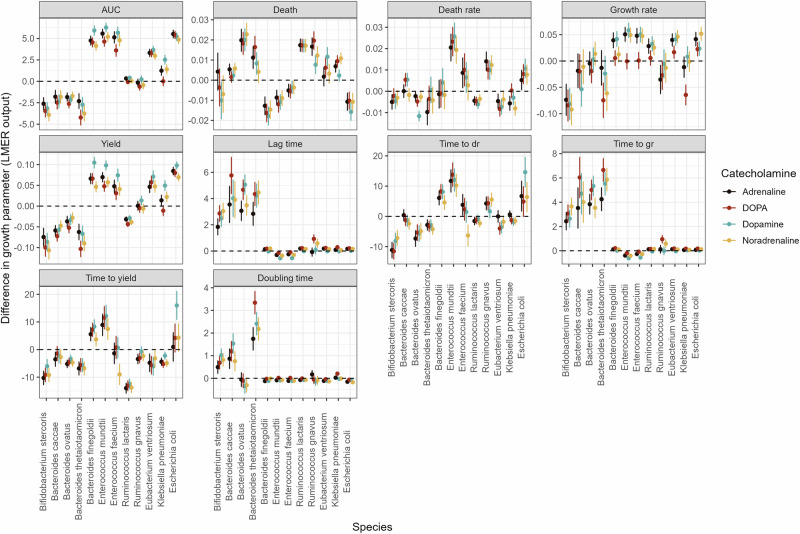
Differences in growth dynamic parameters due to catecholamine exposure. Results of a linear mixed-effect model show marked effects of catecholamines on various aspects of bacterial growth dynamics. Points represent the mean calculated differences (linear mixed-effect model) of each growth dynamic parameter for individual species when exposed to catecholamines compared to a control. Colors represent the four different catecholamines. Detailed info on the results of the statistical analysis can be found in the Supplementary Table 3.

Further analysis of our data using linear mixed-effects models (Fig. 5) supported these findings. Across multiple growth parameters, catecholamine identity only shows minor effects on either direction or magnitude of the individual bacterial response. While certain compounds, such as DOPA, produced subtle differences in growth rate for specific taxa, the overall pattern remained largely species-driven. Notably, taxa belonging to *Bacteroidota* and *Bifidobacteria* tended to exhibit prolonged lag phases and reduced carrying capacities in response to catecholamines, whereas members of *Bacillota* (formerly Firmicutes) and *Pseudomonadota* often displayed accelerated growth dynamics and shortened lag phases.

These results highlight that catecholamines modulate bacterial growth in complex, multidimensional ways that extend beyond overall biomass accumulation. Importantly, the direction and strength of these effects vary across bacterial taxa, reflecting phylogenetically structured response patterns. Collectively, this multidimensional analysis demonstrates that catecholamines exert diverse and species-specific influences on bacterial physiology, underscoring their potential to shape microbial community dynamics through both growth-promoting and growth-inhibiting mechanisms.

### Differences in growth responses cluster bacterial species in a phylum-specific fashion

**Fig. 6:**
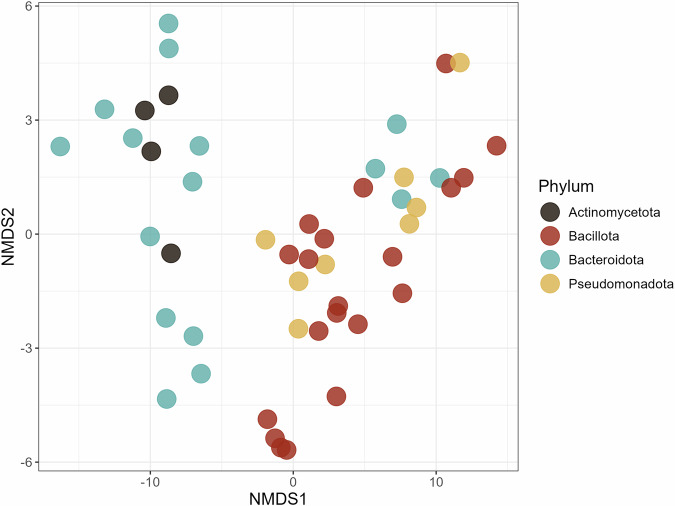
Non-metric multidimensional scaling (NMDS) ordination (stress = 0.053) based on Manhattan distances among bacterial species derived from 11 standardized model estimates of growth dynamic parameters. Each point represents a bacterial species colored by phylum. Proximity indicates similarity in multivariate response profiles to catecholamine exposure. Distinct clustering by phylum suggests lineage-specific patterns in bacterial growth modulation by host catecholamines.

Our analysis so far revealed that catecholamines act as potent, multifaceted modulators of bacterial growth physiology, influencing not only the overall accumulation of growth but also multiple individual dynamic parameters that define the bacterial life cycle. By quantifying a set of distinct growth metrics, we found that individual bacterial species exhibited a unique combination of responses to catecholamine exposure. These responses were highly individualized and multidimensional. Therefore, catecholamines do not elicit a uniform physiological growth response across taxa but instead affect multiple aspects of bacterial growth in a species-specific manner. To determine whether these complex, species-level responses followed any higher-order structure, i.e., through phylogenetic signal or ecological aspects, we integrated all growth-dynamic parameters derived from our mixed linear model outputs into a non-metric multidimensional scaling (NMDS) analysis (Fig. 6).

Strikingly, despite the high dimensionality and diversity of individual growth responses, bacterial species clustered in NMDS space (Fig. 6) according to their phyla (manhattan-distance, stress = 0.053; *k* = 3; 10 growth parameters included). Members of *Bacteroidota* and *Actinomycetota* (i.e, *Bifidobacterium stercoris*) grouped together, whereas *Bacillota* and *Pseudomonadota* formed distinct clusters within the ordination space. A PERMANOVA confirmed that the bacterial phyla significantly explained the observed distribution of bacterial growth responses to catecholamine exposure within the limits of the taxonomic sampling employed (*R*^2^ = 0.40, *p* < 0.0001). This indicates that while responses to catecholamines are species specific, with individual bacteria showing differences in various aspects of their growth curve, overall these individual multi-modular responses appear to be phylogenetically structured and might reflect shared evolutionary or ecological components. The clusters found from our analysis of in vitro growth responses mirror shifts found in mammalian studies of stress-associated conditions and dysbiosis associated with elevated catecholamine levels. Individuals experiencing stress exhibit shifts in their *Bacillota/Bacteroidota* ratio^47^ as well as higher abundances of *Pseudomonadota*^48^, alongside reductions in *Bifidobacterium*^49^, precisely the pattern observed in our NMDS clustering^50^. Intriguingly, our findings indicate that while the responses to catecholamine exposure influence individual bacteria in various aspects of their growth, patterns emerge where some species exhibit overall growth stimulation, e.g., *E. mundtii* and *E. coli*, other taxa such as *B. thetaiotaomicron* and *B. stercoris* seem to be generally inhibited by catecholamines (Figs. 4 and 5). These directionalities of these in vitro response patterns mirror microbiome shifts reported in human studies of stress-associated conditions. These parallels suggest that direct bacterial responses to catecholamines might partially underlie the stress-associated changes of the gut microbiome described in vivo. We note, however, that despite several findings being consistent with our observed patterns, other studies describe divergent taxonomic shifts under stress-related conditions. This highlights that current scientific evidence remains inconclusive and may depend on host, environmental, and methodological factors.

Overall, by systematically quantifying changes in multiple growth-dynamic parameters across phylogenetically diverse gut bacteria, we demonstrate that catecholamines induce species-specific and phylogenetically structured alterations in bacterial physiology. These effects occur independently of host factors, such as immune responses, diet, or age, highlighting that even simple in vitro assays can recapitulate the fundamental stress-linked compositional patterns seen in the human gut microbiome. Such findings provide strong evidence that microbe–hormone interactions at the single-species level might contribute directly to host-associated microbiome shifts under stress and disease.

## Discussion

In this study, we systematically investigated the effects of catecholamines on the growth dynamics of a phylogenetically diverse set of human gut bacteria under controlled anaerobic conditions. Using a unified experimental and analytical framework, we observed pronounced, species-specific responses across multiple growth parameters, with response patterns that were structured by bacterial lineage. By applying a systems-level, bottom-up approach to microbial endocrinology, our aim was to disentangle how catecholamines directly influence microbial growth in vitro and to establish a comparative foundation for understanding how such effects may contribute to gut microbial ecology.

Similar high-throughput growth phenotyping approaches have proven highly informative in identifying conserved and species-specific bacterial responses to chemical compounds, serving as a critical first step toward mechanistic dissection^40^. In this context, our study complements existing frameworks in microbial endocrinology by providing a standardized, cross-species phenotypic baseline for catecholamine responsiveness under identical experimental conditions. Although catecholamine sensitivity has previously been documented for individual bacterial species^12^, a systematic comparison of direct growth responses across a broad range of gut-associated taxa has been lacking. By enabling direct cross-species comparison, our results reveal conserved and divergent response patterns that are not readily apparent from single-organism studies.

One of the most striking outcomes of our study is that catecholamines impact bacterial growth parameters at remarkably low concentrations of down to 1 ng/mL. At these concentrations, catecholamines increase several growth dynamics parameters, including AUC (biomass accumulation over time), lag time, growth rate, and carrying capacity. The observation that catecholamines exert measurable effects at nanogram concentrations is consistent with earlier work demonstrating their roles as signaling molecules at low concentrations^12^. Our results extend these findings by showing that such sensitivity is widespread across diverse gut bacterial taxa and manifests in species-specific growth dynamic responses under standardized conditions.

These findings are notable because, outside vitamins^50^ and trace minerals^51^, only a few chemical compounds are known to elicit such strong effects on bacterial growth at similarly low concentrations. The only biochemical compounds known to the authors that induce such strong effects on bacterial metabolism and growth at such low concentrations are quorum-sensing molecules such as the autoinducer AI-2^52^. This underscores the potential of catecholamines to serve as interkingdom signaling molecules that allow bacteria to sense and respond to the host´s physiological states.

The divide in growth responses between potentially “beneficial” and “pathogenic” taxa is particularly interesting in light of previous findings that members of the *Pseudomonadota* and *Bacillota* phyla, such as *E. coli* and *Enterococcus* spp. whose virulence is often context-dependent, have been shown to induce virulence gene expression in the presence of catecholamines^53^. In contrast, the negative impact of catecholamines on *Bifidobacterium* and *Bacteroides* species could partially explain the observed reduced proportions of these “beneficial groups” in stress-related dysbiosis. One possible explanation for this pattern relates to different metal requirements. Catecholamines are known to mobilize iron from host-associated complexes^37^, which may preferentially benefit taxa with iron-dependent metabolisms, whereas genera such as *Bifidobacterium* are comparatively tolerant of low-iron conditions^54^.

Our findings support the broader concept that catecholamines can act as signaling molecules influencing bacterial physiology and might promote traits associated with pathogenicity. Increases in local catecholamine concentrations, such as those that occur during epithelial barrier disruption or physiological stress, could selectively enhance the growth of opportunistic pathogenic species. This would enable them to outcompete commensals and disrupt gut homeostasis. This notion resonates with Paracelsus´ principal that “the dose makes the poison”—“dosis sola facit venenum”^55^, where local population increases of otherwise harmless bacteria can trigger pathogenic phenotypes through quorum-sensing-dependent activation of virulence factors. We emphasize, however, that our monoculture experiments do not directly test virulence activation, and these interpretations remain hypothetical.

An interesting exception to this taxonomic divide can be found in *Bacteroides finegoldii*, which, despite belonging to the *Bacteroidota*, exhibited growth responses more similar to *E. coli* and *Enterococcus*. This species has recently been described as a potential opportunistic pathogen^56^. This supports the hypothesis that the ability to positively react to host-derived catecholamines might be linked to pathogenic potential. Such exceptions underscore the complexity of host–microbe interactions and suggest that catecholamine sensitivity may represent an adaptive trait conferring ecological advantages under specific host physiological conditions.

Differential responsiveness to catecholamines might also reflect ecological niche specialization within the gastrointestinal tract. Spatial factors such as bacterial association with luminal versus mucosal environments, or preferential localization along the small and large intestine^57^, could influence exposure to host-derived neurochemicals. However, precise spatial localization of many gut taxa remains incompletely resolved, and intestinal mixing likely exposes diverse taxa to host-derived neurochemicals over time. Consistent with this view, imaging-based studies suggest the proximal colon functions as an incompletely mixed bioreactor rather than a sharply stratified system^58^. Consequently, while spatial organization may contribute to observed response patterns, our data do not allow direct inference of niche-specific catecholamine exposure.

The responsiveness of bacteria to host-derived neurochemicals may also be influenced by strain origin and evolutionary history^59^. Prior work has shown that exposure to host environments can select for enhanced sensitivity to catecholamines, even in strains that were previously unresponsive. The strains examined here were derived from human-associated isolates but were not selected for by host health status or disease context. As such, our findings reflect general patterns across representative gut taxa rather than host condition-specific adaptations. Future studies comparing strains isolated from distinct clinical contexts, such as inflammatory bowel disease or stress-associated conditions, will be important to determine how the conditions within the host gut environment shape bacterial neurotransmitter responsiveness.

While our in vitro findings demonstrate robust effects of catecholamines under laboratory conditions, translating these results to the in vivo gut environment requires careful consideration. Reliable quantitative measurements of free catecholamine concentrations within the human gut lumen are currently lacking, particularly at spatial and temporal scales relevant to microbes. Quantifying the actual concentrations of free catecholamines within the gut lumen remains a major challenge. Estimates from murine models suggest levels of 9.0–60.5 ng/g for norepinephrine and 15.7–177.0 ng/g for dopamine^60^, but these values vary across individuals, gut regions, and physiological states. Accordingly, the concentration range used here was not intended to replicate a specific luminal condition, but rather to probe bacterial responses across a broad exposure spectrum.

Several limitations of this study should be acknowledged. First, our analysis focuses on growth-based phenotypic readouts, which represent integrative outcomes of multiple cellular processes but do not resolve the underlying molecular mechanisms. Future studies combining growth phenotyping with metabolomic, transcriptomic, or proteomic analyses will be necessary to dissect the pathways involved in catecholamine responsiveness. Second, our experiments were conducted in monoculture. This does not allow for the assessment of interspecies interactions, such as cross-feeding, competition, or cooperation, that shape microbiome dynamics. Nevertheless, isolating direct hormone–bacterium interactions is a critical prerequisite for interpreting community-level phenomena and complements in vivo studies that are often confounded by immune, dietary, and host-specific factors.

By integrating multiple quantitative growth parameters into a multidimensional analytical framework, including PCA and NDMS, we provide a comparative systems-level assessment of bacterial responses to host-derived neurochemicals across diverse gut taxa. Species identity emerged as the dominant determinant of growth response patterns, exceeding the influence of catecholamine identity or concentration. These findings suggest that bacterial species possess intrinsic metabolic or physiological traits that govern their sensitivity to catecholamines, and that these traits might be phylogenetically structured. Given the limited number of species per phylum examined here, our analysis does not permit formal inference of phylogenetically conserved traits, but instead highlights candidate lineage-associated response patterns that warrant further investigation.

Future research should aim to bridge the gap between single-species insights and community-level behavior. Controlled co-culture experiments and synthetic microbial communities offer a tractable next step to study how catecholamines influence interspecies interactions, competition, and cooperation^61^. Furthermore, recent work shows that catecholamines can induce metabolic and redox changes within bacterial cells^62^. These effects might further affect bacterial growth dynamics. In order to elucidate the precise mechanisms by which catecholamines affect bacterial growth dynamics and physiology (e.g., redox-mediated stress responses), targeted follow-up experiments, such as intracellular ROS quantification, are required. Ultimately, integrating such bottom-up experimental data with systems-level modeling and metagenomic profiling of human cohorts could illuminate how stress and neurochemical signaling shape microbiome structure and function in health and disease.

Taken together, our findings show catecholamines as powerful modulators of bacterial growth dynamics. By utilizing anaerobic incubations with a multidimensional analytical framework, we reveal that catecholamines exert diverse, species-specific effects that advance our understanding of microbial endocrinology and highlight host neurochemistry as a direct factor influencing microbial physiology and ecology.

## Methods

### Bacterial strains, media, and batch cultures

The following strains were used. The strain panel was designed to capture phylogenetic diversity across major bacterial lineages commonly detected in the adult human gastrointestinal tract rather than to represent a specific anatomical region of the gut. Strains were selected based on their prevalence in human microbiome datasets^39^, availability as well-characterized isolates, and ability to grow reproducibly under the anaerobic conditions used in this study. Type strains are uniquely identifiable by their culture collection accession numbers. Strains that were isolated in our previous studies are accompanied by the corresponding literature citation. *Bacteroides caccae* DSMZ 19024, *Bacteroides eggerthii* DSMZ 20697, *Bacteroides finegoldii* DSMZ 17565, *Bacteroides fragilis* DSMZ 2151, *Bacteroides intestinalis* DSMZ 17393, *Bacteroides ovatus* DSMZ 1896, *Bacteroides thetaiotaomicron* DSMZ 2079, *Bacteroides uniformis* DSMZ 6597, *Bacteroides vulgatus* DSMZ 1447, *Bifidobacterium adolescentis* DSMZ 20083, *Bifidobacterium longum* DSMZ 20088, *Bifidobacterium stercoris* DSMZ 20086, *Blautia wexlerae* DSMZ 19850, *Clostridium perfringens* DSMZ 756, *Clostridium innocuum* DSMZ 1286, *Collinsella aerofaciens*^63^, *Coprococcus comes*^63^, *Escherichia coli* K12 wildtype, *Enterococcus faecium*^63^, *Enterococcus mundtii* DSMZ 4838, *Eubacterium ventriousum*^64^, *Faecalibacterium prausznitzii* DSMZ 107840, *Klebsiella pneumoniae* sp. 40.9b^65^, *Lactobacillus gasseri* DSMZ 20243, *Prevotella copri* DSMZ 18205, *Ruminococcus gnavus* DSMZ 108212, *Ruminococcus lactaris*^64^, *Staphylococcus epidermidis* DSMZ 20042, *Streptococcus anginosus* DSMZ 20563. Strains were inoculated from cryo-culture at −80 °C into 3 mL BHI (OXOID) supplemented with L-cysteine (1 g/L), Vitamin K1 (1 mg/L), Hemin (5 mg/L), yeast extract (5 g/L), and NaHCO_3_ (1 g/L). Cultures were grown overnight at 37 °C in an anaerobic chamber (Coy Labs, USA) under anaerobic conditions (85% N_2_, 10% CO_2_, 5% H_2_). 1 mL of these cell cultures was centrifuged (11,300 × *g* for 2 min) in a 1.5 ml microfuge tube. Supernatant was discarded, and the cells were resuspended in 1 ml of PBS and OD600 was adjusted to 0.002.

### Growth assays

The above-described cultures were used for experiments in diluted rich medium. Where described, either supplemented BHI was diluted 1:5 (v/v) with phosphate-buffered saline (PBS) at a pH of 7.4 or VL6 Medium without glucose (Formedium, England, FOR-VL60201)/80% PBS) supplemented with glucose (10 g/L) and hemin (5 mg/L) diluted 1:5 (v/v) with PBS was used. Catecholamines were stored in their original powder form according to the manufacturers’ recommendations and protected from light. L-3,4-dihydroxyphenylalanine (DOPA), dopamine, and epinephrine were stored at 4 °C, while norepinephrine was stored at −20 °C. For each experiment, catecholamines were freshly weighed and dissolved immediately prior to media preparation to minimize oxidative degradation. Prepared media were transferred into the anaerobic chamber approximately 24 h prior to inoculation to allow for passive oxygen removal before use. L-3,4-dihydroxyphenylalanine (DOPA/Levodopa; pharmaceutical secondary standard, certified reference material; Merck, Germany, Cat. No. PHR1271), dopamine hydrochloride (≥98% TLC; Merck, Germany, Cat. No. H8502), (−)-norepinephrine (≥98% TLC; Merck, Germany, Cat. No. A7257), and (−)-epinephrine (≥99% HPLC; Merck, Germany, Cat. No. E4250) were used in this study.

The neurochemicals were dissolved in diluted rich media and filter sterilized using 0.22 μm Surfactant-Free Cellulose Acetate filters (Sartorius, Austria). Catecholamine concentrations are reported in mass-based units for consistency across compounds; corresponding molar concentrations are as follows: L-3,4-dihydroxyphenylalanine (MW 197.2 g/mol), 1 g/L ≈ 5.1 mM; dopamine hydrochloride (MW 189.6 g/mol), 1 g/L ≈ 5.3 mM; norepinephrine (MW 169.2 g/mol), 1 g/L ≈ 5.9 mM; epinephrine (MW 183.2 g/mol), 1 g/L ≈ 5.4 mM. Mixtures were diluted to the respective concentration. A total of 10 μL of cell culture was added to 190 μL diluted media mixture containing one of the catecholamines. This yielded a final starting OD of 0.0001. Cultures were grown in Greiner flat-bottom 96-well plates (Roth, Germany) anaerobically at 37 °C in a custom-made incubator on plate stacker (EMBL, Germany)^66^. Plates were automatically transferred into a plate reader (Epoch 2, BioTek) that was fully housed within the anaerobic chamber, ensuring that cultures remained under strictly anaerobic conditions throughout the entire 48-h measurement period. Plates were agitated for 10 s to ensure homogeneity; optical density was measured in 20 min intervals throughout 48 h. For each experiment, multiple 96-well plates were prepared sequentially within the anaerobic chamber. Upon completion of preparation, plates were sealed with Breath-Easy membranes (Merck, Germany, Cat. No. Z380059). All plates were loaded into an incubated plate reader equipped with an automated plate stacker housed inside the anaerobic chamber. Automated optical density measurements were then initiated, with each plate measured sequentially in a continuous loop at 20-min intervals. The total elapsed time between inoculation of the first well and acquisition of the first measurement was approximately 30 min, reflecting the time required for plate preparation, transfer, and initiation of automated measurements. All plates, therefore, experienced comparable pre-measurement handling and incubation conditions. Growth measurements were acquired over a 48-h period using a plate reader maintained under anaerobic conditions. This duration was chosen to ensure that all strains had reached the stationary phase.

### Growth parameter calculation

Raw growth data were processed and analyzed in Python (v3.8) using the AMiGA software^67^. Across strains and conditions, growth curves displayed the expected sigmoidal shape, with an initial lag phase, exponential growth, and a plateau corresponding to carrying capacity. Gaussian process (GP) regression was used to fit the growth data. From the fitted models, growth parameters were extracted. The extracted growth parameters and their corresponding AMiGA are AUC (auc_lin), Carrying Capacity (k_lin), Death (death_lin), Growth Rage (gr), Death Rate (dr), Doubling time (td), Lag Time (lagC), Time at Max. Growth Rate (t_gr), Time at Max. Death Rate (t_dr), and Time at Carrying Capacity (t_k). For each growth parameter extracted from AMiGA, values from each experimental condition were normalized to the corresponding values obtained from the control condition. Normalization was performed by subtracting each parameter value in the experimental condition from the value of the same parameter in the control. This yielded values relative to the control, where values > 1 indicate an increase and values < 1 indicate a decrease compared to the control. These normalized values were used for statistical comparison and visualization in subsequent analysis.

### Statistical analysis

All statistical analyses were performed in R (version 4.5.1)^68^. Given the exploratory nature of this screening study, experiments were performed with a limited number of biological replicates (triplicates for the initial high-concentration screen and four biological replicates for the concentration-response experiments). Statistical testing for individual strain-condition pairs was used to identify robust and consistent response patterns rather than to provide definitive hypothesis testing for individual comparisons. Individual p-values are therefore reported descriptively and interpreted in conjunction with effect sizes, consistency across concentrations, and concordant responses across multiple growth parameters, rather than as isolated significance calls requiring formal multiple-testing correction. To assess whether catecholamine exposure exerted significant effects on overall bacterial growth, we performed analysis of variance (ANOVA) using the R base function aov() on the raw area under the growth curve (AUC) values. Separate ANOVA models were applied to the datasets corresponding to Figs. 1 and 3. For the initial high-concentration screen (Fig. 1), AUC values were analyzed with catecholamine treatment and bacterial species as explanatory factors (AUC ~ Species*Catecholamine). For the concentration-response experiments (Fig. 3), AUC values were analyzed with bacterial species, catecholamine identity, and catecholamine concentration as explanatory variables (AUC ~ Species*Catecholamine*Concentration). ANOVA was used here to evaluate global effects across the dataset rather than to test individual strain–condition comparisons.

### Principal components analysis

Principal components analysis (PCA) was performed in R (version 4.5.1)^68^ using the FactoMineR (version 2.12)^69^ and factoextra (version 1.0.7)^70^ packages. Summary statistics (difference between the mean of control and the mean of the treatment) derived from the *t*-tests were used as input variables. PCA was carried out using the prcomp() function (base R function) with data centered and scaled (*z*-scored) to unit variance. The resulting components were visualized as a biplot using the fviz_pca_biplot() function from factoextra, which displays both variable loadings and sample scores on the first two principal components. Individual points were colored based on their species identity. Statistical assessment in group difference in the multivariate space a permutational multivariate analysis of variance (PERMANOVA) was applied using the adonis2() function from the vegan package (version 2.7-1)^71^. The analysis was based on a Euclidean distance matrix with the same input data. Significance was evaluated using 999 permutations. This approach provided a formal test of whether the observed clustering patterns in the PCA reflected significant differences among experimental groups.

### Linear mixed-effects models

Linear mixed-effects model analysis was performed in R using the lme4 package (version 1.1-37)^72^. Differences in calculated growth parameters per species were used as input and fitted with the lmer() function. Fixed effects included the experimental condition. Random effects, i.e., replicate were specified to account for repeated measures. Post hoc pairwise comparisons were performed on the estimated marginal means (EMMs) with Tukey adjustment using the lsmeans package (version 1.11.1)^73^. Model estimates and standard errors were used for plotting with ggplot2.

### Non-metric multidimensional scaling

The estimates obtained from the linear mixed-effects models were used to conduct non-metric multidimensional scaling (NMDS) analysis. Model estimates were first standardized using the scale() function in R to produce a normalized matrix suitable for ordination. NMDS was performed using the metaMDS() function in the vegan package using Manhattan distances. The ordination was visualized as a two-dimensional NMDS plot to illustrate relative differences or similarities among species and catecholamines. Phylum information was used to color the points. Statistical assessment of differences in multivariate composition across phyla was performed using PERMANOVA on the same distance matrix. The adonis2() function from the vegan package was used with 999 permutations to evaluate statistically significant group differences in multivariate space. The ordination and statistical results were interpreted jointly to assess the overall separation of phyla on the observed variation.

### Enzyme-linked immunosorbent assay (ELISA)

Dopamine concentrations were measured using the ImmuSmol Dopamine ELISA kit (Any-Sample; Immusmol, France) according to the manufacturer’s instructions. Fresh sBHI and VL6 were prepared, autoclaved, and diluted in PBS at pH 7.4. Assays were run in 96-well plates supplied with the kit using 10 μL of media. In short, the assay uses a *cis*-diol-specific affinity gel to extract, acylate, and enzymatically convert dopamine. Subsequently, a competitive ELISA is performed using a microtiter plate format. The standard row ranges from 0 to 80 ng/mL of dopamine; the sensitivity (limit of detection) for dopamine in this assay is indicated to be 0.25 ng/mL. Two controls at 3.1 and 9.5 ng/mL, respectively, were included. After the assay, absorbance was read at 450 nm using a plate reader (Epoch 2, BioTek).

Dopamine measurements were performed on media after autoclaving in order to reflect the conditions experienced by bacteria during growth experiments. Autoclaving can promote oxidation of catecholamines and may therefore reduce detectable dopamine levels. As a result, the measured concentrations should be considered conservative estimates of baseline catecholaminergic activity. This approach was chosen intentionally to assess dopamine availability under the exact conditions used for bacterial cultivation. Baseline measurements of dopamine in the growth media were performed to assess whether catecholaminergic activity was present prior to supplementation, rather than to comprehensively quantify all neurochemicals. Dopamine was selected as a representative catecholamine for this purpose, as it can be reliably quantified using established analytical methods and serves as a suitable indicator of catecholaminergic contamination in complex media. Other catecholamines were not directly assayed, and their presence in animal-derived media cannot be excluded. We have therefore treated all experiments conducted in such media as potentially exposed to endogenous catecholamines and interpreted these experiments accordingly. Additionally, we did not assay the media for other hormone classes such as steroid hormones (e.g., corticosteroids or estrogens); however, any such compounds potentially present in the base media would be present at identical levels in both control and catecholamine-supplemented conditions, and therefore do not confound the paired, catecholamine-specific comparisons reported here.

## Supplementary information

Supplementary Tables

## Data Availability

Summary data and statistical analyses are provided in the main manuscript and Supplementary Information. Raw growth curve data and additional datasets generated during the study are available from the corresponding author upon reasonable request.

## Code availability

No custom code was generated for this study. All statistical analyses were performed using standard functions and publicly available packages in R, as described in the “Methods” section.
