# Supplementary material for: Widespread effects of catecholamines on growth of human gut bacteria
Source: NPJ Biofilms Microbiomes. 2026 Mar 20;12:103. doi: 10.1038/s41522-026-00948-2 (PMC13223239; doi:10.1038/s41522-026-00948-2)
Supplement: Supplementary file 1 — Supplementary Tables [file 41522_2026_948_MOESM1_ESM.pdf]

Supplementary Table 1

Species	Comparison	Mean_Baseline	Mean_Treatment	Diff	P_Value	T_Statistic	DF	Lower_CI	Upper_CI	NormDiff	Relative_Difference
Bacteroides caccae	Noradrenaline vs NK	11.312	9.542	-1.770	0.087	-3.165	2.000	-4.177	0.637	-0.232	-15.649
Bacteroides caccae	Adrenaline vs NK	11.312	9.490	-1.822	0.244	-1.632	2.000	-6.627	2.982	-0.238	-16.110
Bacteroides caccae	Levodopa vs NK	11.312	9.059	-2.254	0.003	-17.975	2.000	-2.793	-1.714	-0.295	-19.922
Bacteroides caccae	Dopamine vs NK	11.312	5.608	-5.705	0.013	-8.532	2.000	-8.582	-2.828	-0.746	-50.431
Bacteroides eggerthii	Noradrenaline vs NK	5.963	4.845	-1.118	0.169	-2.114	2.000	-3.394	1.158	-0.146	-18.750
Bacteroides eggerthii	Adrenaline vs NK	5.963	4.810	-1.153	0.179	-2.037	2.000	-3.588	1.282	-0.151	-19.332
Bacteroides eggerthii	Levodopa vs NK	5.963	4.516	-1.447	0.035	-5.226	2.000	-2.638	-0.256	-0.189	-24.259
Bacteroides eggerthii	Dopamine vs NK	5.963	2.671	-3.291	0.010	-9.989	2.000	-4.709	-1.874	-0.431	-55.201
Bacteroides finegoldii	Noradrenaline vs NK	5.099	5.485	0.386	0.472	0.879	2.000	-1.505	2.278	0.051	7.578
Bacteroides finegoldii	Adrenaline vs NK	5.099	5.230	0.131	0.824	0.253	2.000	-2.099	2.362	0.017	2.574
Bacteroides finegoldii	Levodopa vs NK	5.099	4.631	-0.468	0.442	-0.951	2.000	-2.584	1.649	-0.061	-9.175
Bacteroides finegoldii	Dopamine vs NK	5.099	3.478	-1.621	0.133	-2.460	2.000	-4.456	1.214	-0.212	-31.792
Bacteroides fragilis	Adrenaline vs NK	8.189	6.973	-1.216	0.326	-1.292	2.000	-5.264	2.833	-0.159	-14.845
Bacteroides fragilis	Noradrenaline vs NK	8.189	6.961	-1.228	0.106	-2.822	2.000	-3.101	0.645	-0.161	-15.001
Bacteroides fragilis	Levodopa vs NK	8.189	6.178	-2.012	0.017	-7.676	2.000	-3.139	-0.884	-0.263	-24.564
Bacteroides fragilis	Dopamine vs NK	8.189	3.710	-4.479	0.009	-10.385	2.000	-6.335	-2.623	-0.586	-54.694
Bacteroides intestinalis	Noradrenaline vs NK	8.913	9.525	0.613	0.302	1.379	2.000	-1.299	2.524	0.080	6.873
Bacteroides intestinalis	Adrenaline vs NK	8.913	8.154	-0.758	0.408	-1.040	2.000	-3.896	2.379	-0.099	-8.508
Bacteroides intestinalis	Levodopa vs NK	8.913	7.022	-1.890	0.283	-1.455	2.000	-7.479	3.698	-0.247	-21.208
Bacteroides intestinalis	Dopamine vs NK	8.913	5.202	-3.710	0.080	-3.314	2.000	-8.528	1.107	-0.485	-41.631
Bacteroides ovatus	Noradrenaline vs NK	10.327	8.477	-1.850	0.198	-1.897	2.000	-6.046	2.346	-0.242	-17.918
Bacteroides ovatus	Levodopa vs NK	10.327	8.369	-1.958	0.058	-3.959	2.000	-4.086	0.170	-0.256	-18.961
Bacteroides ovatus	Adrenaline vs NK	10.327	8.259	-2.068	0.232	-1.696	2.000	-7.313	3.177	-0.271	-20.024
Bacteroides ovatus	Dopamine vs NK	10.327	4.916	-5.411	0.010	-10.036	2.000	-7.731	-3.091	-0.708	-52.395
Bacteroides thetaiotaomicron	Adrenaline vs NK	11.025	9.863	-1.162	0.239	-1.659	2.000	-4.176	1.851	-0.152	-10.541

Species	Comparison	Mean_Baseline	Mean_Treatment	Diff	P_Value	T_Statistic	DF	Lower_CI	Upper_CI	NormDiff	Relative_Difference
Bacteroides thetaiotaomicron	Noradrenaline vs NK	11.025	9.742	-1.283	0.094	-3.034	2.000	-3.103	0.536	-0.168	-11.638
Bacteroides thetaiotaomicron	Levodopa vs NK	11.025	8.654	-2.371	0.067	-3.675	2.000	-5.146	0.405	-0.310	-21.504
Bacteroides thetaiotaomicron	Dopamine vs NK	11.025	4.475	-6.550	0.029	-5.781	2.000	-11.425	-1.675	-0.857	-59.409
Bacteroides uniformis	Noradrenaline vs NK	5.470	5.281	-0.189	0.876	-0.177	2.000	-4.764	4.387	-0.025	-3.447
Bacteroides uniformis	Adrenaline vs NK	5.470	4.877	-0.592	0.599	-0.620	2.000	-4.704	3.520	-0.077	-10.829
Bacteroides uniformis	Levodopa vs NK	5.470	4.384	-1.085	0.035	-5.239	2.000	-1.977	-0.194	-0.142	-19.844
Bacteroides uniformis	Dopamine vs NK	5.470	1.151	-4.319	0.032	-5.500	2.000	-7.697	-0.940	-0.565	-78.961
Bacteroides vulgatus	Adrenaline vs NK	11.893	9.945	-1.948	0.224	-1.739	2.000	-6.767	2.871	-0.255	-16.377
Bacteroides vulgatus	Noradrenaline vs NK	11.893	9.915	-1.978	0.128	-2.518	2.000	-5.358	1.402	-0.259	-16.632
Bacteroides vulgatus	Levodopa vs NK	11.893	8.790	-3.103	0.013	-8.661	2.000	-4.645	-1.561	-0.406	-26.092
Bacteroides vulgatus	Dopamine vs NK	11.893	4.249	-7.644	0.010	-10.111	2.000	-10.897	-4.391	-1.000	-64.276
Bifidobacterium adolescentis	Dopamine vs NK	2.333	3.670	1.337	0.344	1.229	2.000	-3.345	6.019	0.175	57.297
Bifidobacterium adolescentis	Adrenaline vs NK	2.333	3.358	1.025	0.131	2.488	2.000	-0.748	2.798	0.134	43.940
Bifidobacterium adolescentis	Levodopa vs NK	2.333	2.435	0.102	0.870	0.185	2.000	-2.276	2.480	0.013	4.376
Bifidobacterium adolescentis	Noradrenaline vs NK	2.333	2.236	-0.097	0.955	-0.063	2.000	-6.682	6.489	-0.013	-4.145
Bifidobacterium longum	Adrenaline vs NK	2.323	2.353	0.030	0.923	0.110	2.000	-1.134	1.193	0.004	1.277
Bifidobacterium longum	Levodopa vs NK	2.323	2.050	-0.274	0.326	-1.289	2.000	-1.187	0.640	-0.036	-11.775
Bifidobacterium longum	Noradrenaline vs NK	2.323	1.832	-0.491	0.276	-1.483	2.000	-1.917	0.934	-0.064	-21.148
Bifidobacterium longum	Dopamine vs NK	2.323	1.459	-0.864	0.074	-3.468	2.000	-1.936	0.208	-0.113	-37.181
Bifidobacterium stercoris	Noradrenaline vs NK	10.210	9.529	-0.681	0.234	-1.684	2.000	-2.423	1.060	-0.089	-6.673
Bifidobacterium stercoris	Adrenaline vs NK	10.210	8.866	-1.344	0.373	-1.139	2.000	-6.421	3.733	-0.176	-13.161
Bifidobacterium stercoris	Levodopa vs NK	10.210	7.299	-2.911	0.020	-6.884	2.000	-4.731	-1.092	-0.381	-28.514
Bifidobacterium stercoris	Dopamine vs NK	10.210	4.321	-5.889	0.017	-7.465	2.000	-9.283	-2.495	-0.770	-57.678
Blautia wexlerae	Levodopa vs NK	4.714	8.939	4.225	0.049	4.355	2.000	0.051	8.399	0.553	89.629
Blautia wexlerae	Noradrenaline vs NK	4.714	6.962	2.248	0.104	2.851	2.000	-1.144	5.640	0.294	47.691
Blautia wexlerae	Adrenaline vs NK	4.714	6.211	1.497	0.492	0.834	2.000	-6.223	9.217	0.196	31.761
Blautia wexlerae	Dopamine vs NK	4.714	5.733	1.019	0.179	2.035	2.000	-1.135	3.173	0.133	21.618

Species	Comparison	Mean_Baseline	Mean_Treatment	Diff	P_Value	T_Statistic	DF	Lower_CI	Upper_CI	NormDiff	Relative_Difference
Clostridium innocuum	Noradrenaline vs NK	3.208	3.703	0.495	0.174	2.070	2.000	-0.534	1.524	0.065	15.424
Clostridium innocuum	Levodopa vs NK	3.208	3.611	0.402	0.595	0.627	2.000	-2.358	3.163	0.053	12.544
Clostridium innocuum	Adrenaline vs NK	3.208	3.185	-0.023	0.789	-0.305	2.000	-0.349	0.303	-0.003	-0.719
Clostridium innocuum	Dopamine vs NK	3.208	2.994	-0.214	0.501	-0.814	2.000	-1.346	0.918	-0.028	-6.673
Clostridium perfringens	Noradrenaline vs NK	4.933	7.307	2.374	0.125	2.561	2.000	-1.614	6.363	0.311	48.137
Clostridium perfringens	Levodopa vs NK	4.933	6.637	1.704	0.069	3.602	2.000	-0.332	3.740	0.223	34.548
Clostridium perfringens	Adrenaline vs NK	4.933	6.125	1.192	0.375	1.133	2.000	-3.334	5.717	0.156	24.164
Clostridium perfringens	Dopamine vs NK	4.933	5.887	0.954	0.395	1.076	2.000	-2.863	4.772	0.125	19.350
Collinsella aerofaciens	Adrenaline vs NK	3.582	4.194	0.612	0.450	0.932	2.000	-2.213	3.437	0.080	17.074
Collinsella aerofaciens	Levodopa vs NK	3.582	3.274	-0.308	0.752	-0.362	2.000	-3.968	3.351	-0.040	-8.606
Collinsella aerofaciens	Noradrenaline vs NK	3.582	2.913	-0.669	0.714	-0.423	2.000	-7.482	6.143	-0.088	-18.687
Collinsella aerofaciens	Dopamine vs NK	3.582	1.752	-1.830	0.209	-1.830	2.000	-6.133	2.473	-0.239	-51.086
Coprococcus comes	Adrenaline vs NK	7.545	9.213	1.669	0.004	16.020	2.000	1.220	2.117	0.218	22.116
Coprococcus comes	Noradrenaline vs NK	7.545	7.384	-0.161	0.807	-0.278	2.000	-2.646	2.325	-0.021	-2.128
Coprococcus comes	Levodopa vs NK	7.545	6.864	-0.681	0.521	-0.771	2.000	-4.479	3.117	-0.089	-9.022
Coprococcus comes	Dopamine vs NK	7.545	6.059	-1.486	0.211	-1.816	2.000	-5.007	2.035	-0.194	-19.696
Enterococcus faecium	Noradrenaline vs NK	2.067	6.448	4.381	0.012	9.019	2.000	2.291	6.471	0.573	211.915
Enterococcus faecium	Adrenaline vs NK	2.067	6.104	4.037	0.018	7.254	2.000	1.643	6.432	0.528	195.286
Enterococcus faecium	Dopamine vs NK	2.067	4.946	2.879	0.014	8.505	2.000	1.422	4.335	0.377	139.248
Enterococcus faecium	Levodopa vs NK	2.067	3.970	1.903	0.391	1.086	2.000	-5.639	9.445	0.249	92.053
Enterococcus mundtii	Noradrenaline vs NK	2.287	7.623	5.336	0.009	10.572	2.000	3.164	7.507	0.698	233.264
Enterococcus mundtii	Levodopa vs NK	2.287	6.494	4.206	0.049	4.365	2.000	0.060	8.352	0.550	183.881
Enterococcus mundtii	Dopamine vs NK	2.287	6.480	4.192	0.001	31.860	2.000	3.626	4.758	0.548	183.274
Enterococcus mundtii	Adrenaline vs NK	2.287	6.003	3.716	0.031	5.530	2.000	0.825	6.607	0.486	162.455
Escherichia coli	Noradrenaline vs NK	7.999	9.334	1.335	0.141	2.369	2.000	-1.089	3.759	0.175	16.686
Escherichia coli	Adrenaline vs NK	7.999	8.847	0.848	0.269	1.515	2.000	-1.560	3.256	0.111	10.602
Escherichia coli	Levodopa vs NK	7.999	6.916	-1.083	0.503	-0.810	2.000	-6.834	4.668	-0.142	-13.539

Species	Comparison	Mean_Baseline	Mean_Treatment	Diff	P_Value	T_Statistic	DF	Lower_CI	Upper_CI	NormDiff	Relative_Difference
Escherichia coli	Dopamine vs NK	7.999	6.859	-1.140	0.137	-2.413	2.000	-3.172	0.893	-0.149	-14.250
Eubacterium ventriosum	Adrenaline vs NK	12.957	12.299	-0.658	0.384	-1.105	2.000	-3.219	1.904	-0.086	-5.078
Eubacterium ventriosum	Noradrenaline vs NK	12.957	11.993	-0.964	0.537	-0.738	2.000	-6.582	4.655	-0.126	-7.437
Eubacterium ventriosum	Levodopa vs NK	12.957	11.923	-1.034	0.269	-1.513	2.000	-3.973	1.906	-0.135	-7.978
Eubacterium ventriosum	Dopamine vs NK	12.957	10.995	-1.962	0.124	-2.563	2.000	-5.257	1.332	-0.257	-15.144
Faecalibacterium prausnitzii	Adrenaline vs NK	1.958	1.860	-0.098	0.761	-0.349	2.000	-1.314	1.117	-0.013	-5.029
Faecalibacterium prausnitzii	Noradrenaline vs NK	1.958	-0.380	-2.338	0.002	-25.588	2.000	-2.732	-1.945	-0.306	-119.403
Faecalibacterium prausnitzii	Levodopa vs NK	1.958	-0.834	-2.793	0.003	-19.916	2.000	-3.396	-2.190	-0.365	-142.607
Faecalibacterium prausnitzii	Dopamine vs NK	1.958	-1.320	-3.278	0.003	-17.936	2.000	-4.064	-2.492	-0.429	-167.382
Klebsiella pneumoniae	Noradrenaline vs NK	6.087	10.137	4.050	0.039	4.914	2.000	0.504	7.595	0.530	66.527
Klebsiella pneumoniae	Levodopa vs NK	6.087	9.479	3.391	0.032	5.481	2.000	0.729	6.054	0.444	55.713
Klebsiella pneumoniae	Adrenaline vs NK	6.087	9.417	3.330	0.057	4.022	2.000	-0.233	6.892	0.436	54.704
Klebsiella pneumoniae	Dopamine vs NK	6.087	7.230	1.143	0.246	1.624	2.000	-1.886	4.171	0.149	18.772
Lactobacillus gasseri	Levodopa vs NK	1.001	3.235	2.235	0.037	5.087	2.000	0.345	4.125	0.292	223.258
Lactobacillus gasseri	Dopamine vs NK	1.001	2.502	1.501	0.027	6.000	2.000	0.425	2.578	0.196	149.986
Lactobacillus gasseri	Noradrenaline vs NK	1.001	1.938	0.937	0.160	2.189	2.000	-0.904	2.778	0.123	93.597
Lactobacillus gasseri	Adrenaline vs NK	1.001	1.858	0.857	0.049	4.347	2.000	0.009	1.706	0.112	85.659
Prevotella copri	Levodopa vs NK	3.425	3.911	0.486	0.394	1.079	2.000	-1.454	2.427	0.064	14.204
Prevotella copri	Noradrenaline vs NK	3.425	2.776	-0.649	0.492	-0.835	2.000	-3.990	2.693	-0.085	-18.937
Prevotella copri	Dopamine vs NK	3.425	1.209	-2.216	0.025	-6.185	2.000	-3.757	-0.674	-0.290	-64.694
Prevotella copri	Adrenaline vs NK	3.425	1.083	-2.342	0.033	-5.398	2.000	-4.208	-0.475	-0.306	-68.381
Ruminococcus gnavus	Adrenaline vs NK	5.440	6.066	0.626	0.363	1.168	2.000	-1.680	2.932	0.082	11.508
Ruminococcus gnavus	Noradrenaline vs NK	5.440	5.791	0.350	0.575	0.665	2.000	-1.917	2.618	0.046	6.441
Ruminococcus gnavus	Levodopa vs NK	5.440	5.506	0.066	0.936	0.091	2.000	-3.047	3.179	0.009	1.215
Ruminococcus gnavus	Dopamine vs NK	5.440	3.852	-1.588	0.142	-2.362	2.000	-4.481	1.305	-0.208	-29.188
Ruminococcus lactaris	Adrenaline vs NK	10.590	13.929	3.339	0.022	6.566	2.000	1.151	5.526	0.437	31.525
Ruminococcus lactaris	Noradrenaline vs NK	10.590	13.342	2.752	0.136	2.423	2.000	-2.135	7.639	0.360	25.985

Species	Comparison	Mean_Baseline	Mean_Treatment	Diff	P_Value	T_Statistic	DF	Lower_CI	Upper_CI	NormDiff	Relative_Difference
Ruminococcus lactaris	Levodopa vs NK	10.590	12.050	1.460	0.013	8.692	2.000	0.737	2.183	0.191	13.788
Ruminococcus lactaris	Dopamine vs NK	10.590	8.807	-1.783	0.171	-2.100	2.000	-5.438	1.871	-0.233	-16.840
Staphylococcus epidermidis	Dopamine vs NK	0.960	4.521	3.560	0.101	2.910	2.000	-1.704	8.824	0.466	370.756
Staphylococcus epidermidis	Adrenaline vs NK	0.960	4.177	3.217	0.026	6.080	2.000	0.940	5.493	0.421	334.962
Staphylococcus epidermidis	Levodopa vs NK	0.960	4.161	3.201	0.026	6.051	2.000	0.925	5.476	0.419	333.288
Staphylococcus epidermidis	Noradrenaline vs NK	0.960	4.153	3.193	0.016	7.829	2.000	1.438	4.947	0.418	332.460
Streptococcus anginosus	Levodopa vs NK	1.589	3.665	2.076	0.053	4.173	2.000	-0.065	4.217	0.272	130.669
Streptococcus anginosus	Noradrenaline vs NK	1.589	3.504	1.915	0.128	2.521	2.000	-1.353	5.183	0.250	120.510
Streptococcus anginosus	Dopamine vs NK	1.589	3.282	1.693	0.106	2.820	2.000	-0.891	4.277	0.222	106.567
Streptococcus anginosus	Adrenaline vs NK	1.589	2.994	1.405	0.152	2.264	2.000	-1.265	4.076	0.184	88.440

Supplementary Table 2

Species	Hormon	Concentration	Parameter	Mean_Baseline	Mean_Treatment	Diff	P_Value	T_Statistic	DF	Lower_CI	Upper_CI	Diff_Norm
Bacteroides caccae	Levodopa	0.100000	auc_lin	11.758055	6.790253	-4.967801	0.128808	-2.081578	3.000000	-12.562888	2.627285	-1.000000
Bacteroides caccae	Levodopa	0.010000	auc_lin	11.758055	6.992963	-4.765092	0.155576	-1.887254	3.000000	-12.800391	3.270207	-0.959195
Bacteroides caccae	Levodopa	0.001000	auc_lin	11.758055	7.737948	-4.020107	0.213231	-1.575462	3.000000	-12.140758	4.100544	-0.809233
Bacteroides caccae	Levodopa	0.000100	auc_lin	11.758055	8.473987	-3.284068	0.242552	-1.451505	3.000000	-10.484433	3.916298	-0.661071
Bacteroides caccae	Levodopa	0.000010	auc_lin	11.758055	10.132905	-1.625150	0.645933	-0.508829	3.000000	-11.789565	8.539265	-0.327137
Bacteroides caccae	Levodopa	0.000001	auc_lin	11.758055	10.628929	-1.129126	0.742787	-0.359869	3.000000	-11.114363	8.856111	-0.227289
Bacteroides caccae	Dopamine	0.100000	auc_lin	10.606077	7.618992	-2.987085	0.379595	-1.027976	3.000000	-12.234616	6.260446	-0.601289
Bacteroides caccae	Dopamine	0.010000	auc_lin	10.606077	8.927188	-1.678889	0.549376	-0.672596	3.000000	-9.622701	6.264922	-0.337954
Bacteroides caccae	Dopamine	0.001000	auc_lin	10.606077	9.607369	-0.998708	0.706094	-0.414911	3.000000	-8.658989	6.661573	-0.201036
Bacteroides caccae	Dopamine	0.000100	auc_lin	10.606077	9.863212	-0.742864	0.751561	-0.346920	3.000000	-7.557486	6.071757	-0.149536
Bacteroides caccae	Dopamine	0.000010	auc_lin	10.606077	9.822223	-0.783854	0.729052	-0.380300	3.000000	-7.343346	5.775638	-0.157787
Bacteroides caccae	Dopamine	0.000001	auc_lin	10.606077	9.365700	-1.240377	0.602121	-0.580815	3.000000	-8.036743	5.555989	-0.249683
Bacteroides caccae	Noradrenaline	0.100000	auc_lin	10.924594	8.340420	-2.584174	0.387776	-1.007834	3.000000	-10.744243	5.575896	-0.520185
Bacteroides caccae	Noradrenaline	0.010000	auc_lin	10.924594	8.742993	-2.181601	0.456161	-0.853397	3.000000	-10.317116	5.953915	-0.439148
Bacteroides caccae	Noradrenaline	0.001000	auc_lin	10.924594	10.919660	-0.004934	0.998849	-0.001566	3.000000	-10.034316	10.024449	-0.000993
Bacteroides caccae	Noradrenaline	0.000100	auc_lin	10.924594	8.619302	-2.305292	0.431937	-0.905530	3.000000	-10.407140	5.796557	-0.464047
Bacteroides caccae	Noradrenaline	0.000010	auc_lin	10.924594	8.900824	-2.023770	0.482406	-0.799585	3.000000	-10.078619	6.031080	-0.407377
Bacteroides caccae	Noradrenaline	0.000001	auc_lin	10.924594	10.378541	-0.546053	0.866731	-0.182632	3.000000	-10.061264	8.969159	-0.109918
Bacteroides caccae	Adrenaline	0.100000	auc_lin	11.268790	10.637645	-0.631145	0.802564	-0.273006	3.000000	-7.988447	6.726158	-0.127047
Bacteroides caccae	Adrenaline	0.010000	auc_lin	11.268790	9.127561	-2.141229	0.393658	-0.993598	3.000000	-8.999481	4.717022	-0.431021
Bacteroides caccae	Adrenaline	0.001000	auc_lin	11.268790	8.745012	-2.523778	0.374414	-1.040950	3.000000	-10.239605	5.192049	-0.508027
Bacteroides caccae	Adrenaline	0.000100	auc_lin	11.268790	7.817487	-3.451303	0.287299	-1.290636	3.000000	-11.961514	5.058908	-0.694734
Bacteroides caccae	Adrenaline	0.000010	auc_lin	11.268790	10.130662	-1.138128	0.748409	-0.351564	3.000000	-11.440760	9.164503	-0.229101
Bacteroides caccae	Adrenaline	0.000001	auc_lin	11.268790	8.433541	-2.835249	0.306477	-1.229628	3.000000	-10.173264	4.502765	-0.570725
Bacteroides caccae	Levodopa	0.100000	k_lin	0.382943	0.286513	-0.096430	0.055779	-3.042011	3.000000	-0.197312	0.004452	-0.868482

Species	Hormon	Concentration	Parameter	Mean_Baseline	Mean_Treatment	Diff	P_Value	T_Statistic	DF	Lower_CI	Upper_CI	Diff_Norm
Bacteroides caccae	Levodopa	0.010000	k_lin	0.382943	0.271910	-0.111033	0.065319	-2.846078	3.000000	-0.235189	0.013123	-1.000000
Bacteroides caccae	Levodopa	0.001000	k_lin	0.382943	0.293954	-0.088989	0.092743	-2.437068	3.000000	-0.205196	0.027217	-0.801466
Bacteroides caccae	Levodopa	0.000100	k_lin	0.382943	0.314182	-0.068761	0.090254	-2.467632	3.000000	-0.157442	0.019919	-0.619289
Bacteroides caccae	Levodopa	0.000010	k_lin	0.382943	0.338015	-0.044929	0.356984	-1.085903	3.000000	-0.176600	0.086743	-0.404642
Bacteroides caccae	Levodopa	0.000001	k_lin	0.382943	0.360775	-0.022169	0.655950	-0.492828	3.000000	-0.165323	0.120986	-0.199658
Bacteroides caccae	Dopamine	0.100000	k_lin	0.400233	0.316020	-0.084214	0.204899	-1.614160	3.000000	-0.250248	0.081820	-0.758457
Bacteroides caccae	Dopamine	0.010000	k_lin	0.400233	0.327775	-0.072459	0.228718	-1.507809	3.000000	-0.225393	0.080476	-0.652587
Bacteroides caccae	Dopamine	0.001000	k_lin	0.400233	0.329024	-0.071210	0.193976	-1.667655	3.000000	-0.207101	0.064682	-0.641337
Bacteroides caccae	Dopamine	0.000100	k_lin	0.400233	0.340069	-0.060164	0.219591	-1.547042	3.000000	-0.183929	0.063601	-0.541860
Bacteroides caccae	Dopamine	0.000010	k_lin	0.400233	0.347855	-0.052378	0.255298	-1.402643	3.000000	-0.171218	0.066462	-0.471733
Bacteroides caccae	Dopamine	0.000001	k_lin	0.400233	0.346969	-0.053264	0.256621	-1.397720	3.000000	-0.174540	0.068012	-0.479712
Bacteroides caccae	Noradrenaline	0.100000	k_lin	0.382635	0.340187	-0.042449	0.224057	-1.527625	3.000000	-0.130881	0.045983	-0.382308
Bacteroides caccae	Noradrenaline	0.010000	k_lin	0.382635	0.328484	-0.054152	0.119383	-2.161716	3.000000	-0.133873	0.025570	-0.487709
Bacteroides caccae	Noradrenaline	0.001000	k_lin	0.382635	0.352317	-0.030318	0.531273	-0.705636	3.000000	-0.167056	0.106419	-0.273058
Bacteroides caccae	Noradrenaline	0.000100	k_lin	0.382635	0.331584	-0.051052	0.163044	-1.839965	3.000000	-0.139352	0.037249	-0.459789
Bacteroides caccae	Noradrenaline	0.000010	k_lin	0.382635	0.336925	-0.045710	0.129172	-2.078626	3.000000	-0.115693	0.024273	-0.411679
Bacteroides caccae	Noradrenaline	0.000001	k_lin	0.382635	0.366675	-0.015960	0.716458	-0.399211	3.000000	-0.143190	0.111270	-0.143741
Bacteroides caccae	Adrenaline	0.100000	k_lin	0.382909	0.345331	-0.037578	0.198209	-1.646531	3.000000	-0.110210	0.035053	-0.338440
Bacteroides caccae	Adrenaline	0.010000	k_lin	0.382909	0.317252	-0.065657	0.060942	-2.931190	3.000000	-0.136941	0.005628	-0.591325
Bacteroides caccae	Adrenaline	0.001000	k_lin	0.382909	0.312075	-0.070834	0.102028	-2.331283	3.000000	-0.167529	0.025862	-0.637953
Bacteroides caccae	Adrenaline	0.000100	k_lin	0.382909	0.293339	-0.089569	0.062404	-2.901930	3.000000	-0.187797	0.008658	-0.806692
Bacteroides caccae	Adrenaline	0.000010	k_lin	0.382909	0.351568	-0.031341	0.513785	-0.738412	3.000000	-0.166416	0.103734	-0.282268
Bacteroides caccae	Adrenaline	0.000001	k_lin	0.382909	0.328511	-0.054398	0.106334	-2.286133	3.000000	-0.130124	0.021328	-0.489927
Bacteroides caccae	Levodopa	0.100000	death_lin	0.004399	0.000224	-0.004175	0.246274	-1.436957	3.000000	-0.013423	0.005072	-0.324100
Bacteroides caccae	Levodopa	0.010000	death_lin	0.004399	0.002251	-0.002149	0.595271	-0.592394	3.000000	-0.013692	0.009395	-0.166788
Bacteroides caccae	Levodopa	0.001000	death_lin	0.004399	0.006805	0.002406	0.675744	0.461657	3.000000	-0.014180	0.018991	0.186753
Bacteroides caccae	Levodopa	0.000100	death_lin	0.004399	0.011232	0.006833	0.393944	0.992911	3.000000	-0.015067	0.028733	0.530363

Species	Hormon	Concentration	Parameter	Mean_Baseline	Mean_Treatment	Diff	P_Value	T_Statistic	DF	Lower_CI	Upper_CI	Diff_Norm
Bacteroides caccae	Levodopa	0.000010	death_lin	0.004399	0.006862	0.002463	0.720962	0.392427	3.000000	-0.017509	0.022435	0.191163
Bacteroides caccae	Levodopa	0.000001	death_lin	0.004399	0.006896	0.002496	0.723360	0.388825	3.000000	-0.017934	0.022926	0.193754
Bacteroides caccae	Dopamine	0.100000	death_lin	0.002460	0.004439	0.001979	0.642046	0.515081	3.000000	-0.010246	0.014203	0.153575
Bacteroides caccae	Dopamine	0.010000	death_lin	0.002460	0.014918	0.012458	0.329984	1.159988	3.000000	-0.021722	0.046638	0.967044
Bacteroides caccae	Dopamine	0.001000	death_lin	0.002460	0.015343	0.012883	0.109959	2.249793	3.000000	-0.005341	0.031107	1.000000
Bacteroides caccae	Dopamine	0.000100	death_lin	0.002460	0.008866	0.006406	0.235768	1.478663	3.000000	-0.007381	0.020192	0.497212
Bacteroides caccae	Dopamine	0.000010	death_lin	0.002460	0.001322	-0.001138	0.397842	-0.983593	3.000000	-0.004819	0.002544	-0.088323
Bacteroides caccae	Dopamine	0.000001	death_lin	0.002460	0.000097	-0.002363	0.369813	-1.052617	3.000000	-0.009508	0.004782	-0.183431
Bacteroides caccae	Noradrenaline	0.100000	death_lin	0.007053	0.005028	-0.002025	0.659422	-0.487318	3.000000	-0.015249	0.011199	-0.157180
Bacteroides caccae	Noradrenaline	0.010000	death_lin	0.007053	0.013299	0.006245	0.490246	0.783996	3.000000	-0.019106	0.031597	0.484777
Bacteroides caccae	Noradrenaline	0.001000	death_lin	0.007053	0.014990	0.007937	0.272890	1.339340	3.000000	-0.010922	0.026796	0.616079
Bacteroides caccae	Noradrenaline	0.000100	death_lin	0.007053	0.014584	0.007530	0.427643	0.915044	3.000000	-0.018659	0.033719	0.584501
Bacteroides caccae	Noradrenaline	0.000010	death_lin	0.007053	0.013404	0.006351	0.380869	1.024812	3.000000	-0.013371	0.026073	0.492973
Bacteroides caccae	Noradrenaline	0.000001	death_lin	0.007053	0.001973	-0.005081	0.232911	-1.490357	3.000000	-0.015930	0.005768	-0.394367
Bacteroides caccae	Adrenaline	0.100000	death_lin	0.004432	0.009206	0.004774	0.321226	1.185326	3.000000	-0.008044	0.017593	0.370593
Bacteroides caccae	Adrenaline	0.010000	death_lin	0.004432	0.010734	0.006302	0.188105	1.697832	3.000000	-0.005510	0.018114	0.489144
Bacteroides caccae	Adrenaline	0.001000	death_lin	0.004432	0.012638	0.008206	0.335239	1.145107	3.000000	-0.014601	0.031013	0.636989
Bacteroides caccae	Adrenaline	0.000100	death_lin	0.004432	0.012402	0.007970	0.385504	1.013386	3.000000	-0.017058	0.032997	0.618605
Bacteroides caccae	Adrenaline	0.000010	death_lin	0.004432	0.007256	0.002824	0.685055	0.447186	3.000000	-0.017271	0.022919	0.219179
Bacteroides caccae	Adrenaline	0.000001	death_lin	0.004432	0.005069	0.000637	0.909440	0.123611	3.000000	-0.015773	0.017048	0.049476
Bacteroides caccae	Levodopa	0.100000	gr	0.281631	0.209705	-0.071926	0.149983	-1.924434	3.000000	-0.190870	0.047018	-0.494458
Bacteroides caccae	Levodopa	0.010000	gr	0.281631	0.224449	-0.057182	0.165778	-1.823277	3.000000	-0.156991	0.042627	-0.393102
Bacteroides caccae	Levodopa	0.001000	gr	0.281631	0.244827	-0.036804	0.471553	-0.821523	3.000000	-0.179375	0.105767	-0.253008
Bacteroides caccae	Levodopa	0.000100	gr	0.281631	0.245631	-0.036000	0.394933	-0.990539	3.000000	-0.151662	0.079662	-0.247483
Bacteroides caccae	Levodopa	0.000010	gr	0.281631	0.346100	0.064469	0.463467	0.838153	3.000000	-0.180319	0.309258	0.443197
Bacteroides caccae	Levodopa	0.000001	gr	0.281631	0.360276	0.078645	0.479402	0.805616	3.000000	-0.232028	0.389318	0.540647
Bacteroides caccae	Dopamine	0.100000	gr	0.367298	0.221833	-0.145464	0.292990	-1.272101	3.000000	-0.509376	0.218447	-1.000000

Species	Hormon	Concentration	Parameter	Mean_Baseline	Mean_Treatment	Diff	P_Value	T_Statistic	DF	Lower_CI	Upper_CI	Diff_Norm
Bacteroides caccae	Dopamine	0.010000	gr	0.367298	0.265423	-0.101875	0.472069	-0.820470	3.000000	-0.497027	0.293278	-0.700342
Bacteroides caccae	Dopamine	0.001000	gr	0.367298	0.246142	-0.121156	0.412835	-0.948535	3.000000	-0.527648	0.285337	-0.832892
Bacteroides caccae	Dopamine	0.000100	gr	0.367298	0.279267	-0.088031	0.547545	-0.675898	3.000000	-0.502522	0.326460	-0.605173
Bacteroides caccae	Dopamine	0.000010	gr	0.367298	0.246058	-0.121240	0.387942	-1.007429	3.000000	-0.504234	0.261754	-0.833469
Bacteroides caccae	Dopamine	0.000001	gr	0.367298	0.248634	-0.118664	0.384316	-1.016302	3.000000	-0.490248	0.252920	-0.815760
Bacteroides caccae	Noradrenaline	0.100000	gr	0.272556	0.244105	-0.028451	0.376143	-1.036601	3.000000	-0.115799	0.058896	-0.195590
Bacteroides caccae	Noradrenaline	0.010000	gr	0.272556	0.268050	-0.004506	0.901118	-0.135059	3.000000	-0.110689	0.101677	-0.030979
Bacteroides caccae	Noradrenaline	0.001000	gr	0.272556	0.368891	0.096335	0.507289	0.750817	3.000000	-0.311994	0.504663	0.662257
Bacteroides caccae	Noradrenaline	0.000100	gr	0.272556	0.264371	-0.008185	0.701416	-0.422038	3.000000	-0.069903	0.053534	-0.056267
Bacteroides caccae	Noradrenaline	0.000010	gr	0.272556	0.269414	-0.003142	0.884609	-0.157840	3.000000	-0.066494	0.060210	-0.021600
Bacteroides caccae	Noradrenaline	0.000001	gr	0.272556	0.347257	0.074701	0.493169	0.778240	3.000000	-0.230773	0.380175	0.513535
Bacteroides caccae	Adrenaline	0.100000	gr	0.271812	0.306231	0.034418	0.642899	0.513707	3.000000	-0.178806	0.247643	0.236611
Bacteroides caccae	Adrenaline	0.010000	gr	0.271812	0.241187	-0.030626	0.242550	-1.451515	3.000000	-0.097772	0.036521	-0.210536
Bacteroides caccae	Adrenaline	0.001000	gr	0.271812	0.242881	-0.028931	0.273311	-1.337878	3.000000	-0.097751	0.039889	-0.198890
Bacteroides caccae	Adrenaline	0.000100	gr	0.271812	0.251308	-0.020505	0.339872	-1.132179	3.000000	-0.078141	0.037132	-0.140960
Bacteroides caccae	Adrenaline	0.000010	gr	0.271812	0.354838	0.083026	0.498939	0.766956	3.000000	-0.261486	0.427538	0.570765
Bacteroides caccae	Adrenaline	0.000001	gr	0.271812	0.243440	-0.028372	0.141428	-1.984571	3.000000	-0.073870	0.017125	-0.195046
Bacteroides caccae	Levodopa	0.100000	dr	-0.007768	0.001542	0.009309	0.205243	1.612528	3.000000	-0.009063	0.027682	0.677365
Bacteroides caccae	Levodopa	0.010000	dr	-0.007768	0.001646	0.009414	0.583165	0.613091	3.000000	-0.039452	0.058280	0.684968
Bacteroides caccae	Levodopa	0.001000	dr	-0.007768	-0.006825	0.000943	0.916124	0.114432	3.000000	-0.025291	0.027178	0.068637
Bacteroides caccae	Levodopa	0.000100	dr	-0.007768	-0.002768	0.005000	0.125514	2.108767	3.000000	-0.002546	0.012545	0.363791
Bacteroides caccae	Levodopa	0.000010	dr	-0.007768	-0.006976	0.000792	0.861907	0.189348	3.000000	-0.012515	0.014098	0.057604
Bacteroides caccae	Levodopa	0.000001	dr	-0.007768	-0.006994	0.000774	0.878242	0.166653	3.000000	-0.014007	0.015556	0.056321
Bacteroides caccae	Dopamine	0.100000	dr	0.002206	-0.004637	-0.006843	0.498496	-0.767818	3.000000	-0.035206	0.021520	-0.497907
Bacteroides caccae	Dopamine	0.010000	dr	0.002206	-0.011374	-0.013580	0.189834	-1.688832	3.000000	-0.039170	0.012010	-0.988085
Bacteroides caccae	Dopamine	0.001000	dr	0.002206	-0.011538	-0.013744	0.213585	-1.573858	3.000000	-0.041534	0.014047	-1.000000
Bacteroides caccae	Dopamine	0.000100	dr	0.002206	-0.010855	-0.013061	0.131854	-2.057161	3.000000	-0.033265	0.007144	-0.950300

Species	Hormon	Concentration	Parameter	Mean_Baseline	Mean_Treatment	Diff	P_Value	T_Statistic	DF	Lower_CI	Upper_CI	Diff_Norm
Bacteroides caccae	Dopamine	0.000010	dr	0.002206	-0.000343	-0.002548	0.657866	-0.489785	3.000000	-0.019105	0.014009	-0.185407
Bacteroides caccae	Dopamine	0.000001	dr	0.002206	0.005836	0.003630	0.407279	0.961387	3.000000	-0.008387	0.015647	0.264132
Bacteroides caccae	Noradrenaline	0.100000	dr	-0.017633	-0.010198	0.007435	0.455063	0.855706	3.000000	-0.020217	0.035087	0.540984
Bacteroides caccae	Noradrenaline	0.010000	dr	-0.017633	-0.008767	0.008866	0.224617	1.525218	3.000000	-0.009634	0.027366	0.645109
Bacteroides caccae	Noradrenaline	0.001000	dr	-0.017633	-0.014599	0.003034	0.689558	0.440231	3.000000	-0.018899	0.024967	0.220762
Bacteroides caccae	Noradrenaline	0.000100	dr	-0.017633	-0.013558	0.004075	0.576389	0.624814	3.000000	-0.016681	0.024831	0.296504
Bacteroides caccae	Noradrenaline	0.000010	dr	-0.017633	-0.012354	0.005280	0.615553	0.558377	3.000000	-0.024813	0.035372	0.384170
Bacteroides caccae	Noradrenaline	0.000001	dr	-0.017633	-0.006842	0.010792	0.204784	1.614706	3.000000	-0.010478	0.032062	0.785235
Bacteroides caccae	Adrenaline	0.100000	dr	-0.014610	-0.008528	0.006082	0.206257	1.607732	3.000000	-0.005957	0.018120	0.442505
Bacteroides caccae	Adrenaline	0.010000	dr	-0.014610	-0.015878	-0.001268	0.717990	-0.396901	3.000000	-0.011434	0.008898	-0.092251
Bacteroides caccae	Adrenaline	0.001000	dr	-0.014610	-0.004722	0.009888	0.011479	5.559807	3.000000	0.004228	0.015547	0.719442
Bacteroides caccae	Adrenaline	0.000100	dr	-0.014610	-0.005329	0.009281	0.042497	3.398865	3.000000	0.000591	0.017970	0.675266
Bacteroides caccae	Adrenaline	0.000010	dr	-0.014610	-0.011369	0.003241	0.495491	0.773684	3.000000	-0.010089	0.016570	0.235785
Bacteroides caccae	Adrenaline	0.000001	dr	-0.014610	-0.006526	0.008084	0.122983	2.130239	3.000000	-0.003993	0.020162	0.588219
Bacteroides caccae	Levodopa	0.100000	td	3.377224	6.608223	3.230998	0.090985	2.458543	3.000000	-0.951348	7.413345	1.000000
Bacteroides caccae	Levodopa	0.010000	td	3.377224	5.561415	2.184191	0.068344	2.791319	3.000000	-0.306055	4.674436	0.676011
Bacteroides caccae	Levodopa	0.001000	td	3.377224	5.247478	1.870254	0.178551	1.749301	3.000000	-1.532238	5.272746	0.578847
Bacteroides caccae	Levodopa	0.000100	td	3.377224	4.881740	1.504515	0.264435	1.369204	3.000000	-1.992437	5.001468	0.465650
Bacteroides caccae	Levodopa	0.000010	td	3.377224	3.553776	0.176551	0.847362	0.209663	3.000000	-2.503301	2.856404	0.054643
Bacteroides caccae	Levodopa	0.000001	td	3.377224	3.716011	0.338787	0.747204	0.353340	3.000000	-2.712581	3.390154	0.104855
Bacteroides caccae	Dopamine	0.100000	td	3.335601	5.432094	2.096493	0.085942	2.523107	3.000000	-0.547856	4.740842	0.648868
Bacteroides caccae	Dopamine	0.010000	td	3.335601	3.846886	0.511285	0.649691	0.502808	3.000000	-2.724819	3.747389	0.158244
Bacteroides caccae	Dopamine	0.001000	td	3.335601	5.168646	1.833045	0.199979	1.637847	3.000000	-1.728685	5.394775	0.567331
Bacteroides caccae	Dopamine	0.000100	td	3.335601	4.044706	0.709105	0.541883	0.686167	3.000000	-2.579730	3.997941	0.219469
Bacteroides caccae	Dopamine	0.000010	td	3.335601	5.111751	1.776150	0.126223	2.102846	3.000000	-0.911875	4.464175	0.549722
Bacteroides caccae	Dopamine	0.000001	td	3.335601	5.132357	1.796756	0.099297	2.361140	3.000000	-0.624989	4.218502	0.556099
Bacteroides caccae	Noradrenaline	0.100000	td	3.398029	4.598877	1.200848	0.309282	1.221038	3.000000	-1.928976	4.330672	0.371665

Species	Hormon	Concentration	Parameter	Mean_Baseline	Mean_Treatment	Diff	P_Value	T_Statistic	DF	Lower_CI	Upper_CI	Diff_Norm
Bacteroides caccae	Noradrenaline	0.010000	td	3.398029	4.110192	0.712163	0.322271	1.182267	3.000000	-1.204849	2.629176	0.220416
Bacteroides caccae	Noradrenaline	0.001000	td	3.398029	3.860689	0.462660	0.767675	0.323326	3.000000	-4.091230	5.016551	0.143194
Bacteroides caccae	Noradrenaline	0.000100	td	3.398029	3.637534	0.239505	0.666766	0.475724	3.000000	-1.362711	1.841721	0.074127
Bacteroides caccae	Noradrenaline	0.000010	td	3.398029	3.751867	0.353837	0.331345	1.156111	3.000000	-0.620176	1.327851	0.109513
Bacteroides caccae	Noradrenaline	0.000001	td	3.398029	3.644088	0.246058	0.861294	0.190201	3.000000	-3.870997	4.363114	0.076156
Bacteroides caccae	Adrenaline	0.100000	td	3.792321	4.236848	0.444527	0.727068	0.383267	3.000000	-3.246588	4.135642	0.137582
Bacteroides caccae	Adrenaline	0.010000	td	3.792321	4.958594	1.166273	0.327199	1.167971	3.000000	-2.011547	4.344092	0.360964
Bacteroides caccae	Adrenaline	0.001000	td	3.792321	4.979369	1.187048	0.349115	1.106905	3.000000	-2.225815	4.599910	0.367393
Bacteroides caccae	Adrenaline	0.000100	td	3.792321	4.610218	0.817897	0.239962	1.461777	3.000000	-0.962752	2.598546	0.253141
Bacteroides caccae	Adrenaline	0.000010	td	3.792321	3.580193	-0.212128	0.841409	-0.218010	3.000000	-3.308715	2.884459	-0.065654
Bacteroides caccae	Adrenaline	0.000001	td	3.792321	5.229492	1.437171	0.137598	2.012893	3.000000	-0.835041	3.709384	0.444807
Bacteroides caccae	Levodopa	0.100000	lagC	11.765621	20.067521	8.301901	0.248087	1.429953	3.000000	-10.174472	26.778273	0.970589
Bacteroides caccae	Levodopa	0.010000	lagC	11.765621	18.970858	7.205238	0.371343	1.048720	3.000000	-14.659790	29.070265	0.842376
Bacteroides caccae	Levodopa	0.001000	lagC	11.765621	19.345632	7.580011	0.234230	1.484939	3.000000	-8.665089	23.825111	0.886192
Bacteroides caccae	Levodopa	0.000100	lagC	11.765621	15.344873	3.579252	0.360824	1.075820	3.000000	-7.008749	14.167254	0.418456
Bacteroides caccae	Levodopa	0.000010	lagC	11.765621	15.740497	3.974876	0.587576	0.605514	3.000000	-16.916189	24.865941	0.464709
Bacteroides caccae	Levodopa	0.000001	lagC	11.765621	15.514267	3.748646	0.525992	0.715440	3.000000	-12.926216	20.423509	0.438260
Bacteroides caccae	Dopamine	0.100000	lagC	10.879309	19.432776	8.553467	0.100946	2.342998	3.000000	-3.064534	20.171468	1.000000
Bacteroides caccae	Dopamine	0.010000	lagC	10.879309	17.269884	6.390576	0.108505	2.264195	3.000000	-2.591717	15.372868	0.747133
Bacteroides caccae	Dopamine	0.001000	lagC	10.879309	12.430550	1.551241	0.318621	1.192996	3.000000	-2.586862	5.689345	0.181358
Bacteroides caccae	Dopamine	0.000100	lagC	10.879309	12.936313	2.057004	0.148527	1.934384	3.000000	-1.327178	5.441186	0.240488
Bacteroides caccae	Dopamine	0.000010	lagC	10.879309	11.922869	1.043560	0.498584	0.767647	3.000000	-3.282741	5.369861	0.122004
Bacteroides caccae	Dopamine	0.000001	lagC	10.879309	12.816684	1.937375	0.180251	1.739918	3.000000	-1.606235	5.480985	0.226502
Bacteroides caccae	Noradrenaline	0.100000	lagC	12.467629	18.617961	6.150332	0.221469	1.538825	3.000000	-6.569183	18.869848	0.719046
Bacteroides caccae	Noradrenaline	0.010000	lagC	12.467629	16.916171	4.448542	0.409921	0.955255	3.000000	-10.371840	19.268925	0.520086
Bacteroides caccae	Noradrenaline	0.001000	lagC	12.467629	12.357755	-0.109874	0.978194	-0.029670	3.000000	-11.895287	11.675540	-0.012846
Bacteroides caccae	Noradrenaline	0.000100	lagC	12.467629	17.636284	5.168655	0.381767	1.022588	3.000000	-10.916970	21.254280	0.604276

Species	Hormon	Concentration	Parameter	Mean_Baseline	Mean_Treatment	Diff	P_Value	T_Statistic	DF	Lower_CI	Upper_CI	Diff_Norm
Bacteroides caccae	Noradrenaline	0.000010	lagC	12.467629	17.782423	5.314794	0.338456	1.136111	3.000000	-9.572871	20.202459	0.621361
Bacteroides caccae	Noradrenaline	0.000001	lagC	12.467629	14.744891	2.277263	0.645839	0.508980	3.000000	-11.961536	16.516061	0.266239
Bacteroides caccae	Adrenaline	0.100000	lagC	11.241739	12.886965	1.645226	0.677348	0.459155	3.000000	-9.757983	13.048435	0.192346
Bacteroides caccae	Adrenaline	0.010000	lagC	11.241739	13.245814	2.004075	0.477541	0.809367	3.000000	-5.875983	9.884132	0.234300
Bacteroides caccae	Adrenaline	0.001000	lagC	11.241739	14.518636	3.276897	0.334833	1.146249	3.000000	-5.821080	12.374873	0.383107
Bacteroides caccae	Adrenaline	0.000100	lagC	11.241739	19.344935	8.103196	0.206395	1.607085	3.000000	-7.943243	24.149636	0.947358
Bacteroides caccae	Adrenaline	0.000010	lagC	11.241739	16.411353	5.169614	0.410536	0.953834	3.000000	-12.078693	22.417921	0.604388
Bacteroides caccae	Adrenaline	0.000001	lagC	11.241739	15.283434	4.041694	0.413859	0.946183	3.000000	-9.552368	17.635757	0.472521
Bacteroides caccae	Levodopa	0.100000	t_k	38.333333	47.750000	9.416667	0.190660	1.684567	3.000000	-8.373090	27.206423	0.588542
Bacteroides caccae	Levodopa	0.010000	t_k	38.333333	45.166667	6.833333	0.395784	0.988502	3.000000	-15.166335	28.833002	0.427083
Bacteroides caccae	Levodopa	0.001000	t_k	38.333333	41.083333	2.750000	0.717889	0.397053	3.000000	-19.291722	24.791722	0.171875
Bacteroides caccae	Levodopa	0.000100	t_k	38.333333	36.083333	-2.250000	0.815060	-0.255206	3.000000	-30.307766	25.807766	-0.140625
Bacteroides caccae	Levodopa	0.000010	t_k	38.333333	36.083333	-2.250000	0.873414	-0.173349	3.000000	-43.556867	39.056867	-0.140625
Bacteroides caccae	Levodopa	0.000001	t_k	38.333333	36.833333	-1.500000	0.880472	-0.163564	3.000000	-30.685280	27.685280	-0.093750
Bacteroides caccae	Dopamine	0.100000	t_k	46.833333	41.833333	-5.000000	0.216119	-1.562443	3.000000	-15.184197	5.184197	-0.312500
Bacteroides caccae	Dopamine	0.010000	t_k	46.833333	34.500000	-12.333333	0.093035	-2.433544	3.000000	-28.462147	3.795481	-0.770833
Bacteroides caccae	Dopamine	0.001000	t_k	46.833333	30.833333	-16.000000	0.019260	-4.604612	3.000000	-27.058291	-4.941709	-1.000000
Bacteroides caccae	Dopamine	0.000100	t_k	46.833333	33.000000	-13.833333	0.029756	-3.908317	3.000000	-25.097475	-2.569192	-0.864583
Bacteroides caccae	Dopamine	0.000010	t_k	46.833333	42.916667	-3.916667	0.260308	-1.384150	3.000000	-12.921890	5.088557	-0.244792
Bacteroides caccae	Dopamine	0.000001	t_k	46.833333	47.583333	0.750000	0.586569	0.607240	3.000000	-3.180628	4.680628	0.046875
Bacteroides caccae	Noradrenaline	0.100000	t_k	38.166667	42.250000	4.083333	0.474439	0.815648	3.000000	-11.848776	20.015443	0.255208
Bacteroides caccae	Noradrenaline	0.010000	t_k	38.166667	38.000000	-0.166667	0.984763	-0.020730	3.000000	-25.752998	25.419665	-0.010417
Bacteroides caccae	Noradrenaline	0.001000	t_k	38.166667	34.500000	-3.666667	0.711067	-0.407361	3.000000	-32.311957	24.978624	-0.229167
Bacteroides caccae	Noradrenaline	0.000100	t_k	38.166667	39.250000	1.083333	0.877161	0.168152	3.000000	-19.419858	21.586525	0.067708
Bacteroides caccae	Noradrenaline	0.000010	t_k	38.166667	31.500000	-6.666667	0.587743	-0.605228	3.000000	-41.721762	28.388428	-0.416667
Bacteroides caccae	Noradrenaline	0.000001	t_k	38.166667	44.083333	5.916667	0.405055	0.966576	3.000000	-13.563936	25.397270	0.369792
Bacteroides caccae	Adrenaline	0.100000	t_k	42.250000	40.916667	-1.333333	0.843782	-0.214680	3.000000	-21.098880	18.432214	-0.083333

Species	Hormon	Concentration	Parameter	Mean_Baseline	Mean_Treatment	Diff	P_Value	T_Statistic	DF	Lower_CI	Upper_CI	Diff_Norm
Bacteroides caccae	Adrenaline	0.010000	t_k	42.250000	36.250000	-6.000000	0.499897	-0.765092	3.000000	-30.957360	18.957360	-0.375000
Bacteroides caccae	Adrenaline	0.001000	t_k	42.250000	36.000000	-6.250000	0.548072	-0.674946	3.000000	-35.719467	23.219467	-0.390625
Bacteroides caccae	Adrenaline	0.000100	t_k	42.250000	36.833333	-5.416667	0.590214	-0.601002	3.000000	-34.099172	23.265839	-0.338542
Bacteroides caccae	Adrenaline	0.000010	t_k	42.250000	40.416667	-1.833333	0.833509	-0.229117	3.000000	-27.298428	23.631762	-0.114583
Bacteroides caccae	Adrenaline	0.000001	t_k	42.250000	40.000000	-2.250000	0.807394	-0.266112	3.000000	-29.157847	24.657847	-0.140625
Bacteroides caccae	Levodopa	0.100000	t_gr	17.500000	26.500000	9.000000	0.218184	1.553247	3.000000	-9.440093	27.440093	0.818182
Bacteroides caccae	Levodopa	0.010000	t_gr	17.500000	24.750000	7.250000	0.363326	1.069304	3.000000	-14.327340	28.827340	0.659091
Bacteroides caccae	Levodopa	0.001000	t_gr	17.500000	26.916667	9.416667	0.198600	1.644604	3.000000	-8.805367	27.638700	0.856061
Bacteroides caccae	Levodopa	0.000100	t_gr	17.500000	20.583333	3.083333	0.346840	1.113065	3.000000	-5.732451	11.899118	0.280303
Bacteroides caccae	Levodopa	0.000010	t_gr	17.500000	20.750000	3.250000	0.692349	0.435933	3.000000	-20.475990	26.975990	0.295455
Bacteroides caccae	Levodopa	0.000001	t_gr	17.500000	21.166667	3.666667	0.600581	0.583409	3.000000	-16.334698	23.668032	0.333333
Bacteroides caccae	Dopamine	0.100000	t_gr	14.833333	25.833333	11.000000	0.132782	2.049861	3.000000	-6.077701	28.077701	1.000000
Bacteroides caccae	Dopamine	0.010000	t_gr	14.833333	22.916667	8.083333	0.156853	1.878984	3.000000	-5.607455	21.774121	0.734848
Bacteroides caccae	Dopamine	0.001000	t_gr	14.833333	19.583333	4.750000	0.248562	1.428127	3.000000	-5.834924	15.334924	0.431818
Bacteroides caccae	Dopamine	0.000100	t_gr	14.833333	17.916667	3.083333	0.259895	1.385661	3.000000	-3.998156	10.164823	0.280303
Bacteroides caccae	Dopamine	0.000010	t_gr	14.833333	18.250000	3.416667	0.233911	1.486249	3.000000	-3.899308	10.732641	0.310606
Bacteroides caccae	Dopamine	0.000001	t_gr	14.833333	18.583333	3.750000	0.108696	2.262287	3.000000	-1.525269	9.025269	0.340909
Bacteroides caccae	Noradrenaline	0.100000	t_gr	18.250000	24.583333	6.333333	0.285129	1.297803	3.000000	-9.197140	21.863807	0.575758
Bacteroides caccae	Noradrenaline	0.010000	t_gr	18.250000	22.333333	4.083333	0.473845	0.816856	3.000000	-11.825214	19.991881	0.371212
Bacteroides caccae	Noradrenaline	0.001000	t_gr	18.250000	16.666667	-1.583333	0.749391	-0.350115	3.000000	-15.975378	12.808711	-0.143939
Bacteroides caccae	Noradrenaline	0.000100	t_gr	18.250000	22.583333	4.333333	0.462284	0.840607	3.000000	-12.072193	20.738859	0.393939
Bacteroides caccae	Noradrenaline	0.000010	t_gr	18.250000	24.000000	5.750000	0.296557	1.260673	3.000000	-8.765319	20.265319	0.522727
Bacteroides caccae	Noradrenaline	0.000001	t_gr	18.250000	20.166667	1.916667	0.783537	0.300327	3.000000	-18.393517	22.226850	0.174242
Bacteroides caccae	Adrenaline	0.100000	t_gr	17.750000	18.583333	0.833333	0.886288	0.155518	3.000000	-16.219639	17.886306	0.075758
Bacteroides caccae	Adrenaline	0.010000	t_gr	17.750000	18.666667	0.916667	0.745520	0.355827	3.000000	-7.281808	9.115141	0.083333
Bacteroides caccae	Adrenaline	0.001000	t_gr	17.750000	20.333333	2.583333	0.480878	0.802649	3.000000	-7.659397	12.826063	0.234848
Bacteroides caccae	Adrenaline	0.000100	t_gr	17.750000	25.166667	7.416667	0.254497	1.405633	3.000000	-9.375157	24.208490	0.674242

Species	Hormon	Concentration	Parameter	Mean_Baseline	Mean_Treatment	Diff	P_Value	T_Statistic	DF	Lower_CI	Upper_CI	Diff_Norm
Bacteroides caccae	Adrenaline	0.000010	t_gr	17.750000	21.750000	4.000000	0.595623	0.591796	3.000000	-17.510415	25.510415	0.363636
Bacteroides caccae	Adrenaline	0.000001	t_gr	17.750000	21.083333	3.333333	0.562658	0.648886	3.000000	-13.014930	19.681597	0.303030
Bacteroides caccae	Levodopa	0.100000	t_dr	44.583333	48.000000	3.416667	0.308128	1.224563	3.000000	-5.462713	12.296047	0.362832
Bacteroides caccae	Levodopa	0.010000	t_dr	44.583333	47.500000	2.916667	0.406596	0.962979	3.000000	-6.722317	12.555650	0.309735
Bacteroides caccae	Levodopa	0.001000	t_dr	44.583333	47.333333	2.750000	0.443277	0.880809	3.000000	-7.186006	12.686006	0.292035
Bacteroides caccae	Levodopa	0.000100	t_dr	44.583333	45.833333	1.250000	0.776037	0.311175	3.000000	-11.534000	14.034000	0.132743
Bacteroides caccae	Levodopa	0.000010	t_dr	44.583333	39.500000	-5.083333	0.645281	-0.509876	3.000000	-36.811514	26.644848	-0.539823
Bacteroides caccae	Levodopa	0.000001	t_dr	44.583333	45.916667	1.333333	0.766787	0.324621	3.000000	-11.738105	14.404772	0.141593
Bacteroides caccae	Dopamine	0.100000	t_dr	47.750000	48.000000	0.250000	0.391002	1.000000	3.000000	-0.545612	1.045612	0.026549
Bacteroides caccae	Dopamine	0.010000	t_dr	47.750000	47.750000	0.000000	1.000000	0.000000	3.000000	-1.299228	1.299228	0.000000
Bacteroides caccae	Dopamine	0.001000	t_dr	47.750000	38.333333	-9.416667	0.107574	-2.273530	3.000000	-22.597948	3.764614	-1.000000
Bacteroides caccae	Dopamine	0.000100	t_dr	47.750000	43.416667	-4.333333	0.303888	-1.237628	3.000000	-15.476104	6.809438	-0.460177
Bacteroides caccae	Dopamine	0.000010	t_dr	47.750000	47.333333	-0.416667	0.632833	-0.529999	3.000000	-2.918595	2.085262	-0.044248
Bacteroides caccae	Dopamine	0.000001	t_dr	47.750000	48.000000	0.250000	0.391002	1.000000	3.000000	-0.545612	1.045612	0.026549
Bacteroides caccae	Noradrenaline	0.100000	t_dr	47.666667	47.750000	0.083333	0.391002	1.000000	3.000000	-0.181871	0.348537	0.008850
Bacteroides caccae	Noradrenaline	0.010000	t_dr	47.666667	43.333333	-4.333333	0.283815	-1.302172	3.000000	-14.923793	6.257126	-0.460177
Bacteroides caccae	Noradrenaline	0.001000	t_dr	47.666667	43.416667	-4.250000	0.432948	-0.903304	3.000000	-19.223257	10.723257	-0.451327
Bacteroides caccae	Noradrenaline	0.000100	t_dr	47.666667	44.833333	-2.833333	0.452810	-0.860461	3.000000	-13.312522	7.645855	-0.300885
Bacteroides caccae	Noradrenaline	0.000010	t_dr	47.666667	39.833333	-7.833333	0.414102	-0.945627	3.000000	-34.195895	18.529229	-0.831858
Bacteroides caccae	Noradrenaline	0.000001	t_dr	47.666667	48.000000	0.333333	0.391002	1.000000	3.000000	-0.727482	1.394149	0.035398
Bacteroides caccae	Adrenaline	0.100000	t_dr	47.583333	46.500000	-1.083333	0.391002	-1.000000	3.000000	-4.530983	2.364317	-0.115044
Bacteroides caccae	Adrenaline	0.010000	t_dr	47.583333	45.666667	-1.916667	0.499597	-0.765675	3.000000	-9.883089	6.049756	-0.203540
Bacteroides caccae	Adrenaline	0.001000	t_dr	47.583333	48.000000	0.416667	0.391002	1.000000	3.000000	-0.909353	1.742686	0.044248
Bacteroides caccae	Adrenaline	0.000100	t_dr	47.583333	48.000000	0.416667	0.391002	1.000000	3.000000	-0.909353	1.742686	0.044248
Bacteroides caccae	Adrenaline	0.000010	t_dr	47.583333	47.583333	0.000000	1.000000	0.000000	3.000000	-2.165380	2.165380	0.000000
Bacteroides caccae	Adrenaline	0.000001	t_dr	47.583333	48.000000	0.416667	0.391002	1.000000	3.000000	-0.909353	1.742686	0.044248
Bacteroides finegoldii	Levodopa	0.100000	auc_lin	10.735092	14.476113	3.741021	0.025059	4.172843	3.000000	0.887906	6.594136	0.485290

Species	Hormon	Concentration	Parameter	Mean_Baseline	Mean_Treatment	Diff	P_Value	T_Statistic	DF	Lower_CI	Upper_CI	Diff_Norm
Bacteroides finegoldii	Levodopa	0.010000	auc_lin	10.735092	13.394520	2.659429	0.078009	2.634713	3.000000	-0.552872	5.871729	0.344985
Bacteroides finegoldii	Levodopa	0.001000	auc_lin	10.735092	14.128025	3.392933	0.069183	2.776664	3.000000	-0.495844	7.281711	0.440136
Bacteroides finegoldii	Levodopa	0.000100	auc_lin	10.735092	13.953490	3.218398	0.060181	2.946765	3.000000	-0.257406	6.694202	0.417495
Bacteroides finegoldii	Levodopa	0.000010	auc_lin	10.735092	13.225115	2.490023	0.075289	2.676253	3.000000	-0.470970	5.451016	0.323009
Bacteroides finegoldii	Levodopa	0.000001	auc_lin	10.735092	12.293961	1.558869	0.143920	1.966624	3.000000	-0.963737	4.081476	0.202219
Bacteroides finegoldii	Dopamine	0.100000	auc_lin	7.393675	14.397540	7.003866	0.000834	13.738670	3.000000	5.381480	8.626251	0.908551
Bacteroides finegoldii	Dopamine	0.010000	auc_lin	7.393675	14.243296	6.849621	0.000428	17.198451	3.000000	5.582149	8.117093	0.888542
Bacteroides finegoldii	Dopamine	0.001000	auc_lin	7.393675	15.102508	7.708833	0.000366	18.135208	3.000000	6.356053	9.061613	1.000000
Bacteroides finegoldii	Dopamine	0.000100	auc_lin	7.393675	14.794810	7.401135	0.003253	8.647159	3.000000	4.677268	10.125003	0.960085
Bacteroides finegoldii	Dopamine	0.000010	auc_lin	7.393675	14.160192	6.766517	0.003832	8.172540	3.000000	4.131587	9.401448	0.877762
Bacteroides finegoldii	Dopamine	0.000001	auc_lin	7.393675	12.319966	4.926292	0.041548	3.429682	3.000000	0.355123	9.497461	0.639045
Bacteroides finegoldii	Noradrenaline	0.100000	auc_lin	7.150607	13.175773	6.025166	0.021347	4.431880	3.000000	1.698613	10.351720	0.781592
Bacteroides finegoldii	Noradrenaline	0.010000	auc_lin	7.150607	13.366224	6.215617	0.013356	5.264472	3.000000	2.458190	9.973044	0.806298
Bacteroides finegoldii	Noradrenaline	0.001000	auc_lin	7.150607	13.928693	6.778086	0.011024	5.640955	3.000000	2.954107	10.602065	0.879262
Bacteroides finegoldii	Noradrenaline	0.000100	auc_lin	7.150607	12.806935	5.656328	0.015016	5.045298	3.000000	2.088459	9.224197	0.733746
Bacteroides finegoldii	Noradrenaline	0.000010	auc_lin	7.150607	12.176317	5.025710	0.008233	6.258271	3.000000	2.470044	7.581376	0.651942
Bacteroides finegoldii	Noradrenaline	0.000001	auc_lin	7.150607	9.615079	2.464472	0.170354	1.796042	3.000000	-1.902381	6.831326	0.319695
Bacteroides finegoldii	Adrenaline	0.100000	auc_lin	10.201887	15.399801	5.197914	0.012951	5.323403	3.000000	2.090487	8.305340	0.674280
Bacteroides finegoldii	Adrenaline	0.010000	auc_lin	10.201887	14.301635	4.099747	0.035044	3.668201	3.000000	0.542902	7.656593	0.531825
Bacteroides finegoldii	Adrenaline	0.001000	auc_lin	10.201887	14.665134	4.463246	0.060805	2.933982	3.000000	-0.377970	9.304462	0.578978
Bacteroides finegoldii	Adrenaline	0.000100	auc_lin	10.201887	15.484198	5.282311	0.074221	2.693057	3.000000	-0.959916	11.524538	0.685228
Bacteroides finegoldii	Adrenaline	0.000010	auc_lin	10.201887	14.550918	4.349030	0.074548	2.687886	3.000000	-0.800204	9.498264	0.564162
Bacteroides finegoldii	Adrenaline	0.000001	auc_lin	10.201887	13.200429	2.998541	0.117760	2.176275	3.000000	-1.386334	7.383417	0.388975
Bacteroides finegoldii	Levodopa	0.100000	k_lin	0.343976	0.414531	0.070555	0.032352	3.784160	3.000000	0.011219	0.129891	0.604980
Bacteroides finegoldii	Levodopa	0.010000	k_lin	0.343976	0.375882	0.031905	0.180555	1.738250	3.000000	-0.026508	0.090319	0.273575
Bacteroides finegoldii	Levodopa	0.001000	k_lin	0.343976	0.384969	0.040993	0.154849	1.891997	3.000000	-0.027959	0.109945	0.351496
Bacteroides finegoldii	Levodopa	0.000100	k_lin	0.343976	0.376536	0.032560	0.207978	1.599658	3.000000	-0.032216	0.097336	0.279187

Species	Hormon	Concentration	Parameter	Mean_Baseline	Mean_Treatment	Diff	P_Value	T_Statistic	DF	Lower_CI	Upper_CI	Diff_Norm
Bacteroides finegoldii	Levodopa	0.000010	k_lin	0.343976	0.360061	0.016085	0.435475	0.897756	3.000000	-0.040934	0.073103	0.137919
Bacteroides finegoldii	Levodopa	0.000001	k_lin	0.343976	0.353888	0.009912	0.641111	0.516588	3.000000	-0.051152	0.070976	0.084993
Bacteroides finegoldii	Dopamine	0.100000	k_lin	0.294323	0.410947	0.116624	0.011447	5.565347	3.000000	0.049935	0.183313	1.000000
Bacteroides finegoldii	Dopamine	0.010000	k_lin	0.294323	0.388829	0.094506	0.026955	4.058907	3.000000	0.020407	0.168605	0.810349
Bacteroides finegoldii	Dopamine	0.001000	k_lin	0.294323	0.401012	0.106689	0.009199	6.016938	3.000000	0.050259	0.163118	0.914810
Bacteroides finegoldii	Dopamine	0.000100	k_lin	0.294323	0.403242	0.108919	0.043033	3.381828	3.000000	0.006421	0.211416	0.933930
Bacteroides finegoldii	Dopamine	0.000010	k_lin	0.294323	0.406315	0.111991	0.008793	6.114295	3.000000	0.053701	0.170282	0.960278
Bacteroides finegoldii	Dopamine	0.000001	k_lin	0.294323	0.376522	0.082199	0.089848	2.472707	3.000000	-0.023594	0.187992	0.704822
Bacteroides finegoldii	Noradrenaline	0.100000	k_lin	0.267903	0.366125	0.098222	0.079109	2.618423	3.000000	-0.021158	0.217601	0.842210
Bacteroides finegoldii	Noradrenaline	0.010000	k_lin	0.267903	0.374491	0.106588	0.061181	2.926351	3.000000	-0.009328	0.222505	0.913951
Bacteroides finegoldii	Noradrenaline	0.001000	k_lin	0.267903	0.370894	0.102991	0.063284	2.884742	3.000000	-0.010629	0.216611	0.883107
Bacteroides finegoldii	Noradrenaline	0.000100	k_lin	0.267903	0.342236	0.074333	0.092244	2.443111	3.000000	-0.022495	0.171160	0.637372
Bacteroides finegoldii	Noradrenaline	0.000010	k_lin	0.267903	0.330521	0.062618	0.061807	2.913778	3.000000	-0.005774	0.131010	0.536926
Bacteroides finegoldii	Noradrenaline	0.000001	k_lin	0.267903	0.294234	0.026331	0.509617	0.746356	3.000000	-0.085943	0.138604	0.225774
Bacteroides finegoldii	Adrenaline	0.100000	k_lin	0.330117	0.424197	0.094080	0.022514	4.344497	3.000000	0.025164	0.162996	0.806695
Bacteroides finegoldii	Adrenaline	0.010000	k_lin	0.330117	0.390760	0.060643	0.117667	2.177121	3.000000	-0.028003	0.149290	0.519992
Bacteroides finegoldii	Adrenaline	0.001000	k_lin	0.330117	0.392891	0.062774	0.150375	1.921775	3.000000	-0.041180	0.166728	0.538264
Bacteroides finegoldii	Adrenaline	0.000100	k_lin	0.330117	0.418145	0.088028	0.124040	2.121206	3.000000	-0.044040	0.220097	0.754803
Bacteroides finegoldii	Adrenaline	0.000010	k_lin	0.330117	0.387722	0.057605	0.170657	1.794271	3.000000	-0.044567	0.159777	0.493937
Bacteroides finegoldii	Adrenaline	0.000001	k_lin	0.330117	0.374912	0.044795	0.283875	1.301971	3.000000	-0.064699	0.154289	0.384098
Bacteroides finegoldii	Levodopa	0.100000	death_lin	0.019948	0.008201	-0.011747	0.563479	-0.647434	3.000000	-0.069490	0.045995	-0.308384
Bacteroides finegoldii	Levodopa	0.010000	death_lin	0.019948	0.005034	-0.014914	0.498698	-0.767425	3.000000	-0.076763	0.046934	-0.391530
Bacteroides finegoldii	Levodopa	0.001000	death_lin	0.019948	0.003444	-0.016505	0.467769	-0.829274	3.000000	-0.079844	0.046834	-0.433282
Bacteroides finegoldii	Levodopa	0.000100	death_lin	0.019948	0.003502	-0.016446	0.463771	-0.837524	3.000000	-0.078938	0.046046	-0.431741
Bacteroides finegoldii	Levodopa	0.000010	death_lin	0.019948	0.001876	-0.018073	0.407060	-0.961896	3.000000	-0.077867	0.041721	-0.474445
Bacteroides finegoldii	Levodopa	0.000001	death_lin	0.019948	0.001024	-0.018925	0.395889	-0.988250	3.000000	-0.079868	0.042018	-0.496813
Bacteroides finegoldii	Dopamine	0.100000	death_lin	0.016455	0.000764	-0.015690	0.393967	-0.992855	3.000000	-0.065984	0.034603	-0.411904

Species	Hormon	Concentration	Parameter	Mean_Baseline	Mean_Treatment	Diff	P_Value	T_Statistic	DF	Lower_CI	Upper_CI	Diff_Norm
Bacteroides finegoldii	Dopamine	0.010000	death_lin	0.016455	0.000000	-0.016455	0.391002	-1.000000	3.000000	-0.068820	0.035911	-0.431964
Bacteroides finegoldii	Dopamine	0.001000	death_lin	0.016455	0.001332	-0.015123	0.423273	-0.924817	3.000000	-0.067163	0.036918	-0.397007
Bacteroides finegoldii	Dopamine	0.000100	death_lin	0.016455	0.001695	-0.014759	0.450154	-0.866091	3.000000	-0.068993	0.039474	-0.387465
Bacteroides finegoldii	Dopamine	0.000010	death_lin	0.016455	0.001015	-0.015439	0.426245	-0.918160	3.000000	-0.068954	0.038075	-0.405316
Bacteroides finegoldii	Dopamine	0.000001	death_lin	0.016455	0.000520	-0.015935	0.408771	-0.957920	3.000000	-0.068875	0.037005	-0.418324
Bacteroides finegoldii	Noradrenaline	0.100000	death_lin	0.038092	0.000000	-0.038092	0.215929	-1.563293	3.000000	-0.115638	0.039454	-1.000000
Bacteroides finegoldii	Noradrenaline	0.010000	death_lin	0.038092	0.003449	-0.034644	0.233134	-1.489440	3.000000	-0.108666	0.039379	-0.909465
Bacteroides finegoldii	Noradrenaline	0.001000	death_lin	0.038092	0.001388	-0.036705	0.239738	-1.462669	3.000000	-0.116567	0.043157	-0.963574
Bacteroides finegoldii	Noradrenaline	0.000100	death_lin	0.038092	0.000344	-0.037748	0.221689	-1.537863	3.000000	-0.115864	0.040368	-0.990961
Bacteroides finegoldii	Noradrenaline	0.000010	death_lin	0.038092	0.003506	-0.034586	0.277142	-1.324690	3.000000	-0.117677	0.048504	-0.907962
Bacteroides finegoldii	Noradrenaline	0.000001	death_lin	0.038092	0.016497	-0.021595	0.249483	-1.424599	3.000000	-0.069837	0.026647	-0.566918
Bacteroides finegoldii	Adrenaline	0.100000	death_lin	0.001178	0.013603	0.012425	0.323708	1.178074	3.000000	-0.021140	0.045990	0.326182
Bacteroides finegoldii	Adrenaline	0.010000	death_lin	0.001178	0.007600	0.006422	0.281507	1.309897	3.000000	-0.009181	0.022026	0.168599
Bacteroides finegoldii	Adrenaline	0.001000	death_lin	0.001178	0.003717	0.002540	0.393644	0.993630	3.000000	-0.005594	0.010673	0.066668
Bacteroides finegoldii	Adrenaline	0.000100	death_lin	0.001178	0.002711	0.001533	0.379528	1.028144	3.000000	-0.003212	0.006278	0.040246
Bacteroides finegoldii	Adrenaline	0.000010	death_lin	0.001178	0.000952	-0.000226	0.418962	-0.934547	3.000000	-0.000995	0.000543	-0.005927
Bacteroides finegoldii	Adrenaline	0.000001	death_lin	0.001178	0.002728	0.001550	0.321913	1.183314	3.000000	-0.002618	0.005718	0.040686
Bacteroides finegoldii	Levodopa	0.100000	gr	0.495256	0.435247	-0.060009	0.001517	-11.221149	3.000000	-0.077028	-0.042989	-0.766544
Bacteroides finegoldii	Levodopa	0.010000	gr	0.495256	0.469116	-0.026140	0.036984	-3.591488	3.000000	-0.049303	-0.002977	-0.333912
Bacteroides finegoldii	Levodopa	0.001000	gr	0.495256	0.489451	-0.005805	0.688078	-0.442514	3.000000	-0.047551	0.035942	-0.074150
Bacteroides finegoldii	Levodopa	0.000100	gr	0.495256	0.520442	0.025186	0.062923	2.891751	3.000000	-0.002532	0.052903	0.321719
Bacteroides finegoldii	Levodopa	0.000010	gr	0.495256	0.493558	-0.001698	0.872579	-0.174507	3.000000	-0.032657	0.029262	-0.021686
Bacteroides finegoldii	Levodopa	0.000001	gr	0.495256	0.514367	0.019111	0.064729	2.857127	3.000000	-0.002176	0.040398	0.244124
Bacteroides finegoldii	Dopamine	0.100000	gr	0.491958	0.472369	-0.019589	0.202751	-1.624423	3.000000	-0.057966	0.018788	-0.250228
Bacteroides finegoldii	Dopamine	0.010000	gr	0.491958	0.504242	0.012285	0.638600	0.520644	3.000000	-0.062807	0.087377	0.156927
Bacteroides finegoldii	Dopamine	0.001000	gr	0.491958	0.517460	0.025503	0.299438	1.251549	3.000000	-0.039346	0.090351	0.325768
Bacteroides finegoldii	Dopamine	0.000100	gr	0.491958	0.560426	0.068469	0.148575	1.934054	3.000000	-0.044195	0.181132	0.874612

Species	Hormon	Concentration	Parameter	Mean_Baseline	Mean_Treatment	Diff	P_Value	T_Statistic	DF	Lower_CI	Upper_CI	Diff_Norm
Bacteroides finegoldii	Dopamine	0.000010	gr	0.491958	0.557796	0.065838	0.175546	1.766146	3.000000	-0.052797	0.184473	0.841010
Bacteroides finegoldii	Dopamine	0.000001	gr	0.491958	0.527732	0.035774	0.080008	2.605307	3.000000	-0.007925	0.079474	0.456980
Bacteroides finegoldii	Noradrenaline	0.100000	gr	0.441064	0.476211	0.035147	0.404997	0.966712	3.000000	-0.080559	0.150853	0.448968
Bacteroides finegoldii	Noradrenaline	0.010000	gr	0.441064	0.510919	0.069856	0.262179	1.377341	3.000000	-0.091551	0.231263	0.892331
Bacteroides finegoldii	Noradrenaline	0.001000	gr	0.441064	0.512498	0.071434	0.110311	2.246342	3.000000	-0.029768	0.172637	0.912494
Bacteroides finegoldii	Noradrenaline	0.000100	gr	0.441064	0.519348	0.078285	0.135967	2.025236	3.000000	-0.044731	0.201300	1.000000
Bacteroides finegoldii	Noradrenaline	0.000010	gr	0.441064	0.507444	0.066380	0.068246	2.793048	3.000000	-0.009255	0.142015	0.847936
Bacteroides finegoldii	Noradrenaline	0.000001	gr	0.441064	0.437431	-0.003633	0.907809	-0.125853	3.000000	-0.095495	0.088229	-0.046405
Bacteroides finegoldii	Adrenaline	0.100000	gr	0.497943	0.502489	0.004547	0.454936	0.855973	3.000000	-0.012357	0.021450	0.058077
Bacteroides finegoldii	Adrenaline	0.010000	gr	0.497943	0.502927	0.004984	0.756559	0.339576	3.000000	-0.041726	0.051694	0.063666
Bacteroides finegoldii	Adrenaline	0.001000	gr	0.497943	0.538758	0.040815	0.040169	3.476097	3.000000	0.003448	0.078182	0.521368
Bacteroides finegoldii	Adrenaline	0.000100	gr	0.497943	0.526706	0.028763	0.118566	2.169011	3.000000	-0.013439	0.070965	0.367418
Bacteroides finegoldii	Adrenaline	0.000010	gr	0.497943	0.529702	0.031760	0.026300	4.097045	3.000000	0.007090	0.056430	0.405696
Bacteroides finegoldii	Adrenaline	0.000001	gr	0.497943	0.525125	0.027182	0.089692	2.474671	3.000000	-0.007774	0.062139	0.347224
Bacteroides finegoldii	Levodopa	0.100000	dr	-0.014923	-0.021362	-0.006439	0.174224	-1.773659	3.000000	-0.017993	0.005115	-0.280580
Bacteroides finegoldii	Levodopa	0.010000	dr	-0.014923	-0.002613	0.012310	0.389793	1.002928	3.000000	-0.026751	0.051371	0.536375
Bacteroides finegoldii	Levodopa	0.001000	dr	-0.014923	-0.009007	0.005916	0.694281	0.432963	3.000000	-0.037569	0.049401	0.257779
Bacteroides finegoldii	Levodopa	0.000100	dr	-0.014923	-0.005140	0.009783	0.379722	1.027661	3.000000	-0.020512	0.040077	0.426253
Bacteroides finegoldii	Levodopa	0.000010	dr	-0.014923	-0.008476	0.006447	0.468819	0.827118	3.000000	-0.018358	0.031251	0.280905
Bacteroides finegoldii	Levodopa	0.000001	dr	-0.014923	-0.008907	0.006015	0.659358	0.487420	3.000000	-0.033260	0.045291	0.262111
Bacteroides finegoldii	Dopamine	0.100000	dr	0.011929	-0.009354	-0.021283	0.431706	-0.906039	3.000000	-0.096037	0.053472	-0.927347
Bacteroides finegoldii	Dopamine	0.010000	dr	0.011929	0.007783	-0.004146	0.871726	-0.175692	3.000000	-0.079248	0.070956	-0.180659
Bacteroides finegoldii	Dopamine	0.001000	dr	0.011929	-0.010661	-0.022590	0.524814	-0.717639	3.000000	-0.122767	0.077587	-0.984306
Bacteroides finegoldii	Dopamine	0.000100	dr	0.011929	0.005388	-0.006541	0.889233	-0.151448	3.000000	-0.143989	0.130907	-0.285009
Bacteroides finegoldii	Dopamine	0.000010	dr	0.011929	-0.005582	-0.017511	0.638367	-0.521021	3.000000	-0.124468	0.089447	-0.762997
Bacteroides finegoldii	Dopamine	0.000001	dr	0.011929	-0.008306	-0.020235	0.557553	-0.657947	3.000000	-0.118108	0.077639	-0.881683
Bacteroides finegoldii	Noradrenaline	0.100000	dr	-0.023521	-0.000571	0.022950	0.412908	0.948367	3.000000	-0.054064	0.099964	1.000000

Species	Hormon	Concentration	Parameter	Mean_Baseline	Mean_Treatment	Diff	P_Value	T_Statistic	DF	Lower_CI	Upper_CI	Diff_Norm
Bacteroides finegoldii	Noradrenaline	0.010000	dr	-0.023521	-0.022682	0.000839	0.965860	0.046465	3.000000	-0.056601	0.058278	0.036542
Bacteroides finegoldii	Noradrenaline	0.001000	dr	-0.023521	-0.002942	0.020578	0.529670	0.708603	3.000000	-0.071843	0.112999	0.896665
Bacteroides finegoldii	Noradrenaline	0.000100	dr	-0.023521	-0.003851	0.019670	0.513381	0.739179	3.000000	-0.065017	0.104357	0.857086
Bacteroides finegoldii	Noradrenaline	0.000010	dr	-0.023521	-0.010708	0.012813	0.653364	0.496943	3.000000	-0.069243	0.094870	0.558313
Bacteroides finegoldii	Noradrenaline	0.000001	dr	-0.023521	-0.017880	0.005640	0.805517	0.268789	3.000000	-0.061142	0.072423	0.245771
Bacteroides finegoldii	Adrenaline	0.100000	dr	-0.004728	-0.005151	-0.000423	0.979183	-0.028323	3.000000	-0.048004	0.047157	-0.018451
Bacteroides finegoldii	Adrenaline	0.010000	dr	-0.004728	-0.021602	-0.016874	0.300634	-1.247786	3.000000	-0.059911	0.026163	-0.735250
Bacteroides finegoldii	Adrenaline	0.001000	dr	-0.004728	-0.010151	-0.005423	0.585540	-0.609006	3.000000	-0.033763	0.022917	-0.236309
Bacteroides finegoldii	Adrenaline	0.000100	dr	-0.004728	0.001904	0.006632	0.372031	1.046975	3.000000	-0.013527	0.026790	0.288968
Bacteroides finegoldii	Adrenaline	0.000010	dr	-0.004728	-0.009300	-0.004572	0.558569	-0.656140	3.000000	-0.026749	0.017605	-0.199230
Bacteroides finegoldii	Adrenaline	0.000001	dr	-0.004728	-0.007796	-0.003069	0.351034	-1.101740	3.000000	-0.011933	0.005795	-0.133710
Bacteroides finegoldii	Levodopa	0.100000	td	1.399955	1.592920	0.192965	0.001510	11.239691	3.000000	0.138328	0.247602	0.692599
Bacteroides finegoldii	Levodopa	0.010000	td	1.399955	1.479651	0.079696	0.039613	3.495355	3.000000	0.007134	0.152257	0.286048
Bacteroides finegoldii	Levodopa	0.001000	td	1.399955	1.418394	0.018439	0.665757	0.477313	3.000000	-0.104500	0.141377	0.066181
Bacteroides finegoldii	Levodopa	0.000100	td	1.399955	1.332534	-0.067421	0.064170	-2.867726	3.000000	-0.142242	0.007399	-0.241992
Bacteroides finegoldii	Levodopa	0.000010	td	1.399955	1.407913	0.007958	0.805189	0.269257	3.000000	-0.086101	0.102018	0.028563
Bacteroides finegoldii	Levodopa	0.000001	td	1.399955	1.347875	-0.052080	0.065747	-2.838142	3.000000	-0.110478	0.006318	-0.186928
Bacteroides finegoldii	Dopamine	0.100000	td	1.409182	1.469026	0.059844	0.201611	1.629916	3.000000	-0.057002	0.176689	0.214793
Bacteroides finegoldii	Dopamine	0.010000	td	1.409182	1.381674	-0.027509	0.704605	-0.417176	3.000000	-0.237361	0.182344	-0.098736
Bacteroides finegoldii	Dopamine	0.001000	td	1.409182	1.344984	-0.064198	0.324503	-1.175764	3.000000	-0.237963	0.109567	-0.230423
Bacteroides finegoldii	Dopamine	0.000100	td	1.409182	1.252459	-0.156723	0.104680	-2.303206	3.000000	-0.373275	0.059828	-0.562518
Bacteroides finegoldii	Dopamine	0.000010	td	1.409182	1.255797	-0.153385	0.142871	-1.974127	3.000000	-0.400655	0.093884	-0.550538
Bacteroides finegoldii	Dopamine	0.000001	td	1.409182	1.314982	-0.094201	0.074383	-2.690488	3.000000	-0.205626	0.017225	-0.338110
Bacteroides finegoldii	Noradrenaline	0.100000	td	1.613459	1.455757	-0.157703	0.400571	-0.977122	3.000000	-0.671333	0.355928	-0.566033
Bacteroides finegoldii	Noradrenaline	0.010000	td	1.613459	1.364727	-0.248732	0.300952	-1.246787	3.000000	-0.883624	0.386161	-0.892759
Bacteroides finegoldii	Noradrenaline	0.001000	td	1.613459	1.354309	-0.259150	0.179465	-1.744241	3.000000	-0.731981	0.213681	-0.930152
Bacteroides finegoldii	Noradrenaline	0.000100	td	1.613459	1.334849	-0.278610	0.195467	-1.660152	3.000000	-0.812695	0.255475	-1.000000

Species	Hormon	Concentration	Parameter	Mean_Baseline	Mean_Treatment	Diff	P_Value	T_Statistic	DF	Lower_CI	Upper_CI	Diff_Norm
Bacteroides finegoldii	Noradrenaline	0.000010	td	1.613459	1.371129	-0.242330	0.144682	-1.961203	3.000000	-0.635559	0.150899	-0.869782
Bacteroides finegoldii	Noradrenaline	0.000001	td	1.613459	1.754389	0.140930	0.553609	0.664992	3.000000	-0.533517	0.815376	0.505831
Bacteroides finegoldii	Adrenaline	0.100000	td	1.392460	1.379654	-0.012805	0.449682	-0.867096	3.000000	-0.059805	0.034194	-0.045962
Bacteroides finegoldii	Adrenaline	0.010000	td	1.392460	1.380064	-0.012395	0.774753	-0.313036	3.000000	-0.138413	0.113622	-0.044490
Bacteroides finegoldii	Adrenaline	0.001000	td	1.392460	1.287548	-0.104912	0.037904	-3.556894	3.000000	-0.198780	-0.011045	-0.376555
Bacteroides finegoldii	Adrenaline	0.000100	td	1.392460	1.317083	-0.075377	0.114297	-2.208163	3.000000	-0.184011	0.033258	-0.270545
Bacteroides finegoldii	Adrenaline	0.000010	td	1.392460	1.308841	-0.083619	0.027173	-4.046487	3.000000	-0.149383	-0.017855	-0.300128
Bacteroides finegoldii	Adrenaline	0.000001	td	1.392460	1.320468	-0.071991	0.089169	-2.481281	3.000000	-0.164326	0.020343	-0.258395
Bacteroides finegoldii	Levodopa	0.100000	lagC	2.450488	2.654446	0.203958	0.006612	6.760649	3.000000	0.107949	0.299968	0.823676
Bacteroides finegoldii	Levodopa	0.010000	lagC	2.450488	2.625324	0.174836	0.011814	5.502595	3.000000	0.073719	0.275954	0.706069
Bacteroides finegoldii	Levodopa	0.001000	lagC	2.450488	2.586739	0.136252	0.058344	2.985408	3.000000	-0.008993	0.281496	0.550246
Bacteroides finegoldii	Levodopa	0.000100	lagC	2.450488	2.616921	0.166433	0.029013	3.946403	3.000000	0.032219	0.300648	0.672133
Bacteroides finegoldii	Levodopa	0.000010	lagC	2.450488	2.533291	0.082804	0.327843	1.166117	3.000000	-0.143175	0.308782	0.334398
Bacteroides finegoldii	Levodopa	0.000001	lagC	2.450488	2.549994	0.099506	0.053929	3.084886	3.000000	-0.003147	0.202159	0.401851
Bacteroides finegoldii	Dopamine	0.100000	lagC	2.358138	2.481453	0.123315	0.013978	5.178432	3.000000	0.047531	0.199098	0.498000
Bacteroides finegoldii	Dopamine	0.010000	lagC	2.358138	2.355122	-0.003017	0.938651	-0.083586	3.000000	-0.117874	0.111841	-0.012183
Bacteroides finegoldii	Dopamine	0.001000	lagC	2.358138	2.389238	0.031100	0.538797	0.691797	3.000000	-0.111968	0.174168	0.125596
Bacteroides finegoldii	Dopamine	0.000100	lagC	2.358138	2.452608	0.094470	0.051959	3.132634	3.000000	-0.001502	0.190442	0.381512
Bacteroides finegoldii	Dopamine	0.000010	lagC	2.358138	2.434813	0.076675	0.208270	1.598296	3.000000	-0.075997	0.229347	0.309649
Bacteroides finegoldii	Dopamine	0.000001	lagC	2.358138	2.381075	0.022937	0.053616	3.092333	3.000000	-0.000668	0.046542	0.092630
Bacteroides finegoldii	Noradrenaline	0.100000	lagC	2.585433	2.671953	0.086519	0.282572	1.306325	3.000000	-0.124258	0.297296	0.349404
Bacteroides finegoldii	Noradrenaline	0.010000	lagC	2.585433	2.618140	0.032707	0.692874	0.435126	3.000000	-0.206506	0.271919	0.132085
Bacteroides finegoldii	Noradrenaline	0.001000	lagC	2.585433	2.629107	0.043674	0.673014	0.465921	3.000000	-0.254638	0.341985	0.176375
Bacteroides finegoldii	Noradrenaline	0.000100	lagC	2.585433	2.833053	0.247619	0.122918	2.130802	3.000000	-0.122211	0.617450	1.000000
Bacteroides finegoldii	Noradrenaline	0.000010	lagC	2.585433	2.721193	0.135759	0.105435	2.295378	3.000000	-0.052465	0.323983	0.548257
Bacteroides finegoldii	Noradrenaline	0.000001	lagC	2.585433	2.720341	0.134907	0.277336	1.324026	3.000000	-0.189358	0.459172	0.544818
Bacteroides finegoldii	Adrenaline	0.100000	lagC	2.487887	2.700799	0.212912	0.015935	4.937103	3.000000	0.075669	0.350155	0.859837

Species	Hormon	Concentration	Parameter	Mean_Baseline	Mean_Treatment	Diff	P_Value	T_Statistic	DF	Lower_CI	Upper_CI	Diff_Norm
Bacteroides finegoldii	Adrenaline	0.010000	lagC	2.487887	2.661153	0.173266	0.053491	3.095318	3.000000	-0.004877	0.351409	0.699727
Bacteroides finegoldii	Adrenaline	0.001000	lagC	2.487887	2.664729	0.176842	0.015119	5.032806	3.000000	0.065018	0.288667	0.714170
Bacteroides finegoldii	Adrenaline	0.000100	lagC	2.487887	2.641287	0.153400	0.026193	4.103382	3.000000	0.034428	0.272371	0.619498
Bacteroides finegoldii	Adrenaline	0.000010	lagC	2.487887	2.645282	0.157395	0.054122	3.080334	3.000000	-0.005218	0.320007	0.635631
Bacteroides finegoldii	Adrenaline	0.000001	lagC	2.487887	2.575227	0.087340	0.090690	2.462202	3.000000	-0.025549	0.200228	0.352718
Bacteroides finegoldii	Levodopa	0.100000	t_k	36.083333	45.750000	9.666667	0.428036	0.914170	3.000000	-23.985324	43.318657	0.846715
Bacteroides finegoldii	Levodopa	0.010000	t_k	36.083333	42.583333	6.500000	0.549754	0.671915	3.000000	-24.286502	37.286502	0.569343
Bacteroides finegoldii	Levodopa	0.001000	t_k	36.083333	44.250000	8.166667	0.521801	0.723278	3.000000	-27.766907	44.100240	0.715328
Bacteroides finegoldii	Levodopa	0.000100	t_k	36.083333	45.166667	9.083333	0.466572	0.831738	3.000000	-25.671866	43.838533	0.795620
Bacteroides finegoldii	Levodopa	0.000010	t_k	36.083333	45.666667	9.583333	0.437036	0.894344	3.000000	-24.518143	43.684810	0.839416
Bacteroides finegoldii	Levodopa	0.000001	t_k	36.083333	46.583333	10.500000	0.391037	0.999916	3.000000	-22.918492	43.918492	0.919708
Bacteroides finegoldii	Dopamine	0.100000	t_k	37.333333	46.750000	9.416667	0.447092	0.872619	3.000000	-24.925953	43.759286	0.824818
Bacteroides finegoldii	Dopamine	0.010000	t_k	37.333333	48.000000	10.666667	0.391002	1.000000	3.000000	-23.279427	44.612761	0.934307
Bacteroides finegoldii	Dopamine	0.001000	t_k	37.333333	46.750000	9.416667	0.400659	0.976913	3.000000	-21.259599	40.092932	0.824818
Bacteroides finegoldii	Dopamine	0.000100	t_k	37.333333	47.583333	10.250000	0.412954	0.948260	3.000000	-24.149915	44.649915	0.897810
Bacteroides finegoldii	Dopamine	0.000010	t_k	37.333333	47.250000	9.916667	0.431245	0.907059	3.000000	-24.876287	44.709621	0.868613
Bacteroides finegoldii	Dopamine	0.000001	t_k	37.333333	47.916667	10.583333	0.395332	0.989584	3.000000	-23.452080	44.618747	0.927007
Bacteroides finegoldii	Noradrenaline	0.100000	t_k	36.583333	48.000000	11.416667	0.349646	1.105474	3.000000	-21.449714	44.283048	1.000000
Bacteroides finegoldii	Noradrenaline	0.010000	t_k	36.583333	46.250000	9.666667	0.427661	0.915004	3.000000	-23.954656	43.287990	0.846715
Bacteroides finegoldii	Noradrenaline	0.001000	t_k	36.583333	46.166667	9.583333	0.451409	0.863426	3.000000	-25.739259	44.905925	0.839416
Bacteroides finegoldii	Noradrenaline	0.000100	t_k	36.583333	43.833333	7.250000	0.600999	0.582704	3.000000	-32.345981	46.845981	0.635036
Bacteroides finegoldii	Noradrenaline	0.000010	t_k	36.583333	43.750000	7.166667	0.606272	0.573844	3.000000	-32.578536	46.911869	0.627737
Bacteroides finegoldii	Noradrenaline	0.000001	t_k	36.583333	31.166667	-5.416667	0.246531	-1.435960	3.000000	-17.421353	6.588020	-0.474453
Bacteroides finegoldii	Adrenaline	0.100000	t_k	46.083333	43.833333	-2.250000	0.228912	-1.506993	3.000000	-7.001518	2.501518	-0.197080
Bacteroides finegoldii	Adrenaline	0.010000	t_k	46.083333	44.166667	-1.916667	0.162480	-1.843444	3.000000	-5.225521	1.392188	-0.167883
Bacteroides finegoldii	Adrenaline	0.001000	t_k	46.083333	45.416667	-0.666667	0.399441	-0.979796	3.000000	-2.832047	1.498714	-0.058394
Bacteroides finegoldii	Adrenaline	0.000100	t_k	46.083333	46.000000	-0.083333	0.717686	-0.397360	3.000000	-0.750748	0.584082	-0.007299

Species	Hormon	Concentration	Parameter	Mean_Baseline	Mean_Treatment	Diff	P_Value	T_Statistic	DF	Lower_CI	Upper_CI	Diff_Norm
Bacteroides finegoldii	Adrenaline	0.000010	t_k	46.083333	46.250000	0.166667	0.391002	1.000000	3.000000	-0.363741	0.697074	0.014599
Bacteroides finegoldii	Adrenaline	0.000001	t_k	46.083333	45.750000	-0.333333	0.391002	-1.000000	3.000000	-1.394149	0.727482	-0.029197
Bacteroides finegoldii	Levodopa	0.100000	t_gr	3.583333	3.750000	0.166667	0.181690	1.732051	3.000000	-0.139564	0.472898	0.666667
Bacteroides finegoldii	Levodopa	0.010000	t_gr	3.583333	3.833333	0.250000	0.057669	3.000000	3.000000	-0.015204	0.515204	1.000000
Bacteroides finegoldii	Levodopa	0.001000	t_gr	3.583333	3.750000	0.166667	0.181690	1.732051	3.000000	-0.139564	0.472898	0.666667
Bacteroides finegoldii	Levodopa	0.000100	t_gr	3.583333	3.833333	0.250000	0.057669	3.000000	3.000000	-0.015204	0.515204	1.000000
Bacteroides finegoldii	Levodopa	0.000010	t_gr	3.583333	3.750000	0.166667	0.181690	1.732051	3.000000	-0.139564	0.472898	0.666667
Bacteroides finegoldii	Levodopa	0.000001	t_gr	3.583333	3.583333	0.000000	NA	NA	3.000000	NA	NA	0.000000
Bacteroides finegoldii	Dopamine	0.100000	t_gr	3.583333	3.583333	0.000000	NA	NA	3.000000	NA	NA	0.000000
Bacteroides finegoldii	Dopamine	0.010000	t_gr	3.583333	3.500000	-0.083333	0.391002	-1.000000	3.000000	-0.348537	0.181871	-0.333333
Bacteroides finegoldii	Dopamine	0.001000	t_gr	3.583333	3.583333	0.000000	NA	NA	3.000000	NA	NA	0.000000
Bacteroides finegoldii	Dopamine	0.000100	t_gr	3.583333	3.583333	0.000000	NA	NA	3.000000	NA	NA	0.000000
Bacteroides finegoldii	Dopamine	0.000010	t_gr	3.583333	3.583333	0.000000	NA	NA	3.000000	NA	NA	0.000000
Bacteroides finegoldii	Dopamine	0.000001	t_gr	3.583333	3.583333	0.000000	NA	NA	3.000000	NA	NA	0.000000
Bacteroides finegoldii	Noradrenaline	0.100000	t_gr	3.666667	3.833333	0.166667	0.181690	1.732051	3.000000	-0.139564	0.472898	0.666667
Bacteroides finegoldii	Noradrenaline	0.010000	t_gr	3.666667	3.750000	0.083333	0.391002	1.000000	3.000000	-0.181871	0.348537	0.333333
Bacteroides finegoldii	Noradrenaline	0.001000	t_gr	3.666667	3.750000	0.083333	0.391002	1.000000	3.000000	-0.181871	0.348537	0.333333
Bacteroides finegoldii	Noradrenaline	0.000100	t_gr	3.666667	3.833333	0.166667	0.181690	1.732051	3.000000	-0.139564	0.472898	0.666667
Bacteroides finegoldii	Noradrenaline	0.000010	t_gr	3.666667	3.666667	0.000000	NA	NA	3.000000	NA	NA	0.000000
Bacteroides finegoldii	Noradrenaline	0.000001	t_gr	3.666667	3.833333	0.166667	0.181690	1.732051	3.000000	-0.139564	0.472898	0.666667
Bacteroides finegoldii	Adrenaline	0.100000	t_gr	3.583333	3.833333	0.250000	0.057669	3.000000	3.000000	-0.015204	0.515204	1.000000
Bacteroides finegoldii	Adrenaline	0.010000	t_gr	3.583333	3.833333	0.250000	0.057669	3.000000	3.000000	-0.015204	0.515204	1.000000
Bacteroides finegoldii	Adrenaline	0.001000	t_gr	3.583333	3.750000	0.166667	0.181690	1.732051	3.000000	-0.139564	0.472898	0.666667
Bacteroides finegoldii	Adrenaline	0.000100	t_gr	3.583333	3.750000	0.166667	0.181690	1.732051	3.000000	-0.139564	0.472898	0.666667
Bacteroides finegoldii	Adrenaline	0.000010	t_gr	3.583333	3.750000	0.166667	0.181690	1.732051	3.000000	-0.139564	0.472898	0.666667
Bacteroides finegoldii	Adrenaline	0.000001	t_gr	3.583333	3.750000	0.166667	0.181690	1.732051	3.000000	-0.139564	0.472898	0.666667
Bacteroides finegoldii	Levodopa	0.100000	t_dr	37.583333	48.000000	10.416667	0.391002	1.000000	3.000000	-22.733816	43.567149	0.968992

Species	Hormon	Concentration	Parameter	Mean_Baseline	Mean_Treatment	Diff	P_Value	T_Statistic	DF	Lower_CI	Upper_CI	Diff_Norm
Bacteroides finegoldii	Levodopa	0.010000	t_dr	37.583333	47.583333	10.000000	0.413628	0.946713	3.000000	-23.615744	43.615744	0.930233
Bacteroides finegoldii	Levodopa	0.001000	t_dr	37.583333	48.000000	10.416667	0.391002	1.000000	3.000000	-22.733816	43.567149	0.968992
Bacteroides finegoldii	Levodopa	0.000100	t_dr	37.583333	48.000000	10.416667	0.391002	1.000000	3.000000	-22.733816	43.567149	0.968992
Bacteroides finegoldii	Levodopa	0.000010	t_dr	37.583333	48.000000	10.416667	0.391002	1.000000	3.000000	-22.733816	43.567149	0.968992
Bacteroides finegoldii	Levodopa	0.000001	t_dr	37.583333	48.000000	10.416667	0.391002	1.000000	3.000000	-22.733816	43.567149	0.968992
Bacteroides finegoldii	Dopamine	0.100000	t_dr	37.583333	47.250000	9.666667	0.432511	0.904265	3.000000	-24.353934	43.687267	0.899225
Bacteroides finegoldii	Dopamine	0.010000	t_dr	37.583333	48.000000	10.416667	0.391002	1.000000	3.000000	-22.733816	43.567149	0.968992
Bacteroides finegoldii	Dopamine	0.001000	t_dr	37.583333	47.166667	9.583333	0.391002	1.000000	3.000000	-20.915110	40.081777	0.891473
Bacteroides finegoldii	Dopamine	0.000100	t_dr	37.583333	48.000000	10.416667	0.391002	1.000000	3.000000	-22.733816	43.567149	0.968992
Bacteroides finegoldii	Dopamine	0.000010	t_dr	37.583333	48.000000	10.416667	0.391002	1.000000	3.000000	-22.733816	43.567149	0.968992
Bacteroides finegoldii	Dopamine	0.000001	t_dr	37.583333	48.000000	10.416667	0.391002	1.000000	3.000000	-22.733816	43.567149	0.968992
Bacteroides finegoldii	Noradrenaline	0.100000	t_dr	37.250000	48.000000	10.750000	0.369383	1.053714	3.000000	-21.717351	43.217351	1.000000
Bacteroides finegoldii	Noradrenaline	0.010000	t_dr	37.250000	48.000000	10.750000	0.369383	1.053714	3.000000	-21.717351	43.217351	1.000000
Bacteroides finegoldii	Noradrenaline	0.001000	t_dr	37.250000	48.000000	10.750000	0.369383	1.053714	3.000000	-21.717351	43.217351	1.000000
Bacteroides finegoldii	Noradrenaline	0.000100	t_dr	37.250000	44.416667	7.166667	0.590718	0.600143	3.000000	-30.836840	45.170173	0.666667
Bacteroides finegoldii	Noradrenaline	0.000010	t_dr	37.250000	45.083333	7.833333	0.546390	0.677986	3.000000	-28.936076	44.602743	0.728682
Bacteroides finegoldii	Noradrenaline	0.000001	t_dr	37.250000	33.500000	-3.750000	0.310085	-1.218592	3.000000	-13.543410	6.043410	-0.348837
Bacteroides finegoldii	Adrenaline	0.100000	t_dr	48.000000	44.500000	-3.500000	0.235139	-1.481226	3.000000	-11.019827	4.019827	-0.325581
Bacteroides finegoldii	Adrenaline	0.010000	t_dr	48.000000	47.500000	-0.500000	0.391002	-1.000000	3.000000	-2.091223	1.091223	-0.046512
Bacteroides finegoldii	Adrenaline	0.001000	t_dr	48.000000	48.000000	0.000000	NA	NA	3.000000	NA	NA	0.000000
Bacteroides finegoldii	Adrenaline	0.000100	t_dr	48.000000	48.000000	0.000000	NA	NA	3.000000	NA	NA	0.000000
Bacteroides finegoldii	Adrenaline	0.000010	t_dr	48.000000	48.000000	0.000000	NA	NA	3.000000	NA	NA	0.000000
Bacteroides finegoldii	Adrenaline	0.000001	t_dr	48.000000	48.000000	0.000000	NA	NA	3.000000	NA	NA	0.000000
Bacteroides ovatus	Levodopa	0.100000	auc_lin	12.093286	7.075850	-5.017436	0.113126	-2.219216	3.000000	-12.212643	2.177772	-1.000000
Bacteroides ovatus	Levodopa	0.010000	auc_lin	12.093286	7.167289	-4.925997	0.120954	-2.147842	3.000000	-12.224819	2.372826	-0.981776
Bacteroides ovatus	Levodopa	0.001000	auc_lin	12.093286	7.512103	-4.581183	0.137004	-2.017369	3.000000	-11.808105	2.645739	-0.913053
Bacteroides ovatus	Levodopa	0.000100	auc_lin	12.093286	7.934360	-4.158926	0.166873	-1.816680	3.000000	-11.444500	3.126649	-0.828895

Species	Hormon	Concentration	Parameter	Mean_Baseline	Mean_Treatment	Diff	P_Value	T_Statistic	DF	Lower_CI	Upper_CI	Diff_Norm
Bacteroides ovatus	Levodopa	0.000010	auc_lin	12.093286	8.064215	-4.029071	0.174940	-1.769580	3.000000	-11.275028	3.216887	-0.803014
Bacteroides ovatus	Levodopa	0.000001	auc_lin	12.093286	8.129536	-3.963750	0.184774	-1.715433	3.000000	-11.317240	3.389741	-0.789995
Bacteroides ovatus	Dopamine	0.100000	auc_lin	9.295726	6.606305	-2.689421	0.062787	-2.894406	3.000000	-5.646482	0.267641	-0.536015
Bacteroides ovatus	Dopamine	0.010000	auc_lin	9.295726	7.405304	-1.890422	0.138481	-2.006280	3.000000	-4.889090	1.108245	-0.376771
Bacteroides ovatus	Dopamine	0.001000	auc_lin	9.295726	7.827631	-1.468095	0.246110	-1.437592	3.000000	-4.718065	1.781876	-0.292599
Bacteroides ovatus	Dopamine	0.000100	auc_lin	9.295726	7.848124	-1.447602	0.273102	-1.338605	3.000000	-4.889182	1.993978	-0.288514
Bacteroides ovatus	Dopamine	0.000010	auc_lin	9.295726	8.068237	-1.227489	0.340489	-1.130470	3.000000	-4.683058	2.228080	-0.244645
Bacteroides ovatus	Dopamine	0.000001	auc_lin	9.295726	8.079406	-1.216320	0.328110	-1.165352	3.000000	-4.537954	2.105315	-0.242419
Bacteroides ovatus	Noradrenaline	0.100000	auc_lin	9.469057	9.876171	0.407115	0.904884	0.129875	3.000000	-9.568766	10.382995	0.081140
Bacteroides ovatus	Noradrenaline	0.010000	auc_lin	9.469057	9.519900	0.050844	0.980752	0.026188	3.000000	-6.127837	6.229525	0.010133
Bacteroides ovatus	Noradrenaline	0.001000	auc_lin	9.469057	8.052683	-1.416373	0.234329	-1.484535	3.000000	-4.452698	1.619952	-0.282290
Bacteroides ovatus	Noradrenaline	0.000100	auc_lin	9.469057	8.108867	-1.360190	0.258066	-1.392378	3.000000	-4.469066	1.748687	-0.271093
Bacteroides ovatus	Noradrenaline	0.000010	auc_lin	9.469057	7.969015	-1.500042	0.206934	-1.604549	3.000000	-4.475210	1.475126	-0.298966
Bacteroides ovatus	Noradrenaline	0.000001	auc_lin	9.469057	8.248359	-1.220697	0.254402	-1.405989	3.000000	-3.983736	1.542342	-0.243291
Bacteroides ovatus	Adrenaline	0.100000	auc_lin	9.302395	8.104774	-1.197621	0.368246	-1.056621	3.000000	-4.804747	2.409505	-0.238692
Bacteroides ovatus	Adrenaline	0.010000	auc_lin	9.302395	8.119300	-1.183094	0.403134	-0.971081	3.000000	-5.060355	2.694166	-0.235797
Bacteroides ovatus	Adrenaline	0.001000	auc_lin	9.302395	7.922154	-1.380241	0.325627	-1.172508	3.000000	-5.126521	2.366039	-0.275089
Bacteroides ovatus	Adrenaline	0.000100	auc_lin	9.302395	7.652596	-1.649798	0.249902	-1.423000	3.000000	-5.339464	2.039867	-0.328813
Bacteroides ovatus	Adrenaline	0.000010	auc_lin	9.302395	7.817208	-1.485187	0.291382	-1.277299	3.000000	-5.185595	2.215221	-0.296005
Bacteroides ovatus	Adrenaline	0.000001	auc_lin	9.302395	7.996305	-1.306089	0.294286	-1.267931	3.000000	-4.584310	1.972132	-0.260310
Bacteroides ovatus	Levodopa	0.100000	k_lin	0.375540	0.258937	-0.116602	0.143765	-1.967723	3.000000	-0.305186	0.071981	-1.000000
Bacteroides ovatus	Levodopa	0.010000	k_lin	0.375540	0.263846	-0.111694	0.175913	-1.764069	3.000000	-0.313193	0.089806	-0.957903
Bacteroides ovatus	Levodopa	0.001000	k_lin	0.375540	0.270271	-0.105269	0.197271	-1.651164	3.000000	-0.308164	0.097626	-0.902803
Bacteroides ovatus	Levodopa	0.000100	k_lin	0.375540	0.278339	-0.097201	0.228362	-1.509308	3.000000	-0.302154	0.107752	-0.833611
Bacteroides ovatus	Levodopa	0.000010	k_lin	0.375540	0.279805	-0.095735	0.235911	-1.478084	3.000000	-0.301860	0.110391	-0.821035
Bacteroides ovatus	Levodopa	0.000001	k_lin	0.375540	0.281570	-0.093970	0.247018	-1.434075	3.000000	-0.302506	0.114565	-0.805903
Bacteroides ovatus	Dopamine	0.100000	k_lin	0.311902	0.271303	-0.040599	0.152073	-1.910357	3.000000	-0.108232	0.027034	-0.348180

Species	Hormon	Concentration	Parameter	Mean_Baseline	Mean_Treatment	Diff	P_Value	T_Statistic	DF	Lower_CI	Upper_CI	Diff_Norm
Bacteroides ovatus	Dopamine	0.010000	k_lin	0.311902	0.285517	-0.026385	0.312634	-1.210875	3.000000	-0.095732	0.042961	-0.226285
Bacteroides ovatus	Dopamine	0.001000	k_lin	0.311902	0.292770	-0.019132	0.535702	-0.697471	3.000000	-0.106430	0.068165	-0.164081
Bacteroides ovatus	Dopamine	0.000100	k_lin	0.311902	0.297077	-0.014825	0.568016	-0.639441	3.000000	-0.088611	0.058960	-0.127145
Bacteroides ovatus	Dopamine	0.000010	k_lin	0.311902	0.290342	-0.021560	0.343537	-1.122077	3.000000	-0.082708	0.039589	-0.184901
Bacteroides ovatus	Dopamine	0.000001	k_lin	0.311902	0.286630	-0.025272	0.259492	-1.387137	3.000000	-0.083253	0.032709	-0.216739
Bacteroides ovatus	Noradrenaline	0.100000	k_lin	0.308947	0.351492	0.042545	0.587290	0.606004	3.000000	-0.180880	0.265969	0.364870
Bacteroides ovatus	Noradrenaline	0.010000	k_lin	0.308947	0.327505	0.018558	0.727898	0.382025	3.000000	-0.136042	0.173158	0.159159
Bacteroides ovatus	Noradrenaline	0.001000	k_lin	0.308947	0.287388	-0.021559	0.374227	-1.041420	3.000000	-0.087440	0.044322	-0.184892
Bacteroides ovatus	Noradrenaline	0.000100	k_lin	0.308947	0.292312	-0.016635	0.445591	-0.875835	3.000000	-0.077081	0.043811	-0.142666
Bacteroides ovatus	Noradrenaline	0.000010	k_lin	0.308947	0.289163	-0.019784	0.347790	-1.110488	3.000000	-0.076479	0.036912	-0.169667
Bacteroides ovatus	Noradrenaline	0.000001	k_lin	0.308947	0.289520	-0.019427	0.338156	-1.136946	3.000000	-0.073806	0.034952	-0.166609
Bacteroides ovatus	Adrenaline	0.100000	k_lin	0.302558	0.276312	-0.026245	0.296295	-1.261506	3.000000	-0.092455	0.039964	-0.225082
Bacteroides ovatus	Adrenaline	0.010000	k_lin	0.302558	0.277310	-0.025247	0.318586	-1.193102	3.000000	-0.092592	0.042097	-0.216526
Bacteroides ovatus	Adrenaline	0.001000	k_lin	0.302558	0.285548	-0.017010	0.482445	-0.799508	3.000000	-0.084718	0.050698	-0.145880
Bacteroides ovatus	Adrenaline	0.000100	k_lin	0.302558	0.282007	-0.020551	0.387647	-1.008148	3.000000	-0.085423	0.044322	-0.176245
Bacteroides ovatus	Adrenaline	0.000010	k_lin	0.302558	0.290682	-0.011876	0.701141	-0.422459	3.000000	-0.101338	0.077587	-0.101849
Bacteroides ovatus	Adrenaline	0.000001	k_lin	0.302558	0.287387	-0.015170	0.557474	-0.658088	3.000000	-0.088531	0.058191	-0.130101
Bacteroides ovatus	Levodopa	0.100000	death_lin	0.041152	0.031343	-0.009809	0.423779	-0.923679	3.000000	-0.043606	0.023987	-0.217156
Bacteroides ovatus	Levodopa	0.010000	death_lin	0.041152	0.041245	0.000093	0.995928	0.005539	3.000000	-0.053381	0.053567	0.002060
Bacteroides ovatus	Levodopa	0.001000	death_lin	0.041152	0.043968	0.002816	0.882704	0.160474	3.000000	-0.053024	0.058655	0.062334
Bacteroides ovatus	Levodopa	0.000100	death_lin	0.041152	0.038508	-0.002644	0.890963	-0.149059	3.000000	-0.059101	0.053812	-0.058539
Bacteroides ovatus	Levodopa	0.000010	death_lin	0.041152	0.039721	-0.001431	0.937981	-0.084502	3.000000	-0.055333	0.052470	-0.031684
Bacteroides ovatus	Levodopa	0.000001	death_lin	0.041152	0.033212	-0.007940	0.651560	-0.499821	3.000000	-0.058495	0.042615	-0.175775
Bacteroides ovatus	Dopamine	0.100000	death_lin	0.008260	0.035822	0.027562	0.279730	1.315890	3.000000	-0.039096	0.094221	0.610174
Bacteroides ovatus	Dopamine	0.010000	death_lin	0.008260	0.053378	0.045118	0.086711	2.512960	3.000000	-0.012020	0.102256	0.998818
Bacteroides ovatus	Dopamine	0.001000	death_lin	0.008260	0.053431	0.045171	0.118039	2.173751	3.000000	-0.020961	0.111304	1.000000
Bacteroides ovatus	Dopamine	0.000100	death_lin	0.008260	0.049909	0.041649	0.093440	2.428688	3.000000	-0.012926	0.096224	0.922023

Species	Hormon	Concentration	Parameter	Mean_Baseline	Mean_Treatment	Diff	P_Value	T_Statistic	DF	Lower_CI	Upper_CI	Diff_Norm
Bacteroides ovatus	Dopamine	0.000010	death_lin	0.008260	0.041918	0.033658	0.109560	2.253724	3.000000	-0.013870	0.081187	0.745130
Bacteroides ovatus	Dopamine	0.000001	death_lin	0.008260	0.016615	0.008355	0.287665	1.289432	3.000000	-0.012267	0.028977	0.184972
Bacteroides ovatus	Noradrenaline	0.100000	death_lin	0.018072	0.061876	0.043804	0.045539	3.305692	3.000000	0.001633	0.085975	0.969729
Bacteroides ovatus	Noradrenaline	0.010000	death_lin	0.018072	0.055893	0.037821	0.118432	2.170219	3.000000	-0.017640	0.093281	0.837270
Bacteroides ovatus	Noradrenaline	0.001000	death_lin	0.018072	0.049404	0.031332	0.125627	2.107823	3.000000	-0.015974	0.078638	0.693626
Bacteroides ovatus	Noradrenaline	0.000100	death_lin	0.018072	0.045211	0.027139	0.186565	1.705922	3.000000	-0.023490	0.077768	0.600808
Bacteroides ovatus	Noradrenaline	0.000010	death_lin	0.018072	0.047368	0.029296	0.137050	2.017021	3.000000	-0.016927	0.075519	0.648556
Bacteroides ovatus	Noradrenaline	0.000001	death_lin	0.018072	0.033184	0.015112	0.335799	1.143534	3.000000	-0.026945	0.057170	0.334559
Bacteroides ovatus	Adrenaline	0.100000	death_lin	0.021876	0.039340	0.017464	0.112381	2.226317	3.000000	-0.007500	0.042428	0.386613
Bacteroides ovatus	Adrenaline	0.010000	death_lin	0.021876	0.040126	0.018250	0.130043	2.071596	3.000000	-0.009786	0.046285	0.404009
Bacteroides ovatus	Adrenaline	0.001000	death_lin	0.021876	0.046600	0.024724	0.173535	1.777604	3.000000	-0.019539	0.068987	0.547334
Bacteroides ovatus	Adrenaline	0.000100	death_lin	0.021876	0.045241	0.023365	0.177503	1.755138	3.000000	-0.019001	0.065731	0.517253
Bacteroides ovatus	Adrenaline	0.000010	death_lin	0.021876	0.046154	0.024278	0.266982	1.360101	3.000000	-0.032529	0.081085	0.537466
Bacteroides ovatus	Adrenaline	0.000001	death_lin	0.021876	0.032471	0.010594	0.480816	0.802772	3.000000	-0.031405	0.052593	0.234535
Bacteroides ovatus	Levodopa	0.100000	gr	0.279620	0.193137	-0.086483	0.438824	-0.890449	3.000000	-0.395571	0.222605	-0.735663
Bacteroides ovatus	Levodopa	0.010000	gr	0.279620	0.190512	-0.089108	0.434565	-0.899750	3.000000	-0.404286	0.226070	-0.757994
Bacteroides ovatus	Levodopa	0.001000	gr	0.279620	0.162062	-0.117558	0.355034	-1.091064	3.000000	-0.460453	0.225338	-1.000000
Bacteroides ovatus	Levodopa	0.000100	gr	0.279620	0.186502	-0.093118	0.495256	-0.774145	3.000000	-0.475917	0.289682	-0.792103
Bacteroides ovatus	Levodopa	0.000010	gr	0.279620	0.184698	-0.094921	0.477693	-0.809060	3.000000	-0.468296	0.278453	-0.807446
Bacteroides ovatus	Levodopa	0.000001	gr	0.279620	0.178140	-0.101479	0.446687	-0.873487	3.000000	-0.471207	0.268249	-0.863230
Bacteroides ovatus	Dopamine	0.100000	gr	0.181865	0.215455	0.033590	0.441137	0.885431	3.000000	-0.087140	0.154319	0.285730
Bacteroides ovatus	Dopamine	0.010000	gr	0.181865	0.213455	0.031590	0.357759	1.083860	3.000000	-0.061165	0.124345	0.268720
Bacteroides ovatus	Dopamine	0.001000	gr	0.181865	0.200951	0.019086	0.623758	0.544836	3.000000	-0.092397	0.130568	0.162353
Bacteroides ovatus	Dopamine	0.000100	gr	0.181865	0.226824	0.044959	0.224619	1.525208	3.000000	-0.048851	0.138768	0.382441
Bacteroides ovatus	Dopamine	0.000010	gr	0.181865	0.237236	0.055370	0.098547	2.369514	3.000000	-0.018996	0.129737	0.471006
Bacteroides ovatus	Dopamine	0.000001	gr	0.181865	0.183055	0.001189	0.957227	0.058230	3.000000	-0.063814	0.066193	0.010118
Bacteroides ovatus	Noradrenaline	0.100000	gr	0.189500	0.302407	0.112907	0.386688	1.010488	3.000000	-0.242684	0.468497	0.960437

Species	Hormon	Concentration	Parameter	Mean_Baseline	Mean_Treatment	Diff	P_Value	T_Statistic	DF	Lower_CI	Upper_CI	Diff_Norm
Bacteroides ovatus	Noradrenaline	0.010000	gr	0.189500	0.265213	0.075712	0.355369	1.090175	3.000000	-0.145308	0.296732	0.644044
Bacteroides ovatus	Noradrenaline	0.001000	gr	0.189500	0.191403	0.001903	0.958720	0.056195	3.000000	-0.105852	0.109657	0.016185
Bacteroides ovatus	Noradrenaline	0.000100	gr	0.189500	0.236697	0.047197	0.125457	2.109249	3.000000	-0.024014	0.118407	0.401477
Bacteroides ovatus	Noradrenaline	0.000010	gr	0.189500	0.220556	0.031056	0.171403	1.789921	3.000000	-0.024161	0.086273	0.264177
Bacteroides ovatus	Noradrenaline	0.000001	gr	0.189500	0.197995	0.008495	0.725499	0.385617	3.000000	-0.061614	0.078604	0.072263
Bacteroides ovatus	Adrenaline	0.100000	gr	0.192298	0.162948	-0.029350	0.274563	-1.333546	3.000000	-0.099393	0.040693	-0.249666
Bacteroides ovatus	Adrenaline	0.010000	gr	0.192298	0.164344	-0.027955	0.356004	-1.088495	3.000000	-0.109686	0.053777	-0.237795
Bacteroides ovatus	Adrenaline	0.001000	gr	0.192298	0.220569	0.028271	0.025416	4.150537	3.000000	0.006594	0.049948	0.240487
Bacteroides ovatus	Adrenaline	0.000100	gr	0.192298	0.209411	0.017113	0.584783	0.610307	3.000000	-0.072124	0.106350	0.145573
Bacteroides ovatus	Adrenaline	0.000010	gr	0.192298	0.240757	0.048459	0.069486	2.771426	3.000000	-0.007187	0.104104	0.412213
Bacteroides ovatus	Adrenaline	0.000001	gr	0.192298	0.232249	0.039950	0.206830	1.605036	3.000000	-0.039263	0.119164	0.339836
Bacteroides ovatus	Levodopa	0.100000	dr	-0.011089	-0.013604	-0.002516	0.672327	-0.466997	3.000000	-0.019661	0.014629	-0.107294
Bacteroides ovatus	Levodopa	0.010000	dr	-0.011089	-0.014789	-0.003701	0.249333	-1.425172	3.000000	-0.011964	0.004563	-0.157815
Bacteroides ovatus	Levodopa	0.001000	dr	-0.011089	-0.012428	-0.001340	0.725476	-0.385651	3.000000	-0.012396	0.009716	-0.057136
Bacteroides ovatus	Levodopa	0.000100	dr	-0.011089	-0.010246	0.000843	0.840234	0.219659	3.000000	-0.011367	0.013053	0.035941
Bacteroides ovatus	Levodopa	0.000010	dr	-0.011089	-0.010898	0.000191	0.944929	0.075010	3.000000	-0.007910	0.008291	0.008142
Bacteroides ovatus	Levodopa	0.000001	dr	-0.011089	-0.011039	0.000050	0.968945	0.042262	3.000000	-0.003716	0.003816	0.002133
Bacteroides ovatus	Dopamine	0.100000	dr	-0.007320	-0.018113	-0.010793	0.032197	-3.791200	3.000000	-0.019853	-0.001733	-0.460275
Bacteroides ovatus	Dopamine	0.010000	dr	-0.007320	-0.024224	-0.016905	0.097047	-2.386505	3.000000	-0.039447	0.005638	-0.720912
Bacteroides ovatus	Dopamine	0.001000	dr	-0.007320	-0.030768	-0.023449	0.126313	-2.102094	3.000000	-0.058949	0.012051	-1.000000
Bacteroides ovatus	Dopamine	0.000100	dr	-0.007320	-0.027243	-0.019923	0.077053	-2.649107	3.000000	-0.043858	0.004011	-0.849653
Bacteroides ovatus	Dopamine	0.000010	dr	-0.007320	-0.025675	-0.018355	0.125139	-2.111917	3.000000	-0.046014	0.009304	-0.782763
Bacteroides ovatus	Dopamine	0.000001	dr	-0.007320	-0.012518	-0.005199	0.250952	-1.419001	3.000000	-0.016858	0.006461	-0.221703
Bacteroides ovatus	Noradrenaline	0.100000	dr	-0.009049	-0.018297	-0.009248	0.063627	-2.878107	3.000000	-0.019474	0.000978	-0.394393
Bacteroides ovatus	Noradrenaline	0.010000	dr	-0.009049	-0.015065	-0.006017	0.066113	-2.831412	3.000000	-0.012779	0.000746	-0.256591
Bacteroides ovatus	Noradrenaline	0.001000	dr	-0.009049	-0.013716	-0.004668	0.326630	-1.169610	3.000000	-0.017369	0.008033	-0.199070
Bacteroides ovatus	Noradrenaline	0.000100	dr	-0.009049	-0.013519	-0.004470	0.406846	-0.962394	3.000000	-0.019252	0.010312	-0.190636

Species	Hormon	Concentration	Parameter	Mean_Baseline	Mean_Treatment	Diff	P_Value	T_Statistic	DF	Lower_CI	Upper_CI	Diff_Norm
Bacteroides ovatus	Noradrenaline	0.000010	dr	-0.009049	-0.016287	-0.007239	0.187261	-1.702253	3.000000	-0.020772	0.006294	-0.308701
Bacteroides ovatus	Noradrenaline	0.000001	dr	-0.009049	-0.009559	-0.000510	0.844184	-0.214116	3.000000	-0.008096	0.007075	-0.021764
Bacteroides ovatus	Adrenaline	0.100000	dr	-0.014604	-0.012330	0.002273	0.635756	0.525251	3.000000	-0.011501	0.016048	0.096956
Bacteroides ovatus	Adrenaline	0.010000	dr	-0.014604	-0.012641	0.001963	0.807686	0.265696	3.000000	-0.021551	0.025477	0.083720
Bacteroides ovatus	Adrenaline	0.001000	dr	-0.014604	-0.013376	0.001228	0.770275	0.319541	3.000000	-0.011003	0.013459	0.052374
Bacteroides ovatus	Adrenaline	0.000100	dr	-0.014604	-0.013606	0.000998	0.826802	0.238577	3.000000	-0.012319	0.014316	0.042578
Bacteroides ovatus	Adrenaline	0.000010	dr	-0.014604	-0.017069	-0.002465	0.364506	-1.066248	3.000000	-0.009823	0.004893	-0.105133
Bacteroides ovatus	Adrenaline	0.000001	dr	-0.014604	-0.013480	0.001124	0.776963	0.309833	3.000000	-0.010425	0.012674	0.047951
Bacteroides ovatus	Levodopa	0.100000	td	3.438873	3.927656	0.488783	0.690965	0.438062	3.000000	-3.062139	4.039704	0.443620
Bacteroides ovatus	Levodopa	0.010000	td	3.438873	3.777631	0.338758	0.731083	0.377266	3.000000	-2.518853	3.196368	0.307457
Bacteroides ovatus	Levodopa	0.001000	td	3.438873	4.300411	0.861538	0.468005	0.828789	3.000000	-2.446660	4.169735	0.781933
Bacteroides ovatus	Levodopa	0.000100	td	3.438873	3.854274	0.415401	0.751768	0.346614	3.000000	-3.398610	4.229411	0.377018
Bacteroides ovatus	Levodopa	0.000010	td	3.438873	3.845664	0.406790	0.739435	0.364837	3.000000	-3.141610	3.955190	0.369203
Bacteroides ovatus	Levodopa	0.000001	td	3.438873	3.975445	0.536572	0.659057	0.487897	3.000000	-2.963369	4.036512	0.486993
Bacteroides ovatus	Dopamine	0.100000	td	4.102777	3.455501	-0.647276	0.558115	-0.656946	3.000000	-3.782879	2.488327	-0.587469
Bacteroides ovatus	Dopamine	0.010000	td	4.102777	3.436749	-0.666028	0.471650	-0.821326	3.000000	-3.246732	1.914675	-0.604488
Bacteroides ovatus	Dopamine	0.001000	td	4.102777	3.741219	-0.361558	0.711372	-0.406899	3.000000	-3.189388	2.466271	-0.328151
Bacteroides ovatus	Dopamine	0.000100	td	4.102777	3.098581	-1.004196	0.292566	-1.273467	3.000000	-3.513725	1.505332	-0.911410
Bacteroides ovatus	Dopamine	0.000010	td	4.102777	3.000972	-1.101806	0.191522	-1.680141	3.000000	-3.188795	0.985184	-1.000000
Bacteroides ovatus	Dopamine	0.000001	td	4.102777	3.794297	-0.308480	0.654958	-0.494406	3.000000	-2.294136	1.677176	-0.279977
Bacteroides ovatus	Noradrenaline	0.100000	td	3.832543	2.896177	-0.936366	0.438847	-0.890398	3.000000	-4.283112	2.410380	-0.849847
Bacteroides ovatus	Noradrenaline	0.010000	td	3.832543	2.903869	-0.928673	0.374826	-1.039911	3.000000	-3.770696	1.913350	-0.842865
Bacteroides ovatus	Noradrenaline	0.001000	td	3.832543	4.165257	0.332714	0.692737	0.435336	3.000000	-2.099533	2.764960	0.301972
Bacteroides ovatus	Noradrenaline	0.000100	td	3.832543	3.227666	-0.604877	0.014398	-5.123034	3.000000	-0.980629	-0.229125	-0.548987
Bacteroides ovatus	Noradrenaline	0.000010	td	3.832543	3.415082	-0.417461	0.041614	-3.427498	3.000000	-0.805075	-0.029847	-0.378888
Bacteroides ovatus	Noradrenaline	0.000001	td	3.832543	3.777702	-0.054841	0.894396	-0.144322	3.000000	-1.264131	1.154450	-0.049773
Bacteroides ovatus	Adrenaline	0.100000	td	3.931318	4.700397	0.769079	0.308106	1.224630	3.000000	-1.229526	2.767683	0.698017

Species	Hormon	Concentration	Parameter	Mean_Baseline	Mean_Treatment	Diff	P_Value	T_Statistic	DF	Lower_CI	Upper_CI	Diff_Norm
Bacteroides ovatus	Adrenaline	0.010000	td	3.931318	4.535995	0.604676	0.389917	1.002628	3.000000	-1.314630	2.523983	0.548805
Bacteroides ovatus	Adrenaline	0.001000	td	3.931318	3.472877	-0.458441	0.001953	-10.296880	3.000000	-0.600131	-0.316751	-0.416082
Bacteroides ovatus	Adrenaline	0.000100	td	3.931318	3.364838	-0.566480	0.362000	-1.072751	3.000000	-2.247013	1.114053	-0.514138
Bacteroides ovatus	Adrenaline	0.000010	td	3.931318	3.038932	-0.892387	0.164071	-1.833657	3.000000	-2.441190	0.656416	-0.809931
Bacteroides ovatus	Adrenaline	0.000001	td	3.931318	3.187593	-0.743725	0.301168	-1.246111	3.000000	-2.643127	1.155677	-0.675006
Bacteroides ovatus	Levodopa	0.100000	lagC	9.429319	16.685714	7.256395	0.057234	3.009513	3.000000	-0.416968	14.929758	0.855837
Bacteroides ovatus	Levodopa	0.010000	lagC	9.429319	15.919774	6.490455	0.064399	2.863366	3.000000	-0.723266	13.704177	0.765500
Bacteroides ovatus	Levodopa	0.001000	lagC	9.429319	14.385635	4.956316	0.051753	3.137764	3.000000	-0.070579	9.983210	0.584560
Bacteroides ovatus	Levodopa	0.000100	lagC	9.429319	14.176492	4.747173	0.152371	1.908366	3.000000	-3.169351	12.663698	0.559893
Bacteroides ovatus	Levodopa	0.000010	lagC	9.429319	13.659077	4.229758	0.159562	1.861691	3.000000	-3.000754	11.460270	0.498868
Bacteroides ovatus	Levodopa	0.000001	lagC	9.429319	13.837114	4.407795	0.124452	2.117716	3.000000	-2.216121	11.031710	0.519866
Bacteroides ovatus	Dopamine	0.100000	lagC	11.450010	19.928727	8.478716	0.028769	3.959204	3.000000	1.663443	15.293989	1.000000
Bacteroides ovatus	Dopamine	0.010000	lagC	11.450010	16.681199	5.231188	0.103132	2.319489	3.000000	-1.946243	12.408620	0.616979
Bacteroides ovatus	Dopamine	0.001000	lagC	11.450010	14.929047	3.479037	0.125602	2.108034	3.000000	-1.773179	8.731252	0.410326
Bacteroides ovatus	Dopamine	0.000100	lagC	11.450010	16.307572	4.857561	0.180457	1.738789	3.000000	-4.033069	13.748192	0.572912
Bacteroides ovatus	Dopamine	0.000010	lagC	11.450010	16.175250	4.725239	0.153614	1.900114	3.000000	-3.188929	12.639407	0.557306
Bacteroides ovatus	Dopamine	0.000001	lagC	11.450010	15.683185	4.233175	0.166200	1.820729	3.000000	-3.165977	11.632327	0.499271
Bacteroides ovatus	Noradrenaline	0.100000	lagC	11.397004	15.638864	4.241861	0.247686	1.431499	3.000000	-5.188462	13.672183	0.500295
Bacteroides ovatus	Noradrenaline	0.010000	lagC	11.397004	13.151773	1.754770	0.537231	0.694665	3.000000	-6.284305	9.793845	0.206962
Bacteroides ovatus	Noradrenaline	0.001000	lagC	11.397004	12.959821	1.562817	0.205862	1.609600	3.000000	-1.527132	4.652767	0.184322
Bacteroides ovatus	Noradrenaline	0.000100	lagC	11.397004	15.215929	3.818925	0.195796	1.658509	3.000000	-3.509058	11.146909	0.450413
Bacteroides ovatus	Noradrenaline	0.000010	lagC	11.397004	14.766379	3.369376	0.232475	1.492157	3.000000	-3.816769	10.555521	0.397392
Bacteroides ovatus	Noradrenaline	0.000001	lagC	11.397004	14.081665	2.684661	0.216254	1.561838	3.000000	-2.785682	8.155004	0.316635
Bacteroides ovatus	Adrenaline	0.100000	lagC	11.773904	12.539712	0.765807	0.697041	0.428730	3.000000	-4.918755	6.450370	0.090321
Bacteroides ovatus	Adrenaline	0.010000	lagC	11.773904	12.277908	0.504004	0.822041	0.245309	3.000000	-6.034553	7.042561	0.059443
Bacteroides ovatus	Adrenaline	0.001000	lagC	11.773904	15.009686	3.235782	0.357871	1.083564	3.000000	-6.267765	12.739329	0.381636
Bacteroides ovatus	Adrenaline	0.000100	lagC	11.773904	15.875679	4.101775	0.286840	1.292147	3.000000	-6.000546	14.204096	0.483773

Species	Hormon	Concentration	Parameter	Mean_Baseline	Mean_Treatment	Diff	P_Value	T_Statistic	DF	Lower_CI	Upper_CI	Diff_Norm
Bacteroides ovatus	Adrenaline	0.000010	lagC	11.773904	16.137457	4.363553	0.181053	1.735522	3.000000	-3.637943	12.365050	0.514648
Bacteroides ovatus	Adrenaline	0.000001	lagC	11.773904	15.942990	4.169085	0.164269	1.832444	3.000000	-3.071457	11.409628	0.491712
Bacteroides ovatus	Levodopa	0.100000	t_k	25.583333	26.166667	0.583333	0.854270	0.200000	3.000000	-8.698802	9.865468	0.046358
Bacteroides ovatus	Levodopa	0.010000	t_k	25.583333	25.833333	0.250000	0.934015	0.089924	3.000000	-8.597640	9.097640	0.019868
Bacteroides ovatus	Levodopa	0.001000	t_k	25.583333	25.166667	-0.416667	0.901348	-0.134742	3.000000	-10.257838	9.424505	-0.033113
Bacteroides ovatus	Levodopa	0.000100	t_k	25.583333	25.916667	0.333333	0.919122	0.110319	3.000000	-9.282516	9.949183	0.026490
Bacteroides ovatus	Levodopa	0.000010	t_k	25.583333	25.500000	-0.083333	0.978697	-0.028984	3.000000	-9.233187	9.066520	-0.006623
Bacteroides ovatus	Levodopa	0.000001	t_k	25.583333	26.500000	0.916667	0.728089	0.381739	3.000000	-6.725316	8.558650	0.072848
Bacteroides ovatus	Dopamine	0.100000	t_k	37.750000	28.166667	-9.583333	0.171245	-1.790837	3.000000	-26.613607	7.446940	-0.761589
Bacteroides ovatus	Dopamine	0.010000	t_k	37.750000	27.750000	-10.000000	0.175170	-1.768279	3.000000	-27.997425	7.997425	-0.794702
Bacteroides ovatus	Dopamine	0.001000	t_k	37.750000	25.916667	-11.833333	0.173051	-1.780386	3.000000	-32.985454	9.318787	-0.940397
Bacteroides ovatus	Dopamine	0.000100	t_k	37.750000	25.166667	-12.583333	0.126207	-2.102977	3.000000	-31.625758	6.459092	-1.000000
Bacteroides ovatus	Dopamine	0.000010	t_k	37.750000	25.250000	-12.500000	0.131248	-2.061965	3.000000	-31.792555	6.792555	-0.993377
Bacteroides ovatus	Dopamine	0.000001	t_k	37.750000	31.500000	-6.250000	0.327866	-1.166054	3.000000	-23.307784	10.807784	-0.496689
Bacteroides ovatus	Noradrenaline	0.100000	t_k	32.416667	25.416667	-7.000000	0.326920	-1.168773	3.000000	-26.060269	12.060269	-0.556291
Bacteroides ovatus	Noradrenaline	0.010000	t_k	32.416667	24.583333	-7.833333	0.328907	-1.163067	3.000000	-29.267319	13.600652	-0.622517
Bacteroides ovatus	Noradrenaline	0.001000	t_k	32.416667	24.833333	-7.583333	0.309237	-1.221174	3.000000	-27.345915	12.179248	-0.602649
Bacteroides ovatus	Noradrenaline	0.000100	t_k	32.416667	25.583333	-6.833333	0.365673	-1.063233	3.000000	-27.286724	13.620058	-0.543046
Bacteroides ovatus	Noradrenaline	0.000010	t_k	32.416667	24.916667	-7.500000	0.299572	-1.251127	3.000000	-26.577481	11.577481	-0.596026
Bacteroides ovatus	Noradrenaline	0.000001	t_k	32.416667	26.583333	-5.833333	0.467507	-0.829813	3.000000	-28.204973	16.538306	-0.463576
Bacteroides ovatus	Adrenaline	0.100000	t_k	26.000000	25.250000	-0.750000	0.795087	-0.283708	3.000000	-9.162998	7.662998	-0.059603
Bacteroides ovatus	Adrenaline	0.010000	t_k	26.000000	25.083333	-0.916667	0.743824	-0.358336	3.000000	-9.057748	7.224415	-0.072848
Bacteroides ovatus	Adrenaline	0.001000	t_k	26.000000	24.916667	-1.083333	0.759410	-0.335398	3.000000	-11.362620	9.195954	-0.086093
Bacteroides ovatus	Adrenaline	0.000100	t_k	26.000000	25.666667	-0.333333	0.912208	-0.119808	3.000000	-9.187595	8.520928	-0.026490
Bacteroides ovatus	Adrenaline	0.000010	t_k	26.000000	25.416667	-0.583333	0.827980	-0.236914	3.000000	-8.419199	7.252532	-0.046358
Bacteroides ovatus	Adrenaline	0.000001	t_k	26.000000	26.333333	0.333333	0.906600	0.127515	3.000000	-7.985787	8.652453	0.026490
Bacteroides ovatus	Levodopa	0.100000	t_gr	12.833333	20.750000	7.916667	0.041304	3.437729	3.000000	0.587885	15.245448	0.950000

Species	Hormon	Concentration	Parameter	Mean_Baseline	Mean_Treatment	Diff	P_Value	T_Statistic	DF	Lower_CI	Upper_CI	Diff_Norm
Bacteroides ovatus	Levodopa	0.010000	t_gr	12.833333	20.416667	7.583333	0.052345	3.123112	3.000000	-0.144072	15.310738	0.910000
Bacteroides ovatus	Levodopa	0.001000	t_gr	12.833333	20.166667	7.333333	0.062651	2.897078	3.000000	-0.722348	15.389014	0.880000
Bacteroides ovatus	Levodopa	0.000100	t_gr	12.833333	19.166667	6.333333	0.082229	2.573685	3.000000	-1.498043	14.164709	0.760000
Bacteroides ovatus	Levodopa	0.000010	t_gr	12.833333	19.000000	6.166667	0.067424	2.807653	3.000000	-0.823187	13.156520	0.740000
Bacteroides ovatus	Levodopa	0.000001	t_gr	12.833333	19.000000	6.166667	0.077615	2.640613	3.000000	-1.265353	13.598686	0.740000
Bacteroides ovatus	Dopamine	0.100000	t_gr	16.083333	24.416667	8.333333	0.021358	4.430971	3.000000	2.348103	14.318564	1.000000
Bacteroides ovatus	Dopamine	0.010000	t_gr	16.083333	21.166667	5.083333	0.124144	2.120323	3.000000	-2.546369	12.713035	0.610000
Bacteroides ovatus	Dopamine	0.001000	t_gr	16.083333	20.250000	4.166667	0.198544	1.644879	3.000000	-3.894833	12.228166	0.500000
Bacteroides ovatus	Dopamine	0.000100	t_gr	16.083333	21.000000	4.916667	0.168723	1.805652	3.000000	-3.748914	13.582248	0.590000
Bacteroides ovatus	Dopamine	0.000010	t_gr	16.083333	20.750000	4.666667	0.188518	1.695673	3.000000	-4.091755	13.425089	0.560000
Bacteroides ovatus	Dopamine	0.000001	t_gr	16.083333	20.416667	4.333333	0.207432	1.602214	3.000000	-4.273884	12.940550	0.520000
Bacteroides ovatus	Noradrenaline	0.100000	t_gr	15.750000	19.083333	3.333333	0.466731	0.831411	3.000000	-9.425885	16.092552	0.400000
Bacteroides ovatus	Noradrenaline	0.010000	t_gr	15.750000	17.000000	1.250000	0.757762	0.337812	3.000000	-10.525967	13.025967	0.150000
Bacteroides ovatus	Noradrenaline	0.001000	t_gr	15.750000	16.916667	1.166667	0.504441	0.756297	3.000000	-3.742590	6.075924	0.140000
Bacteroides ovatus	Noradrenaline	0.000100	t_gr	15.750000	20.083333	4.333333	0.184036	1.719380	3.000000	-3.687348	12.354015	0.520000
Bacteroides ovatus	Noradrenaline	0.000010	t_gr	15.750000	19.250000	3.500000	0.256565	1.397931	3.000000	-4.467894	11.467894	0.420000
Bacteroides ovatus	Noradrenaline	0.000001	t_gr	15.750000	19.000000	3.250000	0.282651	1.306061	3.000000	-4.669196	11.169196	0.390000
Bacteroides ovatus	Adrenaline	0.100000	t_gr	17.083333	19.083333	2.000000	0.542796	0.684505	3.000000	-7.298538	11.298538	0.240000
Bacteroides ovatus	Adrenaline	0.010000	t_gr	17.083333	17.916667	0.833333	0.834429	0.227823	3.000000	-10.807475	12.474141	0.100000
Bacteroides ovatus	Adrenaline	0.001000	t_gr	17.083333	20.166667	3.083333	0.406509	0.963180	3.000000	-7.104316	13.270982	0.370000
Bacteroides ovatus	Adrenaline	0.000100	t_gr	17.083333	20.833333	3.750000	0.311258	1.215033	3.000000	-6.072095	13.572095	0.450000
Bacteroides ovatus	Adrenaline	0.000010	t_gr	17.083333	20.583333	3.500000	0.293820	1.269429	3.000000	-5.274468	12.274468	0.420000
Bacteroides ovatus	Adrenaline	0.000001	t_gr	17.083333	20.416667	3.333333	0.306059	1.230915	3.000000	-5.284772	11.951439	0.400000
Bacteroides ovatus	Levodopa	0.100000	t_dr	42.083333	41.166667	-0.916667	0.901272	-0.134847	3.000000	-22.550432	20.717099	-0.063953
Bacteroides ovatus	Levodopa	0.010000	t_dr	42.083333	37.666667	-4.416667	0.657630	-0.490160	3.000000	-33.092632	24.259299	-0.308140
Bacteroides ovatus	Levodopa	0.001000	t_dr	42.083333	30.833333	-11.250000	0.127921	-2.088822	3.000000	-28.390050	5.890050	-0.784884
Bacteroides ovatus	Levodopa	0.000100	t_dr	42.083333	36.916667	-5.166667	0.340223	-1.131206	3.000000	-19.702161	9.368828	-0.360465

Species	Hormon	Concentration	Parameter	Mean_Baseline	Mean_Treatment	Diff	P_Value	T_Statistic	DF	Lower_CI	Upper_CI	Diff_Norm
Bacteroides ovatus	Levodopa	0.000010	t_dr	42.083333	44.750000	2.666667	0.475065	0.814379	3.000000	-7.754192	13.087525	0.186047
Bacteroides ovatus	Levodopa	0.000001	t_dr	42.083333	42.166667	0.083333	0.984999	0.020408	3.000000	-12.911656	13.078322	0.005814
Bacteroides ovatus	Dopamine	0.100000	t_dr	44.833333	44.166667	-0.666667	0.914706	-0.116379	3.000000	-18.897061	17.563728	-0.046512
Bacteroides ovatus	Dopamine	0.010000	t_dr	44.833333	36.500000	-8.333333	0.177202	-1.756821	3.000000	-23.429002	6.762335	-0.581395
Bacteroides ovatus	Dopamine	0.001000	t_dr	44.833333	30.500000	-14.333333	0.089119	-2.481917	3.000000	-32.712299	4.045632	-1.000000
Bacteroides ovatus	Dopamine	0.000100	t_dr	44.833333	32.833333	-12.000000	0.244650	-1.443275	3.000000	-38.460205	14.460205	-0.837209
Bacteroides ovatus	Dopamine	0.000010	t_dr	44.833333	38.916667	-5.916667	0.391002	-1.000000	3.000000	-24.746141	12.912807	-0.412791
Bacteroides ovatus	Dopamine	0.000001	t_dr	44.833333	39.583333	-5.250000	0.392249	-0.996990	3.000000	-22.008282	11.508282	-0.366279
Bacteroides ovatus	Noradrenaline	0.100000	t_dr	43.083333	36.583333	-6.500000	0.189952	-1.688222	3.000000	-18.753069	5.753069	-0.453488
Bacteroides ovatus	Noradrenaline	0.010000	t_dr	43.083333	36.833333	-6.250000	0.295681	-1.263467	3.000000	-21.992629	9.492629	-0.436047
Bacteroides ovatus	Noradrenaline	0.001000	t_dr	43.083333	35.833333	-7.250000	0.285381	-1.296967	3.000000	-25.039757	10.539757	-0.505814
Bacteroides ovatus	Noradrenaline	0.000100	t_dr	43.083333	34.833333	-8.250000	0.230901	-1.498682	3.000000	-25.768851	9.268851	-0.575581
Bacteroides ovatus	Noradrenaline	0.000010	t_dr	43.083333	36.000000	-7.083333	0.278319	-1.320676	3.000000	-24.152109	9.985442	-0.494186
Bacteroides ovatus	Noradrenaline	0.000001	t_dr	43.083333	44.000000	0.916667	0.764633	0.327762	3.000000	-7.983811	9.817144	0.063953
Bacteroides ovatus	Adrenaline	0.100000	t_dr	42.000000	34.333333	-7.666667	0.279728	-1.315898	3.000000	-26.208191	10.874858	-0.534884
Bacteroides ovatus	Adrenaline	0.010000	t_dr	42.000000	40.416667	-1.583333	0.513859	-0.738270	3.000000	-8.408577	5.241910	-0.110465
Bacteroides ovatus	Adrenaline	0.001000	t_dr	42.000000	31.166667	-10.833333	0.172597	-1.782998	3.000000	-30.169587	8.502920	-0.755814
Bacteroides ovatus	Adrenaline	0.000100	t_dr	42.000000	35.333333	-6.666667	0.299023	-1.252858	3.000000	-23.600994	10.267661	-0.465116
Bacteroides ovatus	Adrenaline	0.000010	t_dr	42.000000	35.000000	-7.000000	0.305101	-1.233872	3.000000	-25.054651	11.054651	-0.488372
Bacteroides ovatus	Adrenaline	0.000001	t_dr	42.000000	36.333333	-5.666667	0.493996	-0.776615	3.000000	-28.887785	17.554452	-0.395349
Bacteroides thetaiotaomicron	Levodopa	0.100000	auc_lin	11.675362	3.932779	-7.742583	0.015748	-4.958506	3.000000	-12.711893	-2.773272	-1.000000
Bacteroides thetaiotaomicron	Levodopa	0.010000	auc_lin	11.675362	5.446403	-6.228959	0.046332	-3.282698	3.000000	-12.267688	-0.190229	-0.804507
Bacteroides thetaiotaomicron	Levodopa	0.001000	auc_lin	11.675362	6.351018	-5.324343	0.077301	-2.645342	3.000000	-11.729729	1.081043	-0.687670
Bacteroides thetaiotaomicron	Levodopa	0.000100	auc_lin	11.675362	7.281294	-4.394067	0.133829	-2.041693	3.000000	-11.243230	2.455095	-0.567520
Bacteroides thetaiotaomicron	Levodopa	0.000010	auc_lin	11.675362	7.497327	-4.178035	0.146182	-1.950640	3.000000	-10.994451	2.638382	-0.539618
Bacteroides thetaiotaomicron	Levodopa	0.000001	auc_lin	11.675362	7.435556	-4.239806	0.133131	-2.047129	3.000000	-10.830967	2.351356	-0.547596
Bacteroides thetaiotaomicron	Dopamine	0.100000	auc_lin	10.236652	7.046616	-3.190036	0.534734	-0.699250	3.000000	-17.708613	11.328541	-0.412012

Species	Hormon	Concentration	Parameter	Mean_Baseline	Mean_Treatment	Diff	P_Value	T_Statistic	DF	Lower_CI	Upper_CI	Diff_Norm
Bacteroides thetaiotaomicron	Dopamine	0.010000	auc_lin	10.236652	6.558580	-3.678071	0.193890	-1.668089	3.000000	-10.695240	3.339097	-0.475044
Bacteroides thetaiotaomicron	Dopamine	0.001000	auc_lin	10.236652	7.286613	-2.950038	0.303892	-1.237616	3.000000	-10.535866	4.635790	-0.381015
Bacteroides thetaiotaomicron	Dopamine	0.000100	auc_lin	10.236652	8.203778	-2.032873	0.463408	-0.838275	3.000000	-9.750523	5.684776	-0.262558
Bacteroides thetaiotaomicron	Dopamine	0.000010	auc_lin	10.236652	10.193247	-0.043405	0.991867	-0.011064	3.000000	-12.528727	12.441917	-0.005606
Bacteroides thetaiotaomicron	Dopamine	0.000001	auc_lin	10.236652	7.598380	-2.638271	0.330371	-1.158884	3.000000	-9.883307	4.606764	-0.340748
Bacteroides thetaiotaomicron	Noradrenaline	0.100000	auc_lin	9.835960	4.448680	-5.387279	0.059808	-2.954502	3.000000	-11.190195	0.415636	-0.695799
Bacteroides thetaiotaomicron	Noradrenaline	0.010000	auc_lin	9.835960	6.929795	-2.906164	0.280055	-1.314792	3.000000	-9.940520	4.128192	-0.375348
Bacteroides thetaiotaomicron	Noradrenaline	0.001000	auc_lin	9.835960	7.500698	-2.335261	0.380926	-1.024671	3.000000	-9.588168	4.917646	-0.301613
Bacteroides thetaiotaomicron	Noradrenaline	0.000100	auc_lin	9.835960	7.415805	-2.420154	0.385920	-1.012368	3.000000	-10.028072	5.187764	-0.312577
Bacteroides thetaiotaomicron	Noradrenaline	0.000010	auc_lin	9.835960	7.105178	-2.730782	0.294793	-1.266307	3.000000	-9.593703	4.132139	-0.352697
Bacteroides thetaiotaomicron	Noradrenaline	0.000001	auc_lin	9.835960	7.310800	-2.525159	0.311495	-1.214316	3.000000	-9.143030	4.092712	-0.326139
Bacteroides thetaiotaomicron	Adrenaline	0.100000	auc_lin	10.446959	10.284434	-0.162524	0.967225	-0.044605	3.000000	-11.758083	11.433034	-0.020991
Bacteroides thetaiotaomicron	Adrenaline	0.010000	auc_lin	10.446959	7.441702	-3.005256	0.285853	-1.295404	3.000000	-10.388332	4.377820	-0.388147
Bacteroides thetaiotaomicron	Adrenaline	0.001000	auc_lin	10.446959	10.094483	-0.352476	0.935092	-0.088450	3.000000	-13.034558	12.329606	-0.045524
Bacteroides thetaiotaomicron	Adrenaline	0.000100	auc_lin	10.446959	7.419149	-3.027810	0.276952	-1.325341	3.000000	-10.298273	4.242653	-0.391059
Bacteroides thetaiotaomicron	Adrenaline	0.000010	auc_lin	10.446959	7.167274	-3.279685	0.211760	-1.582173	3.000000	-9.876577	3.317207	-0.423591
Bacteroides thetaiotaomicron	Adrenaline	0.000001	auc_lin	10.446959	7.002565	-3.444394	0.188585	-1.695323	3.000000	-9.910181	3.021394	-0.444864
Bacteroides thetaiotaomicron	Levodopa	0.100000	k_lin	0.381642	0.182385	-0.199257	0.009266	-6.001641	3.000000	-0.304916	-0.093599	-1.000000
Bacteroides thetaiotaomicron	Levodopa	0.010000	k_lin	0.381642	0.231167	-0.150475	0.040384	-3.468713	3.000000	-0.288532	-0.012418	-0.755181
Bacteroides thetaiotaomicron	Levodopa	0.001000	k_lin	0.381642	0.250859	-0.130783	0.059650	-2.957790	3.000000	-0.271500	0.009934	-0.656354
Bacteroides thetaiotaomicron	Levodopa	0.000100	k_lin	0.381642	0.276726	-0.104917	0.115279	-2.199006	3.000000	-0.256754	0.046921	-0.526538
Bacteroides thetaiotaomicron	Levodopa	0.000010	k_lin	0.381642	0.281069	-0.100573	0.129313	-2.077481	3.000000	-0.254639	0.053492	-0.504739
Bacteroides thetaiotaomicron	Levodopa	0.000001	k_lin	0.381642	0.277359	-0.104284	0.102637	-2.324757	3.000000	-0.247041	0.038474	-0.523361
Bacteroides thetaiotaomicron	Dopamine	0.100000	k_lin	0.357617	0.264069	-0.093547	0.405462	-0.965624	3.000000	-0.401854	0.214760	-0.469479
Bacteroides thetaiotaomicron	Dopamine	0.010000	k_lin	0.357617	0.260285	-0.097332	0.181215	-1.734639	3.000000	-0.275901	0.081237	-0.488472
Bacteroides thetaiotaomicron	Dopamine	0.001000	k_lin	0.357617	0.281373	-0.076244	0.290054	-1.281616	3.000000	-0.265568	0.113081	-0.382639
Bacteroides thetaiotaomicron	Dopamine	0.000100	k_lin	0.357617	0.293856	-0.063760	0.339531	-1.133125	3.000000	-0.242835	0.115314	-0.319990

Species	Hormon	Concentration	Parameter	Mean_Baseline	Mean_Treatment	Diff	P_Value	T_Statistic	DF	Lower_CI	Upper_CI	Diff_Norm
Bacteroides thetaiotaomicron	Dopamine	0.000010	k_lin	0.357617	0.336695	-0.020922	0.811131	-0.260791	3.000000	-0.276232	0.234389	-0.104999
Bacteroides thetaiotaomicron	Dopamine	0.000001	k_lin	0.357617	0.281561	-0.076056	0.265155	-1.366621	3.000000	-0.253168	0.101055	-0.381697
Bacteroides thetaiotaomicron	Noradrenaline	0.100000	k_lin	0.325828	0.227870	-0.097959	0.094345	-2.417912	3.000000	-0.226892	0.030974	-0.491619
Bacteroides thetaiotaomicron	Noradrenaline	0.010000	k_lin	0.325828	0.263250	-0.062579	0.310277	-1.218008	3.000000	-0.226086	0.100929	-0.314060
Bacteroides thetaiotaomicron	Noradrenaline	0.001000	k_lin	0.325828	0.281853	-0.043976	0.434797	-0.899240	3.000000	-0.199608	0.111656	-0.220698
Bacteroides thetaiotaomicron	Noradrenaline	0.000100	k_lin	0.325828	0.273880	-0.051949	0.450364	-0.865646	3.000000	-0.242933	0.139035	-0.260712
Bacteroides thetaiotaomicron	Noradrenaline	0.000010	k_lin	0.325828	0.262407	-0.063422	0.303548	-1.238685	3.000000	-0.226366	0.099522	-0.318291
Bacteroides thetaiotaomicron	Noradrenaline	0.000001	k_lin	0.325828	0.270674	-0.055155	0.323751	-1.177951	3.000000	-0.204165	0.093856	-0.276801
Bacteroides thetaiotaomicron	Adrenaline	0.100000	k_lin	0.347579	0.339252	-0.008327	0.913004	-0.118715	3.000000	-0.231560	0.214906	-0.041791
Bacteroides thetaiotaomicron	Adrenaline	0.010000	k_lin	0.347579	0.269417	-0.078162	0.241210	-1.456814	3.000000	-0.248909	0.092585	-0.392267
Bacteroides thetaiotaomicron	Adrenaline	0.001000	k_lin	0.347579	0.325366	-0.022213	0.812939	-0.258219	3.000000	-0.295981	0.251555	-0.111479
Bacteroides thetaiotaomicron	Adrenaline	0.000100	k_lin	0.347579	0.277531	-0.070048	0.284935	-1.298447	3.000000	-0.241733	0.101637	-0.351545
Bacteroides thetaiotaomicron	Adrenaline	0.000010	k_lin	0.347579	0.268692	-0.078887	0.208483	-1.597301	3.000000	-0.236062	0.078287	-0.395907
Bacteroides thetaiotaomicron	Adrenaline	0.000001	k_lin	0.347579	0.264165	-0.083414	0.191100	-1.682302	3.000000	-0.241211	0.074382	-0.418626
Bacteroides thetaiotaomicron	Levodopa	0.100000	death_lin	0.010123	0.064497	0.054374	0.012167	5.444665	3.000000	0.022592	0.086156	1.000000
Bacteroides thetaiotaomicron	Levodopa	0.010000	death_lin	0.010123	0.010884	0.000762	0.965575	0.046852	3.000000	-0.050967	0.052490	0.014006
Bacteroides thetaiotaomicron	Levodopa	0.001000	death_lin	0.010123	0.013251	0.003128	0.863969	0.186475	3.000000	-0.050250	0.056506	0.057522
Bacteroides thetaiotaomicron	Levodopa	0.000100	death_lin	0.010123	0.012621	0.002498	0.889790	0.150680	3.000000	-0.050270	0.055267	0.045949
Bacteroides thetaiotaomicron	Levodopa	0.000010	death_lin	0.010123	0.011321	0.001198	0.948324	0.070375	3.000000	-0.052969	0.055364	0.022029
Bacteroides thetaiotaomicron	Levodopa	0.000001	death_lin	0.010123	0.007240	-0.002883	0.825653	-0.240201	3.000000	-0.041085	0.035318	-0.053028
Bacteroides thetaiotaomicron	Dopamine	0.100000	death_lin	0.001366	0.023993	0.022627	0.209716	1.591578	3.000000	-0.022617	0.067871	0.416140
Bacteroides thetaiotaomicron	Dopamine	0.010000	death_lin	0.001366	0.005446	0.004080	0.217265	1.557326	3.000000	-0.004258	0.012418	0.075037
Bacteroides thetaiotaomicron	Dopamine	0.001000	death_lin	0.001366	0.017985	0.016619	0.110831	2.241274	3.000000	-0.006979	0.040216	0.305639
Bacteroides thetaiotaomicron	Dopamine	0.000100	death_lin	0.001366	0.009292	0.007925	0.059061	2.970149	3.000000	-0.000566	0.016417	0.145756
Bacteroides thetaiotaomicron	Dopamine	0.000010	death_lin	0.001366	0.009231	0.007865	0.417045	0.938903	3.000000	-0.018793	0.034522	0.144638
Bacteroides thetaiotaomicron	Dopamine	0.000001	death_lin	0.001366	0.005274	0.003908	0.539930	0.689727	3.000000	-0.014123	0.021938	0.071867
Bacteroides thetaiotaomicron	Noradrenaline	0.100000	death_lin	0.002528	0.002484	-0.000044	0.987749	-0.016667	3.000000	-0.008443	0.008355	-0.000809

Species	Hormon	Concentration	Parameter	Mean_Baseline	Mean_Treatment	Diff	P_Value	T_Statistic	DF	Lower_CI	Upper_CI	Diff_Norm
Bacteroides thetaiotaomicron	Noradrenaline	0.010000	death_lin	0.002528	0.008952	0.006424	0.418024	0.936675	3.000000	-0.015401	0.028249	0.118139
Bacteroides thetaiotaomicron	Noradrenaline	0.001000	death_lin	0.002528	0.006251	0.003723	0.602110	0.580833	3.000000	-0.016676	0.024122	0.068471
Bacteroides thetaiotaomicron	Noradrenaline	0.000100	death_lin	0.002528	0.006398	0.003870	0.461280	0.842693	3.000000	-0.010745	0.018485	0.071174
Bacteroides thetaiotaomicron	Noradrenaline	0.000010	death_lin	0.002528	0.010953	0.008425	0.140499	1.991358	3.000000	-0.005039	0.021889	0.154942
Bacteroides thetaiotaomicron	Noradrenaline	0.000001	death_lin	0.002528	0.011117	0.008589	0.346341	1.114419	3.000000	-0.015939	0.033118	0.157967
Bacteroides thetaiotaomicron	Adrenaline	0.100000	death_lin	0.000086	0.011683	0.011598	0.370028	1.052066	3.000000	-0.023485	0.046680	0.213297
Bacteroides thetaiotaomicron	Adrenaline	0.010000	death_lin	0.000086	0.008879	0.008793	0.174700	1.770945	3.000000	-0.007008	0.024595	0.161716
Bacteroides thetaiotaomicron	Adrenaline	0.001000	death_lin	0.000086	0.016560	0.016474	0.187264	1.702241	3.000000	-0.014325	0.047274	0.302983
Bacteroides thetaiotaomicron	Adrenaline	0.000100	death_lin	0.000086	0.013230	0.013144	0.212525	1.578675	3.000000	-0.013353	0.039642	0.241739
Bacteroides thetaiotaomicron	Adrenaline	0.000010	death_lin	0.000086	0.017548	0.017462	0.136662	2.019957	3.000000	-0.010049	0.044974	0.321149
Bacteroides thetaiotaomicron	Adrenaline	0.000001	death_lin	0.000086	0.020602	0.020517	0.176760	1.759301	3.000000	-0.016596	0.057630	0.377325
Bacteroides thetaiotaomicron	Levodopa	0.100000	gr	0.227062	0.070570	-0.156493	0.100942	-2.343038	3.000000	-0.369050	0.056065	-1.000000
Bacteroides thetaiotaomicron	Levodopa	0.010000	gr	0.227062	0.080812	-0.146250	0.114164	-2.209410	3.000000	-0.356909	0.064409	-0.934548
Bacteroides thetaiotaomicron	Levodopa	0.001000	gr	0.227062	0.092372	-0.134690	0.131038	-2.063642	3.000000	-0.342402	0.073022	-0.860679
Bacteroides thetaiotaomicron	Levodopa	0.000100	gr	0.227062	0.117479	-0.109583	0.199332	-1.641013	3.000000	-0.322101	0.102934	-0.700246
Bacteroides thetaiotaomicron	Levodopa	0.000010	gr	0.227062	0.119663	-0.107399	0.214733	-1.568666	3.000000	-0.325287	0.110488	-0.686290
Bacteroides thetaiotaomicron	Levodopa	0.000001	gr	0.227062	0.121175	-0.105887	0.216650	-1.560066	3.000000	-0.321891	0.110116	-0.676626
Bacteroides thetaiotaomicron	Dopamine	0.100000	gr	0.163265	0.192007	0.028742	0.831452	0.232016	3.000000	-0.365501	0.422985	0.183665
Bacteroides thetaiotaomicron	Dopamine	0.010000	gr	0.163265	0.094048	-0.069217	0.069239	-2.775695	3.000000	-0.148576	0.010143	-0.442300
Bacteroides thetaiotaomicron	Dopamine	0.001000	gr	0.163265	0.103711	-0.059555	0.103714	-2.313333	3.000000	-0.141484	0.022374	-0.380558
Bacteroides thetaiotaomicron	Dopamine	0.000100	gr	0.163265	0.119619	-0.043646	0.208884	-1.595438	3.000000	-0.130708	0.043416	-0.278902
Bacteroides thetaiotaomicron	Dopamine	0.000010	gr	0.163265	0.278042	0.114777	0.553026	0.666036	3.000000	-0.433649	0.663203	0.733433
Bacteroides thetaiotaomicron	Dopamine	0.000001	gr	0.163265	0.119187	-0.044078	0.198547	-1.644866	3.000000	-0.129360	0.041203	-0.281664
Bacteroides thetaiotaomicron	Noradrenaline	0.100000	gr	0.157645	0.103323	-0.054322	0.095887	-2.399855	3.000000	-0.126359	0.017714	-0.347122
Bacteroides thetaiotaomicron	Noradrenaline	0.010000	gr	0.157645	0.109643	-0.048002	0.162783	-1.841573	3.000000	-0.130954	0.034951	-0.306735
Bacteroides thetaiotaomicron	Noradrenaline	0.001000	gr	0.157645	0.115382	-0.042263	0.200047	-1.637513	3.000000	-0.124401	0.039874	-0.270067
Bacteroides thetaiotaomicron	Noradrenaline	0.000100	gr	0.157645	0.118798	-0.038847	0.262594	-1.375839	3.000000	-0.128705	0.051010	-0.248238

Species	Hormon	Concentration	Parameter	Mean_Baseline	Mean_Treatment	Diff	P_Value	T_Statistic	DF	Lower_CI	Upper_CI	Diff_Norm
Bacteroides thetaiotaomicron	Noradrenaline	0.000010	gr	0.157645	0.119444	-0.038201	0.258528	-1.390677	3.000000	-0.125621	0.049219	-0.244109
Bacteroides thetaiotaomicron	Noradrenaline	0.000001	gr	0.157645	0.115125	-0.042520	0.182532	-1.727480	3.000000	-0.120853	0.035813	-0.271707
Bacteroides thetaiotaomicron	Adrenaline	0.100000	gr	0.152049	0.250127	0.098078	0.564468	0.645688	3.000000	-0.385326	0.581482	0.626726
Bacteroides thetaiotaomicron	Adrenaline	0.010000	gr	0.152049	0.112470	-0.039579	0.255629	-1.401408	3.000000	-0.129459	0.050301	-0.252913
Bacteroides thetaiotaomicron	Adrenaline	0.001000	gr	0.152049	0.257101	0.105052	0.543535	0.683161	3.000000	-0.384325	0.594430	0.671292
Bacteroides thetaiotaomicron	Adrenaline	0.000100	gr	0.152049	0.117001	-0.035048	0.336711	-1.140979	3.000000	-0.132805	0.062709	-0.223959
Bacteroides thetaiotaomicron	Adrenaline	0.000010	gr	0.152049	0.118554	-0.033495	0.325318	-1.173402	3.000000	-0.124338	0.057348	-0.214034
Bacteroides thetaiotaomicron	Adrenaline	0.000001	gr	0.152049	0.116513	-0.035536	0.294453	-1.267396	3.000000	-0.124768	0.053696	-0.227080
Bacteroides thetaiotaomicron	Levodopa	0.100000	dr	-0.004744	-0.030224	-0.025480	0.005064	-7.420662	3.000000	-0.036407	-0.014552	-0.792778
Bacteroides thetaiotaomicron	Levodopa	0.010000	dr	-0.004744	0.008692	0.013436	0.402720	0.972054	3.000000	-0.030552	0.057424	0.418046
Bacteroides thetaiotaomicron	Levodopa	0.001000	dr	-0.004744	-0.001265	0.003480	0.729565	0.379533	3.000000	-0.025697	0.032656	0.108262
Bacteroides thetaiotaomicron	Levodopa	0.000100	dr	-0.004744	-0.003509	0.001235	0.879811	0.164480	3.000000	-0.022654	0.025124	0.038416
Bacteroides thetaiotaomicron	Levodopa	0.000010	dr	-0.004744	-0.002763	0.001981	0.796079	0.282286	3.000000	-0.020354	0.024316	0.061640
Bacteroides thetaiotaomicron	Levodopa	0.000001	dr	-0.004744	-0.002028	0.002716	0.744638	0.357132	3.000000	-0.021490	0.026922	0.084518
Bacteroides thetaiotaomicron	Dopamine	0.100000	dr	-0.001148	-0.009774	-0.008626	0.348913	-1.107450	3.000000	-0.033415	0.016163	-0.268397
Bacteroides thetaiotaomicron	Dopamine	0.010000	dr	-0.001148	-0.007997	-0.006849	0.498525	-0.767761	3.000000	-0.035241	0.021542	-0.213116
Bacteroides thetaiotaomicron	Dopamine	0.001000	dr	-0.001148	-0.012649	-0.011501	0.194855	-1.663225	3.000000	-0.033507	0.010505	-0.357842
Bacteroides thetaiotaomicron	Dopamine	0.000100	dr	-0.001148	-0.007568	-0.006420	0.375539	-1.038118	3.000000	-0.026101	0.013261	-0.199754
Bacteroides thetaiotaomicron	Dopamine	0.000010	dr	-0.001148	0.000926	0.002073	0.864216	0.186132	3.000000	-0.033377	0.037524	0.064512
Bacteroides thetaiotaomicron	Dopamine	0.000001	dr	-0.001148	0.030992	0.032140	0.275661	1.329766	3.000000	-0.044778	0.109058	1.000000
Bacteroides thetaiotaomicron	Noradrenaline	0.100000	dr	-0.004553	-0.005971	-0.001419	0.832507	-0.230529	3.000000	-0.021003	0.018166	-0.044141
Bacteroides thetaiotaomicron	Noradrenaline	0.010000	dr	-0.004553	-0.007886	-0.003333	0.812424	-0.258952	3.000000	-0.044301	0.037634	-0.103718
Bacteroides thetaiotaomicron	Noradrenaline	0.001000	dr	-0.004553	0.002742	0.007294	0.688142	0.442415	3.000000	-0.045177	0.059765	0.226958
Bacteroides thetaiotaomicron	Noradrenaline	0.000100	dr	-0.004553	-0.009183	-0.004630	0.793435	-0.286079	3.000000	-0.056135	0.046875	-0.144056
Bacteroides thetaiotaomicron	Noradrenaline	0.000010	dr	-0.004553	-0.011530	-0.006978	0.528179	-0.711370	3.000000	-0.038194	0.024239	-0.217107
Bacteroides thetaiotaomicron	Noradrenaline	0.000001	dr	-0.004553	-0.003152	0.001401	0.820943	0.246863	3.000000	-0.016658	0.019460	0.043585
Bacteroides thetaiotaomicron	Adrenaline	0.100000	dr	0.004027	-0.008161	-0.012189	0.300809	-1.247237	3.000000	-0.043289	0.018912	-0.379236

Species	Hormon	Concentration	Parameter	Mean_Baseline	Mean_Treatment	Diff	P_Value	T_Statistic	DF	Lower_CI	Upper_CI	Diff_Norm
Bacteroides thetaiotaomicron	Adrenaline	0.010000	dr	0.004027	-0.012071	-0.016098	0.206154	-1.608221	3.000000	-0.047954	0.015758	-0.500876
Bacteroides thetaiotaomicron	Adrenaline	0.001000	dr	0.004027	-0.010292	-0.014319	0.209184	-1.594041	3.000000	-0.042907	0.014268	-0.445527
Bacteroides thetaiotaomicron	Adrenaline	0.000100	dr	0.004027	-0.006259	-0.010287	0.294124	-1.268453	3.000000	-0.036095	0.015522	-0.320062
Bacteroides thetaiotaomicron	Adrenaline	0.000010	dr	0.004027	-0.015721	-0.019749	0.075932	-2.666254	3.000000	-0.043321	0.003823	-0.614469
Bacteroides thetaiotaomicron	Adrenaline	0.000001	dr	0.004027	-0.015524	-0.019552	0.062863	-2.892918	3.000000	-0.041060	0.001957	-0.608334
Bacteroides thetaiotaomicron	Levodopa	0.100000	td	3.827142	10.215819	6.388676	0.014141	5.156727	3.000000	2.445939	10.331414	1.000000
Bacteroides thetaiotaomicron	Levodopa	0.010000	td	3.827142	9.294725	5.467583	0.046365	3.281733	3.000000	0.165418	10.769748	0.855824
Bacteroides thetaiotaomicron	Levodopa	0.001000	td	3.827142	8.177780	4.350637	0.062985	2.890541	3.000000	-0.439355	9.140629	0.680992
Bacteroides thetaiotaomicron	Levodopa	0.000100	td	3.827142	6.599821	2.772678	0.141736	1.982331	3.000000	-1.678596	7.223952	0.433999
Bacteroides thetaiotaomicron	Levodopa	0.000010	td	3.827142	6.597406	2.770264	0.146169	1.950731	3.000000	-1.749178	7.289706	0.433621
Bacteroides thetaiotaomicron	Levodopa	0.000001	td	3.827142	6.376005	2.548863	0.133950	2.040750	3.000000	-1.425960	6.523686	0.398966
Bacteroides thetaiotaomicron	Dopamine	0.100000	td	4.560821	7.612860	3.052039	0.315185	1.203216	3.000000	-5.020452	11.124530	0.477726
Bacteroides thetaiotaomicron	Dopamine	0.010000	td	4.560821	8.017650	3.456829	0.091688	2.449896	3.000000	-1.033636	7.947294	0.541087
Bacteroides thetaiotaomicron	Dopamine	0.001000	td	4.560821	7.296273	2.735452	0.118313	2.171285	3.000000	-1.273893	6.744797	0.428172
Bacteroides thetaiotaomicron	Dopamine	0.000100	td	4.560821	6.414049	1.853228	0.239387	1.464073	3.000000	-2.175121	5.881578	0.290080
Bacteroides thetaiotaomicron	Dopamine	0.000010	td	4.560821	5.676067	1.115246	0.616840	0.556244	3.000000	-5.265431	7.495923	0.174566
Bacteroides thetaiotaomicron	Dopamine	0.000001	td	4.560821	6.689910	2.129088	0.179007	1.746777	3.000000	-1.749889	6.008066	0.333260
Bacteroides thetaiotaomicron	Noradrenaline	0.100000	td	4.872574	7.116505	2.243931	0.075207	2.677526	3.000000	-0.423154	4.911017	0.351236
Bacteroides thetaiotaomicron	Noradrenaline	0.010000	td	4.872574	6.830616	1.958043	0.187488	1.701065	3.000000	-1.705171	5.621257	0.306486
Bacteroides thetaiotaomicron	Noradrenaline	0.001000	td	4.872574	6.619565	1.746991	0.214653	1.569025	3.000000	-1.796423	5.290405	0.273451
Bacteroides thetaiotaomicron	Noradrenaline	0.000100	td	4.872574	6.419809	1.547236	0.302619	1.241576	3.000000	-2.418686	5.513157	0.242184
Bacteroides thetaiotaomicron	Noradrenaline	0.000010	td	4.872574	6.588401	1.715827	0.239858	1.462192	3.000000	-2.018652	5.450307	0.268573
Bacteroides thetaiotaomicron	Noradrenaline	0.000001	td	4.872574	6.728351	1.855778	0.164675	1.829966	3.000000	-1.371558	5.083113	0.290479
Bacteroides thetaiotaomicron	Adrenaline	0.100000	td	4.883747	5.374923	0.491176	0.807072	0.266571	3.000000	-5.372714	6.355066	0.076882
Bacteroides thetaiotaomicron	Adrenaline	0.010000	td	4.883747	6.742466	1.858718	0.277406	1.323790	3.000000	-2.609717	6.327153	0.290939
Bacteroides thetaiotaomicron	Adrenaline	0.001000	td	4.883747	5.500394	0.616647	0.799999	0.276672	3.000000	-6.476400	7.709694	0.096522
Bacteroides thetaiotaomicron	Adrenaline	0.000100	td	4.883747	6.721461	1.837714	0.327120	1.168197	3.000000	-3.168655	6.844082	0.287652

Species	Hormon	Concentration	Parameter	Mean_Baseline	Mean_Treatment	Diff	P_Value	T_Statistic	DF	Lower_CI	Upper_CI	Diff_Norm
Bacteroides thetaiotaomicron	Adrenaline	0.000010	td	4.883747	6.576549	1.692802	0.276974	1.325264	3.000000	-2.372238	5.757842	0.264969
Bacteroides thetaiotaomicron	Adrenaline	0.000001	td	4.883747	6.765712	1.881965	0.236568	1.475419	3.000000	-2.177392	5.941321	0.294578
Bacteroides thetaiotaomicron	Levodopa	0.100000	lagC	8.216717	15.460702	7.243985	0.030635	3.864772	3.000000	1.278925	13.209045	0.892241
Bacteroides thetaiotaomicron	Levodopa	0.010000	lagC	8.216717	14.420387	6.203670	0.045709	3.300714	3.000000	0.222283	12.185056	0.764106
Bacteroides thetaiotaomicron	Levodopa	0.001000	lagC	8.216717	13.440751	5.224034	0.083509	2.555942	3.000000	-1.280498	11.728566	0.643444
Bacteroides thetaiotaomicron	Levodopa	0.000100	lagC	8.216717	13.047685	4.830968	0.091898	2.447327	3.000000	-1.451109	11.113046	0.595030
Bacteroides thetaiotaomicron	Levodopa	0.000010	lagC	8.216717	12.664651	4.447934	0.085604	2.527604	3.000000	-1.152354	10.048222	0.547852
Bacteroides thetaiotaomicron	Levodopa	0.000001	lagC	8.216717	12.879747	4.663030	0.086970	2.509570	3.000000	-1.250270	10.576331	0.574345
Bacteroides thetaiotaomicron	Dopamine	0.100000	lagC	9.425477	17.008166	7.582689	0.229183	1.505854	3.000000	-8.442434	23.607811	0.933960
Bacteroides thetaiotaomicron	Dopamine	0.010000	lagC	9.425477	14.152610	4.727132	0.086935	2.510036	3.000000	-1.266346	10.720611	0.582241
Bacteroides thetaiotaomicron	Dopamine	0.001000	lagC	9.425477	13.403711	3.978233	0.137753	2.011728	3.000000	-2.315119	10.271586	0.489999
Bacteroides thetaiotaomicron	Dopamine	0.000100	lagC	9.425477	12.224388	2.798911	0.187868	1.699068	3.000000	-2.443600	8.041423	0.344742
Bacteroides thetaiotaomicron	Dopamine	0.000010	lagC	9.425477	11.619145	2.193667	0.427604	0.915131	3.000000	-5.435001	9.822336	0.270194
Bacteroides thetaiotaomicron	Dopamine	0.000001	lagC	9.425477	12.799676	3.374199	0.119121	2.164050	3.000000	-1.587890	8.336288	0.415600
Bacteroides thetaiotaomicron	Noradrenaline	0.100000	lagC	10.208104	18.326966	8.118861	0.060998	2.930052	3.000000	-0.699359	16.937082	1.000000
Bacteroides thetaiotaomicron	Noradrenaline	0.010000	lagC	10.208104	13.414922	3.206818	0.187126	1.702964	3.000000	-2.785985	9.199621	0.394984
Bacteroides thetaiotaomicron	Noradrenaline	0.001000	lagC	10.208104	12.351931	2.143827	0.362559	1.071298	3.000000	-4.224726	8.512380	0.264055
Bacteroides thetaiotaomicron	Noradrenaline	0.000100	lagC	10.208104	12.766518	2.558414	0.278066	1.321540	3.000000	-3.602592	8.719421	0.315120
Bacteroides thetaiotaomicron	Noradrenaline	0.000010	lagC	10.208104	13.148717	2.940613	0.176532	1.760584	3.000000	-2.374863	8.256090	0.362195
Bacteroides thetaiotaomicron	Noradrenaline	0.000001	lagC	10.208104	12.630775	2.422671	0.230057	1.502198	3.000000	-2.709821	7.555163	0.298400
Bacteroides thetaiotaomicron	Adrenaline	0.100000	lagC	9.372969	10.423066	1.050097	0.675070	0.462709	3.000000	-6.172327	8.272522	0.129340
Bacteroides thetaiotaomicron	Adrenaline	0.010000	lagC	9.372969	11.824909	2.451940	0.300549	1.248054	3.000000	-3.800328	8.704207	0.302005
Bacteroides thetaiotaomicron	Adrenaline	0.001000	lagC	9.372969	10.927149	1.554180	0.580076	0.618423	3.000000	-6.443734	9.552094	0.191428
Bacteroides thetaiotaomicron	Adrenaline	0.000100	lagC	9.372969	12.833327	3.460359	0.126783	2.098197	3.000000	-1.788150	8.708867	0.426212
Bacteroides thetaiotaomicron	Adrenaline	0.000010	lagC	9.372969	13.317773	3.944804	0.080946	2.591833	3.000000	-0.898921	8.788529	0.485881
Bacteroides thetaiotaomicron	Adrenaline	0.000001	lagC	9.372969	13.545101	4.172132	0.065297	2.846486	3.000000	-0.492422	8.836687	0.513881
Bacteroides thetaiotaomicron	Levodopa	0.100000	t_k	43.833333	31.416667	-12.416667	0.091563	-2.451423	3.000000	-28.536030	3.702696	-0.876471

Species	Hormon	Concentration	Parameter	Mean_Baseline	Mean_Treatment	Diff	P_Value	T_Statistic	DF	Lower_CI	Upper_CI	Diff_Norm
Bacteroides thetaiotaomicron	Levodopa	0.010000	t_k	43.833333	43.750000	-0.083333	0.988490	-0.015659	3.000000	-17.019737	16.853070	-0.005882
Bacteroides thetaiotaomicron	Levodopa	0.001000	t_k	43.833333	41.416667	-2.416667	0.715280	-0.400990	3.000000	-21.596486	16.763153	-0.170588
Bacteroides thetaiotaomicron	Levodopa	0.000100	t_k	43.833333	39.000000	-4.833333	0.442078	-0.883397	3.000000	-22.245470	12.578803	-0.341176
Bacteroides thetaiotaomicron	Levodopa	0.000010	t_k	43.833333	41.166667	-2.666667	0.560429	-0.652835	3.000000	-15.666165	10.332832	-0.188235
Bacteroides thetaiotaomicron	Levodopa	0.000001	t_k	43.833333	42.250000	-1.583333	0.774431	-0.313504	3.000000	-17.656088	14.489421	-0.111765
Bacteroides thetaiotaomicron	Dopamine	0.100000	t_k	47.500000	41.000000	-6.500000	0.198397	-1.645604	3.000000	-19.070402	6.070402	-0.458824
Bacteroides thetaiotaomicron	Dopamine	0.010000	t_k	47.500000	39.750000	-7.750000	0.176234	-1.762261	3.000000	-21.745630	6.245630	-0.547059
Bacteroides thetaiotaomicron	Dopamine	0.001000	t_k	47.500000	33.333333	-14.166667	0.068261	-2.792771	3.000000	-30.310010	1.976677	-1.000000
Bacteroides thetaiotaomicron	Dopamine	0.000100	t_k	47.500000	39.666667	-7.833333	0.117912	-2.174901	3.000000	-19.295541	3.628874	-0.552941
Bacteroides thetaiotaomicron	Dopamine	0.000010	t_k	47.500000	41.166667	-6.333333	0.288003	-1.288321	3.000000	-21.978113	9.311447	-0.447059
Bacteroides thetaiotaomicron	Dopamine	0.000001	t_k	47.500000	43.750000	-3.750000	0.448340	-0.869954	3.000000	-17.468159	9.968159	-0.264706
Bacteroides thetaiotaomicron	Noradrenaline	0.100000	t_k	42.500000	39.750000	-2.750000	0.582332	-0.614528	3.000000	-16.991390	11.491390	-0.194118
Bacteroides thetaiotaomicron	Noradrenaline	0.010000	t_k	42.500000	37.666667	-4.833333	0.327145	-1.168127	3.000000	-18.001268	8.334601	-0.341176
Bacteroides thetaiotaomicron	Noradrenaline	0.001000	t_k	42.500000	40.833333	-1.666667	0.861763	-0.189547	3.000000	-29.649549	26.316216	-0.117647
Bacteroides thetaiotaomicron	Noradrenaline	0.000100	t_k	42.500000	37.333333	-5.166667	0.615302	-0.558793	3.000000	-34.591948	24.258615	-0.364706
Bacteroides thetaiotaomicron	Noradrenaline	0.000010	t_k	42.500000	36.583333	-5.916667	0.321663	-1.184046	3.000000	-21.819319	9.985985	-0.417647
Bacteroides thetaiotaomicron	Noradrenaline	0.000001	t_k	42.500000	39.666667	-2.833333	0.519183	-0.728200	3.000000	-15.215826	9.549160	-0.200000
Bacteroides thetaiotaomicron	Adrenaline	0.100000	t_k	47.916667	43.583333	-4.333333	0.261998	-1.377997	3.000000	-14.341046	5.674379	-0.305882
Bacteroides thetaiotaomicron	Adrenaline	0.010000	t_k	47.916667	41.166667	-6.750000	0.215691	-1.564360	3.000000	-20.481825	6.981825	-0.476471
Bacteroides thetaiotaomicron	Adrenaline	0.001000	t_k	47.916667	38.750000	-9.166667	0.103922	-2.311142	3.000000	-21.789182	3.455848	-0.647059
Bacteroides thetaiotaomicron	Adrenaline	0.000100	t_k	47.916667	37.166667	-10.750000	0.065217	-2.847976	3.000000	-22.762496	1.262496	-0.758824
Bacteroides thetaiotaomicron	Adrenaline	0.000010	t_k	47.916667	34.000000	-13.916667	0.007772	-6.386961	3.000000	-20.850957	-6.982376	-0.982353
Bacteroides thetaiotaomicron	Adrenaline	0.000001	t_k	47.916667	37.000000	-10.916667	0.078324	-2.630013	3.000000	-24.126375	2.293041	-0.770588
Bacteroides thetaiotaomicron	Levodopa	0.100000	t_gr	12.583333	23.083333	10.500000	0.037284	3.580085	3.000000	1.166231	19.833769	1.000000
Bacteroides thetaiotaomicron	Levodopa	0.010000	t_gr	12.583333	22.083333	9.500000	0.047123	3.260249	3.000000	0.226709	18.773291	0.904762
Bacteroides thetaiotaomicron	Levodopa	0.001000	t_gr	12.583333	21.416667	8.833333	0.065367	2.845189	3.000000	-1.047068	18.713734	0.841270
Bacteroides thetaiotaomicron	Levodopa	0.000100	t_gr	12.583333	19.916667	7.333333	0.091928	2.446963	3.000000	-2.204178	16.870845	0.698413

Species	Hormon	Concentration	Parameter	Mean_Baseline	Mean_Treatment	Diff	P_Value	T_Statistic	DF	Lower_CI	Upper_CI	Diff_Norm
Bacteroides thetaiotaomicron	Levodopa	0.000010	t_gr	12.583333	19.583333	7.000000	0.094663	2.414165	3.000000	-2.227672	16.227672	0.666667
Bacteroides thetaiotaomicron	Levodopa	0.000001	t_gr	12.583333	19.583333	7.000000	0.101169	2.340568	3.000000	-2.517826	16.517826	0.666667
Bacteroides thetaiotaomicron	Dopamine	0.100000	t_gr	14.500000	20.583333	6.083333	0.315479	1.202335	3.000000	-10.018567	22.185234	0.579365
Bacteroides thetaiotaomicron	Dopamine	0.010000	t_gr	14.500000	21.500000	7.000000	0.081662	2.581664	3.000000	-1.628980	15.628980	0.666667
Bacteroides thetaiotaomicron	Dopamine	0.001000	t_gr	14.500000	20.916667	6.416667	0.090048	2.470200	3.000000	-1.850154	14.683487	0.611111
Bacteroides thetaiotaomicron	Dopamine	0.000100	t_gr	14.500000	19.083333	4.583333	0.119695	2.158939	3.000000	-2.172862	11.339529	0.436508
Bacteroides thetaiotaomicron	Dopamine	0.000010	t_gr	14.500000	16.750000	2.250000	0.615277	0.558833	3.000000	-10.563308	15.063308	0.214286
Bacteroides thetaiotaomicron	Dopamine	0.000001	t_gr	14.500000	20.083333	5.583333	0.099446	2.359486	3.000000	-1.947398	13.114065	0.531746
Bacteroides thetaiotaomicron	Noradrenaline	0.100000	t_gr	15.333333	21.583333	6.250000	0.049151	3.204816	3.000000	0.043625	12.456375	0.595238
Bacteroides thetaiotaomicron	Noradrenaline	0.010000	t_gr	15.333333	20.166667	4.833333	0.151590	1.913591	3.000000	-3.204867	12.871534	0.460317
Bacteroides thetaiotaomicron	Noradrenaline	0.001000	t_gr	15.333333	19.333333	4.000000	0.200976	1.632993	3.000000	-3.795370	11.795370	0.380952
Bacteroides thetaiotaomicron	Noradrenaline	0.000100	t_gr	15.333333	19.666667	4.333333	0.178852	1.747632	3.000000	-3.557689	12.224355	0.412698
Bacteroides thetaiotaomicron	Noradrenaline	0.000010	t_gr	15.333333	20.166667	4.833333	0.116016	2.192194	3.000000	-2.183301	11.849968	0.460317
Bacteroides thetaiotaomicron	Noradrenaline	0.000001	t_gr	15.333333	20.000000	4.666667	0.132375	2.053060	3.000000	-2.567130	11.900463	0.444444
Bacteroides thetaiotaomicron	Adrenaline	0.100000	t_gr	14.750000	15.500000	0.750000	0.847403	0.209605	3.000000	-10.637307	12.137307	0.071429
Bacteroides thetaiotaomicron	Adrenaline	0.010000	t_gr	14.750000	18.583333	3.833333	0.245409	1.440316	3.000000	-4.636599	12.303265	0.365079
Bacteroides thetaiotaomicron	Adrenaline	0.001000	t_gr	14.750000	16.750000	2.000000	0.673610	0.464991	3.000000	-11.688219	15.688219	0.190476
Bacteroides thetaiotaomicron	Adrenaline	0.000100	t_gr	14.750000	19.750000	5.000000	0.171996	1.786474	3.000000	-3.907060	13.907060	0.476190
Bacteroides thetaiotaomicron	Adrenaline	0.000010	t_gr	14.750000	20.333333	5.583333	0.106432	2.285124	3.000000	-2.192463	13.359130	0.531746
Bacteroides thetaiotaomicron	Adrenaline	0.000001	t_gr	14.750000	20.333333	5.583333	0.097685	2.379240	3.000000	-1.884875	13.051542	0.531746
Bacteroides thetaiotaomicron	Levodopa	0.100000	t_dr	47.666667	35.416667	-12.250000	0.030917	-3.851125	3.000000	-22.373009	-2.126991	-1.000000
Bacteroides thetaiotaomicron	Levodopa	0.010000	t_dr	47.666667	45.833333	-1.833333	0.391002	-1.000000	3.000000	-7.667818	4.001152	-0.149660
Bacteroides thetaiotaomicron	Levodopa	0.001000	t_dr	47.666667	45.583333	-2.083333	0.391002	-1.000000	3.000000	-8.713430	4.546763	-0.170068
Bacteroides thetaiotaomicron	Levodopa	0.000100	t_dr	47.666667	44.833333	-2.833333	0.391002	-1.000000	3.000000	-11.850265	6.183598	-0.231293
Bacteroides thetaiotaomicron	Levodopa	0.000010	t_dr	47.666667	44.833333	-2.833333	0.391002	-1.000000	3.000000	-11.850265	6.183598	-0.231293
Bacteroides thetaiotaomicron	Levodopa	0.000001	t_dr	47.666667	44.500000	-3.166667	0.391002	-1.000000	3.000000	-13.244413	6.911080	-0.258503
Bacteroides thetaiotaomicron	Dopamine	0.100000	t_dr	48.000000	46.333333	-1.666667	0.391002	-1.000000	3.000000	-6.970744	3.637411	-0.136054

Species	Hormon	Concentration	Parameter	Mean_Baseline	Mean_Treatment	Diff	P_Value	T_Statistic	DF	Lower_CI	Upper_CI	Diff_Norm
Bacteroides thetaiotaomicron	Dopamine	0.010000	t_dr	48.000000	43.583333	-4.416667	0.187195	-1.702605	3.000000	-12.672135	3.838802	-0.360544
Bacteroides thetaiotaomicron	Dopamine	0.001000	t_dr	48.000000	36.250000	-11.750000	0.064653	-2.858565	3.000000	-24.831300	1.331300	-0.959184
Bacteroides thetaiotaomicron	Dopamine	0.000100	t_dr	48.000000	44.666667	-3.333333	0.320255	-1.188177	3.000000	-12.261426	5.594759	-0.272109
Bacteroides thetaiotaomicron	Dopamine	0.000010	t_dr	48.000000	46.666667	-1.333333	0.391002	-1.000000	3.000000	-5.576595	2.909928	-0.108844
Bacteroides thetaiotaomicron	Dopamine	0.000001	t_dr	48.000000	46.083333	-1.916667	0.391002	-1.000000	3.000000	-8.016355	4.183022	-0.156463
Bacteroides thetaiotaomicron	Noradrenaline	0.100000	t_dr	43.583333	42.083333	-1.500000	0.735114	-0.371259	3.000000	-14.358057	11.358057	-0.122449
Bacteroides thetaiotaomicron	Noradrenaline	0.010000	t_dr	43.583333	42.750000	-0.833333	0.747657	-0.352673	3.000000	-8.353161	6.686494	-0.068027
Bacteroides thetaiotaomicron	Noradrenaline	0.001000	t_dr	43.583333	46.583333	3.000000	0.595536	0.591943	3.000000	-13.128814	19.128814	0.244898
Bacteroides thetaiotaomicron	Noradrenaline	0.000100	t_dr	43.583333	41.750000	-1.833333	0.814534	-0.255952	3.000000	-24.628527	20.961860	-0.149660
Bacteroides thetaiotaomicron	Noradrenaline	0.000010	t_dr	43.583333	40.333333	-3.250000	0.527030	-0.713507	3.000000	-17.745924	11.245924	-0.265306
Bacteroides thetaiotaomicron	Noradrenaline	0.000001	t_dr	43.583333	42.083333	-1.500000	0.708145	-0.411794	3.000000	-13.092372	10.092372	-0.122449
Bacteroides thetaiotaomicron	Adrenaline	0.100000	t_dr	48.000000	47.333333	-0.666667	0.391002	-1.000000	3.000000	-2.788298	1.454964	-0.054422
Bacteroides thetaiotaomicron	Adrenaline	0.010000	t_dr	48.000000	42.750000	-5.250000	0.228089	-1.510454	3.000000	-16.311470	5.811470	-0.428571
Bacteroides thetaiotaomicron	Adrenaline	0.001000	t_dr	48.000000	44.500000	-3.500000	0.391002	-1.000000	3.000000	-14.638562	7.638562	-0.285714
Bacteroides thetaiotaomicron	Adrenaline	0.000100	t_dr	48.000000	44.916667	-3.083333	0.391002	-1.000000	3.000000	-12.895876	6.729209	-0.251701
Bacteroides thetaiotaomicron	Adrenaline	0.000010	t_dr	48.000000	42.083333	-5.916667	0.182258	-1.728967	3.000000	-16.807260	4.973927	-0.482993
Bacteroides thetaiotaomicron	Adrenaline	0.000001	t_dr	48.000000	42.333333	-5.666667	0.188500	-1.695766	3.000000	-16.301308	4.967975	-0.462585
Bifidobacterium stercoris	Levodopa	0.100000	auc_lin	9.240460	7.017443	-2.223017	0.156650	-1.880293	3.000000	-5.985532	1.539498	-0.450315
Bifidobacterium stercoris	Levodopa	0.010000	auc_lin	9.240460	6.063451	-3.177010	0.201392	-1.630977	3.000000	-9.376156	3.022137	-0.643564
Bifidobacterium stercoris	Levodopa	0.001000	auc_lin	9.240460	5.620477	-3.619983	0.142896	-1.973950	3.000000	-9.456203	2.216236	-0.733297
Bifidobacterium stercoris	Levodopa	0.000100	auc_lin	9.240460	5.837360	-3.403100	0.147856	-1.939000	3.000000	-8.988548	2.182348	-0.689363
Bifidobacterium stercoris	Levodopa	0.000010	auc_lin	9.240460	6.365167	-2.875294	0.222607	-1.533880	3.000000	-8.840866	3.090278	-0.582446
Bifidobacterium stercoris	Levodopa	0.000001	auc_lin	9.240460	7.069338	-2.171123	0.275945	-1.328790	3.000000	-7.370951	3.028706	-0.439802
Bifidobacterium stercoris	Dopamine	0.100000	auc_lin	10.637127	7.241692	-3.395435	0.528228	-0.711280	3.000000	-18.587477	11.796607	-0.687810
Bifidobacterium stercoris	Dopamine	0.010000	auc_lin	10.637127	5.700539	-4.936588	0.098285	-2.372464	3.000000	-11.558575	1.685399	-1.000000
Bifidobacterium stercoris	Dopamine	0.001000	auc_lin	10.637127	6.080308	-4.556819	0.140641	-1.990312	3.000000	-11.843027	2.729389	-0.923071
Bifidobacterium stercoris	Dopamine	0.000100	auc_lin	10.637127	7.927222	-2.709905	0.499769	-0.765341	3.000000	-13.978256	8.558445	-0.548943

Species	Hormon	Concentration	Parameter	Mean_Baseline	Mean_Treatment	Diff	P_Value	T_Statistic	DF	Lower_CI	Upper_CI	Diff_Norm
Bifidobacterium stercoris	Dopamine	0.000010	auc_lin	10.637127	5.854491	-4.782636	0.122378	-2.135457	3.000000	-11.910143	2.344871	-0.968814
Bifidobacterium stercoris	Dopamine	0.000001	auc_lin	10.637127	5.866496	-4.770632	0.128562	-2.083581	3.000000	-12.057259	2.515996	-0.966382
Bifidobacterium stercoris	Noradrenaline	0.100000	auc_lin	8.464101	5.790712	-2.673389	0.121563	-2.142520	3.000000	-6.644376	1.297598	-0.541546
Bifidobacterium stercoris	Noradrenaline	0.010000	auc_lin	8.464101	5.253582	-3.210519	0.125783	-2.106516	3.000000	-8.060852	1.639814	-0.650352
Bifidobacterium stercoris	Noradrenaline	0.001000	auc_lin	8.464101	5.861640	-2.602462	0.155869	-1.885346	3.000000	-6.995392	1.790468	-0.527178
Bifidobacterium stercoris	Noradrenaline	0.000100	auc_lin	8.464101	5.232809	-3.231292	0.177714	-1.753964	3.000000	-9.094249	2.631665	-0.654560
Bifidobacterium stercoris	Noradrenaline	0.000010	auc_lin	8.464101	5.746307	-2.717795	0.198891	-1.643171	3.000000	-7.981540	2.545951	-0.550541
Bifidobacterium stercoris	Noradrenaline	0.000001	auc_lin	8.464101	6.160162	-2.303940	0.202678	-1.624773	3.000000	-6.816672	2.208793	-0.466707
Bifidobacterium stercoris	Adrenaline	0.100000	auc_lin	10.363149	8.014583	-2.348566	0.191867	-1.678372	3.000000	-6.801803	2.104670	-0.475747
Bifidobacterium stercoris	Adrenaline	0.010000	auc_lin	10.363149	8.090831	-2.272318	0.392273	-0.996931	3.000000	-9.526107	4.981471	-0.460301
Bifidobacterium stercoris	Adrenaline	0.001000	auc_lin	10.363149	6.441936	-3.921212	0.163434	-1.837561	3.000000	-10.712306	2.869881	-0.794316
Bifidobacterium stercoris	Adrenaline	0.000100	auc_lin	10.363149	6.396841	-3.966308	0.115828	-2.193926	3.000000	-9.719722	1.787106	-0.803451
Bifidobacterium stercoris	Adrenaline	0.000010	auc_lin	10.363149	7.092692	-3.270457	0.058467	-2.982777	3.000000	-6.759841	0.218926	-0.662493
Bifidobacterium stercoris	Adrenaline	0.000001	auc_lin	10.363149	6.627714	-3.735435	0.088560	-2.489028	3.000000	-8.511525	1.040655	-0.756684
Bifidobacterium stercoris	Levodopa	0.100000	k_lin	0.305180	0.243039	-0.062141	0.148294	-1.935982	3.000000	-0.164290	0.040009	-0.402008
Bifidobacterium stercoris	Levodopa	0.010000	k_lin	0.305180	0.214957	-0.090223	0.169852	-1.798988	3.000000	-0.249829	0.069383	-0.583680
Bifidobacterium stercoris	Levodopa	0.001000	k_lin	0.305180	0.192195	-0.112985	0.081955	-2.577532	3.000000	-0.252486	0.026516	-0.730935
Bifidobacterium stercoris	Levodopa	0.000100	k_lin	0.305180	0.194975	-0.110204	0.060798	-2.934120	3.000000	-0.229736	0.009327	-0.712948
Bifidobacterium stercoris	Levodopa	0.000010	k_lin	0.305180	0.212891	-0.092289	0.140937	-1.988152	3.000000	-0.240016	0.055439	-0.597047
Bifidobacterium stercoris	Levodopa	0.000001	k_lin	0.305180	0.243192	-0.061988	0.235204	-1.480961	3.000000	-0.195195	0.071219	-0.401022
Bifidobacterium stercoris	Dopamine	0.100000	k_lin	0.372224	0.267694	-0.104531	0.469781	-0.825147	3.000000	-0.507688	0.298626	-0.676244
Bifidobacterium stercoris	Dopamine	0.010000	k_lin	0.372224	0.218089	-0.154135	0.111821	-2.231685	3.000000	-0.373936	0.065666	-0.997149
Bifidobacterium stercoris	Dopamine	0.001000	k_lin	0.372224	0.225630	-0.146595	0.143190	-1.971837	3.000000	-0.383191	0.090002	-0.948368
Bifidobacterium stercoris	Dopamine	0.000100	k_lin	0.372224	0.292202	-0.080022	0.553636	-0.664942	3.000000	-0.463011	0.302967	-0.517688
Bifidobacterium stercoris	Dopamine	0.000010	k_lin	0.372224	0.222326	-0.149899	0.140104	-1.994255	3.000000	-0.389108	0.089311	-0.969744
Bifidobacterium stercoris	Dopamine	0.000001	k_lin	0.372224	0.217649	-0.154576	0.133173	-2.046801	3.000000	-0.394916	0.085765	-1.000000
Bifidobacterium stercoris	Noradrenaline	0.100000	k_lin	0.297279	0.246103	-0.051176	0.298003	-1.256083	3.000000	-0.180839	0.078486	-0.331077

Species	Hormon	Concentration	Parameter	Mean_Baseline	Mean_Treatment	Diff	P_Value	T_Statistic	DF	Lower_CI	Upper_CI	Diff_Norm
Bifidobacterium stercoris	Noradrenaline	0.010000	k_lin	0.297279	0.197083	-0.100197	0.110056	-2.248840	3.000000	-0.241990	0.041597	-0.648206
Bifidobacterium stercoris	Noradrenaline	0.001000	k_lin	0.297279	0.218973	-0.078307	0.128906	-2.080781	3.000000	-0.198073	0.041459	-0.506591
Bifidobacterium stercoris	Noradrenaline	0.000100	k_lin	0.297279	0.204306	-0.092973	0.204600	-1.615582	3.000000	-0.276115	0.090169	-0.601472
Bifidobacterium stercoris	Noradrenaline	0.000010	k_lin	0.297279	0.221070	-0.076210	0.230873	-1.498798	3.000000	-0.238028	0.085609	-0.493025
Bifidobacterium stercoris	Noradrenaline	0.000001	k_lin	0.297279	0.233790	-0.063490	0.264975	-1.367266	3.000000	-0.211269	0.084289	-0.410737
Bifidobacterium stercoris	Adrenaline	0.100000	k_lin	0.326072	0.278014	-0.048058	0.164996	-1.828014	3.000000	-0.131724	0.035608	-0.310903
Bifidobacterium stercoris	Adrenaline	0.010000	k_lin	0.326072	0.281782	-0.044290	0.498627	-0.767563	3.000000	-0.227924	0.139344	-0.286527
Bifidobacterium stercoris	Adrenaline	0.001000	k_lin	0.326072	0.232215	-0.093857	0.185377	-1.712216	3.000000	-0.268306	0.080592	-0.607191
Bifidobacterium stercoris	Adrenaline	0.000100	k_lin	0.326072	0.234868	-0.091204	0.154164	-1.896492	3.000000	-0.244249	0.061842	-0.590025
Bifidobacterium stercoris	Adrenaline	0.000010	k_lin	0.326072	0.260530	-0.065542	0.108924	-2.260023	3.000000	-0.157835	0.026751	-0.424012
Bifidobacterium stercoris	Adrenaline	0.000001	k_lin	0.326072	0.247325	-0.078747	0.161488	-1.849604	3.000000	-0.214239	0.056746	-0.509437
Bifidobacterium stercoris	Levodopa	0.100000	death_lin	0.046700	0.061334	0.014634	0.673266	0.465528	3.000000	-0.085406	0.114674	0.298624
Bifidobacterium stercoris	Levodopa	0.010000	death_lin	0.046700	0.059401	0.012701	0.576231	0.625089	3.000000	-0.051961	0.077362	0.259174
Bifidobacterium stercoris	Levodopa	0.001000	death_lin	0.046700	0.039311	-0.007390	0.707732	-0.412421	3.000000	-0.064413	0.049633	-0.150798
Bifidobacterium stercoris	Levodopa	0.000100	death_lin	0.046700	0.037038	-0.009663	0.705607	-0.415652	3.000000	-0.083645	0.064320	-0.197180
Bifidobacterium stercoris	Levodopa	0.000010	death_lin	0.046700	0.043354	-0.003346	0.880918	-0.162946	3.000000	-0.068692	0.062000	-0.068276
Bifidobacterium stercoris	Levodopa	0.000001	death_lin	0.046700	0.052220	0.005520	0.815840	0.254098	3.000000	-0.063612	0.074651	0.112637
Bifidobacterium stercoris	Dopamine	0.100000	death_lin	0.075283	0.026279	-0.049004	0.348399	-1.108840	3.000000	-0.189650	0.091641	-1.000000
Bifidobacterium stercoris	Dopamine	0.010000	death_lin	0.075283	0.043130	-0.032153	0.564688	-0.645299	3.000000	-0.190720	0.126415	-0.656116
Bifidobacterium stercoris	Dopamine	0.001000	death_lin	0.075283	0.046530	-0.028753	0.662478	-0.482484	3.000000	-0.218408	0.160902	-0.586749
Bifidobacterium stercoris	Dopamine	0.000100	death_lin	0.075283	0.066443	-0.008840	0.895206	-0.143205	3.000000	-0.205298	0.187617	-0.180397
Bifidobacterium stercoris	Dopamine	0.000010	death_lin	0.075283	0.040988	-0.034295	0.569975	-0.636004	3.000000	-0.205902	0.137312	-0.699841
Bifidobacterium stercoris	Dopamine	0.000001	death_lin	0.075283	0.036607	-0.038676	0.514203	-0.737618	3.000000	-0.205545	0.128192	-0.789244
Bifidobacterium stercoris	Noradrenaline	0.100000	death_lin	0.055786	0.048037	-0.007750	0.731903	-0.376043	3.000000	-0.073336	0.057836	-0.158145
Bifidobacterium stercoris	Noradrenaline	0.010000	death_lin	0.055786	0.037623	-0.018164	0.482176	-0.800047	3.000000	-0.090416	0.054088	-0.370656
Bifidobacterium stercoris	Noradrenaline	0.001000	death_lin	0.055786	0.050514	-0.005272	0.828130	-0.236701	3.000000	-0.076153	0.065610	-0.107582
Bifidobacterium stercoris	Noradrenaline	0.000100	death_lin	0.055786	0.047824	-0.007962	0.680410	-0.454391	3.000000	-0.063725	0.047801	-0.162473

Species	Hormon	Concentration	Parameter	Mean_Baseline	Mean_Treatment	Diff	P_Value	T_Statistic	DF	Lower_CI	Upper_CI	Diff_Norm
Bifidobacterium stercoris	Noradrenaline	0.000010	death_lin	0.055786	0.048056	-0.007731	0.745419	-0.355978	3.000000	-0.076845	0.061383	-0.157759
Bifidobacterium stercoris	Noradrenaline	0.000001	death_lin	0.055786	0.046739	-0.009048	0.693895	-0.433556	3.000000	-0.075462	0.057366	-0.184633
Bifidobacterium stercoris	Adrenaline	0.100000	death_lin	0.025293	0.048191	0.022898	0.430242	0.909276	3.000000	-0.057245	0.103041	0.467268
Bifidobacterium stercoris	Adrenaline	0.010000	death_lin	0.025293	0.063689	0.038396	0.063497	2.880616	3.000000	-0.004023	0.080814	0.783516
Bifidobacterium stercoris	Adrenaline	0.001000	death_lin	0.025293	0.042050	0.016756	0.292980	1.272133	3.000000	-0.025162	0.058675	0.341936
Bifidobacterium stercoris	Adrenaline	0.000100	death_lin	0.025293	0.038943	0.013649	0.494399	0.775825	3.000000	-0.042341	0.069640	0.278536
Bifidobacterium stercoris	Adrenaline	0.000010	death_lin	0.025293	0.056101	0.030808	0.432787	0.903658	3.000000	-0.077688	0.139303	0.628669
Bifidobacterium stercoris	Adrenaline	0.000001	death_lin	0.025293	0.039573	0.014280	0.480305	0.803798	3.000000	-0.042257	0.070817	0.291397
Bifidobacterium stercoris	Levodopa	0.100000	gr	0.307179	0.220396	-0.086783	0.018670	-4.657954	3.000000	-0.146075	-0.027490	-0.483148
Bifidobacterium stercoris	Levodopa	0.010000	gr	0.307179	0.244558	-0.062620	0.084554	-2.541692	3.000000	-0.141027	0.015786	-0.348627
Bifidobacterium stercoris	Levodopa	0.001000	gr	0.307179	0.220340	-0.086838	0.031311	-3.832347	3.000000	-0.158950	-0.014726	-0.483456
Bifidobacterium stercoris	Levodopa	0.000100	gr	0.307179	0.235679	-0.071500	0.042411	-3.401615	3.000000	-0.138393	-0.004607	-0.398064
Bifidobacterium stercoris	Levodopa	0.000010	gr	0.307179	0.258441	-0.048737	0.290032	-1.281687	3.000000	-0.169753	0.072278	-0.271337
Bifidobacterium stercoris	Levodopa	0.000001	gr	0.307179	0.296702	-0.010476	0.694630	-0.432427	3.000000	-0.087576	0.066623	-0.058324
Bifidobacterium stercoris	Dopamine	0.100000	gr	0.272011	0.324821	0.052810	0.812053	0.259480	3.000000	-0.594891	0.700511	0.294010
Bifidobacterium stercoris	Dopamine	0.010000	gr	0.272011	0.191337	-0.080673	0.311875	-1.213164	3.000000	-0.292300	0.130953	-0.449133
Bifidobacterium stercoris	Dopamine	0.001000	gr	0.272011	0.205265	-0.066746	0.427435	-0.915507	3.000000	-0.298766	0.165274	-0.371596
Bifidobacterium stercoris	Dopamine	0.000100	gr	0.272011	0.222682	-0.049328	0.600187	-0.584075	3.000000	-0.318103	0.219447	-0.274626
Bifidobacterium stercoris	Dopamine	0.000010	gr	0.272011	0.193861	-0.078149	0.316408	-1.199566	3.000000	-0.285479	0.129181	-0.435082
Bifidobacterium stercoris	Dopamine	0.000001	gr	0.272011	0.213748	-0.058263	0.453500	-0.859002	3.000000	-0.274117	0.157591	-0.324368
Bifidobacterium stercoris	Noradrenaline	0.100000	gr	0.281041	0.213766	-0.067274	0.026850	-4.064970	3.000000	-0.119943	-0.014606	-0.374537
Bifidobacterium stercoris	Noradrenaline	0.010000	gr	0.281041	0.197033	-0.084008	0.019758	-4.561267	3.000000	-0.142621	-0.025395	-0.467698
Bifidobacterium stercoris	Noradrenaline	0.001000	gr	0.281041	0.217552	-0.063489	0.053454	-3.096189	3.000000	-0.128746	0.001769	-0.353463
Bifidobacterium stercoris	Noradrenaline	0.000100	gr	0.281041	0.214641	-0.066400	0.099506	-2.358825	3.000000	-0.155984	0.023185	-0.369669
Bifidobacterium stercoris	Noradrenaline	0.000010	gr	0.281041	0.221996	-0.059045	0.407273	-0.961399	3.000000	-0.254496	0.136406	-0.328720
Bifidobacterium stercoris	Noradrenaline	0.000001	gr	0.281041	0.224161	-0.056880	0.342456	-1.125045	3.000000	-0.217777	0.104018	-0.316667
Bifidobacterium stercoris	Adrenaline	0.100000	gr	0.384922	0.233442	-0.151480	0.280564	-1.313072	3.000000	-0.518617	0.215657	-0.843338

Species	Hormon	Concentration	Parameter	Mean_Baseline	Mean_Treatment	Diff	P_Value	T_Statistic	DF	Lower_CI	Upper_CI	Diff_Norm
Bifidobacterium stercoris	Adrenaline	0.010000	gr	0.384922	0.259319	-0.125603	0.367914	-1.057471	3.000000	-0.503604	0.252398	-0.699272
Bifidobacterium stercoris	Adrenaline	0.001000	gr	0.384922	0.219327	-0.165595	0.244877	-1.442389	3.000000	-0.530958	0.199769	-0.921917
Bifidobacterium stercoris	Adrenaline	0.000100	gr	0.384922	0.224281	-0.160641	0.248343	-1.428968	3.000000	-0.518404	0.197122	-0.894340
Bifidobacterium stercoris	Adrenaline	0.000010	gr	0.384922	0.205302	-0.179620	0.200661	-1.634526	3.000000	-0.529342	0.170103	-1.000000
Bifidobacterium stercoris	Adrenaline	0.000001	gr	0.384922	0.221179	-0.163743	0.279593	-1.316353	3.000000	-0.559611	0.232125	-0.911606
Bifidobacterium stercoris	Levodopa	0.100000	dr	-0.006394	-0.023338	-0.016943	0.306065	-1.230895	3.000000	-0.060749	0.026863	-1.000000
Bifidobacterium stercoris	Levodopa	0.010000	dr	-0.006394	-0.017731	-0.011337	0.351246	-1.101171	3.000000	-0.044101	0.021427	-0.669103
Bifidobacterium stercoris	Levodopa	0.001000	dr	-0.006394	-0.012144	-0.005750	0.564108	-0.646322	3.000000	-0.034062	0.022562	-0.339360
Bifidobacterium stercoris	Levodopa	0.000100	dr	-0.006394	-0.016344	-0.009950	0.441985	-0.883597	3.000000	-0.045785	0.025886	-0.587232
Bifidobacterium stercoris	Levodopa	0.000010	dr	-0.006394	-0.015726	-0.009331	0.390149	-1.002067	3.000000	-0.038966	0.020304	-0.550734
Bifidobacterium stercoris	Levodopa	0.000001	dr	-0.006394	-0.018211	-0.011816	0.331693	-1.155123	3.000000	-0.044371	0.020738	-0.697400
Bifidobacterium stercoris	Dopamine	0.100000	dr	-0.014608	-0.020289	-0.005681	0.696838	-0.429040	3.000000	-0.047820	0.036458	-0.335292
Bifidobacterium stercoris	Dopamine	0.010000	dr	-0.014608	-0.017078	-0.002470	0.851315	-0.204131	3.000000	-0.040976	0.036036	-0.145775
Bifidobacterium stercoris	Dopamine	0.001000	dr	-0.014608	-0.021918	-0.007310	0.496012	-0.772665	3.000000	-0.037416	0.022797	-0.431417
Bifidobacterium stercoris	Dopamine	0.000100	dr	-0.014608	-0.021165	-0.006557	0.584971	-0.609984	3.000000	-0.040768	0.027653	-0.387009
Bifidobacterium stercoris	Dopamine	0.000010	dr	-0.014608	-0.018549	-0.003941	0.744892	-0.356755	3.000000	-0.039094	0.031213	-0.232584
Bifidobacterium stercoris	Dopamine	0.000001	dr	-0.014608	-0.019742	-0.005134	0.733485	-0.373684	3.000000	-0.048853	0.038586	-0.302986
Bifidobacterium stercoris	Noradrenaline	0.100000	dr	-0.022530	-0.017818	0.004712	0.690231	0.439193	3.000000	-0.029429	0.038852	0.278079
Bifidobacterium stercoris	Noradrenaline	0.010000	dr	-0.022530	-0.016820	0.005710	0.662959	0.481724	3.000000	-0.032011	0.043431	0.336998
Bifidobacterium stercoris	Noradrenaline	0.001000	dr	-0.022530	-0.015978	0.006552	0.582211	0.614736	3.000000	-0.027368	0.040472	0.386708
Bifidobacterium stercoris	Noradrenaline	0.000100	dr	-0.022530	-0.018050	0.004480	0.624987	0.542818	3.000000	-0.021783	0.030742	0.264384
Bifidobacterium stercoris	Noradrenaline	0.000010	dr	-0.022530	-0.016948	0.005581	0.578129	0.621794	3.000000	-0.022985	0.034147	0.329413
Bifidobacterium stercoris	Noradrenaline	0.000001	dr	-0.022530	-0.023547	-0.001017	0.927212	-0.099234	3.000000	-0.033629	0.031595	-0.060017
Bifidobacterium stercoris	Adrenaline	0.100000	dr	-0.015562	-0.016883	-0.001321	0.939439	-0.082509	3.000000	-0.052267	0.049625	-0.077957
Bifidobacterium stercoris	Adrenaline	0.010000	dr	-0.015562	-0.024001	-0.008439	0.588307	-0.604262	3.000000	-0.052885	0.036007	-0.498084
Bifidobacterium stercoris	Adrenaline	0.001000	dr	-0.015562	-0.014732	0.000830	0.957036	0.058491	3.000000	-0.044311	0.045970	0.048966
Bifidobacterium stercoris	Adrenaline	0.000100	dr	-0.015562	-0.021714	-0.006152	0.708850	-0.410724	3.000000	-0.053822	0.041518	-0.363111

Species	Hormon	Concentration	Parameter	Mean_Baseline	Mean_Treatment	Diff	P_Value	T_Statistic	DF	Lower_CI	Upper_CI	Diff_Norm
Bifidobacterium stercoris	Adrenaline	0.000010	dr	-0.015562	-0.016976	-0.001414	0.923152	-0.104795	3.000000	-0.044345	0.041518	-0.083437
Bifidobacterium stercoris	Adrenaline	0.000001	dr	-0.015562	-0.018205	-0.002643	0.877414	-0.167801	3.000000	-0.052776	0.047489	-0.156012
Bifidobacterium stercoris	Levodopa	0.100000	td	2.352131	3.250089	0.897958	0.013900	5.188876	3.000000	0.347222	1.448695	0.761551
Bifidobacterium stercoris	Levodopa	0.010000	td	2.352131	3.026917	0.674785	0.149925	1.924827	3.000000	-0.440883	1.790453	0.572280
Bifidobacterium stercoris	Levodopa	0.001000	td	2.352131	3.367868	1.015736	0.074405	2.690149	3.000000	-0.185880	2.217352	0.861438
Bifidobacterium stercoris	Levodopa	0.000100	td	2.352131	3.109117	0.756985	0.082629	2.568106	3.000000	-0.181085	1.695056	0.641994
Bifidobacterium stercoris	Levodopa	0.000010	td	2.352131	2.803079	0.450948	0.296136	1.262014	3.000000	-0.686216	1.588111	0.382445
Bifidobacterium stercoris	Levodopa	0.000001	td	2.352131	2.382918	0.030787	0.875441	0.170537	3.000000	-0.543739	0.605312	0.026110
Bifidobacterium stercoris	Dopamine	0.100000	td	3.003648	4.050436	1.046788	0.446911	0.873006	3.000000	-2.769160	4.862736	0.887773
Bifidobacterium stercoris	Dopamine	0.010000	td	3.003648	3.926100	0.922452	0.283300	1.303889	3.000000	-1.329008	3.173912	0.782325
Bifidobacterium stercoris	Dopamine	0.001000	td	3.003648	3.731885	0.728237	0.497949	0.768883	3.000000	-2.285974	3.742447	0.617612
Bifidobacterium stercoris	Dopamine	0.000100	td	3.003648	3.468976	0.465328	0.682511	0.451128	3.000000	-2.817291	3.747948	0.394641
Bifidobacterium stercoris	Dopamine	0.000010	td	3.003648	4.030912	1.027264	0.241846	1.454293	3.000000	-1.220709	3.275236	0.871214
Bifidobacterium stercoris	Dopamine	0.000001	td	3.003648	3.330887	0.327239	0.726806	0.383659	3.000000	-2.387201	3.041679	0.277529
Bifidobacterium stercoris	Noradrenaline	0.100000	td	2.814014	3.683524	0.869510	0.041491	3.431536	3.000000	0.063116	1.675904	0.737425
Bifidobacterium stercoris	Noradrenaline	0.010000	td	2.814014	3.993130	1.179117	0.027427	4.032183	3.000000	0.248485	2.109748	1.000000
Bifidobacterium stercoris	Noradrenaline	0.001000	td	2.814014	3.594259	0.780245	0.038090	3.550043	3.000000	0.080792	1.479698	0.661720
Bifidobacterium stercoris	Noradrenaline	0.000100	td	2.814014	3.479023	0.665009	0.125713	2.107098	3.000000	-0.339385	1.669403	0.563989
Bifidobacterium stercoris	Noradrenaline	0.000010	td	2.814014	3.335937	0.521923	0.489973	0.784536	3.000000	-1.595243	2.639089	0.442639
Bifidobacterium stercoris	Noradrenaline	0.000001	td	2.814014	3.202401	0.388388	0.542293	0.685420	3.000000	-1.414918	2.191693	0.329388
Bifidobacterium stercoris	Adrenaline	0.100000	td	2.447370	3.531798	1.084428	0.117594	2.177773	3.000000	-0.500280	2.669135	0.919695
Bifidobacterium stercoris	Adrenaline	0.010000	td	2.447370	2.944014	0.496644	0.403337	0.970604	3.000000	-1.131767	2.125055	0.421200
Bifidobacterium stercoris	Adrenaline	0.001000	td	2.447370	3.570304	1.122933	0.082432	2.570858	3.000000	-0.267137	2.513004	0.952351
Bifidobacterium stercoris	Adrenaline	0.000100	td	2.447370	3.214801	0.767431	0.248198	1.429525	3.000000	-0.941044	2.475906	0.650852
Bifidobacterium stercoris	Adrenaline	0.000010	td	2.447370	3.514297	1.066927	0.161478	1.849665	3.000000	-0.768777	2.902631	0.904853
Bifidobacterium stercoris	Adrenaline	0.000001	td	2.447370	3.232544	0.785173	0.449991	0.866438	3.000000	-2.098787	3.669133	0.665900
Bifidobacterium stercoris	Levodopa	0.100000	lagC	8.938673	11.562497	2.623823	0.024699	4.195702	3.000000	0.633649	4.613998	0.425936

Species	Hormon	Concentration	Parameter	Mean_Baseline	Mean_Treatment	Diff	P_Value	T_Statistic	DF	Lower_CI	Upper_CI	Diff_Norm
Bifidobacterium stercoris	Levodopa	0.010000	lagC	8.938673	11.591756	2.653082	0.041128	3.443610	3.000000	0.201210	5.104955	0.430686
Bifidobacterium stercoris	Levodopa	0.001000	lagC	8.938673	11.284566	2.345893	0.060712	2.935866	3.000000	-0.197029	4.888815	0.380819
Bifidobacterium stercoris	Levodopa	0.000100	lagC	8.938673	11.240604	2.301931	0.047319	3.254746	3.000000	0.051134	4.552728	0.373682
Bifidobacterium stercoris	Levodopa	0.000010	lagC	8.938673	10.933349	1.994675	0.066699	2.820710	3.000000	-0.255803	4.245154	0.323804
Bifidobacterium stercoris	Levodopa	0.000001	lagC	8.938673	10.871070	1.932397	0.056197	3.032549	3.000000	-0.095517	3.960311	0.313694
Bifidobacterium stercoris	Dopamine	0.100000	lagC	10.248176	16.408308	6.160133	0.183617	1.721630	3.000000	-5.226918	17.547184	1.000000
Bifidobacterium stercoris	Dopamine	0.010000	lagC	10.248176	13.591575	3.343399	0.043859	3.356133	3.000000	0.173028	6.513771	0.542748
Bifidobacterium stercoris	Dopamine	0.001000	lagC	10.248176	12.834101	2.585926	0.022559	4.341248	3.000000	0.690256	4.481595	0.419784
Bifidobacterium stercoris	Dopamine	0.000100	lagC	10.248176	11.983537	1.735361	0.094215	2.419454	3.000000	-0.547259	4.017981	0.281708
Bifidobacterium stercoris	Dopamine	0.000010	lagC	10.248176	12.312444	2.064269	0.038786	3.524717	3.000000	0.200453	3.928084	0.335101
Bifidobacterium stercoris	Dopamine	0.000001	lagC	10.248176	13.003188	2.755012	0.098272	2.372606	3.000000	-0.940367	6.450391	0.447233
Bifidobacterium stercoris	Noradrenaline	0.100000	lagC	10.989162	16.740009	5.750846	0.048645	3.218370	3.000000	0.064192	11.437501	0.933559
Bifidobacterium stercoris	Noradrenaline	0.010000	lagC	10.989162	12.526082	1.536919	0.253432	1.409627	3.000000	-1.932909	5.006747	0.249495
Bifidobacterium stercoris	Noradrenaline	0.001000	lagC	10.989162	13.222950	2.233788	0.067594	2.804603	3.000000	-0.300942	4.768518	0.362620
Bifidobacterium stercoris	Noradrenaline	0.000100	lagC	10.989162	13.883535	2.894372	0.101032	2.342061	3.000000	-1.038568	6.827312	0.469855
Bifidobacterium stercoris	Noradrenaline	0.000010	lagC	10.989162	13.862482	2.873320	0.138715	2.004534	3.000000	-1.688432	7.435071	0.466438
Bifidobacterium stercoris	Noradrenaline	0.000001	lagC	10.989162	13.208901	2.219739	0.213022	1.576410	3.000000	-2.261455	6.700932	0.360339
Bifidobacterium stercoris	Adrenaline	0.100000	lagC	9.959236	11.058485	1.099249	0.261917	1.378294	3.000000	-1.438889	3.637387	0.178446
Bifidobacterium stercoris	Adrenaline	0.010000	lagC	9.959236	11.278274	1.319038	0.200173	1.636901	3.000000	-1.245422	3.883499	0.214125
Bifidobacterium stercoris	Adrenaline	0.001000	lagC	9.959236	11.250378	1.291142	0.329404	1.161644	3.000000	-2.246078	4.828362	0.209596
Bifidobacterium stercoris	Adrenaline	0.000100	lagC	9.959236	12.883577	2.924342	0.095674	2.402325	3.000000	-0.949639	6.798322	0.474721
Bifidobacterium stercoris	Adrenaline	0.000010	lagC	9.959236	12.188200	2.228964	0.165876	1.822681	3.000000	-1.662863	6.120791	0.361837
Bifidobacterium stercoris	Adrenaline	0.000001	lagC	9.959236	12.530784	2.571548	0.186230	1.707695	3.000000	-2.220768	7.363864	0.417450
Bifidobacterium stercoris	Levodopa	0.100000	t_k	32.583333	20.833333	-11.750000	0.228942	-1.506866	3.000000	-36.565581	13.065581	-0.754011
Bifidobacterium stercoris	Levodopa	0.010000	t_k	32.583333	18.833333	-13.750000	0.223955	-1.528062	3.000000	-42.386696	14.886696	-0.882353
Bifidobacterium stercoris	Levodopa	0.001000	t_k	32.583333	26.333333	-6.250000	0.477000	-0.810460	3.000000	-30.791987	18.291987	-0.401070
Bifidobacterium stercoris	Levodopa	0.000100	t_k	32.583333	25.583333	-7.000000	0.430347	-0.909043	3.000000	-31.506138	17.506138	-0.449198

Species	Hormon	Concentration	Parameter	Mean_Baseline	Mean_Treatment	Diff	P_Value	T_Statistic	DF	Lower_CI	Upper_CI	Diff_Norm
Bifidobacterium stercoris	Levodopa	0.000010	t_k	32.583333	17.833333	-14.750000	0.203476	-1.620945	3.000000	-43.709079	14.209079	-0.946524
Bifidobacterium stercoris	Levodopa	0.000001	t_k	32.583333	17.000000	-15.583333	0.195517	-1.659901	3.000000	-45.460483	14.293817	-1.000000
Bifidobacterium stercoris	Dopamine	0.100000	t_k	32.166667	33.000000	0.833333	0.933499	0.090630	3.000000	-28.428961	30.095628	0.053476
Bifidobacterium stercoris	Dopamine	0.010000	t_k	32.166667	23.250000	-8.916667	0.324938	-1.174501	3.000000	-33.077403	15.244070	-0.572193
Bifidobacterium stercoris	Dopamine	0.001000	t_k	32.166667	26.583333	-5.583333	0.536738	-0.695568	3.000000	-31.128858	19.962191	-0.358289
Bifidobacterium stercoris	Dopamine	0.000100	t_k	32.166667	24.250000	-7.916667	0.344854	-1.118473	3.000000	-30.442348	14.609015	-0.508021
Bifidobacterium stercoris	Dopamine	0.000010	t_k	32.166667	25.500000	-6.666667	0.430467	-0.908778	3.000000	-30.012641	16.679308	-0.427807
Bifidobacterium stercoris	Dopamine	0.000001	t_k	32.166667	24.916667	-7.250000	0.402131	-0.973440	3.000000	-30.952263	16.452263	-0.465241
Bifidobacterium stercoris	Noradrenaline	0.100000	t_k	30.416667	27.583333	-2.833333	0.736662	-0.368955	3.000000	-27.272412	21.605745	-0.181818
Bifidobacterium stercoris	Noradrenaline	0.010000	t_k	30.416667	22.666667	-7.750000	0.403850	-0.969399	3.000000	-33.192529	17.692529	-0.497326
Bifidobacterium stercoris	Noradrenaline	0.001000	t_k	30.416667	22.166667	-8.250000	0.360875	-1.075685	3.000000	-32.657882	16.157882	-0.529412
Bifidobacterium stercoris	Noradrenaline	0.000100	t_k	30.416667	22.000000	-8.416667	0.348867	-1.107574	3.000000	-32.600680	15.767347	-0.540107
Bifidobacterium stercoris	Noradrenaline	0.000010	t_k	30.416667	22.000000	-8.416667	0.344148	-1.120402	3.000000	-32.323781	15.490447	-0.540107
Bifidobacterium stercoris	Noradrenaline	0.000001	t_k	30.416667	21.916667	-8.500000	0.333115	-1.151092	3.000000	-32.000115	15.000115	-0.545455
Bifidobacterium stercoris	Adrenaline	0.100000	t_k	33.833333	24.000000	-9.833333	0.378141	-1.031601	3.000000	-40.168760	20.502093	-0.631016
Bifidobacterium stercoris	Adrenaline	0.010000	t_k	33.833333	20.916667	-12.916667	0.179470	-1.744217	3.000000	-36.484025	10.650692	-0.828877
Bifidobacterium stercoris	Adrenaline	0.001000	t_k	33.833333	28.166667	-5.666667	0.565513	-0.643844	3.000000	-33.676346	22.343013	-0.363636
Bifidobacterium stercoris	Adrenaline	0.000100	t_k	33.833333	20.916667	-12.916667	0.174306	-1.773195	3.000000	-36.098882	10.265549	-0.828877
Bifidobacterium stercoris	Adrenaline	0.000010	t_k	33.833333	21.083333	-12.750000	0.183818	-1.720550	3.000000	-36.333270	10.833270	-0.818182
Bifidobacterium stercoris	Adrenaline	0.000001	t_k	33.833333	28.583333	-5.250000	0.592324	-0.597405	3.000000	-33.217378	22.717378	-0.336898
Bifidobacterium stercoris	Levodopa	0.100000	t_gr	11.500000	14.166667	2.666667	0.020544	4.495612	3.000000	0.778932	4.554402	0.385542
Bifidobacterium stercoris	Levodopa	0.010000	t_gr	11.500000	14.083333	2.583333	0.037719	3.563768	3.000000	0.276415	4.890251	0.373494
Bifidobacterium stercoris	Levodopa	0.001000	t_gr	11.500000	14.000000	2.500000	0.026979	4.057513	3.000000	0.539165	4.460835	0.361446
Bifidobacterium stercoris	Levodopa	0.000100	t_gr	11.500000	13.250000	1.750000	0.091040	2.457864	3.000000	-0.515903	4.015903	0.253012
Bifidobacterium stercoris	Levodopa	0.000010	t_gr	11.500000	13.333333	1.833333	0.045168	3.316625	3.000000	0.074170	3.592497	0.265060
Bifidobacterium stercoris	Levodopa	0.000001	t_gr	11.500000	13.250000	1.750000	0.053919	3.085123	3.000000	-0.055205	3.555205	0.253012
Bifidobacterium stercoris	Dopamine	0.100000	t_gr	13.583333	19.916667	6.333333	0.267652	1.357720	3.000000	-8.511767	21.178434	0.915663

Species	Hormon	Concentration	Parameter	Mean_Baseline	Mean_Treatment	Diff	P_Value	T_Statistic	DF	Lower_CI	Upper_CI	Diff_Norm
Bifidobacterium stercoris	Dopamine	0.010000	t_gr	13.583333	16.750000	3.166667	0.054615	3.068777	3.000000	-0.117295	6.450629	0.457831
Bifidobacterium stercoris	Dopamine	0.001000	t_gr	13.583333	15.750000	2.166667	0.039015	3.516512	3.000000	0.205831	4.127502	0.313253
Bifidobacterium stercoris	Dopamine	0.000100	t_gr	13.583333	15.166667	1.583333	0.145386	1.956235	3.000000	-0.992468	4.159135	0.228916
Bifidobacterium stercoris	Dopamine	0.000010	t_gr	13.583333	15.666667	2.083333	0.087384	2.504177	3.000000	-0.564282	4.730948	0.301205
Bifidobacterium stercoris	Dopamine	0.000001	t_gr	13.583333	16.250000	2.666667	0.165109	1.827329	3.000000	-1.977557	7.310890	0.385542
Bifidobacterium stercoris	Noradrenaline	0.100000	t_gr	13.916667	20.833333	6.916667	0.061830	2.913327	3.000000	-0.638929	14.472262	1.000000
Bifidobacterium stercoris	Noradrenaline	0.010000	t_gr	13.916667	15.833333	1.916667	0.068077	2.796021	3.000000	-0.264894	4.098227	0.277108
Bifidobacterium stercoris	Noradrenaline	0.001000	t_gr	13.916667	17.500000	3.583333	0.114441	2.206822	3.000000	-1.584174	8.750840	0.518072
Bifidobacterium stercoris	Noradrenaline	0.000100	t_gr	13.916667	17.666667	3.750000	0.156371	1.882093	3.000000	-2.590905	10.090905	0.542169
Bifidobacterium stercoris	Noradrenaline	0.000010	t_gr	13.916667	17.083333	3.166667	0.195490	1.660038	3.000000	-2.904127	9.237460	0.457831
Bifidobacterium stercoris	Noradrenaline	0.000001	t_gr	13.916667	16.583333	2.666667	0.253188	1.410545	3.000000	-3.349819	8.683152	0.385542
Bifidobacterium stercoris	Adrenaline	0.100000	t_gr	12.666667	15.500000	2.833333	0.042461	3.400000	3.000000	0.181295	5.485372	0.409639
Bifidobacterium stercoris	Adrenaline	0.010000	t_gr	12.666667	14.916667	2.250000	0.080230	2.602095	3.000000	-0.501823	5.001823	0.325301
Bifidobacterium stercoris	Adrenaline	0.001000	t_gr	12.666667	14.583333	1.916667	0.127666	2.090909	3.000000	-1.000576	4.833909	0.277108
Bifidobacterium stercoris	Adrenaline	0.000100	t_gr	12.666667	16.666667	4.000000	0.104767	2.302306	3.000000	-1.529145	9.529145	0.578313
Bifidobacterium stercoris	Adrenaline	0.000010	t_gr	12.666667	16.250000	3.583333	0.143225	1.971592	3.000000	-2.200705	9.367372	0.518072
Bifidobacterium stercoris	Adrenaline	0.000001	t_gr	12.666667	15.833333	3.166667	0.130371	2.068969	3.000000	-1.704236	8.037570	0.457831
Bifidobacterium stercoris	Levodopa	0.100000	t_dr	34.083333	24.000000	-10.083333	0.296888	-1.259620	3.000000	-35.559013	15.392347	-0.742331
Bifidobacterium stercoris	Levodopa	0.010000	t_dr	34.083333	21.333333	-12.750000	0.243427	-1.448065	3.000000	-40.770977	15.270977	-0.938650
Bifidobacterium stercoris	Levodopa	0.001000	t_dr	34.083333	28.500000	-5.583333	0.489128	-0.786207	3.000000	-28.183827	17.017160	-0.411043
Bifidobacterium stercoris	Levodopa	0.000100	t_dr	34.083333	28.750000	-5.333333	0.515838	-0.734517	3.000000	-28.441098	17.774431	-0.392638
Bifidobacterium stercoris	Levodopa	0.000010	t_dr	34.083333	23.583333	-10.500000	0.261588	-1.379489	3.000000	-34.723243	13.723243	-0.773006
Bifidobacterium stercoris	Levodopa	0.000001	t_dr	34.083333	21.583333	-12.500000	0.228488	-1.508776	3.000000	-38.866119	13.866119	-0.920245
Bifidobacterium stercoris	Dopamine	0.100000	t_dr	39.666667	36.833333	-2.833333	0.809183	-0.263563	3.000000	-37.044974	31.378307	-0.208589
Bifidobacterium stercoris	Dopamine	0.010000	t_dr	39.666667	31.833333	-7.833333	0.311262	-1.215021	3.000000	-28.350813	12.684146	-0.576687
Bifidobacterium stercoris	Dopamine	0.001000	t_dr	39.666667	30.000000	-9.666667	0.308009	-1.224927	3.000000	-34.781346	15.448013	-0.711656
Bifidobacterium stercoris	Dopamine	0.000100	t_dr	39.666667	28.166667	-11.500000	0.180753	-1.737167	3.000000	-32.567716	9.567716	-0.846626

Species	Hormon	Concentration	Parameter	Mean_Baseline	Mean_Treatment	Diff	P_Value	T_Statistic	DF	Lower_CI	Upper_CI	Diff_Norm
Bifidobacterium stercoris	Dopamine	0.000010	t_dr	39.666667	28.416667	-11.250000	0.203947	-1.618693	3.000000	-33.368171	10.868171	-0.828221
Bifidobacterium stercoris	Dopamine	0.000001	t_dr	39.666667	31.250000	-8.416667	0.435650	-0.897373	3.000000	-38.265554	21.432221	-0.619632
Bifidobacterium stercoris	Noradrenaline	0.100000	t_dr	41.333333	29.916667	-11.416667	0.275029	-1.331940	3.000000	-38.694874	15.861541	-0.840491
Bifidobacterium stercoris	Noradrenaline	0.010000	t_dr	41.333333	29.000000	-12.333333	0.248176	-1.429613	3.000000	-39.788445	15.121778	-0.907975
Bifidobacterium stercoris	Noradrenaline	0.001000	t_dr	41.333333	35.583333	-5.750000	0.672524	-0.466689	3.000000	-44.960430	33.460430	-0.423313
Bifidobacterium stercoris	Noradrenaline	0.000100	t_dr	41.333333	34.250000	-7.083333	0.623870	-0.544652	3.000000	-48.471849	34.305182	-0.521472
Bifidobacterium stercoris	Noradrenaline	0.000010	t_dr	41.333333	36.000000	-5.333333	0.689746	-0.439941	3.000000	-43.913583	33.246916	-0.392638
Bifidobacterium stercoris	Noradrenaline	0.000001	t_dr	41.333333	27.750000	-13.583333	0.189839	-1.688809	3.000000	-39.180200	12.013533	-1.000000
Bifidobacterium stercoris	Adrenaline	0.100000	t_dr	40.083333	27.916667	-12.166667	0.199002	-1.642628	3.000000	-35.738501	11.405168	-0.895706
Bifidobacterium stercoris	Adrenaline	0.010000	t_dr	40.083333	27.000000	-13.083333	0.212874	-1.577087	3.000000	-39.484552	13.317885	-0.963190
Bifidobacterium stercoris	Adrenaline	0.001000	t_dr	40.083333	32.583333	-7.500000	0.347903	-1.110181	3.000000	-28.999513	13.999513	-0.552147
Bifidobacterium stercoris	Adrenaline	0.000100	t_dr	40.083333	26.750000	-13.333333	0.235616	-1.479285	3.000000	-42.017882	15.351215	-0.981595
Bifidobacterium stercoris	Adrenaline	0.000010	t_dr	40.083333	27.666667	-12.416667	0.212982	-1.576595	3.000000	-37.480419	12.647086	-0.914110
Bifidobacterium stercoris	Adrenaline	0.000001	t_dr	40.083333	34.833333	-5.250000	0.522021	-0.722866	3.000000	-28.363344	17.863344	-0.386503
Enterococcus faecium	Levodopa	0.100000	auc_lin	12.983149	11.550862	-1.432287	0.471247	-0.822148	3.000000	-6.976516	4.111942	-0.151454
Enterococcus faecium	Levodopa	0.010000	auc_lin	12.983149	12.879277	-0.103872	0.959582	-0.055019	3.000000	-6.112055	5.904312	-0.010984
Enterococcus faecium	Levodopa	0.001000	auc_lin	12.983149	11.808352	-1.174796	0.670784	-0.469414	3.000000	-9.139460	6.789867	-0.124226
Enterococcus faecium	Levodopa	0.000100	auc_lin	12.983149	12.656809	-0.326340	0.899480	-0.137314	3.000000	-7.889736	7.237055	-0.034508
Enterococcus faecium	Levodopa	0.000010	auc_lin	12.983149	11.688023	-1.295126	0.659530	-0.487147	3.000000	-9.755950	7.165698	-0.136950
Enterococcus faecium	Levodopa	0.000001	auc_lin	12.983149	10.203953	-2.779196	0.344674	-1.118966	3.000000	-10.683495	5.125104	-0.293879
Enterococcus faecium	Dopamine	0.100000	auc_lin	5.720329	13.512719	7.792390	0.000276	19.932610	3.000000	6.548255	9.036525	0.823987
Enterococcus faecium	Dopamine	0.010000	auc_lin	5.720329	13.919461	8.199132	0.000006	70.408589	3.000000	7.828534	8.569730	0.866997
Enterococcus faecium	Dopamine	0.001000	auc_lin	5.720329	15.177263	9.456934	0.004895	7.508470	3.000000	5.448636	13.465232	1.000000
Enterococcus faecium	Dopamine	0.000100	auc_lin	5.720329	14.582659	8.862331	0.001013	12.866636	3.000000	6.670313	11.054348	0.937125
Enterococcus faecium	Dopamine	0.000010	auc_lin	5.720329	13.829685	8.109357	0.001205	12.132791	3.000000	5.982262	10.236451	0.857504
Enterococcus faecium	Dopamine	0.000001	auc_lin	5.720329	12.510378	6.790050	0.019111	4.617870	3.000000	2.110626	11.469473	0.717997
Enterococcus faecium	Noradrenaline	0.100000	auc_lin	6.120023	13.770217	7.650194	0.000168	23.543251	3.000000	6.616083	8.684305	0.808951

Species	Hormon	Concentration	Parameter	Mean_Baseline	Mean_Treatment	Diff	P_Value	T_Statistic	DF	Lower_CI	Upper_CI	Diff_Norm
Enterococcus faecium	Noradrenaline	0.010000	auc_lin	6.120023	13.052789	6.932766	0.002394	9.605930	3.000000	4.635939	9.229593	0.733088
Enterococcus faecium	Noradrenaline	0.001000	auc_lin	6.120023	12.102593	5.982571	0.000387	17.790632	3.000000	4.912389	7.052753	0.632612
Enterococcus faecium	Noradrenaline	0.000100	auc_lin	6.120023	12.899580	6.779557	0.002178	9.921507	3.000000	4.604930	8.954184	0.716887
Enterococcus faecium	Noradrenaline	0.000010	auc_lin	6.120023	13.212833	7.092810	0.000029	42.337638	3.000000	6.559656	7.625965	0.750012
Enterococcus faecium	Noradrenaline	0.000001	auc_lin	6.120023	13.191508	7.071485	0.036642	3.604651	3.000000	0.828268	13.314703	0.747757
Enterococcus faecium	Adrenaline	0.100000	auc_lin	8.111651	13.090117	4.978465	0.029103	3.941735	3.000000	0.958992	8.997939	0.526435
Enterococcus faecium	Adrenaline	0.010000	auc_lin	8.111651	13.016473	4.904822	0.004324	7.838664	3.000000	2.913497	6.896148	0.518648
Enterococcus faecium	Adrenaline	0.001000	auc_lin	8.111651	13.717679	5.606028	0.017791	4.741533	3.000000	1.843346	9.368711	0.592796
Enterococcus faecium	Adrenaline	0.000100	auc_lin	8.111651	14.335512	6.223861	0.000055	34.091788	3.000000	5.642868	6.804855	0.658127
Enterococcus faecium	Adrenaline	0.000010	auc_lin	8.111651	13.122035	5.010384	0.007059	6.607175	3.000000	2.597056	7.423712	0.529811
Enterococcus faecium	Adrenaline	0.000001	auc_lin	8.111651	13.006904	4.895252	0.022342	4.356992	3.000000	1.319648	8.470856	0.517636
Enterococcus faecium	Levodopa	0.100000	k_lin	0.410456	0.336491	-0.073966	0.084361	-2.544315	3.000000	-0.166482	0.018551	-0.550460
Enterococcus faecium	Levodopa	0.010000	k_lin	0.410456	0.354907	-0.055550	0.112662	-2.223625	3.000000	-0.135052	0.023953	-0.413405
Enterococcus faecium	Levodopa	0.001000	k_lin	0.410456	0.313576	-0.096880	0.103327	-2.317423	3.000000	-0.229923	0.036163	-0.720994
Enterococcus faecium	Levodopa	0.000100	k_lin	0.410456	0.343480	-0.066977	0.173868	-1.775698	3.000000	-0.187014	0.053060	-0.498447
Enterococcus faecium	Levodopa	0.000010	k_lin	0.410456	0.326774	-0.083682	0.140018	-1.994886	3.000000	-0.217180	0.049816	-0.622770
Enterococcus faecium	Levodopa	0.000001	k_lin	0.410456	0.308863	-0.101594	0.083450	-2.556749	3.000000	-0.228050	0.024862	-0.756072
Enterococcus faecium	Dopamine	0.100000	k_lin	0.257440	0.376164	0.118724	0.007344	6.515842	3.000000	0.060737	0.176711	0.883558
Enterococcus faecium	Dopamine	0.010000	k_lin	0.257440	0.357588	0.100148	0.023420	4.280610	3.000000	0.025692	0.174604	0.745312
Enterococcus faecium	Dopamine	0.001000	k_lin	0.257440	0.391810	0.134371	0.079467	2.613174	3.000000	-0.029272	0.298014	1.000000
Enterococcus faecium	Dopamine	0.000100	k_lin	0.257440	0.383275	0.125835	0.023524	4.273468	3.000000	0.032126	0.219545	0.936479
Enterococcus faecium	Dopamine	0.000010	k_lin	0.257440	0.366070	0.108631	0.008443	6.202682	3.000000	0.052895	0.164367	0.808443
Enterococcus faecium	Dopamine	0.000001	k_lin	0.257440	0.353152	0.095712	0.032711	3.767977	3.000000	0.014873	0.176551	0.712300
Enterococcus faecium	Noradrenaline	0.100000	k_lin	0.240448	0.364268	0.123821	0.002475	9.496978	3.000000	0.082328	0.165313	0.921486
Enterococcus faecium	Noradrenaline	0.010000	k_lin	0.240448	0.326233	0.085786	0.040331	3.470531	3.000000	0.007121	0.164450	0.638425
Enterococcus faecium	Noradrenaline	0.001000	k_lin	0.240448	0.305552	0.065105	0.013396	5.258868	3.000000	0.025706	0.104503	0.484516
Enterococcus faecium	Noradrenaline	0.000100	k_lin	0.240448	0.325142	0.084694	0.023060	4.305594	3.000000	0.022093	0.147295	0.630301

Species	Hormon	Concentration	Parameter	Mean_Baseline	Mean_Treatment	Diff	P_Value	T_Statistic	DF	Lower_CI	Upper_CI	Diff_Norm
Enterococcus faecium	Noradrenaline	0.000010	k_lin	0.240448	0.338517	0.098070	0.002492	9.474653	3.000000	0.065129	0.131011	0.729845
Enterococcus faecium	Noradrenaline	0.000001	k_lin	0.240448	0.367419	0.126971	0.074816	2.683650	3.000000	-0.023600	0.277542	0.944934
Enterococcus faecium	Adrenaline	0.100000	k_lin	0.282617	0.344740	0.062123	0.245097	1.441531	3.000000	-0.075025	0.199272	0.462328
Enterococcus faecium	Adrenaline	0.010000	k_lin	0.282617	0.335104	0.052487	0.231855	1.494719	3.000000	-0.059265	0.164239	0.390614
Enterococcus faecium	Adrenaline	0.001000	k_lin	0.282617	0.349721	0.067104	0.270406	1.348010	3.000000	-0.091318	0.225526	0.499393
Enterococcus faecium	Adrenaline	0.000100	k_lin	0.282617	0.361324	0.078707	0.028864	3.954205	3.000000	0.015361	0.142052	0.585742
Enterococcus faecium	Adrenaline	0.000010	k_lin	0.282617	0.329142	0.046525	0.218294	1.552764	3.000000	-0.048830	0.141880	0.346245
Enterococcus faecium	Adrenaline	0.000001	k_lin	0.282617	0.349007	0.066390	0.166864	1.816735	3.000000	-0.049908	0.182687	0.494078
Enterococcus faecium	Levodopa	0.100000	death_lin	0.005355	0.005464	0.000108	0.901312	0.134791	3.000000	-0.002453	0.002670	0.006600
Enterococcus faecium	Levodopa	0.010000	death_lin	0.005355	0.002664	-0.002692	0.693717	-0.433829	3.000000	-0.022437	0.017054	-0.163739
Enterococcus faecium	Levodopa	0.001000	death_lin	0.005355	0.001721	-0.003634	0.541019	-0.687741	3.000000	-0.020450	0.013182	-0.221057
Enterococcus faecium	Levodopa	0.000100	death_lin	0.005355	0.001223	-0.004132	0.490080	-0.784324	3.000000	-0.020898	0.012634	-0.251357
Enterococcus faecium	Levodopa	0.000010	death_lin	0.005355	0.000280	-0.005075	0.363856	-1.067932	3.000000	-0.020199	0.010049	-0.308719
Enterococcus faecium	Levodopa	0.000001	death_lin	0.005355	0.000328	-0.005027	0.356535	-1.087090	3.000000	-0.019744	0.009690	-0.305802
Enterococcus faecium	Dopamine	0.100000	death_lin	0.008372	0.000631	-0.007741	0.429921	-0.909986	3.000000	-0.034814	0.019332	-0.470904
Enterococcus faecium	Dopamine	0.010000	death_lin	0.008372	0.009366	0.000994	0.945691	0.073969	3.000000	-0.041758	0.043746	0.060446
Enterococcus faecium	Dopamine	0.001000	death_lin	0.008372	0.018018	0.009646	0.212277	1.579807	3.000000	-0.009785	0.029076	0.586745
Enterococcus faecium	Dopamine	0.000100	death_lin	0.008372	0.001257	-0.007116	0.481119	-0.802164	3.000000	-0.035347	0.021115	-0.432862
Enterococcus faecium	Dopamine	0.000010	death_lin	0.008372	0.000110	-0.008262	0.398331	-0.982431	3.000000	-0.035026	0.018502	-0.502592
Enterococcus faecium	Dopamine	0.000001	death_lin	0.008372	0.004328	-0.004045	0.694341	-0.432871	3.000000	-0.033781	0.025692	-0.246040
Enterococcus faecium	Noradrenaline	0.100000	death_lin	0.018954	0.002515	-0.016439	0.450300	-0.865781	3.000000	-0.076866	0.043988	-1.000000
Enterococcus faecium	Noradrenaline	0.010000	death_lin	0.018954	0.012100	-0.006854	0.683899	-0.448977	3.000000	-0.055434	0.041727	-0.416912
Enterococcus faecium	Noradrenaline	0.001000	death_lin	0.018954	0.010332	-0.008622	0.622220	-0.547365	3.000000	-0.058752	0.041508	-0.524489
Enterococcus faecium	Noradrenaline	0.000100	death_lin	0.018954	0.002939	-0.016015	0.376858	-1.034809	3.000000	-0.065268	0.033238	-0.974214
Enterococcus faecium	Noradrenaline	0.000010	death_lin	0.018954	0.002885	-0.016069	0.389283	-1.004167	3.000000	-0.066995	0.034857	-0.977483
Enterococcus faecium	Noradrenaline	0.000001	death_lin	0.018954	0.003146	-0.015808	0.382641	-1.020429	3.000000	-0.065110	0.033494	-0.961632
Enterococcus faecium	Adrenaline	0.100000	death_lin	0.002647	0.006257	0.003610	0.558439	0.656370	3.000000	-0.013894	0.021114	0.219604

Species	Hormon	Concentration	Parameter	Mean_Baseline	Mean_Treatment	Diff	P_Value	T_Statistic	DF	Lower_CI	Upper_CI	Diff_Norm
Enterococcus faecium	Adrenaline	0.010000	death_lin	0.002647	0.002280	-0.000366	0.417373	-0.938155	3.000000	-0.001609	0.000876	-0.022287
Enterococcus faecium	Adrenaline	0.001000	death_lin	0.002647	0.006521	0.003874	0.303230	1.239675	3.000000	-0.006071	0.013818	0.235642
Enterococcus faecium	Adrenaline	0.000100	death_lin	0.002647	0.001711	-0.000936	0.209627	-1.591991	3.000000	-0.002808	0.000935	-0.056947
Enterococcus faecium	Adrenaline	0.000010	death_lin	0.002647	0.004877	0.002231	0.270121	1.349011	3.000000	-0.003031	0.007493	0.135684
Enterococcus faecium	Adrenaline	0.000001	death_lin	0.002647	0.003398	0.000751	0.468865	0.827025	3.000000	-0.002139	0.003641	0.045690
Enterococcus faecium	Levodopa	0.100000	gr	0.506376	0.468694	-0.037682	0.132477	-2.052256	3.000000	-0.096115	0.020752	-0.306930
Enterococcus faecium	Levodopa	0.010000	gr	0.506376	0.423737	-0.082639	0.199606	-1.639668	3.000000	-0.243034	0.077756	-0.673120
Enterococcus faecium	Levodopa	0.001000	gr	0.506376	0.454614	-0.051762	0.393589	-0.993765	3.000000	-0.217524	0.114001	-0.421615
Enterococcus faecium	Levodopa	0.000100	gr	0.506376	0.496452	-0.009924	0.860321	-0.191557	3.000000	-0.174793	0.154946	-0.080832
Enterococcus faecium	Levodopa	0.000010	gr	0.506376	0.466267	-0.040109	0.441243	-0.885202	3.000000	-0.184308	0.104090	-0.326701
Enterococcus faecium	Levodopa	0.000001	gr	0.506376	0.487914	-0.018462	0.540436	-0.688803	3.000000	-0.103759	0.066836	-0.150376
Enterococcus faecium	Dopamine	0.100000	gr	0.464583	0.512881	0.048298	0.058881	2.973957	3.000000	-0.003386	0.099982	0.393402
Enterococcus faecium	Dopamine	0.010000	gr	0.464583	0.527388	0.062805	0.082357	2.571902	3.000000	-0.014909	0.140519	0.511564
Enterococcus faecium	Dopamine	0.001000	gr	0.464583	0.568440	0.103857	0.012844	5.339419	3.000000	0.041955	0.165759	0.845948
Enterococcus faecium	Dopamine	0.000100	gr	0.464583	0.546733	0.082150	0.004147	7.952675	3.000000	0.049276	0.115024	0.669136
Enterococcus faecium	Dopamine	0.000010	gr	0.464583	0.587353	0.122770	0.101691	2.334923	3.000000	-0.044563	0.290103	1.000000
Enterococcus faecium	Dopamine	0.000001	gr	0.464583	0.519617	0.055033	0.080295	2.601172	3.000000	-0.012298	0.122365	0.448262
Enterococcus faecium	Noradrenaline	0.100000	gr	0.484969	0.514040	0.029071	0.011130	5.621720	3.000000	0.012614	0.045528	0.236792
Enterococcus faecium	Noradrenaline	0.010000	gr	0.484969	0.552268	0.067299	0.098002	2.375652	3.000000	-0.022855	0.157453	0.548171
Enterococcus faecium	Noradrenaline	0.001000	gr	0.484969	0.552903	0.067934	0.031661	3.815888	3.000000	0.011277	0.124590	0.553339
Enterococcus faecium	Noradrenaline	0.000100	gr	0.484969	0.547604	0.062634	0.082671	2.567522	3.000000	-0.015001	0.140269	0.510174
Enterococcus faecium	Noradrenaline	0.000010	gr	0.484969	0.538987	0.054017	0.013308	5.271302	3.000000	0.021405	0.086629	0.439986
Enterococcus faecium	Noradrenaline	0.000001	gr	0.484969	0.540160	0.055191	0.032073	3.796843	3.000000	0.008931	0.101451	0.449547
Enterococcus faecium	Adrenaline	0.100000	gr	0.489735	0.504606	0.014871	0.634814	0.526778	3.000000	-0.074970	0.104713	0.121130
Enterococcus faecium	Adrenaline	0.010000	gr	0.489735	0.536064	0.046329	0.261201	1.380894	3.000000	-0.060442	0.153100	0.377363
Enterococcus faecium	Adrenaline	0.001000	gr	0.489735	0.568217	0.078482	0.019267	4.604002	3.000000	0.024233	0.132732	0.639263
Enterococcus faecium	Adrenaline	0.000100	gr	0.489735	0.564494	0.074759	0.018788	4.647099	3.000000	0.023562	0.125955	0.608933

Species	Hormon	Concentration	Parameter	Mean_Baseline	Mean_Treatment	Diff	P_Value	T_Statistic	DF	Lower_CI	Upper_CI	Diff_Norm
Enterococcus faecium	Adrenaline	0.000010	gr	0.489735	0.544810	0.055075	0.022871	4.318971	3.000000	0.014493	0.095656	0.448599
Enterococcus faecium	Adrenaline	0.000001	gr	0.489735	0.530191	0.040456	0.094942	2.410883	3.000000	-0.012947	0.093860	0.329527
Enterococcus faecium	Levodopa	0.100000	dr	-0.012811	-0.002978	0.009833	0.530100	0.707807	3.000000	-0.034379	0.054046	0.329094
Enterococcus faecium	Levodopa	0.010000	dr	-0.012811	0.017069	0.029880	0.353677	1.094674	3.000000	-0.056987	0.116747	1.000000
Enterococcus faecium	Levodopa	0.001000	dr	-0.012811	-0.008151	0.004660	0.618081	0.554192	3.000000	-0.022098	0.031418	0.155947
Enterococcus faecium	Levodopa	0.000100	dr	-0.012811	-0.001342	0.011469	0.209533	1.592426	3.000000	-0.011451	0.034388	0.383823
Enterococcus faecium	Levodopa	0.000010	dr	-0.012811	-0.004284	0.008527	0.208448	1.597465	3.000000	-0.008460	0.025513	0.285365
Enterococcus faecium	Levodopa	0.000001	dr	-0.012811	-0.007123	0.005688	0.554031	0.664236	3.000000	-0.021563	0.032938	0.190353
Enterococcus faecium	Dopamine	0.100000	dr	-0.006386	-0.008344	-0.001958	0.952293	-0.064959	3.000000	-0.097902	0.093985	-0.065542
Enterococcus faecium	Dopamine	0.010000	dr	-0.006386	-0.009352	-0.002966	0.927038	-0.099472	3.000000	-0.097871	0.091939	-0.099277
Enterococcus faecium	Dopamine	0.001000	dr	-0.006386	-0.035642	-0.029256	0.090588	-2.463466	3.000000	-0.067051	0.008539	-0.979123
Enterococcus faecium	Dopamine	0.000100	dr	-0.006386	-0.002131	0.004255	0.891949	0.147699	3.000000	-0.087422	0.095931	0.142395
Enterococcus faecium	Dopamine	0.000010	dr	-0.006386	0.004819	0.011205	0.720118	0.393696	3.000000	-0.079373	0.101784	0.375013
Enterococcus faecium	Dopamine	0.000001	dr	-0.006386	0.000908	0.007294	0.782815	0.301369	3.000000	-0.069727	0.084314	0.244098
Enterococcus faecium	Noradrenaline	0.100000	dr	-0.024023	-0.005825	0.018199	0.669827	0.470914	3.000000	-0.104788	0.141185	0.609062
Enterococcus faecium	Noradrenaline	0.010000	dr	-0.024023	-0.018479	0.005545	0.886915	0.154651	3.000000	-0.108552	0.119641	0.185560
Enterococcus faecium	Noradrenaline	0.001000	dr	-0.024023	-0.026280	-0.002256	0.949552	-0.068699	3.000000	-0.106786	0.102273	-0.075518
Enterococcus faecium	Noradrenaline	0.000100	dr	-0.024023	-0.006240	0.017783	0.642603	0.514184	3.000000	-0.092284	0.127851	0.595167
Enterococcus faecium	Noradrenaline	0.000010	dr	-0.024023	-0.007888	0.016136	0.702850	0.419851	3.000000	-0.106173	0.138445	0.540026
Enterococcus faecium	Noradrenaline	0.000001	dr	-0.024023	-0.007049	0.016974	0.692467	0.435751	3.000000	-0.106995	0.140943	0.568085
Enterococcus faecium	Adrenaline	0.100000	dr	-0.012307	-0.009354	0.002953	0.874229	0.172218	3.000000	-0.051616	0.057522	0.098829
Enterococcus faecium	Adrenaline	0.010000	dr	-0.012307	-0.008182	0.004125	0.727486	0.382642	3.000000	-0.030183	0.038433	0.138054
Enterococcus faecium	Adrenaline	0.001000	dr	-0.012307	-0.006736	0.005571	0.692076	0.436353	3.000000	-0.035060	0.046202	0.186448
Enterococcus faecium	Adrenaline	0.000100	dr	-0.012307	-0.002150	0.010157	0.533462	0.701593	3.000000	-0.035915	0.056228	0.339920
Enterococcus faecium	Adrenaline	0.000010	dr	-0.012307	-0.006984	0.005323	0.729227	0.380037	3.000000	-0.039254	0.049901	0.178156
Enterococcus faecium	Adrenaline	0.000001	dr	-0.012307	-0.003380	0.008927	0.515451	0.735251	3.000000	-0.029711	0.047565	0.298754
Enterococcus faecium	Levodopa	0.100000	td	1.369980	1.482462	0.112482	0.139064	2.001941	3.000000	-0.066329	0.291293	0.325618

Species	Hormon	Concentration	Parameter	Mean_Baseline	Mean_Treatment	Diff	P_Value	T_Statistic	DF	Lower_CI	Upper_CI	Diff_Norm
Enterococcus faecium	Levodopa	0.010000	td	1.369980	1.715422	0.345442	0.240758	1.458607	3.000000	-0.408257	1.099141	1.000000
Enterococcus faecium	Levodopa	0.001000	td	1.369980	1.596556	0.226576	0.380899	1.024737	3.000000	-0.477084	0.930237	0.655903
Enterococcus faecium	Levodopa	0.000100	td	1.369980	1.444225	0.074246	0.698180	0.426986	3.000000	-0.479129	0.627621	0.214930
Enterococcus faecium	Levodopa	0.000010	td	1.369980	1.533222	0.163243	0.424481	0.922106	3.000000	-0.400154	0.726639	0.472562
Enterococcus faecium	Levodopa	0.000001	td	1.369980	1.428500	0.058521	0.502729	0.759602	3.000000	-0.186659	0.303700	0.169408
Enterococcus faecium	Dopamine	0.100000	td	1.492827	1.353128	-0.139700	0.056823	-3.018588	3.000000	-0.286983	0.007583	-0.404408
Enterococcus faecium	Dopamine	0.010000	td	1.492827	1.321781	-0.171046	0.087358	-2.504516	3.000000	-0.388392	0.046299	-0.495152
Enterococcus faecium	Dopamine	0.001000	td	1.492827	1.221378	-0.271449	0.011418	-5.570403	3.000000	-0.426531	-0.116366	-0.785801
Enterococcus faecium	Dopamine	0.000100	td	1.492827	1.268287	-0.224540	0.004280	-7.866288	3.000000	-0.315382	-0.133698	-0.650008
Enterococcus faecium	Dopamine	0.000010	td	1.492827	1.202041	-0.290786	0.064484	-2.861750	3.000000	-0.614159	0.032586	-0.841780
Enterococcus faecium	Dopamine	0.000001	td	1.492827	1.338793	-0.154034	0.083666	-2.553790	3.000000	-0.345987	0.037918	-0.445905
Enterococcus faecium	Noradrenaline	0.100000	td	1.429920	1.348757	-0.081163	0.012157	-5.446222	3.000000	-0.128589	-0.033736	-0.234953
Enterococcus faecium	Noradrenaline	0.010000	td	1.429920	1.264497	-0.165423	0.069653	-2.768549	3.000000	-0.355576	0.024731	-0.478872
Enterococcus faecium	Noradrenaline	0.001000	td	1.429920	1.258539	-0.171380	0.021314	-4.434425	3.000000	-0.294375	-0.048386	-0.496119
Enterococcus faecium	Noradrenaline	0.000100	td	1.429920	1.273810	-0.156110	0.075129	-2.678744	3.000000	-0.341574	0.029354	-0.451913
Enterococcus faecium	Noradrenaline	0.000010	td	1.429920	1.288038	-0.141882	0.010610	-5.718729	3.000000	-0.220839	-0.062925	-0.410727
Enterococcus faecium	Noradrenaline	0.000001	td	1.429920	1.286387	-0.143533	0.023646	-4.265167	3.000000	-0.250630	-0.036436	-0.415505
Enterococcus faecium	Adrenaline	0.100000	td	1.420422	1.379404	-0.041018	0.655194	-0.494030	3.000000	-0.305247	0.223211	-0.118740
Enterococcus faecium	Adrenaline	0.010000	td	1.420422	1.304363	-0.116059	0.290830	-1.279090	3.000000	-0.404819	0.172702	-0.335972
Enterococcus faecium	Adrenaline	0.001000	td	1.420422	1.223093	-0.197328	0.022267	-4.362519	3.000000	-0.341279	-0.053378	-0.571234
Enterococcus faecium	Adrenaline	0.000100	td	1.420422	1.231079	-0.189342	0.018899	-4.636970	3.000000	-0.319292	-0.059393	-0.548116
Enterococcus faecium	Adrenaline	0.000010	td	1.420422	1.273584	-0.146837	0.031091	-3.842782	3.000000	-0.268442	-0.025232	-0.425071
Enterococcus faecium	Adrenaline	0.000001	td	1.420422	1.307881	-0.112541	0.112211	-2.227942	3.000000	-0.273297	0.048215	-0.325789
Enterococcus faecium	Levodopa	0.100000	lagC	4.324102	4.629289	0.305187	0.145365	1.956383	3.000000	-0.191260	0.801634	0.480534
Enterococcus faecium	Levodopa	0.010000	lagC	4.324102	4.117202	-0.206900	0.118110	-2.173116	3.000000	-0.509897	0.096097	-0.325776
Enterococcus faecium	Levodopa	0.001000	lagC	4.324102	4.076012	-0.248090	0.214529	-1.569587	3.000000	-0.751108	0.254929	-0.390631
Enterococcus faecium	Levodopa	0.000100	lagC	4.324102	4.273776	-0.050325	0.708476	-0.411290	3.000000	-0.439729	0.339078	-0.079240

Species	Hormon	Concentration	Parameter	Mean_Baseline	Mean_Treatment	Diff	P_Value	T_Statistic	DF	Lower_CI	Upper_CI	Diff_Norm
Enterococcus faecium	Levodopa	0.000010	lagC	4.324102	4.210233	-0.113869	0.532947	-0.702544	3.000000	-0.629685	0.401946	-0.179293
Enterococcus faecium	Levodopa	0.000001	lagC	4.324102	4.288395	-0.035707	0.864204	-0.186149	3.000000	-0.646155	0.574742	-0.056222
Enterococcus faecium	Dopamine	0.100000	lagC	4.607346	4.567456	-0.039890	0.801883	-0.273978	3.000000	-0.503234	0.423455	-0.062808
Enterococcus faecium	Dopamine	0.010000	lagC	4.607346	4.064076	-0.543269	0.060661	-2.936907	3.000000	-1.131959	0.045420	-0.855408
Enterococcus faecium	Dopamine	0.001000	lagC	4.607346	3.972246	-0.635100	0.003268	-8.634109	3.000000	-0.869191	-0.401008	-1.000000
Enterococcus faecium	Dopamine	0.000100	lagC	4.607346	4.180227	-0.427118	0.057187	-3.010533	3.000000	-0.878627	0.024390	-0.672522
Enterococcus faecium	Dopamine	0.000010	lagC	4.607346	4.237292	-0.370054	0.159141	-1.864356	3.000000	-1.001734	0.261626	-0.582670
Enterococcus faecium	Dopamine	0.000001	lagC	4.607346	4.223382	-0.383964	0.128264	-2.086011	3.000000	-0.969743	0.201816	-0.604572
Enterococcus faecium	Noradrenaline	0.100000	lagC	4.860145	4.668246	-0.191899	0.407289	-0.961362	3.000000	-0.827153	0.443355	-0.302156
Enterococcus faecium	Noradrenaline	0.010000	lagC	4.860145	4.347195	-0.512951	0.093018	-2.433752	3.000000	-1.183700	0.157799	-0.807669
Enterococcus faecium	Noradrenaline	0.001000	lagC	4.860145	4.367494	-0.492651	0.097336	-2.383202	3.000000	-1.150521	0.165218	-0.775707
Enterococcus faecium	Noradrenaline	0.000100	lagC	4.860145	4.399011	-0.461135	0.098353	-2.371694	3.000000	-1.079906	0.157637	-0.726082
Enterococcus faecium	Noradrenaline	0.000010	lagC	4.860145	4.445355	-0.414791	0.107932	-2.269935	3.000000	-0.996327	0.166745	-0.653111
Enterococcus faecium	Noradrenaline	0.000001	lagC	4.860145	4.506632	-0.353514	0.136659	-2.019976	3.000000	-0.910470	0.203443	-0.556627
Enterococcus faecium	Adrenaline	0.100000	lagC	4.810015	4.650697	-0.159319	0.566029	-0.642936	3.000000	-0.947923	0.629286	-0.250856
Enterococcus faecium	Adrenaline	0.010000	lagC	4.810015	4.445424	-0.364592	0.205836	-1.609721	3.000000	-1.085395	0.356212	-0.574070
Enterococcus faecium	Adrenaline	0.001000	lagC	4.810015	4.412089	-0.397927	0.143885	-1.966867	3.000000	-1.041783	0.245930	-0.626558
Enterococcus faecium	Adrenaline	0.000100	lagC	4.810015	4.480885	-0.329130	0.229951	-1.502641	3.000000	-1.026196	0.367935	-0.518234
Enterococcus faecium	Adrenaline	0.000010	lagC	4.810015	4.501757	-0.308259	0.259994	-1.385297	3.000000	-1.016422	0.399905	-0.485370
Enterococcus faecium	Adrenaline	0.000001	lagC	4.810015	4.542706	-0.267309	0.171031	-1.792084	3.000000	-0.742006	0.207388	-0.420893
Enterococcus faecium	Levodopa	0.100000	t_k	45.916667	46.000000	0.083333	0.637618	0.522233	3.000000	-0.424493	0.591160	0.003145
Enterococcus faecium	Levodopa	0.010000	t_k	45.916667	44.250000	-1.666667	0.752001	-0.346272	3.000000	-16.984342	13.651009	-0.062893
Enterococcus faecium	Levodopa	0.001000	t_k	45.916667	39.083333	-6.833333	0.526432	-0.714621	3.000000	-37.264441	23.597774	-0.257862
Enterococcus faecium	Levodopa	0.000100	t_k	45.916667	44.583333	-1.333333	0.786660	-0.295823	3.000000	-15.677242	13.010576	-0.050314
Enterococcus faecium	Levodopa	0.000010	t_k	45.916667	47.416667	1.500000	0.525286	0.716758	3.000000	-5.160085	8.160085	0.056604
Enterococcus faecium	Levodopa	0.000001	t_k	45.916667	47.833333	1.916667	0.392042	0.997489	3.000000	-4.198377	8.031710	0.072327
Enterococcus faecium	Dopamine	0.100000	t_k	37.666667	47.750000	10.083333	0.400027	0.978408	3.000000	-22.714497	42.881164	0.380503

Species	Hormon	Concentration	Parameter	Mean_Baseline	Mean_Treatment	Diff	P_Value	T_Statistic	DF	Lower_CI	Upper_CI	Diff_Norm
Enterococcus faecium	Dopamine	0.010000	t_k	37.666667	40.250000	2.583333	0.865474	0.184381	3.000000	-42.005368	47.172034	0.097484
Enterococcus faecium	Dopamine	0.001000	t_k	37.666667	31.750000	-5.916667	0.375454	-1.038333	3.000000	-24.051001	12.217667	-0.223270
Enterococcus faecium	Dopamine	0.000100	t_k	37.666667	42.750000	5.083333	0.723048	0.389294	3.000000	-36.472512	46.639179	0.191824
Enterococcus faecium	Dopamine	0.000010	t_k	37.666667	47.916667	10.250000	0.395472	0.989248	3.000000	-22.724628	43.224628	0.386792
Enterococcus faecium	Dopamine	0.000001	t_k	37.666667	40.416667	2.750000	0.858554	0.194020	3.000000	-42.357271	47.857271	0.103774
Enterococcus faecium	Noradrenaline	0.100000	t_k	37.583333	44.583333	7.000000	0.587043	0.606428	3.000000	-29.734963	43.734963	0.264151
Enterococcus faecium	Noradrenaline	0.010000	t_k	37.583333	12.583333	-25.000000	0.079695	-2.609847	3.000000	-55.484988	5.484988	-0.943396
Enterococcus faecium	Noradrenaline	0.001000	t_k	37.583333	11.083333	-26.500000	0.057882	-2.995383	3.000000	-54.654942	1.654942	-1.000000
Enterococcus faecium	Noradrenaline	0.000100	t_k	37.583333	39.000000	1.416667	0.391002	1.000000	3.000000	-3.091799	5.925132	0.053459
Enterococcus faecium	Noradrenaline	0.000010	t_k	37.583333	42.416667	4.833333	0.478244	0.807949	3.000000	-14.204782	23.871449	0.182390
Enterococcus faecium	Noradrenaline	0.000001	t_k	37.583333	43.083333	5.500000	0.399555	0.979526	3.000000	-12.369309	23.369309	0.207547
Enterococcus faecium	Adrenaline	0.100000	t_k	46.333333	41.583333	-4.750000	0.425978	-0.918758	3.000000	-21.203329	11.703329	-0.179245
Enterococcus faecium	Adrenaline	0.010000	t_k	46.333333	40.666667	-5.666667	0.213328	-1.575020	3.000000	-17.116596	5.783262	-0.213836
Enterococcus faecium	Adrenaline	0.001000	t_k	46.333333	36.166667	-10.166667	0.258429	-1.391042	3.000000	-33.426119	13.092785	-0.383648
Enterococcus faecium	Adrenaline	0.000100	t_k	46.333333	40.750000	-5.583333	0.217083	-1.558140	3.000000	-16.987099	5.820433	-0.210692
Enterococcus faecium	Adrenaline	0.000010	t_k	46.333333	37.666667	-8.666667	0.187406	-1.701492	3.000000	-24.876676	7.543343	-0.327044
Enterococcus faecium	Adrenaline	0.000001	t_k	46.333333	41.833333	-4.500000	0.349349	-1.106272	3.000000	-17.445281	8.445281	-0.169811
Enterococcus faecium	Levodopa	0.100000	t_gr	5.583333	5.750000	0.166667	0.495025	0.774597	3.000000	-0.518087	0.851420	0.285714
Enterococcus faecium	Levodopa	0.010000	t_gr	5.583333	5.583333	-0.000000	1.000000	-0.000000	3.000000	-0.866152	0.866152	-0.000000
Enterococcus faecium	Levodopa	0.001000	t_gr	5.583333	5.416667	-0.166667	0.495025	-0.774597	3.000000	-0.851420	0.518087	-0.285714
Enterococcus faecium	Levodopa	0.000100	t_gr	5.583333	5.500000	-0.083333	0.391002	-1.000000	3.000000	-0.348537	0.181871	-0.142857
Enterococcus faecium	Levodopa	0.000010	t_gr	5.583333	5.500000	-0.083333	0.717686	-0.397360	3.000000	-0.750748	0.584082	-0.142857
Enterococcus faecium	Levodopa	0.000001	t_gr	5.583333	5.583333	-0.000000	1.000000	-0.000000	3.000000	-0.866152	0.866152	-0.000000
Enterococcus faecium	Dopamine	0.100000	t_gr	5.833333	5.666667	-0.166667	0.495025	-0.774597	3.000000	-0.851420	0.518087	-0.285714
Enterococcus faecium	Dopamine	0.010000	t_gr	5.833333	5.333333	-0.500000	0.102728	-2.323790	3.000000	-1.184753	0.184753	-0.857143
Enterococcus faecium	Dopamine	0.001000	t_gr	5.833333	5.250000	-0.583333	0.035353	-3.655631	3.000000	-1.091160	-0.075507	-1.000000
Enterococcus faecium	Dopamine	0.000100	t_gr	5.833333	5.500000	-0.333333	0.181690	-1.732051	3.000000	-0.945795	0.279129	-0.571429

Species	Hormon	Concentration	Parameter	Mean_Baseline	Mean_Treatment	Diff	P_Value	T_Statistic	DF	Lower_CI	Upper_CI	Diff_Norm
Enterococcus faecium	Dopamine	0.000010	t_gr	5.833333	5.416667	-0.416667	0.194171	-1.666667	3.000000	-1.212278	0.378945	-0.714286
Enterococcus faecium	Dopamine	0.000001	t_gr	5.833333	5.416667	-0.416667	0.194171	-1.666667	3.000000	-1.212278	0.378945	-0.714286
Enterococcus faecium	Noradrenaline	0.100000	t_gr	6.083333	5.916667	-0.166667	0.495025	-0.774597	3.000000	-0.851420	0.518087	-0.285714
Enterococcus faecium	Noradrenaline	0.010000	t_gr	6.083333	5.500000	-0.583333	0.101838	-2.333333	3.000000	-1.378945	0.212278	-1.000000
Enterococcus faecium	Noradrenaline	0.001000	t_gr	6.083333	5.666667	-0.416667	0.194171	-1.666667	3.000000	-1.212278	0.378945	-0.714286
Enterococcus faecium	Noradrenaline	0.000100	t_gr	6.083333	5.666667	-0.416667	0.141122	-1.986799	3.000000	-1.084082	0.250748	-0.714286
Enterococcus faecium	Noradrenaline	0.000010	t_gr	6.083333	5.750000	-0.333333	0.252215	-1.414214	3.000000	-1.083443	0.416776	-0.571429
Enterococcus faecium	Noradrenaline	0.000001	t_gr	6.083333	5.750000	-0.333333	0.252215	-1.414214	3.000000	-1.083443	0.416776	-0.571429
Enterococcus faecium	Adrenaline	0.100000	t_gr	6.083333	5.750000	-0.333333	0.252215	-1.414214	3.000000	-1.083443	0.416776	-0.571429
Enterococcus faecium	Adrenaline	0.010000	t_gr	6.083333	5.666667	-0.416667	0.194171	-1.666667	3.000000	-1.212278	0.378945	-0.714286
Enterococcus faecium	Adrenaline	0.001000	t_gr	6.083333	5.666667	-0.416667	0.194171	-1.666667	3.000000	-1.212278	0.378945	-0.714286
Enterococcus faecium	Adrenaline	0.000100	t_gr	6.083333	5.750000	-0.333333	0.252215	-1.414214	3.000000	-1.083443	0.416776	-0.571429
Enterococcus faecium	Adrenaline	0.000010	t_gr	6.083333	5.750000	-0.333333	0.252215	-1.414214	3.000000	-1.083443	0.416776	-0.571429
Enterococcus faecium	Adrenaline	0.000001	t_gr	6.083333	5.750000	-0.333333	0.181690	-1.732051	3.000000	-0.945795	0.279129	-0.571429
Enterococcus faecium	Levodopa	0.100000	t_dr	47.166667	46.416667	-0.750000	0.391002	-1.000000	3.000000	-3.136835	1.636835	-0.031034
Enterococcus faecium	Levodopa	0.010000	t_dr	47.166667	45.666667	-1.500000	0.620556	-0.550105	3.000000	-10.177747	7.177747	-0.062069
Enterococcus faecium	Levodopa	0.001000	t_dr	47.166667	39.500000	-7.666667	0.448347	-0.869940	3.000000	-35.713150	20.379817	-0.317241
Enterococcus faecium	Levodopa	0.000100	t_dr	47.166667	48.000000	0.833333	0.391002	1.000000	3.000000	-1.818705	3.485372	0.034483
Enterococcus faecium	Levodopa	0.000010	t_dr	47.166667	48.000000	0.833333	0.391002	1.000000	3.000000	-1.818705	3.485372	0.034483
Enterococcus faecium	Levodopa	0.000001	t_dr	47.166667	48.000000	0.833333	0.391002	1.000000	3.000000	-1.818705	3.485372	0.034483
Enterococcus faecium	Dopamine	0.100000	t_dr	38.500000	48.000000	9.500000	0.391002	1.000000	3.000000	-20.733240	39.733240	0.393103
Enterococcus faecium	Dopamine	0.010000	t_dr	38.500000	48.000000	9.500000	0.391002	1.000000	3.000000	-20.733240	39.733240	0.393103
Enterococcus faecium	Dopamine	0.001000	t_dr	38.500000	33.750000	-4.750000	0.321506	-1.184505	3.000000	-17.511974	8.011974	-0.196552
Enterococcus faecium	Dopamine	0.000100	t_dr	38.500000	43.333333	4.833333	0.711833	0.406201	3.000000	-33.034212	42.700878	0.200000
Enterococcus faecium	Dopamine	0.000010	t_dr	38.500000	48.000000	9.500000	0.391002	1.000000	3.000000	-20.733240	39.733240	0.393103
Enterococcus faecium	Dopamine	0.000001	t_dr	38.500000	43.583333	5.083333	0.687706	0.443089	3.000000	-31.427254	41.593921	0.210345
Enterococcus faecium	Noradrenaline	0.100000	t_dr	37.916667	47.666667	9.750000	0.409621	0.955949	3.000000	-22.708684	42.208684	0.403448

Species	Hormon	Concentration	Parameter	Mean_Baseline	Mean_Treatment	Diff	P_Value	T_Statistic	DF	Lower_CI	Upper_CI	Diff_Norm
Enterococcus faecium	Noradrenaline	0.010000	t_dr	37.916667	22.833333	-15.083333	0.247194	-1.433396	3.000000	-48.571556	18.404889	-0.624138
Enterococcus faecium	Noradrenaline	0.001000	t_dr	37.916667	13.750000	-24.166667	0.059367	-2.963713	3.000000	-50.116924	1.783591	-1.000000
Enterococcus faecium	Noradrenaline	0.000100	t_dr	37.916667	39.500000	1.583333	0.391002	1.000000	3.000000	-3.455540	6.622207	0.065517
Enterococcus faecium	Noradrenaline	0.000010	t_dr	37.916667	48.000000	10.083333	0.391002	1.000000	3.000000	-22.006334	42.173000	0.417241
Enterococcus faecium	Noradrenaline	0.000001	t_dr	37.916667	46.333333	8.416667	0.391002	1.000000	3.000000	-18.368923	35.202256	0.348276
Enterococcus faecium	Adrenaline	0.100000	t_dr	48.000000	48.000000	0.000000	NA	NA	3.000000	NA	NA	0.000000
Enterococcus faecium	Adrenaline	0.010000	t_dr	48.000000	48.000000	0.000000	NA	NA	3.000000	NA	NA	0.000000
Enterococcus faecium	Adrenaline	0.001000	t_dr	48.000000	40.416667	-7.583333	0.391002	-1.000000	3.000000	-31.716884	16.550218	-0.313793
Enterococcus faecium	Adrenaline	0.000100	t_dr	48.000000	46.416667	-1.583333	0.391002	-1.000000	3.000000	-6.622207	3.455540	-0.065517
Enterococcus faecium	Adrenaline	0.000010	t_dr	48.000000	48.000000	0.000000	NA	NA	3.000000	NA	NA	0.000000
Enterococcus faecium	Adrenaline	0.000001	t_dr	48.000000	48.000000	0.000000	NA	NA	3.000000	NA	NA	0.000000
Enterococcus mundtii	Levodopa	0.100000	auc_lin	10.036276	12.975667	2.939391	0.053718	3.089904	3.000000	-0.088035	5.966818	0.338943
Enterococcus mundtii	Levodopa	0.010000	auc_lin	10.036276	12.139419	2.103143	0.333172	1.150933	3.000000	-3.712262	7.918548	0.242515
Enterococcus mundtii	Levodopa	0.001000	auc_lin	10.036276	11.851368	1.815092	0.457526	0.850533	3.000000	-4.976452	8.606636	0.209299
Enterococcus mundtii	Levodopa	0.000100	auc_lin	10.036276	10.887599	0.851323	0.724423	0.387230	3.000000	-6.145272	7.847918	0.098167
Enterococcus mundtii	Levodopa	0.000010	auc_lin	10.036276	11.437431	1.401155	0.538111	0.693052	3.000000	-5.032848	7.835159	0.161568
Enterococcus mundtii	Levodopa	0.000001	auc_lin	10.036276	11.057487	1.021211	0.557399	0.658222	3.000000	-3.916253	5.958674	0.117756
Enterococcus mundtii	Dopamine	0.100000	auc_lin	6.472466	14.437880	7.965415	0.000611	15.257183	3.000000	6.303935	9.626895	0.918497
Enterococcus mundtii	Dopamine	0.010000	auc_lin	6.472466	14.661774	8.189308	0.001759	10.671616	3.000000	5.747126	10.631491	0.944314
Enterococcus mundtii	Dopamine	0.001000	auc_lin	6.472466	14.867220	8.394754	0.003064	8.827036	3.000000	5.368160	11.421348	0.968004
Enterococcus mundtii	Dopamine	0.000100	auc_lin	6.472466	14.834693	8.362227	0.000797	13.954351	3.000000	6.455128	10.269327	0.964253
Enterococcus mundtii	Dopamine	0.000010	auc_lin	6.472466	15.144697	8.672232	0.003848	8.160902	3.000000	5.290386	12.054077	1.000000
Enterococcus mundtii	Dopamine	0.000001	auc_lin	6.472466	13.985004	7.512538	0.003361	8.550987	3.000000	4.716575	10.308502	0.866275
Enterococcus mundtii	Noradrenaline	0.100000	auc_lin	7.496643	14.081136	6.584492	0.010669	5.707482	3.000000	2.913032	10.255953	0.759262
Enterococcus mundtii	Noradrenaline	0.010000	auc_lin	7.496643	13.981085	6.484441	0.018050	4.716330	3.000000	2.108923	10.859959	0.747725
Enterococcus mundtii	Noradrenaline	0.001000	auc_lin	7.496643	13.630734	6.134090	0.009679	5.909117	3.000000	2.830481	9.437699	0.707325
Enterococcus mundtii	Noradrenaline	0.000100	auc_lin	7.496643	13.993802	6.497158	0.005581	7.173453	3.000000	3.614745	9.379572	0.749191

Species	Hormon	Concentration	Parameter	Mean_Baseline	Mean_Treatment	Diff	P_Value	T_Statistic	DF	Lower_CI	Upper_CI	Diff_Norm
Enterococcus mundtii	Noradrenaline	0.000010	auc_lin	7.496643	13.377339	5.880696	0.013217	5.284468	3.000000	2.339185	9.422206	0.678106
Enterococcus mundtii	Noradrenaline	0.000001	auc_lin	7.496643	12.350760	4.854116	0.008048	6.308704	3.000000	2.405441	7.302791	0.559731
Enterococcus mundtii	Adrenaline	0.100000	auc_lin	8.208247	14.711942	6.503695	0.017151	4.805842	3.000000	2.196924	10.810465	0.749945
Enterococcus mundtii	Adrenaline	0.010000	auc_lin	8.208247	14.382236	6.173989	0.023876	4.249667	3.000000	1.550477	10.797502	0.711926
Enterococcus mundtii	Adrenaline	0.001000	auc_lin	8.208247	13.770847	5.562600	0.012410	5.406055	3.000000	2.287999	8.837202	0.641427
Enterococcus mundtii	Adrenaline	0.000100	auc_lin	8.208247	13.830025	5.621777	0.000821	13.814671	3.000000	4.326705	6.916850	0.648250
Enterococcus mundtii	Adrenaline	0.000010	auc_lin	8.208247	13.714323	5.506075	0.007558	6.450165	3.000000	2.789434	8.222717	0.634909
Enterococcus mundtii	Adrenaline	0.000001	auc_lin	8.208247	13.296549	5.088301	0.005286	7.310381	3.000000	2.873198	7.303404	0.586735
Enterococcus mundtii	Levodopa	0.100000	k_lin	0.315750	0.351343	0.035593	0.113444	2.216203	3.000000	-0.015518	0.086704	0.283217
Enterococcus mundtii	Levodopa	0.010000	k_lin	0.315750	0.317451	0.001701	0.969439	0.041590	3.000000	-0.128460	0.131862	0.013535
Enterococcus mundtii	Levodopa	0.001000	k_lin	0.315750	0.299268	-0.016482	0.690639	-0.438565	3.000000	-0.136084	0.103120	-0.131148
Enterococcus mundtii	Levodopa	0.000100	k_lin	0.315750	0.275476	-0.040274	0.396268	-0.987346	3.000000	-0.170086	0.089539	-0.320463
Enterococcus mundtii	Levodopa	0.000010	k_lin	0.315750	0.296071	-0.019679	0.622929	-0.546198	3.000000	-0.134338	0.094981	-0.156586
Enterococcus mundtii	Levodopa	0.000001	k_lin	0.315750	0.305799	-0.009951	0.785199	-0.297930	3.000000	-0.116247	0.096345	-0.079181
Enterococcus mundtii	Dopamine	0.100000	k_lin	0.277692	0.403367	0.125674	0.009885	5.865031	3.000000	0.057482	0.193867	1.000000
Enterococcus mundtii	Dopamine	0.010000	k_lin	0.277692	0.383881	0.106189	0.027819	4.010389	3.000000	0.021923	0.190455	0.844951
Enterococcus mundtii	Dopamine	0.001000	k_lin	0.277692	0.385165	0.107473	0.014441	5.117566	3.000000	0.040639	0.174307	0.855170
Enterococcus mundtii	Dopamine	0.000100	k_lin	0.277692	0.391128	0.113436	0.003538	8.400837	3.000000	0.070463	0.156408	0.902615
Enterococcus mundtii	Dopamine	0.000010	k_lin	0.277692	0.401594	0.123901	0.019551	4.579080	3.000000	0.037790	0.210012	0.985889
Enterococcus mundtii	Dopamine	0.000001	k_lin	0.277692	0.375702	0.098009	0.007148	6.577867	3.000000	0.050591	0.145427	0.779865
Enterococcus mundtii	Noradrenaline	0.100000	k_lin	0.267281	0.373254	0.105974	0.019588	4.575911	3.000000	0.032271	0.179676	0.843240
Enterococcus mundtii	Noradrenaline	0.010000	k_lin	0.267281	0.354338	0.087057	0.067376	2.808509	3.000000	-0.011591	0.185706	0.692720
Enterococcus mundtii	Noradrenaline	0.001000	k_lin	0.267281	0.333483	0.066202	0.064817	2.855482	3.000000	-0.007580	0.139984	0.526773
Enterococcus mundtii	Noradrenaline	0.000100	k_lin	0.267281	0.347722	0.080441	0.034953	3.671923	3.000000	0.010723	0.150160	0.640078
Enterococcus mundtii	Noradrenaline	0.000010	k_lin	0.267281	0.339199	0.071918	0.061096	2.928067	3.000000	-0.006248	0.150085	0.572260
Enterococcus mundtii	Noradrenaline	0.000001	k_lin	0.267281	0.346743	0.079462	0.030559	3.868433	3.000000	0.014091	0.144834	0.632287
Enterococcus mundtii	Adrenaline	0.100000	k_lin	0.274883	0.396829	0.121946	0.024016	4.240327	3.000000	0.030423	0.213468	0.970330

Species	Hormon	Concentration	Parameter	Mean_Baseline	Mean_Treatment	Diff	P_Value	T_Statistic	DF	Lower_CI	Upper_CI	Diff_Norm
Enterococcus mundtii	Adrenaline	0.010000	k_lin	0.274883	0.377337	0.102455	0.041400	3.434546	3.000000	0.007520	0.197389	0.815237
Enterococcus mundtii	Adrenaline	0.001000	k_lin	0.274883	0.350031	0.075148	0.017943	4.726708	3.000000	0.024552	0.125745	0.597960
Enterococcus mundtii	Adrenaline	0.000100	k_lin	0.274883	0.340683	0.065801	0.006307	6.873213	3.000000	0.035333	0.096268	0.523579
Enterococcus mundtii	Adrenaline	0.000010	k_lin	0.274883	0.345731	0.070848	0.046806	3.269181	3.000000	0.001880	0.139817	0.563746
Enterococcus mundtii	Adrenaline	0.000001	k_lin	0.274883	0.359466	0.084583	0.018649	4.659907	3.000000	0.026818	0.142349	0.673035
Enterococcus mundtii	Levodopa	0.100000	death_lin	0.015073	0.000672	-0.014400	0.390803	-1.000483	3.000000	-0.060207	0.031406	-0.582907
Enterococcus mundtii	Levodopa	0.010000	death_lin	0.015073	0.000949	-0.014124	0.394199	-0.992299	3.000000	-0.059421	0.031173	-0.571711
Enterococcus mundtii	Levodopa	0.001000	death_lin	0.015073	0.000858	-0.014215	0.395440	-0.989324	3.000000	-0.059940	0.031511	-0.575384
Enterococcus mundtii	Levodopa	0.000100	death_lin	0.015073	0.000557	-0.014516	0.397781	-0.983739	3.000000	-0.061477	0.032444	-0.587592
Enterococcus mundtii	Levodopa	0.000010	death_lin	0.015073	0.000635	-0.014438	0.401136	-0.975787	3.000000	-0.061527	0.032650	-0.584428
Enterococcus mundtii	Levodopa	0.000001	death_lin	0.015073	0.019908	0.004835	0.102527	2.325941	3.000000	-0.001780	0.011450	0.195697
Enterococcus mundtii	Dopamine	0.100000	death_lin	0.024775	0.000532	-0.024243	0.208226	-1.598499	3.000000	-0.072509	0.024022	-0.981322
Enterococcus mundtii	Dopamine	0.010000	death_lin	0.024775	0.001071	-0.023704	0.218273	-1.552853	3.000000	-0.072284	0.024876	-0.959510
Enterococcus mundtii	Dopamine	0.001000	death_lin	0.024775	0.000334	-0.024442	0.210794	-1.586605	3.000000	-0.073467	0.024584	-0.989359
Enterococcus mundtii	Dopamine	0.000100	death_lin	0.024775	0.000071	-0.024705	0.207655	-1.601170	3.000000	-0.073807	0.024398	-1.000000
Enterococcus mundtii	Dopamine	0.000010	death_lin	0.024775	0.009745	-0.015031	0.387414	-1.008716	3.000000	-0.062452	0.032390	-0.608416
Enterococcus mundtii	Dopamine	0.000001	death_lin	0.024775	0.000162	-0.024613	0.212187	-1.580219	3.000000	-0.074183	0.024956	-0.996310
Enterococcus mundtii	Noradrenaline	0.100000	death_lin	0.009953	0.000637	-0.009317	0.396177	-0.987563	3.000000	-0.039340	0.020707	-0.377127
Enterococcus mundtii	Noradrenaline	0.010000	death_lin	0.009953	0.009406	-0.000548	0.910991	-0.121480	3.000000	-0.014893	0.013797	-0.022165
Enterococcus mundtii	Noradrenaline	0.001000	death_lin	0.009953	0.004352	-0.005602	0.641324	-0.516244	3.000000	-0.040135	0.028931	-0.226751
Enterococcus mundtii	Noradrenaline	0.000100	death_lin	0.009953	0.005323	-0.004630	0.503303	-0.758493	3.000000	-0.024058	0.014797	-0.187428
Enterococcus mundtii	Noradrenaline	0.000010	death_lin	0.009953	0.002053	-0.007900	0.486130	-0.792154	3.000000	-0.039639	0.023839	-0.319787
Enterococcus mundtii	Noradrenaline	0.000001	death_lin	0.009953	0.001755	-0.008198	0.428853	-0.912355	3.000000	-0.036795	0.020399	-0.331851
Enterococcus mundtii	Adrenaline	0.100000	death_lin	0.004713	0.002734	-0.001979	0.187948	-1.698650	3.000000	-0.005687	0.001729	-0.080114
Enterococcus mundtii	Adrenaline	0.010000	death_lin	0.004713	0.000032	-0.004681	0.126561	-2.100034	3.000000	-0.011774	0.002413	-0.189468
Enterococcus mundtii	Adrenaline	0.001000	death_lin	0.004713	0.013986	0.009273	0.425367	0.920123	3.000000	-0.022800	0.041346	0.375357
Enterococcus mundtii	Adrenaline	0.000100	death_lin	0.004713	0.002690	-0.002023	0.657888	-0.489751	3.000000	-0.015167	0.011122	-0.081881

Species	Hormon	Concentration	Parameter	Mean_Baseline	Mean_Treatment	Diff	P_Value	T_Statistic	DF	Lower_CI	Upper_CI	Diff_Norm
Enterococcus mundtii	Adrenaline	0.000010	death_lin	0.004713	0.003143	-0.001570	0.691239	-0.437642	3.000000	-0.012984	0.009844	-0.063536
Enterococcus mundtii	Adrenaline	0.000001	death_lin	0.004713	0.002577	-0.002136	0.538975	-0.691472	3.000000	-0.011969	0.007696	-0.086477
Enterococcus mundtii	Levodopa	0.100000	gr	0.593680	0.540974	-0.052706	0.004019	-8.039601	3.000000	-0.073570	-0.031843	-0.409534
Enterococcus mundtii	Levodopa	0.010000	gr	0.593680	0.550614	-0.043066	0.387795	-1.007787	3.000000	-0.179064	0.092931	-0.334632
Enterococcus mundtii	Levodopa	0.001000	gr	0.593680	0.557770	-0.035910	0.611752	-0.564692	3.000000	-0.238288	0.166468	-0.279025
Enterococcus mundtii	Levodopa	0.000100	gr	0.593680	0.554234	-0.039446	0.568192	-0.639132	3.000000	-0.235859	0.156968	-0.306499
Enterococcus mundtii	Levodopa	0.000010	gr	0.593680	0.555742	-0.037938	0.490503	-0.783490	3.000000	-0.192039	0.116162	-0.294785
Enterococcus mundtii	Levodopa	0.000001	gr	0.593680	0.550271	-0.043409	0.378515	-1.030667	3.000000	-0.177444	0.090627	-0.337292
Enterococcus mundtii	Dopamine	0.100000	gr	0.573154	0.597995	0.024841	0.366659	1.060695	3.000000	-0.049690	0.099372	0.193016
Enterococcus mundtii	Dopamine	0.010000	gr	0.573154	0.631824	0.058669	0.015948	4.935632	3.000000	0.020840	0.096499	0.455870
Enterococcus mundtii	Dopamine	0.001000	gr	0.573154	0.650997	0.077842	0.028821	3.956499	3.000000	0.015229	0.140455	0.604845
Enterococcus mundtii	Dopamine	0.000100	gr	0.573154	0.689305	0.116151	0.037787	3.561236	3.000000	0.012354	0.219948	0.902510
Enterococcus mundtii	Dopamine	0.000010	gr	0.573154	0.701852	0.128698	0.083238	2.559668	3.000000	-0.031313	0.288708	1.000000
Enterococcus mundtii	Dopamine	0.000001	gr	0.573154	0.631576	0.058422	0.007397	6.499347	3.000000	0.029815	0.087029	0.453946
Enterococcus mundtii	Noradrenaline	0.100000	gr	0.573438	0.623692	0.050255	0.075872	2.667194	3.000000	-0.009708	0.110218	0.390485
Enterococcus mundtii	Noradrenaline	0.010000	gr	0.573438	0.643632	0.070195	0.087489	2.502820	3.000000	-0.019061	0.159450	0.545421
Enterococcus mundtii	Noradrenaline	0.001000	gr	0.573438	0.668611	0.095173	0.006601	6.764456	3.000000	0.050397	0.139948	0.739505
Enterococcus mundtii	Noradrenaline	0.000100	gr	0.573438	0.666008	0.092570	0.002316	9.715467	3.000000	0.062247	0.122893	0.719282
Enterococcus mundtii	Noradrenaline	0.000010	gr	0.573438	0.635341	0.061904	0.011568	5.544427	3.000000	0.026372	0.097436	0.481000
Enterococcus mundtii	Noradrenaline	0.000001	gr	0.573438	0.612853	0.039415	0.075563	2.671973	3.000000	-0.007530	0.086361	0.306263
Enterococcus mundtii	Adrenaline	0.100000	gr	0.588762	0.620568	0.031806	0.086758	2.512355	3.000000	-0.008483	0.072096	0.247140
Enterococcus mundtii	Adrenaline	0.010000	gr	0.588762	0.642210	0.053448	0.011345	5.583267	3.000000	0.022983	0.083913	0.415299
Enterococcus mundtii	Adrenaline	0.001000	gr	0.588762	0.667041	0.078279	0.002788	9.117643	3.000000	0.050956	0.105601	0.608237
Enterococcus mundtii	Adrenaline	0.000100	gr	0.588762	0.660909	0.072147	0.006276	6.885164	3.000000	0.038800	0.105495	0.560595
Enterococcus mundtii	Adrenaline	0.000010	gr	0.588762	0.649023	0.060262	0.000532	15.984865	3.000000	0.048264	0.072259	0.468241
Enterococcus mundtii	Adrenaline	0.000001	gr	0.588762	0.618054	0.029292	0.008111	6.291321	3.000000	0.014475	0.044110	0.227605
Enterococcus mundtii	Levodopa	0.100000	dr	-0.019790	-0.011396	0.008394	0.671626	0.468094	3.000000	-0.048676	0.065464	0.152741

Species	Hormon	Concentration	Parameter	Mean_Baseline	Mean_Treatment	Diff	P_Value	T_Statistic	DF	Lower_CI	Upper_CI	Diff_Norm
Enterococcus mundtii	Levodopa	0.010000	dr	-0.019790	-0.014322	0.005468	0.764196	0.328400	3.000000	-0.047524	0.058461	0.099502
Enterococcus mundtii	Levodopa	0.001000	dr	-0.019790	-0.008970	0.010820	0.624213	0.544088	3.000000	-0.052469	0.074110	0.196886
Enterococcus mundtii	Levodopa	0.000100	dr	-0.019790	-0.009443	0.010347	0.653766	0.496304	3.000000	-0.056002	0.076697	0.188278
Enterococcus mundtii	Levodopa	0.000010	dr	-0.019790	-0.008424	0.011367	0.627827	0.538166	3.000000	-0.055850	0.078583	0.206826
Enterococcus mundtii	Levodopa	0.000001	dr	-0.019790	-0.023982	-0.004192	0.801662	-0.274294	3.000000	-0.052828	0.044444	-0.076277
Enterococcus mundtii	Dopamine	0.100000	dr	-0.049869	-0.008501	0.041368	0.138983	2.002542	3.000000	-0.024374	0.107109	0.752722
Enterococcus mundtii	Dopamine	0.010000	dr	-0.049869	-0.007354	0.042515	0.137502	2.013617	3.000000	-0.024678	0.109708	0.773602
Enterococcus mundtii	Dopamine	0.001000	dr	-0.049869	-0.007612	0.042257	0.121866	2.139886	3.000000	-0.020588	0.105102	0.768906
Enterococcus mundtii	Dopamine	0.000100	dr	-0.049869	-0.002677	0.047192	0.096622	2.391376	3.000000	-0.015611	0.109995	0.858703
Enterococcus mundtii	Dopamine	0.000010	dr	-0.049869	-0.019241	0.030628	0.182478	1.727775	3.000000	-0.025787	0.087043	0.557308
Enterococcus mundtii	Dopamine	0.000001	dr	-0.049869	0.005089	0.054957	0.121095	2.146607	3.000000	-0.026519	0.136434	1.000000
Enterococcus mundtii	Noradrenaline	0.100000	dr	-0.013590	-0.010262	0.003328	0.903766	0.131413	3.000000	-0.077270	0.083926	0.060559
Enterococcus mundtii	Noradrenaline	0.010000	dr	-0.013590	-0.021435	-0.007845	0.649135	-0.503697	3.000000	-0.057408	0.041719	-0.142740
Enterococcus mundtii	Noradrenaline	0.001000	dr	-0.013590	-0.007716	0.005874	0.829326	0.235014	3.000000	-0.073669	0.085418	0.106884
Enterococcus mundtii	Noradrenaline	0.000100	dr	-0.013590	-0.017526	-0.003936	0.828371	-0.236361	3.000000	-0.056933	0.049061	-0.071621
Enterococcus mundtii	Noradrenaline	0.000010	dr	-0.013590	-0.011412	0.002179	0.940247	0.081405	3.000000	-0.082994	0.087351	0.039643
Enterococcus mundtii	Noradrenaline	0.000001	dr	-0.013590	-0.009354	0.004236	0.813228	0.257809	3.000000	-0.048056	0.056528	0.077081
Enterococcus mundtii	Adrenaline	0.100000	dr	-0.049784	-0.011739	0.038045	0.201300	1.631423	3.000000	-0.036171	0.112261	0.692274
Enterococcus mundtii	Adrenaline	0.010000	dr	-0.049784	-0.001855	0.047930	0.120400	2.152705	3.000000	-0.022927	0.118786	0.872125
Enterococcus mundtii	Adrenaline	0.001000	dr	-0.049784	-0.023263	0.026521	0.309815	1.219413	3.000000	-0.042695	0.095738	0.482584
Enterococcus mundtii	Adrenaline	0.000100	dr	-0.049784	-0.008614	0.041170	0.201529	1.630315	3.000000	-0.039196	0.121536	0.749130
Enterococcus mundtii	Adrenaline	0.000010	dr	-0.049784	-0.011979	0.037806	0.212806	1.577397	3.000000	-0.038469	0.114080	0.687913
Enterococcus mundtii	Adrenaline	0.000001	dr	-0.049784	-0.013507	0.036277	0.241827	1.454368	3.000000	-0.043104	0.115658	0.660094
Enterococcus mundtii	Levodopa	0.100000	td	1.167734	1.282253	0.114519	0.005593	7.168011	3.000000	0.063675	0.165363	0.542214
Enterococcus mundtii	Levodopa	0.010000	td	1.167734	1.284807	0.117074	0.366240	1.061773	3.000000	-0.233830	0.467978	0.554309
Enterococcus mundtii	Levodopa	0.001000	td	1.167734	1.303340	0.135606	0.504182	0.756796	3.000000	-0.434640	0.705852	0.642056
Enterococcus mundtii	Levodopa	0.000100	td	1.167734	1.309104	0.141370	0.482139	0.800120	3.000000	-0.420924	0.703664	0.669345

Species	Hormon	Concentration	Parameter	Mean_Baseline	Mean_Treatment	Diff	P_Value	T_Statistic	DF	Lower_CI	Upper_CI	Diff_Norm
Enterococcus mundtii	Levodopa	0.000010	td	1.167734	1.279883	0.112149	0.446350	0.874208	3.000000	-0.296115	0.520413	0.530992
Enterococcus mundtii	Levodopa	0.000001	td	1.167734	1.283828	0.116094	0.329758	1.160634	3.000000	-0.202235	0.434424	0.549673
Enterococcus mundtii	Dopamine	0.100000	td	1.209873	1.163159	-0.046713	0.373312	-1.043730	3.000000	-0.189147	0.095720	-0.221173
Enterococcus mundtii	Dopamine	0.010000	td	1.209873	1.098362	-0.111510	0.013613	-5.228353	3.000000	-0.179385	-0.043635	-0.527968
Enterococcus mundtii	Dopamine	0.001000	td	1.209873	1.066095	-0.143778	0.026898	-4.062163	3.000000	-0.256418	-0.031137	-0.680744
Enterococcus mundtii	Dopamine	0.000100	td	1.209873	1.010684	-0.199189	0.025206	-4.163597	3.000000	-0.351439	-0.046939	-0.943100
Enterococcus mundtii	Dopamine	0.000010	td	1.209873	0.998666	-0.211206	0.053026	-3.106504	3.000000	-0.427576	0.005163	-1.000000
Enterococcus mundtii	Dopamine	0.000001	td	1.209873	1.097613	-0.112260	0.008419	-6.209084	3.000000	-0.169799	-0.054722	-0.531519
Enterococcus mundtii	Noradrenaline	0.100000	td	1.208778	1.114511	-0.094268	0.065601	-2.840848	3.000000	-0.199870	0.011335	-0.446329
Enterococcus mundtii	Noradrenaline	0.010000	td	1.208778	1.082924	-0.125854	0.075426	-2.674107	3.000000	-0.275633	0.023925	-0.595883
Enterococcus mundtii	Noradrenaline	0.001000	td	1.208778	1.037938	-0.170840	0.004782	-7.569472	3.000000	-0.242666	-0.099013	-0.808877
Enterococcus mundtii	Noradrenaline	0.000100	td	1.208778	1.041359	-0.167419	0.001579	-11.070053	3.000000	-0.215549	-0.119289	-0.792681
Enterococcus mundtii	Noradrenaline	0.000010	td	1.208778	1.091962	-0.116817	0.008343	-6.228936	3.000000	-0.176500	-0.057133	-0.553092
Enterococcus mundtii	Noradrenaline	0.000001	td	1.208778	1.132908	-0.075870	0.076720	-2.654168	3.000000	-0.166840	0.015101	-0.359221
Enterococcus mundtii	Adrenaline	0.100000	td	1.177415	1.118046	-0.059370	0.080520	-2.597931	3.000000	-0.132097	0.013358	-0.281099
Enterococcus mundtii	Adrenaline	0.010000	td	1.177415	1.079857	-0.097558	0.009997	-5.841527	3.000000	-0.150708	-0.044409	-0.461909
Enterococcus mundtii	Adrenaline	0.001000	td	1.177415	1.039454	-0.137962	0.002358	-9.656287	3.000000	-0.183430	-0.092493	-0.653207
Enterococcus mundtii	Adrenaline	0.000100	td	1.177415	1.049887	-0.127529	0.003771	-8.218308	3.000000	-0.176913	-0.078145	-0.603812
Enterococcus mundtii	Adrenaline	0.000010	td	1.177415	1.068061	-0.109354	0.000587	-15.465416	3.000000	-0.131857	-0.086851	-0.517760
Enterococcus mundtii	Adrenaline	0.000001	td	1.177415	1.122028	-0.055387	0.006028	-6.982976	3.000000	-0.080629	-0.030145	-0.262241
Enterococcus mundtii	Levodopa	0.100000	lagC	4.133551	4.250772	0.117221	0.687575	0.443291	3.000000	-0.724327	0.958769	0.197714
Enterococcus mundtii	Levodopa	0.010000	lagC	4.133551	3.978471	-0.155080	0.609508	-0.568432	3.000000	-1.023313	0.713154	-0.261569
Enterococcus mundtii	Levodopa	0.001000	lagC	4.133551	3.900795	-0.232756	0.383550	-1.018187	3.000000	-0.960260	0.494747	-0.392585
Enterococcus mundtii	Levodopa	0.000100	lagC	4.133551	3.973392	-0.160158	0.503488	-0.758137	3.000000	-0.832459	0.512142	-0.270135
Enterococcus mundtii	Levodopa	0.000010	lagC	4.133551	3.987689	-0.145862	0.491650	-0.781227	3.000000	-0.740054	0.448330	-0.246022
Enterococcus mundtii	Levodopa	0.000001	lagC	4.133551	3.910732	-0.222819	0.381483	-1.023292	3.000000	-0.915789	0.470150	-0.375824
Enterococcus mundtii	Dopamine	0.100000	lagC	4.371308	4.158062	-0.213246	0.409980	-0.955118	3.000000	-0.923782	0.497289	-0.359678

Species	Hormon	Concentration	Parameter	Mean_Baseline	Mean_Treatment	Diff	P_Value	T_Statistic	DF	Lower_CI	Upper_CI	Diff_Norm
Enterococcus mundtii	Dopamine	0.010000	lagC	4.371308	3.924816	-0.446493	0.095146	-2.408492	3.000000	-1.036463	0.143478	-0.753089
Enterococcus mundtii	Dopamine	0.001000	lagC	4.371308	3.983362	-0.387946	0.172566	-1.783180	3.000000	-1.080316	0.304423	-0.654340
Enterococcus mundtii	Dopamine	0.000100	lagC	4.371308	3.900878	-0.470431	0.194106	-1.666995	3.000000	-1.368526	0.427665	-0.793464
Enterococcus mundtii	Dopamine	0.000010	lagC	4.371308	3.936630	-0.434678	0.138455	-2.006474	3.000000	-1.124117	0.254760	-0.733162
Enterococcus mundtii	Dopamine	0.000001	lagC	4.371308	3.970827	-0.400482	0.210758	-1.586769	3.000000	-1.203693	0.402730	-0.675483
Enterococcus mundtii	Noradrenaline	0.100000	lagC	4.642512	4.224959	-0.417553	0.243957	-1.445987	3.000000	-1.336539	0.501432	-0.704277
Enterococcus mundtii	Noradrenaline	0.010000	lagC	4.642512	4.049630	-0.592882	0.109719	-2.252158	3.000000	-1.430663	0.244899	-1.000000
Enterococcus mundtii	Noradrenaline	0.001000	lagC	4.642512	4.139541	-0.502971	0.166120	-1.821209	3.000000	-1.381881	0.375939	-0.848350
Enterococcus mundtii	Noradrenaline	0.000100	lagC	4.642512	4.174855	-0.467658	0.149156	-1.930068	3.000000	-1.238768	0.303453	-0.788787
Enterococcus mundtii	Noradrenaline	0.000010	lagC	4.642512	4.200876	-0.441636	0.197720	-1.648943	3.000000	-1.293991	0.410718	-0.744898
Enterococcus mundtii	Noradrenaline	0.000001	lagC	4.642512	4.254148	-0.388364	0.199146	-1.641923	3.000000	-1.141108	0.364380	-0.655044
Enterococcus mundtii	Adrenaline	0.100000	lagC	4.577189	4.237632	-0.339557	0.186381	-1.706896	3.000000	-0.972649	0.293535	-0.572723
Enterococcus mundtii	Adrenaline	0.010000	lagC	4.577189	4.213526	-0.363662	0.222794	-1.533070	3.000000	-1.118576	0.391252	-0.613381
Enterococcus mundtii	Adrenaline	0.001000	lagC	4.577189	4.181895	-0.395294	0.197322	-1.650911	3.000000	-1.157298	0.366710	-0.666733
Enterococcus mundtii	Adrenaline	0.000100	lagC	4.577189	4.251060	-0.326128	0.239411	-1.463977	3.000000	-1.035078	0.382821	-0.550073
Enterococcus mundtii	Adrenaline	0.000010	lagC	4.577189	4.231426	-0.345762	0.193969	-1.667689	3.000000	-1.005579	0.314055	-0.583189
Enterococcus mundtii	Adrenaline	0.000001	lagC	4.577189	4.231148	-0.346040	0.167080	-1.815437	3.000000	-0.952646	0.260565	-0.583658
Enterococcus mundtii	Levodopa	0.100000	t_k	37.500000	47.666667	10.166667	0.395545	0.989074	3.000000	-22.545632	42.878965	0.500000
Enterococcus mundtii	Levodopa	0.010000	t_k	37.500000	47.666667	10.166667	0.391039	0.999910	3.000000	-22.191102	42.524436	0.500000
Enterococcus mundtii	Levodopa	0.001000	t_k	37.500000	46.333333	8.833333	0.396282	0.987311	3.000000	-19.639567	37.306234	0.434426
Enterococcus mundtii	Levodopa	0.000100	t_k	37.500000	47.750000	10.250000	0.395544	0.989077	3.000000	-22.730315	43.230315	0.504098
Enterococcus mundtii	Levodopa	0.000010	t_k	37.500000	46.000000	8.500000	0.499585	0.765699	3.000000	-26.828233	43.828233	0.418033
Enterococcus mundtii	Levodopa	0.000001	t_k	37.500000	23.416667	-14.083333	0.230478	-1.500440	3.000000	-43.954205	15.787538	-0.692623
Enterococcus mundtii	Dopamine	0.100000	t_k	27.583333	47.666667	20.083333	0.184337	1.717766	3.000000	-17.124368	57.291035	0.987705
Enterococcus mundtii	Dopamine	0.010000	t_k	27.583333	46.083333	18.500000	0.187374	1.701662	3.000000	-16.098687	53.098687	0.909836
Enterococcus mundtii	Dopamine	0.001000	t_k	27.583333	47.833333	20.250000	0.181696	1.732021	3.000000	-16.957701	57.457701	0.995902
Enterococcus mundtii	Dopamine	0.000100	t_k	27.583333	47.916667	20.333333	0.181690	1.732051	3.000000	-17.026853	57.693520	1.000000

Species	Hormon	Concentration	Parameter	Mean_Baseline	Mean_Treatment	Diff	P_Value	T_Statistic	DF	Lower_CI	Upper_CI	Diff_Norm
Enterococcus mundtii	Dopamine	0.000010	t_k	27.583333	33.500000	5.916667	0.406378	0.963485	3.000000	-13.626417	25.459750	0.290984
Enterococcus mundtii	Dopamine	0.000001	t_k	27.583333	47.916667	20.333333	0.184310	1.717912	3.000000	-17.334329	58.000996	1.000000
Enterococcus mundtii	Noradrenaline	0.100000	t_k	37.833333	47.666667	9.833333	0.395700	0.988703	3.000000	-21.818278	41.484944	0.483607
Enterococcus mundtii	Noradrenaline	0.010000	t_k	37.833333	34.166667	-3.666667	0.365925	-1.062583	3.000000	-14.648370	7.315036	-0.180328
Enterococcus mundtii	Noradrenaline	0.001000	t_k	37.833333	39.500000	1.666667	0.907798	0.125868	3.000000	-40.473217	43.806550	0.081967
Enterococcus mundtii	Noradrenaline	0.000100	t_k	37.833333	35.083333	-2.750000	0.356422	-1.087387	3.000000	-10.798402	5.298402	-0.135246
Enterococcus mundtii	Noradrenaline	0.000010	t_k	37.833333	41.750000	3.916667	0.770849	0.318707	3.000000	-35.193185	43.026519	0.192623
Enterococcus mundtii	Noradrenaline	0.000001	t_k	37.833333	44.500000	6.666667	0.591441	0.598908	3.000000	-28.758322	42.091655	0.327869
Enterococcus mundtii	Adrenaline	0.100000	t_k	27.666667	44.500000	16.833333	0.196381	1.655589	3.000000	-15.524436	49.191102	0.827869
Enterococcus mundtii	Adrenaline	0.010000	t_k	27.666667	47.916667	20.250000	0.179098	1.746274	3.000000	-16.654017	57.154017	0.995902
Enterococcus mundtii	Adrenaline	0.001000	t_k	27.666667	29.333333	1.666667	0.873853	0.172739	3.000000	-29.039008	32.372342	0.081967
Enterococcus mundtii	Adrenaline	0.000100	t_k	27.666667	42.500000	14.833333	0.390043	1.002323	3.000000	-32.263567	61.930233	0.729508
Enterococcus mundtii	Adrenaline	0.000010	t_k	27.666667	42.916667	15.250000	0.367011	1.059788	3.000000	-30.544341	61.044341	0.750000
Enterococcus mundtii	Adrenaline	0.000001	t_k	27.666667	44.333333	16.666667	0.305149	1.233722	3.000000	-26.325815	59.659149	0.819672
Enterococcus mundtii	Levodopa	0.100000	t_gr	5.250000	5.250000	0.000000	1.000000	0.000000	3.000000	-0.866152	0.866152	0.000000
Enterococcus mundtii	Levodopa	0.010000	t_gr	5.250000	5.083333	-0.166667	0.604181	-0.577350	3.000000	-1.085360	0.752026	-0.250000
Enterococcus mundtii	Levodopa	0.001000	t_gr	5.250000	4.916667	-0.333333	0.252215	-1.414214	3.000000	-1.083443	0.416776	-0.500000
Enterococcus mundtii	Levodopa	0.000100	t_gr	5.250000	5.000000	-0.250000	0.391002	-1.000000	3.000000	-1.045612	0.545612	-0.375000
Enterococcus mundtii	Levodopa	0.000010	t_gr	5.250000	5.083333	-0.166667	0.391002	-1.000000	3.000000	-0.697074	0.363741	-0.250000
Enterococcus mundtii	Levodopa	0.000001	t_gr	5.250000	5.000000	-0.250000	0.444438	-0.878310	3.000000	-1.155844	0.655844	-0.375000
Enterococcus mundtii	Dopamine	0.100000	t_gr	5.500000	5.166667	-0.333333	0.353387	-1.095445	3.000000	-1.301721	0.635054	-0.500000
Enterococcus mundtii	Dopamine	0.010000	t_gr	5.500000	5.000000	-0.500000	0.102728	-2.323790	3.000000	-1.184753	0.184753	-0.750000
Enterococcus mundtii	Dopamine	0.001000	t_gr	5.500000	5.083333	-0.416667	0.194171	-1.666667	3.000000	-1.212278	0.378945	-0.625000
Enterococcus mundtii	Dopamine	0.000100	t_gr	5.500000	5.083333	-0.416667	0.341576	-1.127469	3.000000	-1.592769	0.759436	-0.625000
Enterococcus mundtii	Dopamine	0.000010	t_gr	5.500000	5.083333	-0.416667	0.194171	-1.666667	3.000000	-1.212278	0.378945	-0.625000
Enterococcus mundtii	Dopamine	0.000001	t_gr	5.500000	5.083333	-0.416667	0.194171	-1.666667	3.000000	-1.212278	0.378945	-0.625000
Enterococcus mundtii	Noradrenaline	0.100000	t_gr	5.833333	5.250000	-0.583333	0.132842	-2.049390	3.000000	-1.489177	0.322510	-0.875000

Species	Hormon	Concentration	Parameter	Mean_Baseline	Mean_Treatment	Diff	P_Value	T_Statistic	DF	Lower_CI	Upper_CI	Diff_Norm
Enterococcus mundtii	Noradrenaline	0.010000	t_gr	5.833333	5.166667	-0.666667	0.116158	-2.190890	3.000000	-1.635054	0.301721	-1.000000
Enterococcus mundtii	Noradrenaline	0.001000	t_gr	5.833333	5.250000	-0.583333	0.188120	-1.697749	3.000000	-1.676797	0.510130	-0.875000
Enterococcus mundtii	Noradrenaline	0.000100	t_gr	5.833333	5.333333	-0.500000	0.102728	-2.323790	3.000000	-1.184753	0.184753	-0.750000
Enterococcus mundtii	Noradrenaline	0.000010	t_gr	5.833333	5.333333	-0.500000	0.215170	-1.566699	3.000000	-1.515653	0.515653	-0.750000
Enterococcus mundtii	Noradrenaline	0.000001	t_gr	5.833333	5.416667	-0.416667	0.194171	-1.666667	3.000000	-1.212278	0.378945	-0.625000
Enterococcus mundtii	Adrenaline	0.100000	t_gr	5.750000	5.250000	-0.500000	0.102728	-2.323790	3.000000	-1.184753	0.184753	-0.750000
Enterococcus mundtii	Adrenaline	0.010000	t_gr	5.750000	5.250000	-0.500000	0.102728	-2.323790	3.000000	-1.184753	0.184753	-0.750000
Enterococcus mundtii	Adrenaline	0.001000	t_gr	5.750000	5.333333	-0.416667	0.141122	-1.986799	3.000000	-1.084082	0.250748	-0.625000
Enterococcus mundtii	Adrenaline	0.000100	t_gr	5.750000	5.333333	-0.416667	0.141122	-1.986799	3.000000	-1.084082	0.250748	-0.625000
Enterococcus mundtii	Adrenaline	0.000010	t_gr	5.750000	5.333333	-0.416667	0.141122	-1.986799	3.000000	-1.084082	0.250748	-0.625000
Enterococcus mundtii	Adrenaline	0.000001	t_gr	5.750000	5.333333	-0.416667	0.079605	-2.611165	3.000000	-0.924493	0.091160	-0.625000
Enterococcus mundtii	Levodopa	0.100000	t_dr	37.833333	48.000000	10.166667	0.391002	1.000000	3.000000	-22.188204	42.521537	0.506224
Enterococcus mundtii	Levodopa	0.010000	t_dr	37.833333	48.000000	10.166667	0.391002	1.000000	3.000000	-22.188204	42.521537	0.506224
Enterococcus mundtii	Levodopa	0.001000	t_dr	37.833333	48.000000	10.166667	0.391002	1.000000	3.000000	-22.188204	42.521537	0.506224
Enterococcus mundtii	Levodopa	0.000100	t_dr	37.833333	48.000000	10.166667	0.391002	1.000000	3.000000	-22.188204	42.521537	0.506224
Enterococcus mundtii	Levodopa	0.000010	t_dr	37.833333	48.000000	10.166667	0.391002	1.000000	3.000000	-22.188204	42.521537	0.506224
Enterococcus mundtii	Levodopa	0.000001	t_dr	37.833333	28.333333	-9.500000	0.381409	-1.023475	3.000000	-39.039789	20.039789	-0.473029
Enterococcus mundtii	Dopamine	0.100000	t_dr	27.916667	48.000000	20.083333	0.181696	1.732021	3.000000	-16.818142	56.984809	1.000000
Enterococcus mundtii	Dopamine	0.010000	t_dr	27.916667	48.000000	20.083333	0.181696	1.732021	3.000000	-16.818142	56.984809	1.000000
Enterococcus mundtii	Dopamine	0.001000	t_dr	27.916667	48.000000	20.083333	0.181696	1.732021	3.000000	-16.818142	56.984809	1.000000
Enterococcus mundtii	Dopamine	0.000100	t_dr	27.916667	48.000000	20.083333	0.181696	1.732021	3.000000	-16.818142	56.984809	1.000000
Enterococcus mundtii	Dopamine	0.000010	t_dr	27.916667	34.333333	6.416667	0.405192	0.966257	3.000000	-14.717158	27.550491	0.319502
Enterococcus mundtii	Dopamine	0.000001	t_dr	27.916667	48.000000	20.083333	0.181696	1.732021	3.000000	-16.818142	56.984809	1.000000
Enterococcus mundtii	Noradrenaline	0.100000	t_dr	38.166667	48.000000	9.833333	0.391002	1.000000	3.000000	-21.460722	41.127389	0.489627
Enterococcus mundtii	Noradrenaline	0.010000	t_dr	38.166667	38.000000	-0.166667	0.391002	-1.000000	3.000000	-0.697074	0.363741	-0.008299
Enterococcus mundtii	Noradrenaline	0.001000	t_dr	38.166667	44.333333	6.166667	0.631377	0.532370	3.000000	-30.696988	43.030321	0.307054
Enterococcus mundtii	Noradrenaline	0.000100	t_dr	38.166667	38.583333	0.416667	0.391002	1.000000	3.000000	-0.909353	1.742686	0.020747

Species	Hormon	Concentration	Parameter	Mean_Baseline	Mean_Treatment	Diff	P_Value	T_Statistic	DF	Lower_CI	Upper_CI	Diff_Norm
Enterococcus mundtii	Noradrenaline	0.000010	t_dr	38.166667	48.000000	9.833333	0.391002	1.000000	3.000000	-21.460722	41.127389	0.489627
Enterococcus mundtii	Noradrenaline	0.000001	t_dr	38.166667	46.500000	8.333333	0.482697	0.799003	3.000000	-24.858495	41.525162	0.414938
Enterococcus mundtii	Adrenaline	0.100000	t_dr	28.083333	46.500000	18.416667	0.184551	1.716620	3.000000	-15.726034	52.559368	0.917012
Enterococcus mundtii	Adrenaline	0.010000	t_dr	28.083333	48.000000	19.916667	0.181740	1.731778	3.000000	-16.683708	56.517041	0.991701
Enterococcus mundtii	Adrenaline	0.001000	t_dr	28.083333	34.083333	6.000000	0.630992	0.532997	3.000000	-29.825105	41.825105	0.298755
Enterococcus mundtii	Adrenaline	0.000100	t_dr	28.083333	48.000000	19.916667	0.181740	1.731778	3.000000	-16.683708	56.517041	0.991701
Enterococcus mundtii	Adrenaline	0.000010	t_dr	28.083333	48.000000	19.916667	0.181740	1.731778	3.000000	-16.683708	56.517041	0.991701
Enterococcus mundtii	Adrenaline	0.000001	t_dr	28.083333	47.833333	19.750000	0.181832	1.731280	3.000000	-16.554526	56.054526	0.983402
Escherichia coli	Levodopa	0.100000	auc_lin	7.791542	11.395026	3.603484	0.021269	4.437935	3.000000	1.019423	6.187545	0.494523
Escherichia coli	Levodopa	0.010000	auc_lin	7.791542	11.820645	4.029103	0.018706	4.654620	3.000000	1.274334	6.783872	0.552932
Escherichia coli	Levodopa	0.001000	auc_lin	7.791542	12.338090	4.546548	0.011654	5.529690	3.000000	1.929920	7.163177	0.623944
Escherichia coli	Levodopa	0.000100	auc_lin	7.791542	12.433324	4.641782	0.009847	5.873018	3.000000	2.126513	7.157052	0.637013
Escherichia coli	Levodopa	0.000010	auc_lin	7.791542	11.955650	4.164109	0.009160	6.026024	3.000000	1.964972	6.363246	0.571460
Escherichia coli	Levodopa	0.000001	auc_lin	7.791542	10.637043	2.845501	0.016386	4.886964	3.000000	0.992479	4.698524	0.390501
Escherichia coli	Dopamine	0.100000	auc_lin	5.270732	12.192471	6.921739	0.006619	6.757909	3.000000	3.662141	10.181336	0.949902
Escherichia coli	Dopamine	0.010000	auc_lin	5.270732	12.274976	7.004244	0.000985	12.991530	3.000000	5.288462	8.720026	0.961224
Escherichia coli	Dopamine	0.001000	auc_lin	5.270732	12.414442	7.143710	0.000455	16.853996	3.000000	5.794803	8.492617	0.980364
Escherichia coli	Dopamine	0.000100	auc_lin	5.270732	12.557526	7.286795	0.000485	16.488760	3.000000	5.880392	8.693197	1.000000
Escherichia coli	Dopamine	0.000010	auc_lin	5.270732	11.676337	6.405605	0.006655	6.745028	3.000000	3.383306	9.427904	0.879070
Escherichia coli	Dopamine	0.000001	auc_lin	5.270732	8.197887	2.927156	0.008644	6.151349	3.000000	1.412770	4.441541	0.401707
Escherichia coli	Noradrenaline	0.100000	auc_lin	5.380552	12.033050	6.652497	0.000090	28.956423	3.000000	5.921357	7.383638	0.912953
Escherichia coli	Noradrenaline	0.010000	auc_lin	5.380552	11.838222	6.457670	0.000118	26.518943	3.000000	5.682707	7.232632	0.886215
Escherichia coli	Noradrenaline	0.001000	auc_lin	5.380552	11.955646	6.575094	0.000097	28.327608	3.000000	5.836419	7.313768	0.902330
Escherichia coli	Noradrenaline	0.000100	auc_lin	5.380552	11.895798	6.515245	0.000035	39.753363	3.000000	5.993669	7.036822	0.894117
Escherichia coli	Noradrenaline	0.000010	auc_lin	5.380552	11.628699	6.248146	0.001211	12.111864	3.000000	4.606418	7.889874	0.857462
Escherichia coli	Noradrenaline	0.000001	auc_lin	5.380552	7.940040	2.559487	0.001615	10.985824	3.000000	1.818038	3.300936	0.351250
Escherichia coli	Adrenaline	0.100000	auc_lin	7.116155	12.906224	5.790068	0.004789	7.565578	3.000000	3.354487	8.225650	0.794597

Species	Hormon	Concentration	Parameter	Mean_Baseline	Mean_Treatment	Diff	P_Value	T_Statistic	DF	Lower_CI	Upper_CI	Diff_Norm
Escherichia coli	Adrenaline	0.010000	auc_lin	7.116155	12.330920	5.214764	0.000562	15.698582	3.000000	4.157617	6.271911	0.715646
Escherichia coli	Adrenaline	0.001000	auc_lin	7.116155	12.227601	5.111446	0.000349	18.420261	3.000000	4.228348	5.994544	0.701467
Escherichia coli	Adrenaline	0.000100	auc_lin	7.116155	12.585071	5.468916	0.000120	26.378802	3.000000	4.809124	6.128709	0.750524
Escherichia coli	Adrenaline	0.000010	auc_lin	7.116155	11.780579	4.664423	0.002763	9.146593	3.000000	3.041494	6.287353	0.640120
Escherichia coli	Adrenaline	0.000001	auc_lin	7.116155	10.415096	3.298940	0.027632	4.020710	3.000000	0.687784	5.910096	0.452729
Escherichia coli	Levodopa	0.100000	k_lin	0.234260	0.291440	0.057180	0.061133	2.927312	3.000000	-0.004984	0.119345	0.440310
Escherichia coli	Levodopa	0.010000	k_lin	0.234260	0.284200	0.049940	0.082971	2.563363	3.000000	-0.012061	0.111942	0.384559
Escherichia coli	Levodopa	0.001000	k_lin	0.234260	0.297482	0.063222	0.048966	3.209747	3.000000	0.000538	0.125906	0.486830
Escherichia coli	Levodopa	0.000100	k_lin	0.234260	0.300420	0.066160	0.033049	3.752957	3.000000	0.010057	0.122262	0.509454
Escherichia coli	Levodopa	0.000010	k_lin	0.234260	0.282881	0.048621	0.057973	2.993411	3.000000	-0.003070	0.100312	0.374399
Escherichia coli	Levodopa	0.000001	k_lin	0.234260	0.272919	0.038659	0.065225	2.847835	3.000000	-0.004542	0.081860	0.297688
Escherichia coli	Dopamine	0.100000	k_lin	0.193167	0.323031	0.129864	0.028025	3.999095	3.000000	0.026519	0.233209	1.000000
Escherichia coli	Dopamine	0.010000	k_lin	0.193167	0.306872	0.113704	0.008571	6.169931	3.000000	0.055056	0.172353	0.875564
Escherichia coli	Dopamine	0.001000	k_lin	0.193167	0.313555	0.120388	0.003158	8.735683	3.000000	0.076530	0.164246	0.927031
Escherichia coli	Dopamine	0.000100	k_lin	0.193167	0.320058	0.126891	0.007207	6.559036	3.000000	0.065324	0.188459	0.977108
Escherichia coli	Dopamine	0.000010	k_lin	0.193167	0.315638	0.122471	0.026390	4.091774	3.000000	0.027217	0.217725	0.943071
Escherichia coli	Dopamine	0.000001	k_lin	0.193167	0.256844	0.063677	0.006128	6.942922	3.000000	0.034489	0.092865	0.490336
Escherichia coli	Noradrenaline	0.100000	k_lin	0.183344	0.295261	0.111916	0.000583	15.502885	3.000000	0.088942	0.134891	0.861796
Escherichia coli	Noradrenaline	0.010000	k_lin	0.183344	0.288821	0.105476	0.000356	18.296583	3.000000	0.087130	0.123822	0.812204
Escherichia coli	Noradrenaline	0.001000	k_lin	0.183344	0.285060	0.101716	0.000127	25.834636	3.000000	0.089186	0.114246	0.783248
Escherichia coli	Noradrenaline	0.000100	k_lin	0.183344	0.288810	0.105465	0.000437	17.083466	3.000000	0.085818	0.125112	0.812120
Escherichia coli	Noradrenaline	0.000010	k_lin	0.183344	0.285860	0.102515	0.001426	11.460754	3.000000	0.074049	0.130982	0.789406
Escherichia coli	Noradrenaline	0.000001	k_lin	0.183344	0.221435	0.038091	0.025608	4.138708	3.000000	0.008801	0.067381	0.293314
Escherichia coli	Adrenaline	0.100000	k_lin	0.226686	0.325899	0.099212	0.028322	3.983004	3.000000	0.019941	0.178483	0.763970
Escherichia coli	Adrenaline	0.010000	k_lin	0.226686	0.293783	0.067097	0.020029	4.538258	3.000000	0.020045	0.114149	0.516671
Escherichia coli	Adrenaline	0.001000	k_lin	0.226686	0.292605	0.065919	0.011696	5.522408	3.000000	0.027931	0.103906	0.507597
Escherichia coli	Adrenaline	0.000100	k_lin	0.226686	0.303452	0.076766	0.008199	6.267416	3.000000	0.037786	0.115745	0.591122

Species	Hormon	Concentration	Parameter	Mean_Baseline	Mean_Treatment	Diff	P_Value	T_Statistic	DF	Lower_CI	Upper_CI	Diff_Norm
Escherichia coli	Adrenaline	0.000010	k_lin	0.226686	0.287565	0.060879	0.035548	3.647764	3.000000	0.007766	0.113992	0.468789
Escherichia coli	Adrenaline	0.000001	k_lin	0.226686	0.281384	0.054698	0.030788	3.857340	3.000000	0.009570	0.099826	0.421196
Escherichia coli	Levodopa	0.100000	death_lin	0.004329	0.002046	-0.002284	0.609511	-0.568426	3.000000	-0.015069	0.010501	-0.053675
Escherichia coli	Levodopa	0.010000	death_lin	0.004329	0.003998	-0.000331	0.938715	-0.083498	3.000000	-0.012944	0.012282	-0.007779
Escherichia coli	Levodopa	0.001000	death_lin	0.004329	0.011420	0.007090	0.219293	1.548351	3.000000	-0.007483	0.021664	0.166657
Escherichia coli	Levodopa	0.000100	death_lin	0.004329	0.019996	0.015667	0.028702	3.962749	3.000000	0.003085	0.028248	0.368239
Escherichia coli	Levodopa	0.000010	death_lin	0.004329	0.008123	0.003793	0.438170	0.891872	3.000000	-0.009743	0.017329	0.089163
Escherichia coli	Levodopa	0.000001	death_lin	0.004329	0.006418	0.002089	0.716846	0.398626	3.000000	-0.014586	0.018764	0.049094
Escherichia coli	Dopamine	0.100000	death_lin	0.027645	0.001404	-0.026241	0.206292	-1.607568	3.000000	-0.078190	0.025708	-0.616793
Escherichia coli	Dopamine	0.010000	death_lin	0.027645	0.004232	-0.023413	0.244342	-1.444478	3.000000	-0.074997	0.028170	-0.550323
Escherichia coli	Dopamine	0.001000	death_lin	0.027645	0.003169	-0.024476	0.245505	-1.439944	3.000000	-0.078571	0.029619	-0.575308
Escherichia coli	Dopamine	0.000100	death_lin	0.027645	0.002788	-0.024856	0.241826	-1.454373	3.000000	-0.079247	0.029534	-0.584247
Escherichia coli	Dopamine	0.000010	death_lin	0.027645	0.005549	-0.022095	0.339789	-1.132408	3.000000	-0.084191	0.040000	-0.519350
Escherichia coli	Dopamine	0.000001	death_lin	0.027645	0.001573	-0.026072	0.214172	-1.571199	3.000000	-0.078879	0.026736	-0.612809
Escherichia coli	Noradrenaline	0.100000	death_lin	0.043102	0.002018	-0.041083	0.152722	-1.906025	3.000000	-0.109680	0.027513	-0.965660
Escherichia coli	Noradrenaline	0.010000	death_lin	0.043102	0.014714	-0.028388	0.215481	-1.565299	3.000000	-0.086105	0.029329	-0.667261
Escherichia coli	Noradrenaline	0.001000	death_lin	0.043102	0.011796	-0.031306	0.292479	-1.273748	3.000000	-0.109524	0.046912	-0.735843
Escherichia coli	Noradrenaline	0.000100	death_lin	0.043102	0.014646	-0.028456	0.346791	-1.113196	3.000000	-0.109806	0.052895	-0.668850
Escherichia coli	Noradrenaline	0.000010	death_lin	0.043102	0.005058	-0.038044	0.202264	-1.626766	3.000000	-0.112470	0.036382	-0.894219
Escherichia coli	Noradrenaline	0.000001	death_lin	0.043102	0.000557	-0.042544	0.144004	-1.966022	3.000000	-0.111412	0.026323	-1.000000
Escherichia coli	Adrenaline	0.100000	death_lin	0.000198	0.001194	0.000996	0.189632	1.689880	3.000000	-0.000879	0.002871	0.023403
Escherichia coli	Adrenaline	0.010000	death_lin	0.000198	0.005640	0.005443	0.056749	3.020215	3.000000	-0.000292	0.011177	0.127925
Escherichia coli	Adrenaline	0.001000	death_lin	0.000198	0.013199	0.013001	0.050694	3.164533	3.000000	-0.000074	0.026075	0.305580
Escherichia coli	Adrenaline	0.000100	death_lin	0.000198	0.018809	0.018611	0.004290	7.859850	3.000000	0.011076	0.026147	0.437455
Escherichia coli	Adrenaline	0.000010	death_lin	0.000198	0.006463	0.006265	0.093285	2.430536	3.000000	-0.001938	0.014469	0.147267
Escherichia coli	Adrenaline	0.000001	death_lin	0.000198	0.001773	0.001576	0.204663	1.615282	3.000000	-0.001529	0.004680	0.037032
Escherichia coli	Levodopa	0.100000	gr	0.410687	0.381503	-0.029184	0.052449	-3.120557	3.000000	-0.058946	0.000579	-0.269359

Species	Hormon	Concentration	Parameter	Mean_Baseline	Mean_Treatment	Diff	P_Value	T_Statistic	DF	Lower_CI	Upper_CI	Diff_Norm
Escherichia coli	Levodopa	0.010000	gr	0.410687	0.434168	0.023482	0.417689	0.937437	3.000000	-0.056235	0.103199	0.216731
Escherichia coli	Levodopa	0.001000	gr	0.410687	0.465176	0.054490	0.036984	3.591486	3.000000	0.006206	0.102774	0.502927
Escherichia coli	Levodopa	0.000100	gr	0.410687	0.474826	0.064139	0.000820	13.822066	3.000000	0.049372	0.078907	0.591989
Escherichia coli	Levodopa	0.000010	gr	0.410687	0.451430	0.040743	0.028313	3.983500	3.000000	0.008193	0.073293	0.376048
Escherichia coli	Levodopa	0.000001	gr	0.410687	0.386391	-0.024295	0.581782	-0.615477	3.000000	-0.149919	0.101328	-0.224239
Escherichia coli	Dopamine	0.100000	gr	0.398923	0.376733	-0.022190	0.045722	-3.300340	3.000000	-0.043587	-0.000793	-0.204808
Escherichia coli	Dopamine	0.010000	gr	0.398923	0.442733	0.043811	0.406640	0.962875	3.000000	-0.100990	0.188612	0.404362
Escherichia coli	Dopamine	0.001000	gr	0.398923	0.468799	0.069876	0.009882	5.865755	3.000000	0.031965	0.107787	0.644937
Escherichia coli	Dopamine	0.000100	gr	0.398923	0.450405	0.051482	0.000262	20.289367	3.000000	0.043407	0.059557	0.475168
Escherichia coli	Dopamine	0.000010	gr	0.398923	0.438986	0.040063	0.001625	10.961777	3.000000	0.028432	0.051694	0.369771
Escherichia coli	Dopamine	0.000001	gr	0.398923	0.421976	0.023053	0.002586	9.355554	3.000000	0.015211	0.030895	0.212778
Escherichia coli	Noradrenaline	0.100000	gr	0.414351	0.432052	0.017701	0.079300	2.615620	3.000000	-0.003836	0.039237	0.163372
Escherichia coli	Noradrenaline	0.010000	gr	0.414351	0.522697	0.108345	0.116738	2.185564	3.000000	-0.049419	0.266109	1.000000
Escherichia coli	Noradrenaline	0.001000	gr	0.414351	0.483976	0.069625	0.018979	4.629758	3.000000	0.021766	0.117484	0.642621
Escherichia coli	Noradrenaline	0.000100	gr	0.414351	0.450862	0.036511	0.034690	3.682786	3.000000	0.004960	0.068062	0.336989
Escherichia coli	Noradrenaline	0.000010	gr	0.414351	0.440975	0.026624	0.039360	3.504251	3.000000	0.002445	0.050804	0.245736
Escherichia coli	Noradrenaline	0.000001	gr	0.414351	0.438526	0.024175	0.013209	5.285618	3.000000	0.009619	0.038730	0.223126
Escherichia coli	Adrenaline	0.100000	gr	0.415051	0.432256	0.017205	0.260037	1.385142	3.000000	-0.022325	0.056736	0.158802
Escherichia coli	Adrenaline	0.010000	gr	0.415051	0.472827	0.057776	0.016408	4.884604	3.000000	0.020133	0.095418	0.533254
Escherichia coli	Adrenaline	0.001000	gr	0.415051	0.460783	0.045732	0.016422	4.883094	3.000000	0.015927	0.075537	0.422097
Escherichia coli	Adrenaline	0.000100	gr	0.415051	0.468027	0.052976	0.000086	29.485825	3.000000	0.047258	0.058693	0.488951
Escherichia coli	Adrenaline	0.000010	gr	0.415051	0.444622	0.029571	0.001188	12.191075	3.000000	0.021851	0.037290	0.272931
Escherichia coli	Adrenaline	0.000001	gr	0.415051	0.428975	0.013924	0.043721	3.360394	3.000000	0.000737	0.027110	0.128513
Escherichia coli	Levodopa	0.100000	dr	-0.015800	-0.008206	0.007594	0.595642	0.591763	3.000000	-0.033248	0.048437	0.176706
Escherichia coli	Levodopa	0.010000	dr	-0.015800	-0.020453	-0.004653	0.767619	-0.323409	3.000000	-0.050442	0.041136	-0.108269
Escherichia coli	Levodopa	0.001000	dr	-0.015800	-0.012989	0.002811	0.787595	0.294476	3.000000	-0.027568	0.033190	0.065406
Escherichia coli	Levodopa	0.000100	dr	-0.015800	-0.014133	0.001668	0.882930	0.160163	3.000000	-0.031468	0.034803	0.038801

Species	Hormon	Concentration	Parameter	Mean_Baseline	Mean_Treatment	Diff	P_Value	T_Statistic	DF	Lower_CI	Upper_CI	Diff_Norm
Escherichia coli	Levodopa	0.000010	dr	-0.015800	-0.007257	0.008544	0.497630	0.769505	3.000000	-0.026790	0.043877	0.198790
Escherichia coli	Levodopa	0.000001	dr	-0.015800	-0.016209	-0.000409	0.978147	-0.029733	3.000000	-0.044152	0.043335	-0.009509
Escherichia coli	Dopamine	0.100000	dr	-0.021008	-0.012324	0.008684	0.672013	0.467489	3.000000	-0.050433	0.067801	0.202057
Escherichia coli	Dopamine	0.010000	dr	-0.021008	-0.013768	0.007240	0.718009	0.396872	3.000000	-0.050817	0.065297	0.168459
Escherichia coli	Dopamine	0.001000	dr	-0.021008	-0.004913	0.016095	0.496341	0.772023	3.000000	-0.050252	0.082443	0.374497
Escherichia coli	Dopamine	0.000100	dr	-0.021008	-0.011656	0.009352	0.644653	0.510886	3.000000	-0.048904	0.067608	0.217598
Escherichia coli	Dopamine	0.000010	dr	-0.021008	-0.011010	0.009998	0.542398	0.685228	3.000000	-0.036438	0.056434	0.232638
Escherichia coli	Dopamine	0.000001	dr	-0.021008	-0.009892	0.011116	0.420142	0.931875	3.000000	-0.026845	0.049077	0.258635
Escherichia coli	Noradrenaline	0.100000	dr	-0.046126	-0.010308	0.035817	0.001066	12.648015	3.000000	0.026805	0.044830	0.833391
Escherichia coli	Noradrenaline	0.010000	dr	-0.046126	-0.027222	0.018904	0.231254	1.497212	3.000000	-0.021278	0.059085	0.439842
Escherichia coli	Noradrenaline	0.001000	dr	-0.046126	-0.015901	0.030225	0.002322	9.706581	3.000000	0.020315	0.040134	0.703262
Escherichia coli	Noradrenaline	0.000100	dr	-0.046126	-0.014127	0.031999	0.003381	8.533411	3.000000	0.020065	0.043932	0.744536
Escherichia coli	Noradrenaline	0.000010	dr	-0.046126	-0.010077	0.036048	0.003093	8.798490	3.000000	0.023010	0.049087	0.838766
Escherichia coli	Noradrenaline	0.000001	dr	-0.046126	-0.003148	0.042978	0.015814	4.950867	3.000000	0.015351	0.070605	1.000000
Escherichia coli	Adrenaline	0.100000	dr	-0.002153	-0.012122	-0.009969	0.136061	-2.024521	3.000000	-0.025641	0.005702	-0.231963
Escherichia coli	Adrenaline	0.010000	dr	-0.002153	-0.021850	-0.019697	0.042697	-3.392473	3.000000	-0.038174	-0.001219	-0.458296
Escherichia coli	Adrenaline	0.001000	dr	-0.002153	-0.021704	-0.019551	0.144603	-1.961768	3.000000	-0.051267	0.012165	-0.454906
Escherichia coli	Adrenaline	0.000100	dr	-0.002153	-0.014498	-0.012345	0.140969	-1.987914	3.000000	-0.032109	0.007418	-0.287245
Escherichia coli	Adrenaline	0.000010	dr	-0.002153	-0.016770	-0.014617	0.107910	-2.270150	3.000000	-0.035109	0.005874	-0.340113
Escherichia coli	Adrenaline	0.000001	dr	-0.002153	-0.009267	-0.007114	0.333839	-1.149048	3.000000	-0.026818	0.012589	-0.165529
Escherichia coli	Levodopa	0.100000	td	1.688731	1.817985	0.129254	0.054160	3.079438	3.000000	-0.004324	0.262831	0.409220
Escherichia coli	Levodopa	0.010000	td	1.688731	1.605739	-0.082992	0.431080	-0.907423	3.000000	-0.374055	0.208071	-0.262754
Escherichia coli	Levodopa	0.001000	td	1.688731	1.495979	-0.192752	0.022993	-4.310295	3.000000	-0.335068	-0.050436	-0.610257
Escherichia coli	Levodopa	0.000100	td	1.688731	1.459911	-0.228820	0.001424	-11.465751	3.000000	-0.292332	-0.165309	-0.724449
Escherichia coli	Levodopa	0.000010	td	1.688731	1.536816	-0.151915	0.027749	-4.014250	3.000000	-0.272352	-0.031479	-0.480966
Escherichia coli	Levodopa	0.000001	td	1.688731	1.851018	0.162287	0.519203	0.728162	3.000000	-0.546992	0.871566	0.513804
Escherichia coli	Dopamine	0.100000	td	1.737881	1.841651	0.103770	0.045504	3.306712	3.000000	0.003900	0.203640	0.328538

Species	Hormon	Concentration	Parameter	Mean_Baseline	Mean_Treatment	Diff	P_Value	T_Statistic	DF	Lower_CI	Upper_CI	Diff_Norm
Escherichia coli	Dopamine	0.010000	td	1.737881	1.604662	-0.133219	0.423839	-0.923546	3.000000	-0.592277	0.325840	-0.421773
Escherichia coli	Dopamine	0.001000	td	1.737881	1.481784	-0.256097	0.005534	-7.194487	3.000000	-0.369381	-0.142814	-0.810809
Escherichia coli	Dopamine	0.000100	td	1.737881	1.539141	-0.198740	0.000333	-18.713071	3.000000	-0.232539	-0.164941	-0.629214
Escherichia coli	Dopamine	0.000010	td	1.737881	1.579566	-0.158315	0.001342	-11.699084	3.000000	-0.201380	-0.115249	-0.501227
Escherichia coli	Dopamine	0.000001	td	1.737881	1.643024	-0.094857	0.002464	-9.511900	3.000000	-0.126593	-0.063120	-0.300318
Escherichia coli	Noradrenaline	0.100000	td	1.672911	1.605654	-0.067257	0.077091	-2.648531	3.000000	-0.148072	0.013558	-0.212937
Escherichia coli	Noradrenaline	0.010000	td	1.672911	1.357057	-0.315854	0.071305	-2.740561	3.000000	-0.682636	0.050928	-1.000000
Escherichia coli	Noradrenaline	0.001000	td	1.672911	1.436060	-0.236851	0.012196	-5.440015	3.000000	-0.375411	-0.098292	-0.749876
Escherichia coli	Noradrenaline	0.000100	td	1.672911	1.539350	-0.133561	0.030369	-3.877745	3.000000	-0.243173	-0.023948	-0.422855
Escherichia coli	Noradrenaline	0.000010	td	1.672911	1.573461	-0.099450	0.037137	-3.585655	3.000000	-0.187717	-0.011183	-0.314862
Escherichia coli	Noradrenaline	0.000001	td	1.672911	1.580954	-0.091956	0.012194	-5.440333	3.000000	-0.145749	-0.038164	-0.291136
Escherichia coli	Adrenaline	0.100000	td	1.670921	1.605029	-0.065892	0.262074	-1.377724	3.000000	-0.218098	0.086314	-0.208615
Escherichia coli	Adrenaline	0.010000	td	1.670921	1.469536	-0.201385	0.011762	-5.511340	3.000000	-0.317672	-0.085098	-0.637590
Escherichia coli	Adrenaline	0.001000	td	1.670921	1.505623	-0.165299	0.015549	-4.981522	3.000000	-0.270900	-0.059698	-0.523339
Escherichia coli	Adrenaline	0.000100	td	1.670921	1.481524	-0.189397	0.000212	-21.765333	3.000000	-0.217090	-0.161704	-0.599636
Escherichia coli	Adrenaline	0.000010	td	1.670921	1.559390	-0.111532	0.001964	-10.278022	3.000000	-0.146066	-0.076998	-0.353112
Escherichia coli	Adrenaline	0.000001	td	1.670921	1.616025	-0.054896	0.047288	-3.255628	3.000000	-0.108559	-0.001234	-0.173803
Escherichia coli	Levodopa	0.100000	lagC	2.123481	2.329835	0.206354	0.003798	8.198355	3.000000	0.126251	0.286456	0.805990
Escherichia coli	Levodopa	0.010000	lagC	2.123481	2.327850	0.204369	0.001642	10.925155	3.000000	0.144837	0.263901	0.798239
Escherichia coli	Levodopa	0.001000	lagC	2.123481	2.309099	0.185618	0.030293	3.881506	3.000000	0.033430	0.337806	0.724999
Escherichia coli	Levodopa	0.000100	lagC	2.123481	2.271433	0.147952	0.011675	5.526108	3.000000	0.062748	0.233157	0.577881
Escherichia coli	Levodopa	0.000010	lagC	2.123481	2.271488	0.148007	0.015258	5.015993	3.000000	0.054103	0.241912	0.578095
Escherichia coli	Levodopa	0.000001	lagC	2.123481	1.932539	-0.190942	0.566842	-0.641504	3.000000	-1.138189	0.756305	-0.745793
Escherichia coli	Dopamine	0.100000	lagC	1.936279	2.088978	0.152699	0.072958	2.713349	3.000000	-0.026399	0.331798	0.596423
Escherichia coli	Dopamine	0.010000	lagC	1.936279	2.073666	0.137387	0.130572	2.067357	3.000000	-0.074104	0.348878	0.536615
Escherichia coli	Dopamine	0.001000	lagC	1.936279	2.110499	0.174220	0.085724	2.525997	3.000000	-0.045276	0.393716	0.680479
Escherichia coli	Dopamine	0.000100	lagC	1.936279	2.032916	0.096637	0.092619	2.438565	3.000000	-0.029479	0.222752	0.377450

Species	Hormon	Concentration	Parameter	Mean_Baseline	Mean_Treatment	Diff	P_Value	T_Statistic	DF	Lower_CI	Upper_CI	Diff_Norm
Escherichia coli	Dopamine	0.000010	lagC	1.936279	1.994784	0.058505	0.055233	3.054481	3.000000	-0.002451	0.119462	0.228514
Escherichia coli	Dopamine	0.000001	lagC	1.936279	2.016779	0.080500	0.018436	4.679706	3.000000	0.025756	0.135244	0.314421
Escherichia coli	Noradrenaline	0.100000	lagC	2.069580	2.250957	0.181377	0.022377	4.354476	3.000000	0.048818	0.313935	0.708432
Escherichia coli	Noradrenaline	0.010000	lagC	2.069580	2.265984	0.196404	0.125964	2.105004	3.000000	-0.100529	0.493337	0.767127
Escherichia coli	Noradrenaline	0.001000	lagC	2.069580	2.242476	0.172896	0.060073	2.949002	3.000000	-0.013686	0.359478	0.675308
Escherichia coli	Noradrenaline	0.000100	lagC	2.069580	2.140673	0.071093	0.012472	5.396375	3.000000	0.029167	0.113019	0.277679
Escherichia coli	Noradrenaline	0.000010	lagC	2.069580	2.118983	0.049404	0.009343	5.983876	3.000000	0.023129	0.075678	0.192963
Escherichia coli	Noradrenaline	0.000001	lagC	2.069580	2.124506	0.054926	0.043002	3.382813	3.000000	0.003253	0.106599	0.214535
Escherichia coli	Adrenaline	0.100000	lagC	2.128819	2.384844	0.256025	0.037304	3.579349	3.000000	0.028390	0.483661	1.000000
Escherichia coli	Adrenaline	0.010000	lagC	2.128819	2.359403	0.230584	0.021294	4.435973	3.000000	0.065159	0.396010	0.900631
Escherichia coli	Adrenaline	0.001000	lagC	2.128819	2.319592	0.190774	0.084019	2.548965	3.000000	-0.047412	0.428960	0.745136
Escherichia coli	Adrenaline	0.000100	lagC	2.128819	2.313349	0.184530	0.093689	2.425702	3.000000	-0.057568	0.426629	0.720751
Escherichia coli	Adrenaline	0.000010	lagC	2.128819	2.278846	0.150027	0.191601	1.679732	3.000000	-0.134217	0.434271	0.585986
Escherichia coli	Adrenaline	0.000001	lagC	2.128819	2.250477	0.121658	0.202600	1.625147	3.000000	-0.116579	0.359896	0.475181
Escherichia coli	Levodopa	0.100000	t_k	37.000000	44.416667	7.416667	0.575147	0.626973	3.000000	-30.229517	45.062850	0.178000
Escherichia coli	Levodopa	0.010000	t_k	37.000000	36.750000	-0.250000	0.987896	-0.016467	3.000000	-48.565407	48.065407	-0.006000
Escherichia coli	Levodopa	0.001000	t_k	37.000000	19.916667	-17.083333	0.226655	-1.516523	3.000000	-52.932971	18.766304	-0.410000
Escherichia coli	Levodopa	0.000100	t_k	37.000000	10.750000	-26.250000	0.088289	-2.492502	3.000000	-59.766214	7.266214	-0.630000
Escherichia coli	Levodopa	0.000010	t_k	37.000000	29.750000	-7.250000	0.575616	-0.626159	3.000000	-44.098070	29.598070	-0.174000
Escherichia coli	Levodopa	0.000001	t_k	37.000000	43.583333	6.583333	0.590350	0.600771	3.000000	-28.290386	41.457053	0.158000
Escherichia coli	Dopamine	0.100000	t_k	16.333333	44.916667	28.583333	0.063365	2.883167	3.000000	-2.967010	60.133677	0.686000
Escherichia coli	Dopamine	0.010000	t_k	16.333333	35.583333	19.250000	0.149332	1.928866	3.000000	-12.510677	51.010677	0.462000
Escherichia coli	Dopamine	0.001000	t_k	16.333333	39.000000	22.666667	0.137648	2.012516	3.000000	-13.176757	58.510091	0.544000
Escherichia coli	Dopamine	0.000100	t_k	16.333333	43.916667	27.583333	0.067415	2.807808	3.000000	-3.680366	58.847033	0.662000
Escherichia coli	Dopamine	0.000010	t_k	16.333333	44.833333	28.500000	0.060059	2.949299	3.000000	-2.252977	59.252977	0.684000
Escherichia coli	Dopamine	0.000001	t_k	16.333333	47.583333	31.250000	0.057681	2.999744	3.000000	-1.903311	64.403311	0.750000
Escherichia coli	Noradrenaline	0.100000	t_k	5.750000	38.500000	32.750000	0.004263	7.877349	3.000000	19.519012	45.980988	0.786000

Species	Hormon	Concentration	Parameter	Mean_Baseline	Mean_Treatment	Diff	P_Value	T_Statistic	DF	Lower_CI	Upper_CI	Diff_Norm
Escherichia coli	Noradrenaline	0.010000	t_k	5.750000	30.166667	24.416667	0.055113	3.057229	3.000000	-1.000048	49.833382	0.586000
Escherichia coli	Noradrenaline	0.001000	t_k	5.750000	11.000000	5.250000	0.000756	14.206110	3.000000	4.073897	6.426103	0.126000
Escherichia coli	Noradrenaline	0.000100	t_k	5.750000	18.916667	13.166667	0.228579	1.508392	3.000000	-14.612725	40.946059	0.316000
Escherichia coli	Noradrenaline	0.000010	t_k	5.750000	39.916667	34.166667	0.002768	9.140498	3.000000	22.270862	46.062471	0.820000
Escherichia coli	Noradrenaline	0.000001	t_k	5.750000	47.416667	41.666667	0.000000	176.776695	3.000000	40.916557	42.416776	1.000000
Escherichia coli	Adrenaline	0.100000	t_k	47.833333	41.000000	-6.833333	0.377365	-1.033540	3.000000	-27.874325	14.207658	-0.164000
Escherichia coli	Adrenaline	0.010000	t_k	47.833333	34.333333	-13.500000	0.206170	-1.608143	3.000000	-40.215916	13.215916	-0.324000
Escherichia coli	Adrenaline	0.001000	t_k	47.833333	10.166667	-37.666667	0.000002	-104.617671	3.000000	-38.812478	-36.520855	-0.904000
Escherichia coli	Adrenaline	0.000100	t_k	47.833333	10.500000	-37.333333	0.000005	-76.089017	3.000000	-38.894811	-35.771855	-0.896000
Escherichia coli	Adrenaline	0.000010	t_k	47.833333	27.416667	-20.416667	0.101052	-2.341845	3.000000	-48.161858	7.328525	-0.490000
Escherichia coli	Adrenaline	0.000001	t_k	47.833333	43.583333	-4.250000	0.200336	-1.636106	3.000000	-12.516820	4.016820	-0.102000
Escherichia coli	Levodopa	0.100000	t_gr	3.250000	3.500000	0.250000	0.057669	3.000000	3.000000	-0.015204	0.515204	1.000000
Escherichia coli	Levodopa	0.010000	t_gr	3.250000	3.416667	0.166667	0.181690	1.732051	3.000000	-0.139564	0.472898	0.666667
Escherichia coli	Levodopa	0.001000	t_gr	3.250000	3.416667	0.166667	0.181690	1.732051	3.000000	-0.139564	0.472898	0.666667
Escherichia coli	Levodopa	0.000100	t_gr	3.250000	3.416667	0.166667	0.181690	1.732051	3.000000	-0.139564	0.472898	0.666667
Escherichia coli	Levodopa	0.000010	t_gr	3.250000	3.416667	0.166667	0.181690	1.732051	3.000000	-0.139564	0.472898	0.666667
Escherichia coli	Levodopa	0.000001	t_gr	3.250000	3.250000	-0.000000	1.000000	-0.000000	3.000000	-0.750110	0.750110	-0.000000
Escherichia coli	Dopamine	0.100000	t_gr	3.083333	3.333333	0.250000	0.057669	3.000000	3.000000	-0.015204	0.515204	1.000000
Escherichia coli	Dopamine	0.010000	t_gr	3.083333	3.250000	0.166667	0.181690	1.732051	3.000000	-0.139564	0.472898	0.666667
Escherichia coli	Dopamine	0.001000	t_gr	3.083333	3.166667	0.083333	0.391002	1.000000	3.000000	-0.181871	0.348537	0.333333
Escherichia coli	Dopamine	0.000100	t_gr	3.083333	3.250000	0.166667	0.181690	1.732051	3.000000	-0.139564	0.472898	0.666667
Escherichia coli	Dopamine	0.000010	t_gr	3.083333	3.250000	0.166667	0.181690	1.732051	3.000000	-0.139564	0.472898	0.666667
Escherichia coli	Dopamine	0.000001	t_gr	3.083333	3.250000	0.166667	0.181690	1.732051	3.000000	-0.139564	0.472898	0.666667
Escherichia coli	Noradrenaline	0.100000	t_gr	3.333333	3.416667	0.083333	0.391002	1.000000	3.000000	-0.181871	0.348537	0.333333
Escherichia coli	Noradrenaline	0.010000	t_gr	3.333333	3.333333	0.000000	NA	NA	3.000000	NA	NA	0.000000
Escherichia coli	Noradrenaline	0.001000	t_gr	3.333333	3.416667	0.083333	0.391002	1.000000	3.000000	-0.181871	0.348537	0.333333
Escherichia coli	Noradrenaline	0.000100	t_gr	3.333333	3.250000	-0.083333	0.391002	-1.000000	3.000000	-0.348537	0.181871	-0.333333

Species	Hormon	Concentration	Parameter	Mean_Baseline	Mean_Treatment	Diff	P_Value	T_Statistic	DF	Lower_CI	Upper_CI	Diff_Norm
Escherichia coli	Noradrenaline	0.000010	t_gr	3.333333	3.333333	0.000000	NA	NA	3.000000	NA	NA	0.000000
Escherichia coli	Noradrenaline	0.000001	t_gr	3.333333	3.333333	0.000000	NA	NA	3.000000	NA	NA	0.000000
Escherichia coli	Adrenaline	0.100000	t_gr	3.333333	3.500000	0.166667	0.181690	1.732051	3.000000	-0.139564	0.472898	0.666667
Escherichia coli	Adrenaline	0.010000	t_gr	3.333333	3.333333	0.000000	NA	NA	3.000000	NA	NA	0.000000
Escherichia coli	Adrenaline	0.001000	t_gr	3.333333	3.250000	-0.083333	0.391002	-1.000000	3.000000	-0.348537	0.181871	-0.333333
Escherichia coli	Adrenaline	0.000100	t_gr	3.333333	3.416667	0.083333	0.391002	1.000000	3.000000	-0.181871	0.348537	0.333333
Escherichia coli	Adrenaline	0.000010	t_gr	3.333333	3.500000	0.166667	0.181690	1.732051	3.000000	-0.139564	0.472898	0.666667
Escherichia coli	Adrenaline	0.000001	t_gr	3.333333	3.416667	0.083333	0.391002	1.000000	3.000000	-0.181871	0.348537	0.333333
Escherichia coli	Levodopa	0.100000	t_dr	38.250000	48.000000	9.750000	0.391002	1.000000	3.000000	-21.278851	40.778851	0.251073
Escherichia coli	Levodopa	0.010000	t_dr	38.250000	39.750000	1.500000	0.925276	0.101885	3.000000	-45.353349	48.353349	0.038627
Escherichia coli	Levodopa	0.001000	t_dr	38.250000	22.583333	-15.666667	0.237595	-1.471267	3.000000	-49.554698	18.221364	-0.403433
Escherichia coli	Levodopa	0.000100	t_dr	38.250000	13.250000	-25.000000	0.094454	-2.416632	3.000000	-57.922329	7.922329	-0.643777
Escherichia coli	Levodopa	0.000010	t_dr	38.250000	44.416667	6.166667	0.600015	0.584364	3.000000	-27.416981	39.750314	0.158798
Escherichia coli	Levodopa	0.000001	t_dr	38.250000	47.500000	9.250000	0.391002	1.000000	3.000000	-20.187628	38.687628	0.238197
Escherichia coli	Dopamine	0.100000	t_dr	28.666667	45.583333	16.916667	0.275443	1.330516	3.000000	-23.546119	57.379452	0.435622
Escherichia coli	Dopamine	0.010000	t_dr	28.666667	45.416667	16.750000	0.187382	1.701619	3.000000	-14.576627	48.076627	0.431330
Escherichia coli	Dopamine	0.001000	t_dr	28.666667	39.416667	10.750000	0.584046	0.611576	3.000000	-45.189577	66.689577	0.276824
Escherichia coli	Dopamine	0.000100	t_dr	28.666667	48.000000	19.333333	0.181785	1.731536	3.000000	-16.200025	54.866692	0.497854
Escherichia coli	Dopamine	0.000010	t_dr	28.666667	47.333333	18.666667	0.182602	1.727101	3.000000	-15.729500	53.062834	0.480687
Escherichia coli	Dopamine	0.000001	t_dr	28.666667	48.000000	19.333333	0.181785	1.731536	3.000000	-16.200025	54.866692	0.497854
Escherichia coli	Noradrenaline	0.100000	t_dr	9.166667	48.000000	38.833333	0.000001	134.522613	3.000000	37.914640	39.752026	1.000000
Escherichia coli	Noradrenaline	0.010000	t_dr	9.166667	37.250000	28.083333	0.038905	3.520426	3.000000	2.696152	53.470514	0.723176
Escherichia coli	Noradrenaline	0.001000	t_dr	9.166667	14.166667	5.000000	0.024832	4.187179	3.000000	1.199773	8.800227	0.128755
Escherichia coli	Noradrenaline	0.000100	t_dr	9.166667	29.750000	20.583333	0.125822	2.106193	3.000000	-10.517968	51.684635	0.530043
Escherichia coli	Noradrenaline	0.000010	t_dr	9.166667	47.500000	38.333333	0.000006	72.732386	3.000000	36.656037	40.010630	0.987124
Escherichia coli	Noradrenaline	0.000001	t_dr	9.166667	47.833333	38.666667	0.000000	164.048773	3.000000	37.916557	39.416776	0.995708
Escherichia coli	Adrenaline	0.100000	t_dr	48.000000	48.000000	0.000000	NA	NA	3.000000	NA	NA	0.000000

Species	Hormon	Concentration	Parameter	Mean_Baseline	Mean_Treatment	Diff	P_Value	T_Statistic	DF	Lower_CI	Upper_CI	Diff_Norm
Escherichia coli	Adrenaline	0.010000	t_dr	48.000000	48.000000	0.000000	NA	NA	3.000000	NA	NA	0.000000
Escherichia coli	Adrenaline	0.001000	t_dr	48.000000	22.583333	-25.416667	0.057746	-2.998324	3.000000	-52.394126	1.560793	-0.654506
Escherichia coli	Adrenaline	0.000100	t_dr	48.000000	21.333333	-26.666667	0.057707	-2.999180	3.000000	-54.962812	1.629479	-0.686695
Escherichia coli	Adrenaline	0.000010	t_dr	48.000000	38.333333	-9.666667	0.391002	-1.000000	3.000000	-40.430314	21.096981	-0.248927
Escherichia coli	Adrenaline	0.000001	t_dr	48.000000	48.000000	0.000000	NA	NA	3.000000	NA	NA	0.000000
Eubacterium ventriosum	Levodopa	0.100000	auc_lin	12.289812	16.476216	4.186404	0.043740	3.359781	3.000000	0.220966	8.151841	0.731228
Eubacterium ventriosum	Levodopa	0.010000	auc_lin	12.289812	15.317356	3.027544	0.101768	2.334084	3.000000	-1.100413	7.155500	0.528813
Eubacterium ventriosum	Levodopa	0.001000	auc_lin	12.289812	16.022086	3.732273	0.044739	3.329419	3.000000	0.164756	7.299791	0.651906
Eubacterium ventriosum	Levodopa	0.000100	auc_lin	12.289812	18.014979	5.725167	0.011557	5.546223	3.000000	2.440042	9.010292	1.000000
Eubacterium ventriosum	Levodopa	0.000010	auc_lin	12.289812	15.843752	3.553940	0.056999	3.014693	3.000000	-0.197759	7.305639	0.620757
Eubacterium ventriosum	Levodopa	0.000001	auc_lin	12.289812	15.182140	2.892327	0.017207	4.800080	3.000000	0.974718	4.809936	0.505195
Eubacterium ventriosum	Dopamine	0.100000	auc_lin	11.922257	16.464901	4.542644	0.011626	5.534438	3.000000	1.930505	7.154783	0.793452
Eubacterium ventriosum	Dopamine	0.010000	auc_lin	11.922257	16.149966	4.227709	0.013047	5.309200	3.000000	1.693531	6.761887	0.738443
Eubacterium ventriosum	Dopamine	0.001000	auc_lin	11.922257	16.879671	4.957413	0.005351	7.279319	3.000000	2.790081	7.124746	0.865899
Eubacterium ventriosum	Dopamine	0.000100	auc_lin	11.922257	16.931521	5.009264	0.025584	4.140193	3.000000	1.158788	8.859740	0.874955
Eubacterium ventriosum	Dopamine	0.000010	auc_lin	11.922257	16.816523	4.894266	0.000525	16.063136	3.000000	3.924608	5.863923	0.854869
Eubacterium ventriosum	Dopamine	0.000001	auc_lin	11.922257	15.924747	4.002490	0.012385	5.409952	3.000000	1.647994	6.356986	0.699105
Eubacterium ventriosum	Noradrenaline	0.100000	auc_lin	14.742620	17.070250	2.327630	0.079563	2.611775	3.000000	-0.508586	5.163846	0.406561
Eubacterium ventriosum	Noradrenaline	0.010000	auc_lin	14.742620	15.838885	1.096265	0.389990	1.002451	3.000000	-2.384011	4.576541	0.191482
Eubacterium ventriosum	Noradrenaline	0.001000	auc_lin	14.742620	15.760527	1.017907	0.342433	1.125110	3.000000	-1.861310	3.897123	0.177795
Eubacterium ventriosum	Noradrenaline	0.000100	auc_lin	14.742620	16.691046	1.948426	0.129785	2.073673	3.000000	-1.041805	4.938657	0.340326
Eubacterium ventriosum	Noradrenaline	0.000010	auc_lin	14.742620	15.481579	0.738959	0.647111	0.506939	3.000000	-3.900055	5.377974	0.129072
Eubacterium ventriosum	Noradrenaline	0.000001	auc_lin	14.742620	15.067615	0.324995	0.774138	0.313929	3.000000	-2.969634	3.619624	0.056766
Eubacterium ventriosum	Adrenaline	0.100000	auc_lin	12.622768	15.745984	3.123216	0.015219	5.020679	3.000000	1.143510	5.102922	0.545524
Eubacterium ventriosum	Adrenaline	0.010000	auc_lin	12.622768	17.138948	4.516180	0.018071	4.714317	3.000000	1.467488	7.564872	0.788829
Eubacterium ventriosum	Adrenaline	0.001000	auc_lin	12.622768	16.215853	3.593085	0.009500	5.948485	3.000000	1.670780	5.515390	0.627595
Eubacterium ventriosum	Adrenaline	0.000100	auc_lin	12.622768	16.531318	3.908549	0.006089	6.958458	3.000000	2.120977	5.696122	0.682696

Species	Hormon	Concentration	Parameter	Mean_Baseline	Mean_Treatment	Diff	P_Value	T_Statistic	DF	Lower_CI	Upper_CI	Diff_Norm
Eubacterium ventriosum	Adrenaline	0.000010	auc_lin	12.622768	16.141769	3.519001	0.007215	6.556413	3.000000	1.810897	5.227104	0.614655
Eubacterium ventriosum	Adrenaline	0.000001	auc_lin	12.622768	15.317051	2.694283	0.010508	5.738505	3.000000	1.200094	4.188472	0.470603
Eubacterium ventriosum	Levodopa	0.100000	k_lin	0.338623	0.454407	0.115784	0.093651	2.426166	3.000000	-0.036092	0.267660	0.786414
Eubacterium ventriosum	Levodopa	0.010000	k_lin	0.338623	0.397813	0.059190	0.286134	1.294476	3.000000	-0.086327	0.204707	0.402023
Eubacterium ventriosum	Levodopa	0.001000	k_lin	0.338623	0.410166	0.071543	0.170605	1.794574	3.000000	-0.055329	0.198415	0.485925
Eubacterium ventriosum	Levodopa	0.000100	k_lin	0.338623	0.485853	0.147230	0.036612	3.605807	3.000000	0.017286	0.277174	1.000000
Eubacterium ventriosum	Levodopa	0.000010	k_lin	0.338623	0.418770	0.080147	0.183898	1.720119	3.000000	-0.068136	0.228430	0.544366
Eubacterium ventriosum	Levodopa	0.000001	k_lin	0.338623	0.408210	0.069587	0.134677	2.035129	3.000000	-0.039230	0.178405	0.472643
Eubacterium ventriosum	Dopamine	0.100000	k_lin	0.359554	0.448710	0.089156	0.012389	5.409355	3.000000	0.036703	0.141608	0.605555
Eubacterium ventriosum	Dopamine	0.010000	k_lin	0.359554	0.426791	0.067237	0.138779	2.004063	3.000000	-0.039535	0.174010	0.456682
Eubacterium ventriosum	Dopamine	0.001000	k_lin	0.359554	0.428605	0.069051	0.022707	4.330628	3.000000	0.018308	0.119795	0.469001
Eubacterium ventriosum	Dopamine	0.000100	k_lin	0.359554	0.439387	0.079833	0.100066	2.352634	3.000000	-0.028158	0.187823	0.542230
Eubacterium ventriosum	Dopamine	0.000010	k_lin	0.359554	0.456392	0.096838	0.000177	23.121170	3.000000	0.083509	0.110167	0.657730
Eubacterium ventriosum	Dopamine	0.000001	k_lin	0.359554	0.456047	0.096493	0.009713	5.901726	3.000000	0.044460	0.148526	0.655390
Eubacterium ventriosum	Noradrenaline	0.100000	k_lin	0.419714	0.472230	0.052516	0.036212	3.621395	3.000000	0.006366	0.098667	0.356696
Eubacterium ventriosum	Noradrenaline	0.010000	k_lin	0.419714	0.402642	-0.017072	0.588934	-0.603190	3.000000	-0.107146	0.073001	-0.115956
Eubacterium ventriosum	Noradrenaline	0.001000	k_lin	0.419714	0.398141	-0.021572	0.427840	-0.914605	3.000000	-0.096636	0.053491	-0.146522
Eubacterium ventriosum	Noradrenaline	0.000100	k_lin	0.419714	0.428821	0.009107	0.675628	0.461838	3.000000	-0.053651	0.071866	0.061859
Eubacterium ventriosum	Noradrenaline	0.000010	k_lin	0.419714	0.404022	-0.015692	0.695909	-0.430465	3.000000	-0.131702	0.100318	-0.106580
Eubacterium ventriosum	Noradrenaline	0.000001	k_lin	0.419714	0.404850	-0.014864	0.690812	-0.438298	3.000000	-0.122787	0.093060	-0.100955
Eubacterium ventriosum	Adrenaline	0.100000	k_lin	0.367698	0.402295	0.034596	0.216476	1.560845	3.000000	-0.035943	0.105135	0.234980
Eubacterium ventriosum	Adrenaline	0.010000	k_lin	0.367698	0.437322	0.069623	0.087309	2.505158	3.000000	-0.018823	0.158070	0.472888
Eubacterium ventriosum	Adrenaline	0.001000	k_lin	0.367698	0.406976	0.039278	0.110844	2.241146	3.000000	-0.016497	0.095052	0.266777
Eubacterium ventriosum	Adrenaline	0.000100	k_lin	0.367698	0.420748	0.053050	0.055674	3.044384	3.000000	-0.002406	0.108506	0.360320
Eubacterium ventriosum	Adrenaline	0.000010	k_lin	0.367698	0.416645	0.048946	0.065797	2.837211	3.000000	-0.005956	0.103848	0.332447
Eubacterium ventriosum	Adrenaline	0.000001	k_lin	0.367698	0.423907	0.056208	0.038835	3.522956	3.000000	0.005433	0.106984	0.381773
Eubacterium ventriosum	Levodopa	0.100000	death_lin	0.010095	0.014646	0.004551	0.785210	0.297914	3.000000	-0.044066	0.053168	0.214308

Species	Hormon	Concentration	Parameter	Mean_Baseline	Mean_Treatment	Diff	P_Value	T_Statistic	DF	Lower_CI	Upper_CI	Diff_Norm
Eubacterium ventriosum	Levodopa	0.010000	death_lin	0.010095	0.009263	-0.000831	0.939115	-0.082952	3.000000	-0.032726	0.031064	-0.039148
Eubacterium ventriosum	Levodopa	0.001000	death_lin	0.010095	0.011114	0.001019	0.878330	0.166531	3.000000	-0.018461	0.020500	0.048002
Eubacterium ventriosum	Levodopa	0.000100	death_lin	0.010095	0.031331	0.021236	0.209328	1.593375	3.000000	-0.021179	0.063652	1.000000
Eubacterium ventriosum	Levodopa	0.000010	death_lin	0.010095	0.013817	0.003722	0.828595	0.236045	3.000000	-0.046460	0.053905	0.175269
Eubacterium ventriosum	Levodopa	0.000001	death_lin	0.010095	0.012315	0.002220	0.625179	0.542503	3.000000	-0.010804	0.015245	0.104548
Eubacterium ventriosum	Dopamine	0.100000	death_lin	0.005198	0.022579	0.017380	0.063798	2.874829	3.000000	-0.001860	0.036621	0.818428
Eubacterium ventriosum	Dopamine	0.010000	death_lin	0.005198	0.026202	0.021004	0.213517	1.574164	3.000000	-0.021459	0.063467	0.989058
Eubacterium ventriosum	Dopamine	0.001000	death_lin	0.005198	0.020779	0.015581	0.106984	2.279505	3.000000	-0.006172	0.037334	0.733695
Eubacterium ventriosum	Dopamine	0.000100	death_lin	0.005198	0.016348	0.011150	0.378580	1.030505	3.000000	-0.023284	0.045584	0.525047
Eubacterium ventriosum	Dopamine	0.000010	death_lin	0.005198	0.018549	0.013351	0.181836	1.731255	3.000000	-0.011191	0.037893	0.628676
Eubacterium ventriosum	Dopamine	0.000001	death_lin	0.005198	0.020509	0.015311	0.087882	2.497734	3.000000	-0.004197	0.034819	0.720968
Eubacterium ventriosum	Noradrenaline	0.100000	death_lin	0.017305	0.021658	0.004352	0.639691	0.518881	3.000000	-0.022342	0.031046	0.204947
Eubacterium ventriosum	Noradrenaline	0.010000	death_lin	0.017305	0.006903	-0.010402	0.547285	-0.676368	3.000000	-0.059346	0.038542	-0.489822
Eubacterium ventriosum	Noradrenaline	0.001000	death_lin	0.017305	0.008757	-0.008548	0.620384	-0.550389	3.000000	-0.057976	0.040879	-0.402531
Eubacterium ventriosum	Noradrenaline	0.000100	death_lin	0.017305	0.009126	-0.008179	0.407728	-0.960343	3.000000	-0.035284	0.018926	-0.385153
Eubacterium ventriosum	Noradrenaline	0.000010	death_lin	0.017305	0.017344	0.000038	0.998149	0.002518	3.000000	-0.048487	0.048564	0.001808
Eubacterium ventriosum	Noradrenaline	0.000001	death_lin	0.017305	0.015397	-0.001908	0.899927	-0.136698	3.000000	-0.046339	0.042522	-0.089867
Eubacterium ventriosum	Adrenaline	0.100000	death_lin	0.004959	0.001463	-0.003496	0.485228	-0.793949	3.000000	-0.017508	0.010516	-0.164611
Eubacterium ventriosum	Adrenaline	0.010000	death_lin	0.004959	0.009488	0.004529	0.390534	1.001133	3.000000	-0.009868	0.018926	0.213266
Eubacterium ventriosum	Adrenaline	0.001000	death_lin	0.004959	0.004228	-0.000731	0.913807	-0.117612	3.000000	-0.020515	0.019053	-0.034429
Eubacterium ventriosum	Adrenaline	0.000100	death_lin	0.004959	0.012232	0.007273	0.008904	6.086934	3.000000	0.003470	0.011076	0.342482
Eubacterium ventriosum	Adrenaline	0.000010	death_lin	0.004959	0.015187	0.010228	0.166698	1.817732	3.000000	-0.007679	0.028134	0.481608
Eubacterium ventriosum	Adrenaline	0.000001	death_lin	0.004959	0.023041	0.018082	0.221309	1.539519	3.000000	-0.019296	0.055460	0.851454
Eubacterium ventriosum	Levodopa	0.100000	gr	0.464555	0.402448	-0.062106	0.004741	-7.592582	3.000000	-0.088139	-0.036074	-0.729917
Eubacterium ventriosum	Levodopa	0.010000	gr	0.464555	0.435534	-0.029021	0.103215	-2.318610	3.000000	-0.068854	0.010812	-0.341072
Eubacterium ventriosum	Levodopa	0.001000	gr	0.464555	0.505591	0.041036	0.145399	1.956141	3.000000	-0.025726	0.107798	0.482283
Eubacterium ventriosum	Levodopa	0.000100	gr	0.464555	0.512329	0.047774	0.027207	4.044559	3.000000	0.010183	0.085365	0.561473

Species	Hormon	Concentration	Parameter	Mean_Baseline	Mean_Treatment	Diff	P_Value	T_Statistic	DF	Lower_CI	Upper_CI	Diff_Norm
Eubacterium ventriosum	Levodopa	0.000010	gr	0.464555	0.503026	0.038472	0.001469	11.346215	3.000000	0.027681	0.049263	0.452147
Eubacterium ventriosum	Levodopa	0.000001	gr	0.464555	0.498760	0.034205	0.036493	3.610409	3.000000	0.004055	0.064356	0.402001
Eubacterium ventriosum	Dopamine	0.100000	gr	0.449733	0.468901	0.019168	0.493805	0.776989	3.000000	-0.059343	0.097680	0.225280
Eubacterium ventriosum	Dopamine	0.010000	gr	0.449733	0.474581	0.024848	0.162965	1.840451	3.000000	-0.018119	0.067815	0.292034
Eubacterium ventriosum	Dopamine	0.001000	gr	0.449733	0.534820	0.085087	0.010036	5.833345	3.000000	0.038667	0.131507	1.000000
Eubacterium ventriosum	Dopamine	0.000100	gr	0.449733	0.523325	0.073592	0.000315	19.062987	3.000000	0.061307	0.085878	0.864905
Eubacterium ventriosum	Dopamine	0.000010	gr	0.449733	0.523177	0.073445	0.025842	4.124459	3.000000	0.016774	0.130115	0.863169
Eubacterium ventriosum	Dopamine	0.000001	gr	0.449733	0.482161	0.032428	0.029680	3.912152	3.000000	0.006049	0.058807	0.381115
Eubacterium ventriosum	Noradrenaline	0.100000	gr	0.466726	0.456780	-0.009946	0.152997	-1.904200	3.000000	-0.026569	0.006677	-0.116896
Eubacterium ventriosum	Noradrenaline	0.010000	gr	0.466726	0.507255	0.040528	0.033677	3.725581	3.000000	0.005908	0.075148	0.476317
Eubacterium ventriosum	Noradrenaline	0.001000	gr	0.466726	0.532940	0.066213	0.016297	4.896711	3.000000	0.023180	0.109247	0.778185
Eubacterium ventriosum	Noradrenaline	0.000100	gr	0.466726	0.509575	0.042849	0.026555	4.082074	3.000000	0.009443	0.076255	0.503591
Eubacterium ventriosum	Noradrenaline	0.000010	gr	0.466726	0.522787	0.056061	0.001269	11.921540	3.000000	0.041096	0.071027	0.658869
Eubacterium ventriosum	Noradrenaline	0.000001	gr	0.466726	0.502696	0.035970	0.002961	8.931010	3.000000	0.023152	0.048787	0.422739
Eubacterium ventriosum	Adrenaline	0.100000	gr	0.456624	0.471056	0.014432	0.013583	5.232558	3.000000	0.005655	0.023210	0.169620
Eubacterium ventriosum	Adrenaline	0.010000	gr	0.456624	0.506490	0.049866	0.371730	1.047738	3.000000	-0.101599	0.201331	0.586058
Eubacterium ventriosum	Adrenaline	0.001000	gr	0.456624	0.499691	0.043068	0.000834	13.743470	3.000000	0.033095	0.053040	0.506159
Eubacterium ventriosum	Adrenaline	0.000100	gr	0.456624	0.506391	0.049767	0.001969	10.269171	3.000000	0.034344	0.065190	0.584891
Eubacterium ventriosum	Adrenaline	0.000010	gr	0.456624	0.520491	0.063867	0.007463	6.479001	3.000000	0.032496	0.095239	0.750611
Eubacterium ventriosum	Adrenaline	0.000001	gr	0.456624	0.493560	0.036936	0.043487	3.367641	3.000000	0.002031	0.071841	0.434097
Eubacterium ventriosum	Levodopa	0.100000	dr	-0.013308	-0.025732	-0.012424	0.529541	-0.708843	3.000000	-0.068205	0.043357	-0.491318
Eubacterium ventriosum	Levodopa	0.010000	dr	-0.013308	-0.019290	-0.005982	0.678982	-0.456611	3.000000	-0.047677	0.035713	-0.236568
Eubacterium ventriosum	Levodopa	0.001000	dr	-0.013308	-0.014309	-0.001001	0.882076	-0.161344	3.000000	-0.020746	0.018744	-0.039585
Eubacterium ventriosum	Levodopa	0.000100	dr	-0.013308	-0.015728	-0.002420	0.789438	-0.291824	3.000000	-0.028810	0.023970	-0.095694
Eubacterium ventriosum	Levodopa	0.000010	dr	-0.013308	-0.009984	0.003324	0.557342	0.658324	3.000000	-0.012743	0.019391	0.131433
Eubacterium ventriosum	Levodopa	0.000001	dr	-0.013308	-0.011280	0.002028	0.785975	0.296810	3.000000	-0.019714	0.023770	0.080187
Eubacterium ventriosum	Dopamine	0.100000	dr	-0.000607	-0.012718	-0.012111	0.068270	-2.792618	3.000000	-0.025913	0.001691	-0.478926

Species	Hormon	Concentration	Parameter	Mean_Baseline	Mean_Treatment	Diff	P_Value	T_Statistic	DF	Lower_CI	Upper_CI	Diff_Norm
Eubacterium ventriosum	Dopamine	0.010000	dr	-0.000607	-0.025895	-0.025288	0.244684	-1.443144	3.000000	-0.081053	0.030477	-1.000000
Eubacterium ventriosum	Dopamine	0.001000	dr	-0.000607	-0.013320	-0.012713	0.062533	-2.899391	3.000000	-0.026668	0.001241	-0.502740
Eubacterium ventriosum	Dopamine	0.000100	dr	-0.000607	-0.010016	-0.009409	0.219883	-1.545757	3.000000	-0.028779	0.009962	-0.372059
Eubacterium ventriosum	Dopamine	0.000010	dr	-0.000607	-0.008739	-0.008132	0.145077	-1.958414	3.000000	-0.021346	0.005082	-0.321567
Eubacterium ventriosum	Dopamine	0.000001	dr	-0.000607	-0.020111	-0.019504	0.255055	-1.403548	3.000000	-0.063729	0.024720	-0.771289
Eubacterium ventriosum	Noradrenaline	0.100000	dr	-0.010140	-0.012670	-0.002531	0.665307	-0.478021	3.000000	-0.019378	0.014317	-0.100072
Eubacterium ventriosum	Noradrenaline	0.010000	dr	-0.010140	-0.013273	-0.003134	0.514539	-0.736981	3.000000	-0.016666	0.010398	-0.123922
Eubacterium ventriosum	Noradrenaline	0.001000	dr	-0.010140	-0.011848	-0.001708	0.566328	-0.642409	3.000000	-0.010168	0.006753	-0.067537
Eubacterium ventriosum	Noradrenaline	0.000100	dr	-0.010140	-0.010152	-0.000012	0.997624	-0.003232	3.000000	-0.011841	0.011817	-0.000475
Eubacterium ventriosum	Noradrenaline	0.000010	dr	-0.010140	-0.008878	0.001262	0.790342	0.290523	3.000000	-0.012560	0.015083	0.049895
Eubacterium ventriosum	Noradrenaline	0.000001	dr	-0.010140	-0.010189	-0.000049	0.986436	-0.018453	3.000000	-0.008477	0.008379	-0.001932
Eubacterium ventriosum	Adrenaline	0.100000	dr	-0.006532	-0.010819	-0.004286	0.422728	-0.926040	3.000000	-0.019017	0.010444	-0.169507
Eubacterium ventriosum	Adrenaline	0.010000	dr	-0.006532	-0.017671	-0.011139	0.023160	-4.298628	3.000000	-0.019385	-0.002892	-0.440477
Eubacterium ventriosum	Adrenaline	0.001000	dr	-0.006532	-0.010368	-0.003835	0.258224	-1.391795	3.000000	-0.012605	0.004934	-0.151666
Eubacterium ventriosum	Adrenaline	0.000100	dr	-0.006532	-0.012563	-0.006030	0.028879	-3.953442	3.000000	-0.010885	-0.001176	-0.238467
Eubacterium ventriosum	Adrenaline	0.000010	dr	-0.006532	-0.010683	-0.004151	0.244567	-1.443601	3.000000	-0.013301	0.005000	-0.164139
Eubacterium ventriosum	Adrenaline	0.000001	dr	-0.006532	-0.012104	-0.005572	0.123408	-2.126598	3.000000	-0.013910	0.002766	-0.220334
Eubacterium ventriosum	Levodopa	0.100000	td	1.493003	1.725947	0.232944	0.008440	6.203464	3.000000	0.113441	0.352448	0.958729
Eubacterium ventriosum	Levodopa	0.010000	td	1.493003	1.593284	0.100281	0.103455	2.316073	3.000000	-0.037512	0.238075	0.412727
Eubacterium ventriosum	Levodopa	0.001000	td	1.493003	1.379574	-0.113428	0.141557	-1.983630	3.000000	-0.295408	0.068551	-0.466837
Eubacterium ventriosum	Levodopa	0.000100	td	1.493003	1.357419	-0.135584	0.019464	-4.586671	3.000000	-0.229658	-0.041509	-0.558021
Eubacterium ventriosum	Levodopa	0.000010	td	1.493003	1.379418	-0.113585	0.000621	-15.180527	3.000000	-0.137397	-0.089773	-0.467480
Eubacterium ventriosum	Levodopa	0.000001	td	1.493003	1.390153	-0.102849	0.037785	-3.561291	3.000000	-0.194758	-0.010941	-0.423296
Eubacterium ventriosum	Dopamine	0.100000	td	1.541339	1.490942	-0.050397	0.522603	-0.721775	3.000000	-0.272607	0.171813	-0.207418
Eubacterium ventriosum	Dopamine	0.010000	td	1.541339	1.463346	-0.077993	0.152800	-1.905511	3.000000	-0.208252	0.052265	-0.320996
Eubacterium ventriosum	Dopamine	0.001000	td	1.541339	1.298367	-0.242972	0.007273	-6.538108	3.000000	-0.361240	-0.124705	-1.000000
Eubacterium ventriosum	Dopamine	0.000100	td	1.541339	1.324958	-0.216381	0.000145	-24.742132	3.000000	-0.244213	-0.188549	-0.890559

Species	Hormon	Concentration	Parameter	Mean_Baseline	Mean_Treatment	Diff	P_Value	T_Statistic	DF	Lower_CI	Upper_CI	Diff_Norm
Eubacterium ventriosum	Dopamine	0.000010	td	1.541339	1.329054	-0.212285	0.017138	-4.807206	3.000000	-0.352822	-0.071749	-0.873702
Eubacterium ventriosum	Dopamine	0.000001	td	1.541339	1.439273	-0.102066	0.023709	-4.260932	3.000000	-0.178298	-0.025834	-0.420072
Eubacterium ventriosum	Noradrenaline	0.100000	td	1.486840	1.517946	0.031107	0.135887	2.025850	3.000000	-0.017759	0.079973	0.128026
Eubacterium ventriosum	Noradrenaline	0.010000	td	1.486840	1.367038	-0.119801	0.034191	-3.703688	3.000000	-0.222742	-0.016860	-0.493066
Eubacterium ventriosum	Noradrenaline	0.001000	td	1.486840	1.301444	-0.185395	0.016505	-4.874067	3.000000	-0.306446	-0.064344	-0.763031
Eubacterium ventriosum	Noradrenaline	0.000100	td	1.486840	1.361507	-0.125333	0.026100	-4.108931	3.000000	-0.222406	-0.028260	-0.515833
Eubacterium ventriosum	Noradrenaline	0.000010	td	1.486840	1.326184	-0.160656	0.002536	-9.418821	3.000000	-0.214938	-0.106373	-0.661211
Eubacterium ventriosum	Noradrenaline	0.000001	td	1.486840	1.380649	-0.106190	0.002833	-9.068104	3.000000	-0.143458	-0.068923	-0.437047
Eubacterium ventriosum	Adrenaline	0.100000	td	1.518542	1.472406	-0.046136	0.011631	-5.533474	3.000000	-0.072670	-0.019602	-0.189881
Eubacterium ventriosum	Adrenaline	0.010000	td	1.518542	1.404350	-0.114192	0.428969	-0.912097	3.000000	-0.512624	0.284241	-0.469979
Eubacterium ventriosum	Adrenaline	0.001000	td	1.518542	1.387235	-0.131307	0.001488	-11.294674	3.000000	-0.168305	-0.094309	-0.540420
Eubacterium ventriosum	Adrenaline	0.000100	td	1.518542	1.370382	-0.148160	0.000780	-14.054853	3.000000	-0.181708	-0.114612	-0.609781
Eubacterium ventriosum	Adrenaline	0.000010	td	1.518542	1.332296	-0.186245	0.007439	-6.486202	3.000000	-0.277626	-0.094864	-0.766529
Eubacterium ventriosum	Adrenaline	0.000001	td	1.518542	1.404990	-0.113551	0.042872	-3.386929	3.000000	-0.220247	-0.006856	-0.467343
Eubacterium ventriosum	Levodopa	0.100000	lagC	2.611256	2.822834	0.211577	0.016000	4.929773	3.000000	0.074992	0.348163	0.856430
Eubacterium ventriosum	Levodopa	0.010000	lagC	2.611256	2.706307	0.095051	0.233068	1.489711	3.000000	-0.108005	0.298106	0.384750
Eubacterium ventriosum	Levodopa	0.001000	lagC	2.611256	2.765234	0.153977	0.025009	4.175990	3.000000	0.036634	0.271321	0.623274
Eubacterium ventriosum	Levodopa	0.000100	lagC	2.611256	2.737574	0.126318	0.043568	3.365105	3.000000	0.006857	0.245779	0.511314
Eubacterium ventriosum	Levodopa	0.000010	lagC	2.611256	2.777999	0.166743	0.029874	3.902343	3.000000	0.030760	0.302725	0.674947
Eubacterium ventriosum	Levodopa	0.000001	lagC	2.611256	2.727733	0.116476	0.058774	2.976238	3.000000	-0.008070	0.241023	0.471477
Eubacterium ventriosum	Dopamine	0.100000	lagC	2.426653	2.596433	0.169781	0.088669	2.487634	3.000000	-0.047421	0.386982	0.687243
Eubacterium ventriosum	Dopamine	0.010000	lagC	2.426653	2.455516	0.028864	0.545680	0.679271	3.000000	-0.106366	0.164093	0.116836
Eubacterium ventriosum	Dopamine	0.001000	lagC	2.426653	2.548476	0.121823	0.067871	2.799682	3.000000	-0.016655	0.260302	0.493120
Eubacterium ventriosum	Dopamine	0.000100	lagC	2.426653	2.480886	0.054233	0.391004	0.999997	3.000000	-0.118361	0.226827	0.219526
Eubacterium ventriosum	Dopamine	0.000010	lagC	2.426653	2.503443	0.076791	0.012511	5.390250	3.000000	0.031453	0.122128	0.310836
Eubacterium ventriosum	Dopamine	0.000001	lagC	2.426653	2.475872	0.049219	0.106259	2.286894	3.000000	-0.019274	0.117713	0.199231
Eubacterium ventriosum	Noradrenaline	0.100000	lagC	2.505996	2.667132	0.161136	0.012018	5.468857	3.000000	0.067367	0.254904	0.652250

Species	Hormon	Concentration	Parameter	Mean_Baseline	Mean_Treatment	Diff	P_Value	T_Statistic	DF	Lower_CI	Upper_CI	Diff_Norm
Eubacterium ventriosum	Noradrenaline	0.010000	lagC	2.505996	2.606173	0.100177	0.006299	6.876562	3.000000	0.053815	0.146538	0.405498
Eubacterium ventriosum	Noradrenaline	0.001000	lagC	2.505996	2.689559	0.183562	0.004295	7.856682	3.000000	0.109208	0.257916	0.743029
Eubacterium ventriosum	Noradrenaline	0.000100	lagC	2.505996	2.680297	0.174300	0.003774	8.215804	3.000000	0.106784	0.241817	0.705538
Eubacterium ventriosum	Noradrenaline	0.000010	lagC	2.505996	2.632976	0.126979	0.000174	23.277794	3.000000	0.109619	0.144339	0.513991
Eubacterium ventriosum	Noradrenaline	0.000001	lagC	2.505996	2.605907	0.099911	0.039791	3.489153	3.000000	0.008782	0.191039	0.404421
Eubacterium ventriosum	Adrenaline	0.100000	lagC	2.534735	2.748862	0.214127	0.002397	9.602371	3.000000	0.143160	0.285094	0.866750
Eubacterium ventriosum	Adrenaline	0.010000	lagC	2.534735	2.663526	0.128792	0.124731	2.115356	3.000000	-0.064969	0.322552	0.521326
Eubacterium ventriosum	Adrenaline	0.001000	lagC	2.534735	2.682651	0.147916	0.000731	14.363272	3.000000	0.115143	0.180690	0.598741
Eubacterium ventriosum	Adrenaline	0.000100	lagC	2.534735	2.715559	0.180824	0.004648	7.644769	3.000000	0.105549	0.256100	0.731947
Eubacterium ventriosum	Adrenaline	0.000010	lagC	2.534735	2.781780	0.247046	0.060429	2.941656	3.000000	-0.020222	0.514314	1.000000
Eubacterium ventriosum	Adrenaline	0.000001	lagC	2.534735	2.659126	0.124392	0.034455	3.692580	3.000000	0.017185	0.231599	0.503517
Eubacterium ventriosum	Levodopa	0.100000	t_k	32.583333	37.750000	5.166667	0.691017	0.437983	3.000000	-32.375061	42.708394	0.306931
Eubacterium ventriosum	Levodopa	0.010000	t_k	32.583333	30.416667	-2.166667	0.836897	-0.224349	3.000000	-32.901342	28.568008	-0.128713
Eubacterium ventriosum	Levodopa	0.001000	t_k	32.583333	32.666667	0.083333	0.994671	0.007249	3.000000	-36.501665	36.668331	0.004950
Eubacterium ventriosum	Levodopa	0.000100	t_k	32.583333	29.416667	-3.166667	0.822214	-0.245064	3.000000	-44.289516	37.956183	-0.188119
Eubacterium ventriosum	Levodopa	0.000010	t_k	32.583333	32.000000	-0.583333	0.965718	-0.046659	3.000000	-40.370689	39.204022	-0.034653
Eubacterium ventriosum	Levodopa	0.000001	t_k	32.583333	31.000000	-1.583333	0.906832	-0.127196	3.000000	-41.198257	38.031590	-0.094059
Eubacterium ventriosum	Dopamine	0.100000	t_k	38.583333	36.083333	-2.500000	0.526322	-0.714826	3.000000	-13.630140	8.630140	-0.148515
Eubacterium ventriosum	Dopamine	0.010000	t_k	38.583333	30.666667	-7.916667	0.113157	-2.218924	3.000000	-19.270985	3.437652	-0.470297
Eubacterium ventriosum	Dopamine	0.001000	t_k	38.583333	27.166667	-11.416667	0.054350	-3.074966	3.000000	-23.232384	0.399051	-0.678218
Eubacterium ventriosum	Dopamine	0.000100	t_k	38.583333	29.416667	-9.166667	0.308101	-1.224644	3.000000	-32.987820	14.654487	-0.544554
Eubacterium ventriosum	Dopamine	0.000010	t_k	38.583333	33.916667	-4.666667	0.230009	-1.502397	3.000000	-14.551812	5.218479	-0.277228
Eubacterium ventriosum	Dopamine	0.000001	t_k	38.583333	33.416667	-5.166667	0.220234	-1.544220	3.000000	-15.814527	5.481194	-0.306931
Eubacterium ventriosum	Noradrenaline	0.100000	t_k	36.416667	37.750000	1.333333	0.746119	0.354943	3.000000	-10.621449	13.288116	0.079208
Eubacterium ventriosum	Noradrenaline	0.010000	t_k	36.416667	36.250000	-0.166667	0.989101	-0.014828	3.000000	-35.938070	35.604737	-0.009901
Eubacterium ventriosum	Noradrenaline	0.001000	t_k	36.416667	37.583333	1.166667	0.887518	0.153818	3.000000	-22.971256	25.304589	0.069307
Eubacterium ventriosum	Noradrenaline	0.000100	t_k	36.416667	34.416667	-2.000000	0.767611	-0.323420	3.000000	-21.679961	17.679961	-0.118812

Species	Hormon	Concentration	Parameter	Mean_Baseline	Mean_Treatment	Diff	P_Value	T_Statistic	DF	Lower_CI	Upper_CI	Diff_Norm
Eubacterium ventriosum	Noradrenaline	0.000010	t_k	36.416667	29.250000	-7.166667	0.542832	-0.684439	3.000000	-40.489613	26.156280	-0.425743
Eubacterium ventriosum	Noradrenaline	0.000001	t_k	36.416667	28.250000	-8.166667	0.316942	-1.197976	3.000000	-29.861574	13.528241	-0.485149
Eubacterium ventriosum	Adrenaline	0.100000	t_k	43.083333	45.416667	2.333333	0.599218	0.585711	3.000000	-10.344780	15.011446	0.138614
Eubacterium ventriosum	Adrenaline	0.010000	t_k	43.083333	26.250000	-16.833333	0.099677	-2.356924	3.000000	-39.562609	5.895942	-1.000000
Eubacterium ventriosum	Adrenaline	0.001000	t_k	43.083333	34.250000	-8.833333	0.409609	-0.955977	3.000000	-38.239487	20.572820	-0.524752
Eubacterium ventriosum	Adrenaline	0.000100	t_k	43.083333	27.500000	-15.583333	0.050406	-3.171922	3.000000	-31.218370	0.051703	-0.925743
Eubacterium ventriosum	Adrenaline	0.000010	t_k	43.083333	31.166667	-11.916667	0.131592	-2.059242	3.000000	-30.333224	6.499891	-0.707921
Eubacterium ventriosum	Adrenaline	0.000001	t_k	43.083333	33.833333	-9.250000	0.261229	-1.380792	3.000000	-30.569377	12.069377	-0.549505
Eubacterium ventriosum	Levodopa	0.100000	t_gr	3.750000	3.916667	0.166667	0.181690	1.732051	3.000000	-0.139564	0.472898	0.500000
Eubacterium ventriosum	Levodopa	0.010000	t_gr	3.750000	3.833333	0.083333	0.637618	0.522233	3.000000	-0.424493	0.591160	0.250000
Eubacterium ventriosum	Levodopa	0.001000	t_gr	3.750000	3.833333	0.083333	0.391002	1.000000	3.000000	-0.181871	0.348537	0.250000
Eubacterium ventriosum	Levodopa	0.000100	t_gr	3.750000	3.833333	0.083333	0.391002	1.000000	3.000000	-0.181871	0.348537	0.250000
Eubacterium ventriosum	Levodopa	0.000010	t_gr	3.750000	3.916667	0.166667	0.181690	1.732051	3.000000	-0.139564	0.472898	0.500000
Eubacterium ventriosum	Levodopa	0.000001	t_gr	3.750000	3.916667	0.166667	0.181690	1.732051	3.000000	-0.139564	0.472898	0.500000
Eubacterium ventriosum	Dopamine	0.100000	t_gr	3.666667	3.750000	0.083333	0.391002	1.000000	3.000000	-0.181871	0.348537	0.250000
Eubacterium ventriosum	Dopamine	0.010000	t_gr	3.666667	3.583333	-0.083333	0.391002	-1.000000	3.000000	-0.348537	0.181871	-0.250000
Eubacterium ventriosum	Dopamine	0.001000	t_gr	3.666667	3.666667	0.000000	NA	NA	3.000000	NA	NA	0.000000
Eubacterium ventriosum	Dopamine	0.000100	t_gr	3.666667	3.583333	-0.083333	0.391002	-1.000000	3.000000	-0.348537	0.181871	-0.250000
Eubacterium ventriosum	Dopamine	0.000010	t_gr	3.666667	3.666667	0.000000	NA	NA	3.000000	NA	NA	0.000000
Eubacterium ventriosum	Dopamine	0.000001	t_gr	3.666667	3.666667	0.000000	NA	NA	3.000000	NA	NA	0.000000
Eubacterium ventriosum	Noradrenaline	0.100000	t_gr	3.666667	3.833333	0.166667	0.181690	1.732051	3.000000	-0.139564	0.472898	0.500000
Eubacterium ventriosum	Noradrenaline	0.010000	t_gr	3.666667	3.666667	0.000000	NA	NA	3.000000	NA	NA	0.000000
Eubacterium ventriosum	Noradrenaline	0.001000	t_gr	3.666667	3.916667	0.250000	0.057669	3.000000	3.000000	-0.015204	0.515204	0.750000
Eubacterium ventriosum	Noradrenaline	0.000100	t_gr	3.666667	4.000000	0.333333	0.000000	1333333617.000000	3.000000	0.333333	0.333333	1.000000
Eubacterium ventriosum	Noradrenaline	0.000010	t_gr	3.666667	3.666667	0.000000	NA	NA	3.000000	NA	NA	0.000000
Eubacterium ventriosum	Noradrenaline	0.000001	t_gr	3.666667	3.666667	0.000000	NA	NA	3.000000	NA	NA	0.000000
Eubacterium ventriosum	Adrenaline	0.100000	t_gr	3.666667	3.750000	0.083333	0.391002	1.000000	3.000000	-0.181871	0.348537	0.250000

Species	Hormon	Concentration	Parameter	Mean_Baseline	Mean_Treatment	Diff	P_Value	T_Statistic	DF	Lower_CI	Upper_CI	Diff_Norm
Eubacterium ventriosum	Adrenaline	0.010000	t_gr	3.666667	3.750000	0.083333	0.391002	1.000000	3.000000	-0.181871	0.348537	0.250000
Eubacterium ventriosum	Adrenaline	0.001000	t_gr	3.666667	3.916667	0.250000	0.057669	3.000000	3.000000	-0.015204	0.515204	0.750000
Eubacterium ventriosum	Adrenaline	0.000100	t_gr	3.666667	3.916667	0.250000	0.057669	3.000000	3.000000	-0.015204	0.515204	0.750000
Eubacterium ventriosum	Adrenaline	0.000010	t_gr	3.666667	3.750000	0.083333	0.391002	1.000000	3.000000	-0.181871	0.348537	0.250000
Eubacterium ventriosum	Adrenaline	0.000001	t_gr	3.666667	3.833333	0.166667	0.181690	1.732051	3.000000	-0.139564	0.472898	0.500000
Eubacterium ventriosum	Levodopa	0.100000	t_dr	48.000000	47.666667	-0.333333	0.391002	-1.000000	3.000000	-1.394149	0.727482	-0.031250
Eubacterium ventriosum	Levodopa	0.010000	t_dr	48.000000	44.500000	-3.500000	0.350858	-1.102214	3.000000	-13.605624	6.605624	-0.328125
Eubacterium ventriosum	Levodopa	0.001000	t_dr	48.000000	48.000000	0.000000	NA	NA	3.000000	NA	NA	0.000000
Eubacterium ventriosum	Levodopa	0.000100	t_dr	48.000000	42.833333	-5.166667	0.391002	-1.000000	3.000000	-21.609306	11.275973	-0.484375
Eubacterium ventriosum	Levodopa	0.000010	t_dr	48.000000	39.000000	-9.000000	0.211728	-1.582315	3.000000	-27.101337	9.101337	-0.843750
Eubacterium ventriosum	Levodopa	0.000001	t_dr	48.000000	42.166667	-5.833333	0.391002	-1.000000	3.000000	-24.397603	12.730937	-0.546875
Eubacterium ventriosum	Dopamine	0.100000	t_dr	48.000000	48.000000	0.000000	NA	NA	3.000000	NA	NA	0.000000
Eubacterium ventriosum	Dopamine	0.010000	t_dr	48.000000	44.500000	-3.500000	0.391002	-1.000000	3.000000	-14.638562	7.638562	-0.328125
Eubacterium ventriosum	Dopamine	0.001000	t_dr	48.000000	43.000000	-5.000000	0.391002	-1.000000	3.000000	-20.912232	10.912232	-0.468750
Eubacterium ventriosum	Dopamine	0.000100	t_dr	48.000000	48.000000	0.000000	NA	NA	3.000000	NA	NA	0.000000
Eubacterium ventriosum	Dopamine	0.000010	t_dr	48.000000	40.916667	-7.083333	0.391002	-1.000000	3.000000	-29.625661	15.458995	-0.664062
Eubacterium ventriosum	Dopamine	0.000001	t_dr	48.000000	42.500000	-5.500000	0.391002	-1.000000	3.000000	-23.003455	12.003455	-0.515625
Eubacterium ventriosum	Noradrenaline	0.100000	t_dr	48.000000	48.000000	0.000000	NA	NA	3.000000	NA	NA	0.000000
Eubacterium ventriosum	Noradrenaline	0.010000	t_dr	48.000000	48.000000	0.000000	NA	NA	3.000000	NA	NA	0.000000
Eubacterium ventriosum	Noradrenaline	0.001000	t_dr	48.000000	48.000000	0.000000	NA	NA	3.000000	NA	NA	0.000000
Eubacterium ventriosum	Noradrenaline	0.000100	t_dr	48.000000	48.000000	0.000000	NA	NA	3.000000	NA	NA	0.000000
Eubacterium ventriosum	Noradrenaline	0.000010	t_dr	48.000000	37.333333	-10.666667	0.200976	-1.632993	3.000000	-31.454319	10.120986	-1.000000
Eubacterium ventriosum	Noradrenaline	0.000001	t_dr	48.000000	48.000000	0.000000	NA	NA	3.000000	NA	NA	0.000000
Eubacterium ventriosum	Adrenaline	0.100000	t_dr	48.000000	48.000000	0.000000	NA	NA	3.000000	NA	NA	0.000000
Eubacterium ventriosum	Adrenaline	0.010000	t_dr	48.000000	48.000000	0.000000	NA	NA	3.000000	NA	NA	0.000000
Eubacterium ventriosum	Adrenaline	0.001000	t_dr	48.000000	48.000000	0.000000	NA	NA	3.000000	NA	NA	0.000000
Eubacterium ventriosum	Adrenaline	0.000100	t_dr	48.000000	48.000000	0.000000	NA	NA	3.000000	NA	NA	0.000000

Species	Hormon	Concentration	Parameter	Mean_Baseline	Mean_Treatment	Diff	P_Value	T_Statistic	DF	Lower_CI	Upper_CI	Diff_Norm
Eubacterium ventriosum	Adrenaline	0.000010	t_dr	48.000000	48.000000	0.000000	NA	NA	3.000000	NA	NA	0.000000
Eubacterium ventriosum	Adrenaline	0.000001	t_dr	48.000000	48.000000	0.000000	NA	NA	3.000000	NA	NA	0.000000
Klebsiella pneumoniae	Levodopa	0.100000	auc_lin	15.497648	14.332554	-1.165095	0.115117	-2.200510	3.000000	-2.850090	0.519901	-0.346715
Klebsiella pneumoniae	Levodopa	0.010000	auc_lin	15.497648	17.591250	2.093602	0.347174	1.112158	3.000000	-3.897250	8.084454	0.623025
Klebsiella pneumoniae	Levodopa	0.001000	auc_lin	15.497648	17.787651	2.290003	0.393040	0.995084	3.000000	-5.033810	9.613816	0.681471
Klebsiella pneumoniae	Levodopa	0.000100	auc_lin	15.497648	16.980643	1.482995	0.084372	2.544166	3.000000	-0.372054	3.338044	0.441317
Klebsiella pneumoniae	Levodopa	0.000010	auc_lin	15.497648	17.193991	1.696343	0.226270	1.518160	3.000000	-1.859619	5.252305	0.504806
Klebsiella pneumoniae	Levodopa	0.000001	auc_lin	15.497648	15.027843	-0.469805	0.520671	-0.725400	3.000000	-2.530918	1.591307	-0.139807
Klebsiella pneumoniae	Dopamine	0.100000	auc_lin	16.748261	18.914492	2.166232	0.030676	3.862741	3.000000	0.381510	3.950953	0.644638
Klebsiella pneumoniae	Dopamine	0.010000	auc_lin	16.748261	19.747503	2.999242	0.018578	4.666464	3.000000	0.953812	5.044672	0.892530
Klebsiella pneumoniae	Dopamine	0.001000	auc_lin	16.748261	20.108644	3.360383	0.016169	4.910939	3.000000	1.182747	5.538019	1.000000
Klebsiella pneumoniae	Dopamine	0.000100	auc_lin	16.748261	19.700149	2.951888	0.012732	5.356281	3.000000	1.198017	4.705759	0.878438
Klebsiella pneumoniae	Dopamine	0.000010	auc_lin	16.748261	19.176661	2.428401	0.027687	4.017652	3.000000	0.504826	4.351975	0.722656
Klebsiella pneumoniae	Dopamine	0.000001	auc_lin	16.748261	18.388304	1.640043	0.048321	3.227147	3.000000	0.022717	3.257368	0.488052
Klebsiella pneumoniae	Noradrenaline	0.100000	auc_lin	17.489228	18.516358	1.027130	0.183512	1.722195	3.000000	-0.870905	2.925164	0.305658
Klebsiella pneumoniae	Noradrenaline	0.010000	auc_lin	17.489228	18.350623	0.861395	0.236244	1.476730	3.000000	-0.994966	2.717756	0.256338
Klebsiella pneumoniae	Noradrenaline	0.001000	auc_lin	17.489228	17.266949	-0.222279	0.788562	-0.293084	3.000000	-2.635897	2.191338	-0.066147
Klebsiella pneumoniae	Noradrenaline	0.000100	auc_lin	17.489228	19.294811	1.805583	0.145179	1.957693	3.000000	-1.129592	4.740759	0.537315
Klebsiella pneumoniae	Noradrenaline	0.000010	auc_lin	17.489228	17.972428	0.483200	0.026212	4.102290	3.000000	0.108346	0.858053	0.143793
Klebsiella pneumoniae	Noradrenaline	0.000001	auc_lin	17.489228	19.270057	1.780829	0.162282	1.844673	3.000000	-1.291473	4.853130	0.529948
Klebsiella pneumoniae	Adrenaline	0.100000	auc_lin	17.906235	18.910356	1.004121	0.268635	1.354243	3.000000	-1.355545	3.363788	0.298811
Klebsiella pneumoniae	Adrenaline	0.010000	auc_lin	17.906235	18.396901	0.490666	0.460493	0.844332	3.000000	-1.358745	2.340076	0.146015
Klebsiella pneumoniae	Adrenaline	0.001000	auc_lin	17.906235	18.417786	0.511551	0.486664	0.791093	3.000000	-1.546338	2.569439	0.152230
Klebsiella pneumoniae	Adrenaline	0.000100	auc_lin	17.906235	19.084575	1.178340	0.022614	4.337270	3.000000	0.313740	2.042940	0.350656
Klebsiella pneumoniae	Adrenaline	0.000010	auc_lin	17.906235	18.283620	0.377385	0.425251	0.920381	3.000000	-0.927516	1.682285	0.112304
Klebsiella pneumoniae	Adrenaline	0.000001	auc_lin	17.906235	18.162220	0.255985	0.537805	0.693614	3.000000	-0.918528	1.430498	0.076177
Klebsiella pneumoniae	Levodopa	0.100000	k_lin	0.417808	0.382212	-0.035597	0.012975	-5.319906	3.000000	-0.056891	-0.014302	-0.732584

Species	Hormon	Concentration	Parameter	Mean_Baseline	Mean_Treatment	Diff	P_Value	T_Statistic	DF	Lower_CI	Upper_CI	Diff_Norm
Klebsiella pneumoniae	Levodopa	0.010000	k_lin	0.417808	0.453600	0.035792	0.464792	0.835411	3.000000	-0.100555	0.172138	0.736601
Klebsiella pneumoniae	Levodopa	0.001000	k_lin	0.417808	0.451801	0.033993	0.582679	0.613929	3.000000	-0.142217	0.210202	0.699577
Klebsiella pneumoniae	Levodopa	0.000100	k_lin	0.417808	0.433554	0.015746	0.323865	1.177619	3.000000	-0.026807	0.058299	0.324058
Klebsiella pneumoniae	Levodopa	0.000010	k_lin	0.417808	0.441981	0.024173	0.440283	0.887281	3.000000	-0.062529	0.110876	0.497487
Klebsiella pneumoniae	Levodopa	0.000001	k_lin	0.417808	0.395687	-0.022122	0.164608	-1.830373	3.000000	-0.060584	0.016341	-0.455266
Klebsiella pneumoniae	Dopamine	0.100000	k_lin	0.465580	0.492875	0.027295	0.031002	3.847021	3.000000	0.004715	0.049875	0.561744
Klebsiella pneumoniae	Dopamine	0.010000	k_lin	0.465580	0.507567	0.041987	0.016431	4.882110	3.000000	0.014618	0.069357	0.864110
Klebsiella pneumoniae	Dopamine	0.001000	k_lin	0.465580	0.514170	0.048590	0.033614	3.728318	3.000000	0.007114	0.090067	1.000000
Klebsiella pneumoniae	Dopamine	0.000100	k_lin	0.465580	0.503941	0.038362	0.024604	4.201835	3.000000	0.009307	0.067416	0.789489
Klebsiella pneumoniae	Dopamine	0.000010	k_lin	0.465580	0.495719	0.030139	0.045264	3.313773	3.000000	0.001194	0.059084	0.620267
Klebsiella pneumoniae	Dopamine	0.000001	k_lin	0.465580	0.492028	0.026448	0.058580	2.980357	3.000000	-0.001793	0.054689	0.544306
Klebsiella pneumoniae	Noradrenaline	0.100000	k_lin	0.453984	0.478827	0.024844	0.059571	2.959433	3.000000	-0.001872	0.051560	0.511289
Klebsiella pneumoniae	Noradrenaline	0.010000	k_lin	0.453984	0.475314	0.021330	0.142823	1.974476	3.000000	-0.013050	0.055710	0.438983
Klebsiella pneumoniae	Noradrenaline	0.001000	k_lin	0.453984	0.447006	-0.006978	0.772556	-0.316226	3.000000	-0.077205	0.063249	-0.143611
Klebsiella pneumoniae	Noradrenaline	0.000100	k_lin	0.453984	0.495393	0.041410	0.185258	1.712849	3.000000	-0.035529	0.118348	0.852218
Klebsiella pneumoniae	Noradrenaline	0.000010	k_lin	0.453984	0.465176	0.011192	0.029342	3.929380	3.000000	0.002128	0.020257	0.230338
Klebsiella pneumoniae	Noradrenaline	0.000001	k_lin	0.453984	0.496896	0.042912	0.226045	1.519116	3.000000	-0.046986	0.132809	0.883134
Klebsiella pneumoniae	Adrenaline	0.100000	k_lin	0.467640	0.483286	0.015647	0.386245	1.011573	3.000000	-0.033578	0.064871	0.322010
Klebsiella pneumoniae	Adrenaline	0.010000	k_lin	0.467640	0.474371	0.006732	0.615839	0.557902	3.000000	-0.031667	0.045130	0.138536
Klebsiella pneumoniae	Adrenaline	0.001000	k_lin	0.467640	0.474567	0.006928	0.642742	0.513960	3.000000	-0.035968	0.049823	0.142570
Klebsiella pneumoniae	Adrenaline	0.000100	k_lin	0.467640	0.485911	0.018271	0.035291	3.658146	3.000000	0.002376	0.034166	0.376020
Klebsiella pneumoniae	Adrenaline	0.000010	k_lin	0.467640	0.470319	0.002680	0.626097	0.540998	3.000000	-0.013083	0.018442	0.055146
Klebsiella pneumoniae	Adrenaline	0.000001	k_lin	0.467640	0.465034	-0.002605	0.773759	-0.314480	3.000000	-0.028970	0.023759	-0.053617
Klebsiella pneumoniae	Levodopa	0.100000	death_lin	0.017922	0.018868	0.000946	0.931719	0.093065	3.000000	-0.031414	0.033306	0.045513
Klebsiella pneumoniae	Levodopa	0.010000	death_lin	0.017922	0.018168	0.000247	0.950963	0.066774	3.000000	-0.011514	0.012008	0.011868
Klebsiella pneumoniae	Levodopa	0.001000	death_lin	0.017922	0.018365	0.000443	0.951428	0.066139	3.000000	-0.020896	0.021783	0.021330
Klebsiella pneumoniae	Levodopa	0.000100	death_lin	0.017922	0.025436	0.007515	0.324339	1.176241	3.000000	-0.012817	0.027846	0.361413

Species	Hormon	Concentration	Parameter	Mean_Baseline	Mean_Treatment	Diff	P_Value	T_Statistic	DF	Lower_CI	Upper_CI	Diff_Norm
Klebsiella pneumoniae	Levodopa	0.000010	death_lin	0.017922	0.028083	0.010162	0.153989	1.897644	3.000000	-0.006880	0.027203	0.488727
Klebsiella pneumoniae	Levodopa	0.000001	death_lin	0.017922	0.023752	0.005830	0.302720	1.241263	3.000000	-0.009117	0.020777	0.280396
Klebsiella pneumoniae	Dopamine	0.100000	death_lin	0.002947	0.016229	0.013283	0.010911	5.661862	3.000000	0.005817	0.020748	0.638828
Klebsiella pneumoniae	Dopamine	0.010000	death_lin	0.002947	0.023739	0.020792	0.000517	16.145921	3.000000	0.016694	0.024890	1.000000
Klebsiella pneumoniae	Dopamine	0.001000	death_lin	0.002947	0.014729	0.011783	0.087287	2.505441	3.000000	-0.003184	0.026749	0.566696
Klebsiella pneumoniae	Dopamine	0.000100	death_lin	0.002947	0.015400	0.012453	0.076991	2.650039	3.000000	-0.002502	0.027408	0.598940
Klebsiella pneumoniae	Dopamine	0.000010	death_lin	0.002947	0.008955	0.006008	0.346396	1.114271	3.000000	-0.011152	0.023168	0.288967
Klebsiella pneumoniae	Dopamine	0.000001	death_lin	0.002947	0.010391	0.007444	0.202307	1.626560	3.000000	-0.007121	0.022010	0.358043
Klebsiella pneumoniae	Noradrenaline	0.100000	death_lin	0.016859	0.027169	0.010310	0.090629	2.462960	3.000000	-0.003012	0.023633	0.495882
Klebsiella pneumoniae	Noradrenaline	0.010000	death_lin	0.016859	0.019350	0.002491	0.702001	0.421146	3.000000	-0.016335	0.021317	0.119820
Klebsiella pneumoniae	Noradrenaline	0.001000	death_lin	0.016859	0.027015	0.010156	0.028892	3.952737	3.000000	0.001979	0.018333	0.488464
Klebsiella pneumoniae	Noradrenaline	0.000100	death_lin	0.016859	0.025718	0.008859	0.222906	1.532586	3.000000	-0.009537	0.027255	0.426074
Klebsiella pneumoniae	Noradrenaline	0.000010	death_lin	0.016859	0.026960	0.010101	0.074107	2.694876	3.000000	-0.001828	0.022031	0.485833
Klebsiella pneumoniae	Noradrenaline	0.000001	death_lin	0.016859	0.021570	0.004711	0.635549	0.525586	3.000000	-0.023813	0.033235	0.226568
Klebsiella pneumoniae	Adrenaline	0.100000	death_lin	0.013623	0.023169	0.009546	0.298970	1.253024	3.000000	-0.014699	0.033790	0.459102
Klebsiella pneumoniae	Adrenaline	0.010000	death_lin	0.013623	0.020683	0.007060	0.400234	0.977920	3.000000	-0.015915	0.030034	0.339542
Klebsiella pneumoniae	Adrenaline	0.001000	death_lin	0.013623	0.023424	0.009801	0.196252	1.656232	3.000000	-0.009032	0.028634	0.471384
Klebsiella pneumoniae	Adrenaline	0.000100	death_lin	0.013623	0.017221	0.003598	0.636012	0.524836	3.000000	-0.018220	0.025416	0.173054
Klebsiella pneumoniae	Adrenaline	0.000010	death_lin	0.013623	0.016645	0.003021	0.713818	0.403198	3.000000	-0.020826	0.026869	0.145311
Klebsiella pneumoniae	Adrenaline	0.000001	death_lin	0.013623	0.013662	0.000039	0.994994	0.006811	3.000000	-0.018137	0.018215	0.001871
Klebsiella pneumoniae	Levodopa	0.100000	gr	0.595516	0.477190	-0.118326	0.019404	-4.591958	3.000000	-0.200332	-0.036321	-1.000000
Klebsiella pneumoniae	Levodopa	0.010000	gr	0.595516	0.599318	0.003802	0.945868	0.073727	3.000000	-0.160329	0.167934	0.032135
Klebsiella pneumoniae	Levodopa	0.001000	gr	0.595516	0.629856	0.034340	0.559079	0.655231	3.000000	-0.132449	0.201129	0.290215
Klebsiella pneumoniae	Levodopa	0.000100	gr	0.595516	0.554529	-0.040987	0.307297	-1.227109	3.000000	-0.147284	0.065310	-0.346386
Klebsiella pneumoniae	Levodopa	0.000010	gr	0.595516	0.574812	-0.020704	0.057745	-2.998344	3.000000	-0.042679	0.001271	-0.174971
Klebsiella pneumoniae	Levodopa	0.000001	gr	0.595516	0.547541	-0.047975	0.303643	-1.238389	3.000000	-0.171261	0.075312	-0.405443
Klebsiella pneumoniae	Dopamine	0.100000	gr	0.636372	0.588474	-0.047898	0.026201	-4.102921	3.000000	-0.085050	-0.010746	-0.404795

Species	Hormon	Concentration	Parameter	Mean_Baseline	Mean_Treatment	Diff	P_Value	T_Statistic	DF	Lower_CI	Upper_CI	Diff_Norm
Klebsiella pneumoniae	Dopamine	0.010000	gr	0.636372	0.633544	-0.002828	0.680736	-0.453884	3.000000	-0.022655	0.016999	-0.023898
Klebsiella pneumoniae	Dopamine	0.001000	gr	0.636372	0.634966	-0.001406	0.841966	-0.217228	3.000000	-0.022004	0.019192	-0.011882
Klebsiella pneumoniae	Dopamine	0.000100	gr	0.636372	0.675291	0.038919	0.143737	1.967929	3.000000	-0.024019	0.101858	0.328914
Klebsiella pneumoniae	Dopamine	0.000010	gr	0.636372	0.649196	0.012825	0.490226	0.784036	3.000000	-0.039232	0.064881	0.108384
Klebsiella pneumoniae	Dopamine	0.000001	gr	0.636372	0.639747	0.003375	0.723584	0.388489	3.000000	-0.024272	0.031022	0.028523
Klebsiella pneumoniae	Noradrenaline	0.100000	gr	0.653593	0.616943	-0.036650	0.007873	-6.358051	3.000000	-0.054995	-0.018305	-0.309736
Klebsiella pneumoniae	Noradrenaline	0.010000	gr	0.653593	0.630536	-0.023057	0.200671	-1.634476	3.000000	-0.067952	0.021837	-0.194863
Klebsiella pneumoniae	Noradrenaline	0.001000	gr	0.653593	0.607689	-0.045904	0.308407	-1.223708	3.000000	-0.165285	0.073477	-0.387946
Klebsiella pneumoniae	Noradrenaline	0.000100	gr	0.653593	0.628350	-0.025244	0.151439	-1.914604	3.000000	-0.067203	0.016716	-0.213338
Klebsiella pneumoniae	Noradrenaline	0.000010	gr	0.653593	0.629243	-0.024350	0.559211	-0.654997	3.000000	-0.142657	0.093958	-0.205783
Klebsiella pneumoniae	Noradrenaline	0.000001	gr	0.653593	0.655900	0.002307	0.874068	0.172441	3.000000	-0.040267	0.044881	0.019496
Klebsiella pneumoniae	Adrenaline	0.100000	gr	0.647305	0.581610	-0.065694	0.183461	-1.722466	3.000000	-0.187072	0.055683	-0.555197
Klebsiella pneumoniae	Adrenaline	0.010000	gr	0.647305	0.637006	-0.010299	0.419872	-0.932485	3.000000	-0.045449	0.024851	-0.087041
Klebsiella pneumoniae	Adrenaline	0.001000	gr	0.647305	0.648832	0.001527	0.835147	0.226812	3.000000	-0.019896	0.022950	0.012903
Klebsiella pneumoniae	Adrenaline	0.000100	gr	0.647305	0.655446	0.008141	0.494114	0.776384	3.000000	-0.025230	0.041513	0.068803
Klebsiella pneumoniae	Adrenaline	0.000010	gr	0.647305	0.638583	-0.008722	0.070956	-2.746403	3.000000	-0.018828	0.001385	-0.073709
Klebsiella pneumoniae	Adrenaline	0.000001	gr	0.647305	0.647846	0.000541	0.959442	0.055210	3.000000	-0.030662	0.031744	0.004575
Klebsiella pneumoniae	Levodopa	0.100000	dr	-0.025688	-0.011253	0.014435	0.101936	2.332281	3.000000	-0.005262	0.034133	0.762168
Klebsiella pneumoniae	Levodopa	0.010000	dr	-0.025688	-0.011791	0.013897	0.088596	2.488565	3.000000	-0.003875	0.031668	0.733730
Klebsiella pneumoniae	Levodopa	0.001000	dr	-0.025688	-0.009012	0.016677	0.081436	2.584864	3.000000	-0.003855	0.037209	0.880504
Klebsiella pneumoniae	Levodopa	0.000100	dr	-0.025688	-0.012085	0.013604	0.164051	1.833778	3.000000	-0.010005	0.037212	0.718249
Klebsiella pneumoniae	Levodopa	0.000010	dr	-0.025688	-0.014600	0.011088	0.311425	1.214527	3.000000	-0.017966	0.040142	0.585426
Klebsiella pneumoniae	Levodopa	0.000001	dr	-0.025688	-0.014244	0.011444	0.288409	1.286989	3.000000	-0.016854	0.039742	0.604223
Klebsiella pneumoniae	Dopamine	0.100000	dr	-0.003627	-0.018463	-0.014836	0.071033	-2.745103	3.000000	-0.032035	0.002364	-0.783310
Klebsiella pneumoniae	Dopamine	0.010000	dr	-0.003627	-0.021163	-0.017536	0.076570	-2.656461	3.000000	-0.038544	0.003472	-0.925877
Klebsiella pneumoniae	Dopamine	0.001000	dr	-0.003627	-0.015798	-0.012171	0.171966	-1.786650	3.000000	-0.033850	0.009508	-0.642594
Klebsiella pneumoniae	Dopamine	0.000100	dr	-0.003627	-0.008814	-0.005187	0.366575	-1.060910	3.000000	-0.020745	0.010372	-0.273850

Species	Hormon	Concentration	Parameter	Mean_Baseline	Mean_Treatment	Diff	P_Value	T_Statistic	DF	Lower_CI	Upper_CI	Diff_Norm
Klebsiella pneumoniae	Dopamine	0.000010	dr	-0.003627	-0.010873	-0.007245	0.293233	-1.271317	3.000000	-0.025383	0.010892	-0.382548
Klebsiella pneumoniae	Dopamine	0.000001	dr	-0.003627	-0.018800	-0.015173	0.197883	-1.648139	3.000000	-0.044471	0.014125	-0.801103
Klebsiella pneumoniae	Noradrenaline	0.100000	dr	-0.011428	-0.030368	-0.018940	0.329147	-1.162379	3.000000	-0.070795	0.032915	-1.000000
Klebsiella pneumoniae	Noradrenaline	0.010000	dr	-0.011428	-0.011101	0.000327	0.856251	0.197234	3.000000	-0.004952	0.005607	0.017275
Klebsiella pneumoniae	Noradrenaline	0.001000	dr	-0.011428	-0.016034	-0.004606	0.240630	-1.459114	3.000000	-0.014652	0.005440	-0.243181
Klebsiella pneumoniae	Noradrenaline	0.000100	dr	-0.011428	-0.023703	-0.012275	0.357476	-1.084606	3.000000	-0.048292	0.023742	-0.648099
Klebsiella pneumoniae	Noradrenaline	0.000010	dr	-0.011428	-0.021933	-0.010505	0.035284	-3.658422	3.000000	-0.019643	-0.001367	-0.554635
Klebsiella pneumoniae	Noradrenaline	0.000001	dr	-0.011428	-0.018931	-0.007503	0.206152	-1.608231	3.000000	-0.022350	0.007344	-0.396146
Klebsiella pneumoniae	Adrenaline	0.100000	dr	-0.006714	-0.017399	-0.010685	0.226158	-1.518637	3.000000	-0.033077	0.011707	-0.564157
Klebsiella pneumoniae	Adrenaline	0.010000	dr	-0.006714	-0.014959	-0.008245	0.317380	-1.196674	3.000000	-0.030172	0.013682	-0.435319
Klebsiella pneumoniae	Adrenaline	0.001000	dr	-0.006714	-0.017418	-0.010704	0.112811	-2.222212	3.000000	-0.026034	0.004625	-0.565178
Klebsiella pneumoniae	Adrenaline	0.000100	dr	-0.006714	-0.010872	-0.004158	0.561366	-0.651173	3.000000	-0.024481	0.016165	-0.219557
Klebsiella pneumoniae	Adrenaline	0.000010	dr	-0.006714	-0.016030	-0.009316	0.201494	-1.630482	3.000000	-0.027500	0.008867	-0.491877
Klebsiella pneumoniae	Adrenaline	0.000001	dr	-0.006714	-0.017556	-0.010842	0.063122	-2.887888	3.000000	-0.022791	0.001106	-0.572463
Klebsiella pneumoniae	Levodopa	0.100000	td	1.192434	1.594862	0.402428	0.149599	1.927045	3.000000	-0.262167	1.067023	1.000000
Klebsiella pneumoniae	Levodopa	0.010000	td	1.192434	1.156812	-0.035623	0.788720	-0.292857	3.000000	-0.422729	0.351484	-0.088519
Klebsiella pneumoniae	Levodopa	0.001000	td	1.192434	1.100848	-0.091586	0.509213	-0.747129	3.000000	-0.481705	0.298532	-0.227585
Klebsiella pneumoniae	Levodopa	0.000100	td	1.192434	1.377511	0.185076	0.361146	1.074977	3.000000	-0.362838	0.732991	0.459900
Klebsiella pneumoniae	Levodopa	0.000010	td	1.192434	1.246987	0.054553	0.163335	1.838170	3.000000	-0.039895	0.149001	0.135559
Klebsiella pneumoniae	Levodopa	0.000001	td	1.192434	1.419754	0.227319	0.357110	1.085570	3.000000	-0.439087	0.893725	0.564869
Klebsiella pneumoniae	Dopamine	0.100000	td	1.089490	1.178315	0.088825	0.026901	4.062030	3.000000	0.019234	0.158415	0.220722
Klebsiella pneumoniae	Dopamine	0.010000	td	1.089490	1.094597	0.005107	0.666280	0.476489	3.000000	-0.029000	0.039213	0.012689
Klebsiella pneumoniae	Dopamine	0.001000	td	1.089490	1.092010	0.002520	0.836999	0.224206	3.000000	-0.033244	0.038283	0.006261
Klebsiella pneumoniae	Dopamine	0.000100	td	1.089490	1.030686	-0.058805	0.131703	-2.058358	3.000000	-0.149723	0.032114	-0.146125
Klebsiella pneumoniae	Dopamine	0.000010	td	1.089490	1.070062	-0.019428	0.527684	-0.712291	3.000000	-0.106229	0.067374	-0.048277
Klebsiella pneumoniae	Dopamine	0.000001	td	1.089490	1.084607	-0.004883	0.754059	-0.343247	3.000000	-0.050155	0.040390	-0.012134
Klebsiella pneumoniae	Noradrenaline	0.100000	td	1.060910	1.123544	0.062634	0.006634	6.752513	3.000000	0.033115	0.092154	0.155641

Species	Hormon	Concentration	Parameter	Mean_Baseline	Mean_Treatment	Diff	P_Value	T_Statistic	DF	Lower_CI	Upper_CI	Diff_Norm
Klebsiella pneumoniae	Noradrenaline	0.010000	td	1.060910	1.100296	0.039386	0.208412	1.597634	3.000000	-0.039070	0.117842	0.097871
Klebsiella pneumoniae	Noradrenaline	0.001000	td	1.060910	1.152340	0.091429	0.321602	1.184224	3.000000	-0.154275	0.337134	0.227195
Klebsiella pneumoniae	Noradrenaline	0.000100	td	1.060910	1.103454	0.042544	0.152494	1.907542	3.000000	-0.028434	0.113523	0.105719
Klebsiella pneumoniae	Noradrenaline	0.000010	td	1.060910	1.112878	0.051968	0.524946	0.717391	3.000000	-0.178568	0.282503	0.129135
Klebsiella pneumoniae	Noradrenaline	0.000001	td	1.060910	1.057520	-0.003390	0.888074	-0.153050	3.000000	-0.073877	0.067097	-0.008424
Klebsiella pneumoniae	Adrenaline	0.100000	td	1.071812	1.200586	0.128773	0.203683	1.619956	3.000000	-0.124205	0.381752	0.319991
Klebsiella pneumoniae	Adrenaline	0.010000	td	1.071812	1.088193	0.016380	0.429215	0.911551	3.000000	-0.040807	0.073567	0.040703
Klebsiella pneumoniae	Adrenaline	0.001000	td	1.071812	1.068847	-0.002965	0.811181	-0.260719	3.000000	-0.039161	0.033230	-0.007368
Klebsiella pneumoniae	Adrenaline	0.000100	td	1.071812	1.058092	-0.013720	0.490396	-0.783700	3.000000	-0.069435	0.041995	-0.034093
Klebsiella pneumoniae	Adrenaline	0.000010	td	1.071812	1.086088	0.014275	0.065862	2.836012	3.000000	-0.001744	0.030295	0.035473
Klebsiella pneumoniae	Adrenaline	0.000001	td	1.071812	1.070193	-0.001620	0.926351	-0.100413	3.000000	-0.052952	0.049713	-0.004025
Klebsiella pneumoniae	Levodopa	0.100000	lagC	2.672385	2.954494	0.282108	0.043151	3.378129	3.000000	0.016342	0.547875	0.835190
Klebsiella pneumoniae	Levodopa	0.010000	lagC	2.672385	2.630344	-0.042041	0.893733	-0.145237	3.000000	-0.963246	0.879164	-0.124464
Klebsiella pneumoniae	Levodopa	0.001000	lagC	2.672385	2.623709	-0.048676	0.862211	-0.188924	3.000000	-0.868628	0.771276	-0.144106
Klebsiella pneumoniae	Levodopa	0.000100	lagC	2.672385	2.763006	0.090620	0.548437	0.674288	3.000000	-0.337083	0.518324	0.268285
Klebsiella pneumoniae	Levodopa	0.000010	lagC	2.672385	2.717907	0.045521	0.784519	0.298910	3.000000	-0.439137	0.530179	0.134767
Klebsiella pneumoniae	Levodopa	0.000001	lagC	2.672385	2.859461	0.187076	0.141040	1.987400	3.000000	-0.112491	0.486642	0.553843
Klebsiella pneumoniae	Dopamine	0.100000	lagC	2.503509	2.654137	0.150628	0.018773	4.648507	3.000000	0.047505	0.253750	0.445938
Klebsiella pneumoniae	Dopamine	0.010000	lagC	2.503509	2.588281	0.084772	0.012262	5.429381	3.000000	0.035083	0.134462	0.250971
Klebsiella pneumoniae	Dopamine	0.001000	lagC	2.503509	2.560441	0.056932	0.117659	2.177189	3.000000	-0.026287	0.140151	0.168549
Klebsiella pneumoniae	Dopamine	0.000100	lagC	2.503509	2.590488	0.086979	0.116848	2.184563	3.000000	-0.039731	0.213689	0.257504
Klebsiella pneumoniae	Dopamine	0.000010	lagC	2.503509	2.569457	0.065948	0.061975	2.910424	3.000000	-0.006164	0.138060	0.195242
Klebsiella pneumoniae	Dopamine	0.000001	lagC	2.503509	2.547259	0.043751	0.333708	1.149417	3.000000	-0.077384	0.164885	0.129525
Klebsiella pneumoniae	Noradrenaline	0.100000	lagC	2.460209	2.625561	0.165351	0.036845	3.596817	3.000000	0.019049	0.311654	0.489528
Klebsiella pneumoniae	Noradrenaline	0.010000	lagC	2.460209	2.582110	0.121900	0.023502	4.274978	3.000000	0.031153	0.212647	0.360889
Klebsiella pneumoniae	Noradrenaline	0.001000	lagC	2.460209	2.797987	0.337777	0.244243	1.444864	3.000000	-0.406209	1.081764	1.000000
Klebsiella pneumoniae	Noradrenaline	0.000100	lagC	2.460209	2.569013	0.108804	0.057633	3.000784	3.000000	-0.006587	0.224194	0.322116

Species	Hormon	Concentration	Parameter	Mean_Baseline	Mean_Treatment	Diff	P_Value	T_Statistic	DF	Lower_CI	Upper_CI	Diff_Norm
Klebsiella pneumoniae	Noradrenaline	0.000010	lagC	2.460209	2.614596	0.154386	0.210506	1.587929	3.000000	-0.155027	0.463799	0.457065
Klebsiella pneumoniae	Noradrenaline	0.000001	lagC	2.460209	2.490852	0.030643	0.364583	1.066047	3.000000	-0.060834	0.122119	0.090718
Klebsiella pneumoniae	Adrenaline	0.100000	lagC	2.448877	2.507321	0.058444	0.571780	0.632846	3.000000	-0.235459	0.352347	0.173026
Klebsiella pneumoniae	Adrenaline	0.010000	lagC	2.448877	2.594843	0.145966	0.010345	5.770622	3.000000	0.065467	0.226466	0.432138
Klebsiella pneumoniae	Adrenaline	0.001000	lagC	2.448877	2.581896	0.133019	0.002143	9.976465	3.000000	0.090586	0.175451	0.393806
Klebsiella pneumoniae	Adrenaline	0.000100	lagC	2.448877	2.552284	0.103408	0.004262	7.877562	3.000000	0.061632	0.145183	0.306141
Klebsiella pneumoniae	Adrenaline	0.000010	lagC	2.448877	2.539711	0.090834	0.009356	5.980999	3.000000	0.042502	0.139166	0.268916
Klebsiella pneumoniae	Adrenaline	0.000001	lagC	2.448877	2.536544	0.087667	0.020301	4.515544	3.000000	0.025881	0.149452	0.259540
Klebsiella pneumoniae	Levodopa	0.100000	t_k	40.583333	35.583333	-5.000000	0.290317	-1.280758	3.000000	-17.424077	7.424077	-0.689655
Klebsiella pneumoniae	Levodopa	0.010000	t_k	40.583333	35.166667	-5.416667	0.086132	-2.520592	3.000000	-12.255636	1.422303	-0.747126
Klebsiella pneumoniae	Levodopa	0.001000	t_k	40.583333	36.916667	-3.666667	0.264800	-1.367894	3.000000	-12.197276	4.863943	-0.505747
Klebsiella pneumoniae	Levodopa	0.000100	t_k	40.583333	33.333333	-7.250000	0.017053	-4.816023	3.000000	-12.040828	-2.459172	-1.000000
Klebsiella pneumoniae	Levodopa	0.000010	t_k	40.583333	35.083333	-5.500000	0.080323	-2.600764	3.000000	-12.230119	1.230119	-0.758621
Klebsiella pneumoniae	Levodopa	0.000001	t_k	40.583333	33.750000	-6.833333	0.024531	-4.206511	3.000000	-12.003108	-1.663558	-0.942529
Klebsiella pneumoniae	Dopamine	0.100000	t_k	42.250000	37.083333	-5.166667	0.039831	-3.487772	3.000000	-9.881034	-0.452300	-0.712644
Klebsiella pneumoniae	Dopamine	0.010000	t_k	42.250000	36.083333	-6.166667	0.117738	-2.176471	3.000000	-15.183598	2.850265	-0.850575
Klebsiella pneumoniae	Dopamine	0.001000	t_k	42.250000	37.416667	-4.833333	0.016674	-4.855866	3.000000	-8.001012	-1.665655	-0.666667
Klebsiella pneumoniae	Dopamine	0.000100	t_k	42.250000	39.083333	-3.166667	0.102969	-2.321219	3.000000	-7.508241	1.174908	-0.436782
Klebsiella pneumoniae	Dopamine	0.000010	t_k	42.250000	37.833333	-4.416667	0.230279	-1.501270	3.000000	-13.779277	4.945944	-0.609195
Klebsiella pneumoniae	Dopamine	0.000001	t_k	42.250000	41.333333	-0.916667	0.576615	-0.624422	3.000000	-5.588572	3.755239	-0.126437
Klebsiella pneumoniae	Noradrenaline	0.100000	t_k	36.500000	34.166667	-2.333333	0.355058	-1.091001	3.000000	-9.139658	4.472992	-0.321839
Klebsiella pneumoniae	Noradrenaline	0.010000	t_k	36.500000	35.666667	-0.833333	0.569570	-0.636715	3.000000	-4.998526	3.331859	-0.114943
Klebsiella pneumoniae	Noradrenaline	0.001000	t_k	36.500000	33.583333	-2.916667	0.105672	-2.292926	3.000000	-6.964828	1.131495	-0.402299
Klebsiella pneumoniae	Noradrenaline	0.000100	t_k	36.500000	34.500000	-2.000000	0.478913	-0.806599	3.000000	-9.891022	5.891022	-0.275862
Klebsiella pneumoniae	Noradrenaline	0.000010	t_k	36.500000	36.333333	-0.166667	0.896156	-0.141895	3.000000	-3.904693	3.571360	-0.022989
Klebsiella pneumoniae	Noradrenaline	0.000001	t_k	36.500000	35.500000	-1.000000	0.876752	-0.168719	3.000000	-19.862440	17.862440	-0.137931
Klebsiella pneumoniae	Adrenaline	0.100000	t_k	41.083333	34.666667	-6.416667	0.311834	-1.213288	3.000000	-23.247538	10.414205	-0.885057

Species	Hormon	Concentration	Parameter	Mean_Baseline	Mean_Treatment	Diff	P_Value	T_Statistic	DF	Lower_CI	Upper_CI	Diff_Norm
Klebsiella pneumoniae	Adrenaline	0.010000	t_k	41.083333	36.000000	-5.083333	0.327098	-1.168262	3.000000	-18.930768	8.764101	-0.701149
Klebsiella pneumoniae	Adrenaline	0.001000	t_k	41.083333	34.750000	-6.333333	0.194222	-1.666410	3.000000	-18.428490	5.761824	-0.873563
Klebsiella pneumoniae	Adrenaline	0.000100	t_k	41.083333	37.250000	-3.833333	0.496810	-0.771106	3.000000	-19.653953	11.987286	-0.528736
Klebsiella pneumoniae	Adrenaline	0.000010	t_k	41.083333	36.333333	-4.750000	0.376965	-1.034541	3.000000	-19.361906	9.861906	-0.655172
Klebsiella pneumoniae	Adrenaline	0.000001	t_k	41.083333	34.666667	-6.416667	0.234780	-1.482690	3.000000	-20.189405	7.356072	-0.885057
Klebsiella pneumoniae	Levodopa	0.100000	t_gr	3.666667	3.833333	0.166667	0.181690	1.732051	3.000000	-0.139564	0.472898	0.666667
Klebsiella pneumoniae	Levodopa	0.010000	t_gr	3.666667	3.750000	0.083333	0.391002	1.000000	3.000000	-0.181871	0.348537	0.333333
Klebsiella pneumoniae	Levodopa	0.001000	t_gr	3.666667	3.750000	0.083333	0.391002	1.000000	3.000000	-0.181871	0.348537	0.333333
Klebsiella pneumoniae	Levodopa	0.000100	t_gr	3.666667	3.750000	0.083333	0.391002	1.000000	3.000000	-0.181871	0.348537	0.333333
Klebsiella pneumoniae	Levodopa	0.000010	t_gr	3.666667	3.750000	0.083333	0.391002	1.000000	3.000000	-0.181871	0.348537	0.333333
Klebsiella pneumoniae	Levodopa	0.000001	t_gr	3.666667	3.666667	0.000000	NA	NA	3.000000	NA	NA	0.000000
Klebsiella pneumoniae	Dopamine	0.100000	t_gr	3.666667	3.833333	0.166667	0.181690	1.732051	3.000000	-0.139564	0.472898	0.666667
Klebsiella pneumoniae	Dopamine	0.010000	t_gr	3.666667	3.666667	0.000000	NA	NA	3.000000	NA	NA	0.000000
Klebsiella pneumoniae	Dopamine	0.001000	t_gr	3.666667	3.750000	0.083333	0.391002	1.000000	3.000000	-0.181871	0.348537	0.333333
Klebsiella pneumoniae	Dopamine	0.000100	t_gr	3.666667	3.750000	0.083333	0.391002	1.000000	3.000000	-0.181871	0.348537	0.333333
Klebsiella pneumoniae	Dopamine	0.000010	t_gr	3.666667	3.666667	0.000000	NA	NA	3.000000	NA	NA	0.000000
Klebsiella pneumoniae	Dopamine	0.000001	t_gr	3.666667	3.750000	0.083333	0.391002	1.000000	3.000000	-0.181871	0.348537	0.333333
Klebsiella pneumoniae	Noradrenaline	0.100000	t_gr	3.583333	3.833333	0.250000	0.057669	3.000000	3.000000	-0.015204	0.515204	1.000000
Klebsiella pneumoniae	Noradrenaline	0.010000	t_gr	3.583333	3.666667	0.083333	0.391002	1.000000	3.000000	-0.181871	0.348537	0.333333
Klebsiella pneumoniae	Noradrenaline	0.001000	t_gr	3.583333	3.750000	0.166667	0.181690	1.732051	3.000000	-0.139564	0.472898	0.666667
Klebsiella pneumoniae	Noradrenaline	0.000100	t_gr	3.583333	3.666667	0.083333	0.391002	1.000000	3.000000	-0.181871	0.348537	0.333333
Klebsiella pneumoniae	Noradrenaline	0.000010	t_gr	3.583333	3.750000	0.166667	0.181690	1.732051	3.000000	-0.139564	0.472898	0.666667
Klebsiella pneumoniae	Noradrenaline	0.000001	t_gr	3.583333	3.583333	0.000000	NA	NA	3.000000	NA	NA	0.000000
Klebsiella pneumoniae	Adrenaline	0.100000	t_gr	3.583333	3.666667	0.083333	0.391002	1.000000	3.000000	-0.181871	0.348537	0.333333
Klebsiella pneumoniae	Adrenaline	0.010000	t_gr	3.583333	3.666667	0.083333	0.391002	1.000000	3.000000	-0.181871	0.348537	0.333333
Klebsiella pneumoniae	Adrenaline	0.001000	t_gr	3.583333	3.666667	0.083333	0.391002	1.000000	3.000000	-0.181871	0.348537	0.333333
Klebsiella pneumoniae	Adrenaline	0.000100	t_gr	3.583333	3.666667	0.083333	0.391002	1.000000	3.000000	-0.181871	0.348537	0.333333

Species	Hormon	Concentration	Parameter	Mean_Baseline	Mean_Treatment	Diff	P_Value	T_Statistic	DF	Lower_CI	Upper_CI	Diff_Norm
Klebsiella pneumoniae	Adrenaline	0.000010	t_gr	3.583333	3.666667	0.083333	0.391002	1.000000	3.000000	-0.181871	0.348537	0.333333
Klebsiella pneumoniae	Adrenaline	0.000001	t_gr	3.583333	3.666667	0.083333	0.391002	1.000000	3.000000	-0.181871	0.348537	0.333333
Klebsiella pneumoniae	Levodopa	0.100000	t_dr	48.000000	45.500000	-2.500000	0.391002	-1.000000	3.000000	-10.456116	5.456116	-0.526316
Klebsiella pneumoniae	Levodopa	0.010000	t_dr	48.000000	44.583333	-3.416667	0.349865	-1.104885	3.000000	-13.257838	6.424505	-0.719298
Klebsiella pneumoniae	Levodopa	0.001000	t_dr	48.000000	43.750000	-4.250000	0.151060	-1.917149	3.000000	-11.304954	2.804954	-0.894737
Klebsiella pneumoniae	Levodopa	0.000100	t_dr	48.000000	48.000000	0.000000	NA	NA	3.000000	NA	NA	0.000000
Klebsiella pneumoniae	Levodopa	0.000010	t_dr	48.000000	44.833333	-3.166667	0.391002	-1.000000	3.000000	-13.244413	6.911080	-0.666667
Klebsiella pneumoniae	Levodopa	0.000001	t_dr	48.000000	48.000000	0.000000	NA	NA	3.000000	NA	NA	0.000000
Klebsiella pneumoniae	Dopamine	0.100000	t_dr	44.750000	46.000000	1.250000	0.741065	0.362420	3.000000	-9.726364	12.226365	0.263158
Klebsiella pneumoniae	Dopamine	0.010000	t_dr	44.750000	44.666667	-0.083333	0.986909	-0.017809	3.000000	-14.974949	14.808283	-0.017544
Klebsiella pneumoniae	Dopamine	0.001000	t_dr	44.750000	47.083333	2.333333	0.424023	0.923133	3.000000	-5.710698	10.377365	0.491228
Klebsiella pneumoniae	Dopamine	0.000100	t_dr	44.750000	45.583333	0.833333	0.733463	0.373718	3.000000	-6.263038	7.929705	0.175439
Klebsiella pneumoniae	Dopamine	0.000010	t_dr	44.750000	45.083333	0.333333	0.895104	0.143346	3.000000	-7.067074	7.733741	0.070175
Klebsiella pneumoniae	Dopamine	0.000001	t_dr	44.750000	45.583333	0.833333	0.733463	0.373718	3.000000	-6.263038	7.929705	0.175439
Klebsiella pneumoniae	Noradrenaline	0.100000	t_dr	47.583333	44.083333	-3.500000	0.453504	-0.858993	3.000000	-16.466995	9.466995	-0.736842
Klebsiella pneumoniae	Noradrenaline	0.010000	t_dr	47.583333	46.250000	-1.333333	0.539181	-0.691095	3.000000	-7.473247	4.806580	-0.280702
Klebsiella pneumoniae	Noradrenaline	0.001000	t_dr	47.583333	44.083333	-3.500000	0.453504	-0.858993	3.000000	-16.466995	9.466995	-0.736842
Klebsiella pneumoniae	Noradrenaline	0.000100	t_dr	47.583333	42.833333	-4.750000	0.351979	-1.099207	3.000000	-18.502297	9.002297	-1.000000
Klebsiella pneumoniae	Noradrenaline	0.000010	t_dr	47.583333	48.000000	0.416667	0.391002	1.000000	3.000000	-0.909353	1.742686	0.087719
Klebsiella pneumoniae	Noradrenaline	0.000001	t_dr	47.583333	47.666667	0.083333	0.391002	1.000000	3.000000	-0.181871	0.348537	0.017544
Klebsiella pneumoniae	Adrenaline	0.100000	t_dr	48.000000	48.000000	0.000000	NA	NA	3.000000	NA	NA	0.000000
Klebsiella pneumoniae	Adrenaline	0.010000	t_dr	48.000000	48.000000	0.000000	NA	NA	3.000000	NA	NA	0.000000
Klebsiella pneumoniae	Adrenaline	0.001000	t_dr	48.000000	48.000000	0.000000	NA	NA	3.000000	NA	NA	0.000000
Klebsiella pneumoniae	Adrenaline	0.000100	t_dr	48.000000	47.083333	-0.916667	0.391002	-1.000000	3.000000	-3.833909	2.000576	-0.192982
Klebsiella pneumoniae	Adrenaline	0.000010	t_dr	48.000000	48.000000	0.000000	NA	NA	3.000000	NA	NA	0.000000
Klebsiella pneumoniae	Adrenaline	0.000001	t_dr	48.000000	45.750000	-2.250000	0.391002	-1.000000	3.000000	-9.410504	4.910504	-0.473684
Ruminococcus gnavus	Levodopa	0.100000	auc_lin	9.751321	7.045549	-2.705772	0.228804	-1.507448	3.000000	-8.418059	3.006515	-1.000000

Species	Hormon	Concentration	Parameter	Mean_Baseline	Mean_Treatment	Diff	P_Value	T_Statistic	DF	Lower_CI	Upper_CI	Diff_Norm
Ruminococcus gnavus	Levodopa	0.010000	auc_lin	9.751321	7.921539	-1.829782	0.330640	-1.158116	3.000000	-6.857934	3.198371	-0.676251
Ruminococcus gnavus	Levodopa	0.001000	auc_lin	9.751321	8.492561	-1.258760	0.514430	-0.737187	3.000000	-6.692846	4.175325	-0.465213
Ruminococcus gnavus	Levodopa	0.000100	auc_lin	9.751321	8.391199	-1.360122	0.432096	-0.905179	3.000000	-6.142068	3.421823	-0.502674
Ruminococcus gnavus	Levodopa	0.000010	auc_lin	9.751321	8.455139	-1.296182	0.512532	-0.740794	3.000000	-6.864568	4.272205	-0.479043
Ruminococcus gnavus	Levodopa	0.000001	auc_lin	9.751321	7.137330	-2.613991	0.264872	-1.367632	3.000000	-8.696684	3.468701	-0.966080
Ruminococcus gnavus	Dopamine	0.100000	auc_lin	7.987116	7.267723	-0.719394	0.145608	-1.954672	3.000000	-1.890655	0.451868	-0.265874
Ruminococcus gnavus	Dopamine	0.010000	auc_lin	7.987116	8.609766	0.622650	0.347790	1.110487	3.000000	-1.161747	2.407047	0.230119
Ruminococcus gnavus	Dopamine	0.001000	auc_lin	7.987116	10.139419	2.152303	0.443241	0.880887	3.000000	-5.623484	9.928089	0.795449
Ruminococcus gnavus	Dopamine	0.000100	auc_lin	7.987116	8.719788	0.732672	0.387313	1.008963	3.000000	-1.578304	3.043647	0.270781
Ruminococcus gnavus	Dopamine	0.000010	auc_lin	7.987116	9.378243	1.391127	0.220507	1.543024	3.000000	-1.478035	4.260288	0.514133
Ruminococcus gnavus	Dopamine	0.000001	auc_lin	7.987116	8.493275	0.506158	0.432100	0.905172	3.000000	-1.273416	2.285732	0.187066
Ruminococcus gnavus	Noradrenaline	0.100000	auc_lin	8.486293	7.614438	-0.871855	0.111362	-2.236116	3.000000	-2.112682	0.368971	-0.322221
Ruminococcus gnavus	Noradrenaline	0.010000	auc_lin	8.486293	8.085502	-0.400792	0.369639	-1.053060	3.000000	-1.612022	0.810438	-0.148125
Ruminococcus gnavus	Noradrenaline	0.001000	auc_lin	8.486293	8.176405	-0.309888	0.534129	-0.700365	3.000000	-1.718016	1.098240	-0.114529
Ruminococcus gnavus	Noradrenaline	0.000100	auc_lin	8.486293	8.231889	-0.254405	0.189726	-1.689392	3.000000	-0.733648	0.224838	-0.094023
Ruminococcus gnavus	Noradrenaline	0.000010	auc_lin	8.486293	7.935288	-0.551005	0.104349	-2.306670	3.000000	-1.311212	0.209201	-0.203641
Ruminococcus gnavus	Noradrenaline	0.000001	auc_lin	8.486293	7.805076	-0.681218	0.134335	-2.037764	3.000000	-1.745098	0.382663	-0.251765
Ruminococcus gnavus	Adrenaline	0.100000	auc_lin	7.682066	8.423230	0.741164	0.285793	1.295604	3.000000	-1.079388	2.561715	0.273920
Ruminococcus gnavus	Adrenaline	0.010000	auc_lin	7.682066	8.209379	0.527313	0.078699	2.624457	3.000000	-0.112113	1.166738	0.194884
Ruminococcus gnavus	Adrenaline	0.001000	auc_lin	7.682066	8.436672	0.754606	0.145676	1.954191	3.000000	-0.474288	1.983499	0.278887
Ruminococcus gnavus	Adrenaline	0.000100	auc_lin	7.682066	8.000283	0.318217	0.209981	1.590351	3.000000	-0.318566	0.955000	0.117607
Ruminococcus gnavus	Adrenaline	0.000010	auc_lin	7.682066	7.782619	0.100553	0.781414	0.303393	3.000000	-0.954203	1.155309	0.037162
Ruminococcus gnavus	Adrenaline	0.000001	auc_lin	7.682066	8.637294	0.955228	0.556609	0.659630	3.000000	-3.653359	5.563815	0.353033
Ruminococcus gnavus	Levodopa	0.100000	k_lin	0.276087	0.212687	-0.063400	0.223745	-1.528965	3.000000	-0.195364	0.068563	-1.000000
Ruminococcus gnavus	Levodopa	0.010000	k_lin	0.276087	0.243527	-0.032561	0.384995	-1.014635	3.000000	-0.134689	0.069567	-0.513575
Ruminococcus gnavus	Levodopa	0.001000	k_lin	0.276087	0.259415	-0.016673	0.700099	-0.424050	3.000000	-0.141800	0.108454	-0.262977
Ruminococcus gnavus	Levodopa	0.000100	k_lin	0.276087	0.257269	-0.018819	0.563518	-0.647364	3.000000	-0.111331	0.073694	-0.296824

Species	Hormon	Concentration	Parameter	Mean_Baseline	Mean_Treatment	Diff	P_Value	T_Statistic	DF	Lower_CI	Upper_CI	Diff_Norm
Ruminococcus gnavus	Levodopa	0.000010	k_lin	0.276087	0.259161	-0.016926	0.706431	-0.414398	3.000000	-0.146914	0.113061	-0.266973
Ruminococcus gnavus	Levodopa	0.000001	k_lin	0.276087	0.217350	-0.058737	0.235527	-1.479646	3.000000	-0.185070	0.067596	-0.926452
Ruminococcus gnavus	Dopamine	0.100000	k_lin	0.232928	0.221410	-0.011518	0.316558	-1.199118	3.000000	-0.042088	0.019051	-0.181679
Ruminococcus gnavus	Dopamine	0.010000	k_lin	0.232928	0.257015	0.024087	0.243252	1.448750	3.000000	-0.028824	0.076998	0.379917
Ruminococcus gnavus	Dopamine	0.001000	k_lin	0.232928	0.287154	0.054226	0.411936	0.950602	3.000000	-0.127312	0.235764	0.855294
Ruminococcus gnavus	Dopamine	0.000100	k_lin	0.232928	0.266507	0.033578	0.263000	1.374371	3.000000	-0.044175	0.111332	0.529628
Ruminococcus gnavus	Dopamine	0.000010	k_lin	0.232928	0.273699	0.040771	0.130366	2.069010	3.000000	-0.021941	0.103484	0.643079
Ruminococcus gnavus	Dopamine	0.000001	k_lin	0.232928	0.252461	0.019532	0.476535	0.811399	3.000000	-0.057077	0.096142	0.308081
Ruminococcus gnavus	Noradrenaline	0.100000	k_lin	0.245341	0.231741	-0.013601	0.329915	-1.160186	3.000000	-0.050907	0.023706	-0.214519
Ruminococcus gnavus	Noradrenaline	0.010000	k_lin	0.245341	0.237768	-0.007573	0.620679	-0.549903	3.000000	-0.051403	0.036256	-0.119455
Ruminococcus gnavus	Noradrenaline	0.001000	k_lin	0.245341	0.240648	-0.004693	0.735527	-0.370643	3.000000	-0.044993	0.035606	-0.074029
Ruminococcus gnavus	Noradrenaline	0.000100	k_lin	0.245341	0.241400	-0.003942	0.538272	-0.692758	3.000000	-0.022048	0.014165	-0.062169
Ruminococcus gnavus	Noradrenaline	0.000010	k_lin	0.245341	0.231902	-0.013439	0.173630	-1.777059	3.000000	-0.037506	0.010628	-0.211969
Ruminococcus gnavus	Noradrenaline	0.000001	k_lin	0.245341	0.226166	-0.019175	0.117644	-2.177323	3.000000	-0.047202	0.008852	-0.302443
Ruminococcus gnavus	Adrenaline	0.100000	k_lin	0.218459	0.245330	0.026871	0.205848	1.609665	3.000000	-0.026255	0.079997	0.423832
Ruminococcus gnavus	Adrenaline	0.010000	k_lin	0.218459	0.241310	0.022851	0.100388	2.349101	3.000000	-0.008106	0.053808	0.360421
Ruminococcus gnavus	Adrenaline	0.001000	k_lin	0.218459	0.251778	0.033319	0.121121	2.146384	3.000000	-0.016083	0.082721	0.525533
Ruminococcus gnavus	Adrenaline	0.000100	k_lin	0.218459	0.232681	0.014222	0.175812	1.764641	3.000000	-0.011427	0.039870	0.224318
Ruminococcus gnavus	Adrenaline	0.000010	k_lin	0.218459	0.231558	0.013098	0.325992	1.171452	3.000000	-0.022486	0.048682	0.206598
Ruminococcus gnavus	Adrenaline	0.000001	k_lin	0.218459	0.237411	0.018952	0.610926	0.566067	3.000000	-0.087595	0.125498	0.298920
Ruminococcus gnavus	Levodopa	0.100000	death_lin	0.038019	0.033983	-0.004036	0.657406	-0.490516	3.000000	-0.030220	0.022148	-0.112213
Ruminococcus gnavus	Levodopa	0.010000	death_lin	0.038019	0.045307	0.007288	0.544425	0.681546	3.000000	-0.026744	0.041321	0.202647
Ruminococcus gnavus	Levodopa	0.001000	death_lin	0.038019	0.049864	0.011845	0.409166	0.957002	3.000000	-0.027545	0.051235	0.329344
Ruminococcus gnavus	Levodopa	0.000100	death_lin	0.038019	0.046278	0.008259	0.527673	0.712311	3.000000	-0.028639	0.045157	0.229626
Ruminococcus gnavus	Levodopa	0.000010	death_lin	0.038019	0.051621	0.013602	0.120994	2.147495	3.000000	-0.006555	0.033760	0.378204
Ruminococcus gnavus	Levodopa	0.000001	death_lin	0.038019	0.039135	0.001115	0.884619	0.157825	3.000000	-0.021376	0.023607	0.031014
Ruminococcus gnavus	Dopamine	0.100000	death_lin	0.012219	0.022555	0.010336	0.484800	0.794804	3.000000	-0.031049	0.051721	0.287379

Species	Hormon	Concentration	Parameter	Mean_Baseline	Mean_Treatment	Diff	P_Value	T_Statistic	DF	Lower_CI	Upper_CI	Diff_Norm
Ruminococcus gnavus	Dopamine	0.010000	death_lin	0.012219	0.039323	0.027104	0.013456	5.250336	3.000000	0.010675	0.043533	0.753604
Ruminococcus gnavus	Dopamine	0.001000	death_lin	0.012219	0.027747	0.015528	0.176661	1.759856	3.000000	-0.012552	0.043608	0.431744
Ruminococcus gnavus	Dopamine	0.000100	death_lin	0.012219	0.048184	0.035966	0.018111	4.710541	3.000000	0.011667	0.060264	1.000000
Ruminococcus gnavus	Dopamine	0.000010	death_lin	0.012219	0.033247	0.021028	0.243339	1.448409	3.000000	-0.025174	0.067230	0.584662
Ruminococcus gnavus	Dopamine	0.000001	death_lin	0.012219	0.023008	0.010790	0.255214	1.402956	3.000000	-0.013685	0.035265	0.299998
Ruminococcus gnavus	Noradrenaline	0.100000	death_lin	0.031575	0.038451	0.006877	0.338556	1.135833	3.000000	-0.012391	0.026145	0.191206
Ruminococcus gnavus	Noradrenaline	0.010000	death_lin	0.031575	0.039789	0.008214	0.410068	0.954915	3.000000	-0.019162	0.035590	0.228395
Ruminococcus gnavus	Noradrenaline	0.001000	death_lin	0.031575	0.038892	0.007317	0.326643	1.169572	3.000000	-0.012593	0.027228	0.203450
Ruminococcus gnavus	Noradrenaline	0.000100	death_lin	0.031575	0.037236	0.005661	0.489348	0.785772	3.000000	-0.017268	0.028591	0.157413
Ruminococcus gnavus	Noradrenaline	0.000010	death_lin	0.031575	0.034998	0.003423	0.488816	0.786825	3.000000	-0.010423	0.017270	0.095186
Ruminococcus gnavus	Noradrenaline	0.000001	death_lin	0.031575	0.032525	0.000951	0.893347	0.145769	3.000000	-0.019809	0.021710	0.026438
Ruminococcus gnavus	Adrenaline	0.100000	death_lin	0.016926	0.052738	0.035812	0.010933	5.657778	3.000000	0.015668	0.055956	0.995729
Ruminococcus gnavus	Adrenaline	0.010000	death_lin	0.016926	0.051916	0.034991	0.011568	5.544433	3.000000	0.014906	0.055075	0.972890
Ruminococcus gnavus	Adrenaline	0.001000	death_lin	0.016926	0.048748	0.031822	0.044500	3.336611	3.000000	0.001470	0.062174	0.884791
Ruminococcus gnavus	Adrenaline	0.000100	death_lin	0.016926	0.033648	0.016722	0.072156	2.726448	3.000000	-0.002797	0.036241	0.464943
Ruminococcus gnavus	Adrenaline	0.000010	death_lin	0.016926	0.040098	0.023173	0.033721	3.723695	3.000000	0.003368	0.042977	0.644298
Ruminococcus gnavus	Adrenaline	0.000001	death_lin	0.016926	0.021016	0.004090	0.674684	0.463312	3.000000	-0.024002	0.032181	0.113711
Ruminococcus gnavus	Levodopa	0.100000	gr	0.463712	0.290496	-0.173216	0.085799	-2.525002	3.000000	-0.391533	0.045101	-0.972522
Ruminococcus gnavus	Levodopa	0.010000	gr	0.463712	0.345122	-0.118590	0.223639	-1.529423	3.000000	-0.365353	0.128174	-0.665822
Ruminococcus gnavus	Levodopa	0.001000	gr	0.463712	0.316778	-0.146934	0.281384	-1.310311	3.000000	-0.503803	0.209935	-0.824961
Ruminococcus gnavus	Levodopa	0.000100	gr	0.463712	0.285602	-0.178110	0.083954	-2.549843	3.000000	-0.400409	0.044188	-1.000000
Ruminococcus gnavus	Levodopa	0.000010	gr	0.463712	0.304000	-0.159712	0.093284	-2.430547	3.000000	-0.368832	0.049408	-0.896703
Ruminococcus gnavus	Levodopa	0.000001	gr	0.463712	0.296745	-0.166966	0.073028	-2.712213	3.000000	-0.362881	0.028948	-0.937433
Ruminococcus gnavus	Dopamine	0.100000	gr	0.263386	0.304940	0.041554	0.577602	0.622709	3.000000	-0.170813	0.253920	0.233303
Ruminococcus gnavus	Dopamine	0.010000	gr	0.263386	0.327224	0.063838	0.270530	1.347576	3.000000	-0.086923	0.214599	0.358419
Ruminococcus gnavus	Dopamine	0.001000	gr	0.263386	0.388250	0.124864	0.320569	1.187255	3.000000	-0.209835	0.459563	0.701049
Ruminococcus gnavus	Dopamine	0.000100	gr	0.263386	0.277255	0.013869	0.664722	0.478943	3.000000	-0.078286	0.106024	0.077867

Species	Hormon	Concentration	Parameter	Mean_Baseline	Mean_Treatment	Diff	P_Value	T_Statistic	DF	Lower_CI	Upper_CI	Diff_Norm
Ruminococcus gnavus	Dopamine	0.000010	gr	0.263386	0.342815	0.079428	0.171445	1.789672	3.000000	-0.061813	0.220670	0.445951
Ruminococcus gnavus	Dopamine	0.000001	gr	0.263386	0.327050	0.063664	0.214597	1.569279	3.000000	-0.065444	0.192772	0.357440
Ruminococcus gnavus	Noradrenaline	0.100000	gr	0.276846	0.301288	0.024441	0.714450	0.402243	3.000000	-0.168932	0.217815	0.137226
Ruminococcus gnavus	Noradrenaline	0.010000	gr	0.276846	0.272890	-0.003957	0.925242	-0.101932	3.000000	-0.127496	0.119582	-0.022216
Ruminococcus gnavus	Noradrenaline	0.001000	gr	0.276846	0.236992	-0.039854	0.293580	-1.270200	3.000000	-0.139708	0.059999	-0.223762
Ruminococcus gnavus	Noradrenaline	0.000100	gr	0.276846	0.337439	0.060592	0.442248	0.883029	3.000000	-0.157783	0.278967	0.340195
Ruminococcus gnavus	Noradrenaline	0.000010	gr	0.276846	0.310236	0.033390	0.567003	0.641222	3.000000	-0.132327	0.199106	0.187467
Ruminococcus gnavus	Noradrenaline	0.000001	gr	0.276846	0.313527	0.036680	0.427764	0.914775	3.000000	-0.090928	0.164288	0.205941
Ruminococcus gnavus	Adrenaline	0.100000	gr	0.285252	0.233311	-0.051942	0.297026	-1.259182	3.000000	-0.183218	0.079335	-0.291626
Ruminococcus gnavus	Adrenaline	0.010000	gr	0.285252	0.220142	-0.065111	0.298312	-1.255104	3.000000	-0.230206	0.099984	-0.365564
Ruminococcus gnavus	Adrenaline	0.001000	gr	0.285252	0.269260	-0.015992	0.823132	-0.243766	3.000000	-0.224778	0.192794	-0.089789
Ruminococcus gnavus	Adrenaline	0.000100	gr	0.285252	0.326053	0.040801	0.624586	0.543476	3.000000	-0.198118	0.279720	0.229077
Ruminococcus gnavus	Adrenaline	0.000010	gr	0.285252	0.294709	0.009457	0.913587	0.117915	3.000000	-0.245777	0.264691	0.053095
Ruminococcus gnavus	Adrenaline	0.000001	gr	0.285252	0.327457	0.042205	0.714086	0.402794	3.000000	-0.291255	0.375665	0.236961
Ruminococcus gnavus	Levodopa	0.100000	dr	-0.064436	-0.020732	0.043704	0.155442	1.888128	3.000000	-0.029959	0.117367	0.929404
Ruminococcus gnavus	Levodopa	0.010000	dr	-0.064436	-0.020396	0.044040	0.172518	1.783455	3.000000	-0.034546	0.122625	0.936539
Ruminococcus gnavus	Levodopa	0.001000	dr	-0.064436	-0.020183	0.044252	0.175811	1.764649	3.000000	-0.035554	0.124059	0.941064
Ruminococcus gnavus	Levodopa	0.000100	dr	-0.064436	-0.018390	0.046046	0.169947	1.798427	3.000000	-0.035435	0.127527	0.979199
Ruminococcus gnavus	Levodopa	0.000010	dr	-0.064436	-0.017412	0.047024	0.154909	1.891605	3.000000	-0.032089	0.126137	1.000000
Ruminococcus gnavus	Levodopa	0.000001	dr	-0.064436	-0.025355	0.039081	0.215063	1.567181	3.000000	-0.040280	0.118442	0.831090
Ruminococcus gnavus	Dopamine	0.100000	dr	-0.008548	-0.023249	-0.014701	0.168260	-1.808395	3.000000	-0.040572	0.011170	-0.312630
Ruminococcus gnavus	Dopamine	0.010000	dr	-0.008548	-0.018312	-0.009765	0.371498	-1.048327	3.000000	-0.039408	0.019878	-0.207654
Ruminococcus gnavus	Dopamine	0.001000	dr	-0.008548	-0.018937	-0.010389	0.309776	-1.219531	3.000000	-0.037501	0.016722	-0.220939
Ruminococcus gnavus	Dopamine	0.000100	dr	-0.008548	-0.017985	-0.009437	0.295197	-1.265014	3.000000	-0.033177	0.014304	-0.200681
Ruminococcus gnavus	Dopamine	0.000010	dr	-0.008548	-0.027535	-0.018987	0.039589	-3.496201	3.000000	-0.036271	-0.001704	-0.403782
Ruminococcus gnavus	Dopamine	0.000001	dr	-0.008548	-0.017628	-0.009080	0.445827	-0.875328	3.000000	-0.042095	0.023934	-0.193104
Ruminococcus gnavus	Noradrenaline	0.100000	dr	-0.026526	-0.020383	0.006143	0.431561	0.906361	3.000000	-0.015426	0.027712	0.130632

Species	Hormon	Concentration	Parameter	Mean_Baseline	Mean_Treatment	Diff	P_Value	T_Statistic	DF	Lower_CI	Upper_CI	Diff_Norm
Ruminococcus gnavus	Noradrenaline	0.010000	dr	-0.026526	-0.015272	0.011253	0.240655	1.459016	3.000000	-0.013293	0.035800	0.239313
Ruminococcus gnavus	Noradrenaline	0.001000	dr	-0.026526	-0.014554	0.011971	0.162174	1.845342	3.000000	-0.008674	0.032617	0.254580
Ruminococcus gnavus	Noradrenaline	0.000100	dr	-0.026526	-0.018647	0.007879	0.349684	1.105370	3.000000	-0.014805	0.030563	0.167553
Ruminococcus gnavus	Noradrenaline	0.000010	dr	-0.026526	-0.019502	0.007023	0.381340	1.023647	3.000000	-0.014812	0.028859	0.149359
Ruminococcus gnavus	Noradrenaline	0.000001	dr	-0.026526	-0.020933	0.005593	0.460332	0.844669	3.000000	-0.015480	0.026666	0.118941
Ruminococcus gnavus	Adrenaline	0.100000	dr	-0.022560	-0.013594	0.008965	0.372864	1.044863	3.000000	-0.018341	0.036272	0.190655
Ruminococcus gnavus	Adrenaline	0.010000	dr	-0.022560	-0.015979	0.006581	0.525038	0.717221	3.000000	-0.022620	0.035782	0.139948
Ruminococcus gnavus	Adrenaline	0.001000	dr	-0.022560	-0.017890	0.004669	0.673188	0.465650	3.000000	-0.027241	0.036579	0.099292
Ruminococcus gnavus	Adrenaline	0.000100	dr	-0.022560	-0.019473	0.003087	0.720487	0.393142	3.000000	-0.021899	0.028072	0.065639
Ruminococcus gnavus	Adrenaline	0.000010	dr	-0.022560	-0.019290	0.003270	0.739634	0.364542	3.000000	-0.025276	0.031816	0.069537
Ruminococcus gnavus	Adrenaline	0.000001	dr	-0.022560	-0.012608	0.009951	0.449388	0.867720	3.000000	-0.026546	0.046448	0.211618
Ruminococcus gnavus	Levodopa	0.100000	td	1.671371	2.406801	0.735430	0.101249	2.339706	3.000000	-0.264895	1.735755	0.891590
Ruminococcus gnavus	Levodopa	0.010000	td	1.671371	2.141596	0.470225	0.401166	0.975716	3.000000	-1.063485	2.003935	0.570071
Ruminococcus gnavus	Levodopa	0.001000	td	1.671371	2.266574	0.595203	0.385842	1.012559	3.000000	-1.275504	2.465910	0.721587
Ruminococcus gnavus	Levodopa	0.000100	td	1.671371	2.496223	0.824853	0.134013	2.040261	3.000000	-0.461772	2.111477	1.000000
Ruminococcus gnavus	Levodopa	0.000010	td	1.671371	2.315037	0.643666	0.120225	2.154251	3.000000	-0.307213	1.594546	0.780341
Ruminococcus gnavus	Levodopa	0.000001	td	1.671371	2.407289	0.735919	0.051592	3.141792	3.000000	-0.009523	1.481360	0.892182
Ruminococcus gnavus	Dopamine	0.100000	td	2.749907	2.369954	-0.379952	0.554891	-0.662698	3.000000	-2.204581	1.444676	-0.460631
Ruminococcus gnavus	Dopamine	0.010000	td	2.749907	2.155027	-0.594880	0.264469	-1.369081	3.000000	-1.977685	0.787925	-0.721195
Ruminococcus gnavus	Dopamine	0.001000	td	2.749907	2.001523	-0.748384	0.338682	-1.135483	3.000000	-2.845897	1.349130	-0.907294
Ruminococcus gnavus	Dopamine	0.000100	td	2.749907	2.547400	-0.202507	0.515849	-0.734496	3.000000	-1.079935	0.674921	-0.245507
Ruminococcus gnavus	Dopamine	0.000010	td	2.749907	2.175983	-0.573924	0.251453	-1.417098	3.000000	-1.862813	0.714965	-0.695790
Ruminococcus gnavus	Dopamine	0.000001	td	2.749907	2.295877	-0.454029	0.253910	-1.407832	3.000000	-1.480376	0.572317	-0.550437
Ruminococcus gnavus	Noradrenaline	0.100000	td	2.635396	2.375392	-0.260004	0.601919	-0.581155	3.000000	-1.683804	1.163796	-0.315212
Ruminococcus gnavus	Noradrenaline	0.010000	td	2.635396	2.567375	-0.068021	0.841764	-0.217512	3.000000	-1.063244	0.927203	-0.082464
Ruminococcus gnavus	Noradrenaline	0.001000	td	2.635396	2.967426	0.332030	0.239774	1.462527	3.000000	-0.390465	1.054526	0.402533
Ruminococcus gnavus	Noradrenaline	0.000100	td	2.635396	2.115302	-0.520094	0.361101	-1.075095	3.000000	-2.059650	1.019463	-0.630529

Species	Hormon	Concentration	Parameter	Mean_Baseline	Mean_Treatment	Diff	P_Value	T_Statistic	DF	Lower_CI	Upper_CI	Diff_Norm
Ruminococcus gnavus	Noradrenaline	0.000010	td	2.635396	2.357859	-0.277537	0.479617	-0.805182	3.000000	-1.374488	0.819415	-0.336468
Ruminococcus gnavus	Noradrenaline	0.000001	td	2.635396	2.268930	-0.366465	0.337969	-1.137466	3.000000	-1.391776	0.658845	-0.444280
Ruminococcus gnavus	Adrenaline	0.100000	td	2.789735	3.111621	0.321886	0.133495	2.044287	3.000000	-0.179211	0.822983	0.390235
Ruminococcus gnavus	Adrenaline	0.010000	td	2.789735	3.229763	0.440028	0.155831	1.885596	3.000000	-0.302636	1.182692	0.533462
Ruminococcus gnavus	Adrenaline	0.001000	td	2.789735	2.609161	-0.180574	0.699448	-0.425046	3.000000	-1.532585	1.171438	-0.218916
Ruminococcus gnavus	Adrenaline	0.000100	td	2.789735	2.292098	-0.497636	0.345605	-1.116425	3.000000	-1.916184	0.920911	-0.603303
Ruminococcus gnavus	Adrenaline	0.000010	td	2.789735	2.446393	-0.343342	0.562346	-0.649437	3.000000	-2.025827	1.339143	-0.416247
Ruminococcus gnavus	Adrenaline	0.000001	td	2.789735	2.454159	-0.335576	0.595492	-0.592019	3.000000	-2.139492	1.468340	-0.406831
Ruminococcus gnavus	Levodopa	0.100000	lagC	9.005531	10.533078	1.527546	0.336423	1.141785	3.000000	-2.730115	5.785208	1.000000
Ruminococcus gnavus	Levodopa	0.010000	lagC	9.005531	10.471648	1.466117	0.318082	1.194594	3.000000	-2.439678	5.371913	0.959786
Ruminococcus gnavus	Levodopa	0.001000	lagC	9.005531	9.867246	0.861715	0.418592	0.935387	3.000000	-2.070079	3.793509	0.564117
Ruminococcus gnavus	Levodopa	0.000100	lagC	9.005531	9.873546	0.868014	0.588907	0.603236	3.000000	-3.711307	5.447336	0.568241
Ruminococcus gnavus	Levodopa	0.000010	lagC	9.005531	9.576444	0.570913	0.717276	0.397977	3.000000	-3.994428	5.136254	0.373745
Ruminococcus gnavus	Levodopa	0.000001	lagC	9.005531	10.169872	1.164341	0.551009	0.669656	3.000000	-4.369029	6.697710	0.762229
Ruminococcus gnavus	Dopamine	0.100000	lagC	9.005284	10.478397	1.473112	0.078952	2.620722	3.000000	-0.315747	3.261971	0.964365
Ruminococcus gnavus	Dopamine	0.010000	lagC	9.005284	9.611734	0.606450	0.092916	2.434970	3.000000	-0.186165	1.399065	0.397009
Ruminococcus gnavus	Dopamine	0.001000	lagC	9.005284	8.577880	-0.427404	0.667842	-0.474033	3.000000	-3.296807	2.441999	-0.279798
Ruminococcus gnavus	Dopamine	0.000100	lagC	9.005284	9.456343	0.451058	0.063406	2.882372	3.000000	-0.046958	0.949075	0.295283
Ruminococcus gnavus	Dopamine	0.000010	lagC	9.005284	8.819546	-0.185738	0.812002	-0.259551	3.000000	-2.463137	2.091660	-0.121593
Ruminococcus gnavus	Dopamine	0.000001	lagC	9.005284	8.470802	-0.534482	0.442696	-0.882062	3.000000	-2.462874	1.393909	-0.349896
Ruminococcus gnavus	Noradrenaline	0.100000	lagC	8.472146	9.690945	1.218799	0.058958	2.972338	3.000000	-0.086154	2.523753	0.797881
Ruminococcus gnavus	Noradrenaline	0.010000	lagC	8.472146	8.829510	0.357365	0.268161	1.355920	3.000000	-0.481397	1.196127	0.233947
Ruminococcus gnavus	Noradrenaline	0.001000	lagC	8.472146	8.634685	0.162539	0.448309	0.870020	3.000000	-0.432013	0.757091	0.106405
Ruminococcus gnavus	Noradrenaline	0.000100	lagC	8.472146	9.196080	0.723935	0.114902	2.202515	3.000000	-0.322089	1.769959	0.473920
Ruminococcus gnavus	Noradrenaline	0.000010	lagC	8.472146	8.941472	0.469326	0.342520	1.124871	3.000000	-0.858475	1.797128	0.307242
Ruminococcus gnavus	Noradrenaline	0.000001	lagC	8.472146	9.072882	0.600737	0.145866	1.952855	3.000000	-0.378246	1.579720	0.393269
Ruminococcus gnavus	Adrenaline	0.100000	lagC	8.643037	7.221527	-1.421511	0.069288	-2.774837	3.000000	-3.051834	0.208813	-0.930584

Species	Hormon	Concentration	Parameter	Mean_Baseline	Mean_Treatment	Diff	P_Value	T_Statistic	DF	Lower_CI	Upper_CI	Diff_Norm
Ruminococcus gnavus	Adrenaline	0.010000	lagC	8.643037	7.760947	-0.882091	0.003138	-8.754811	3.000000	-1.202738	-0.561443	-0.577456
Ruminococcus gnavus	Adrenaline	0.001000	lagC	8.643037	8.664922	0.021885	0.959973	0.054487	3.000000	-1.256360	1.300130	0.014327
Ruminococcus gnavus	Adrenaline	0.000100	lagC	8.643037	8.935834	0.292797	0.604915	0.576119	3.000000	-1.324597	1.910191	0.191678
Ruminococcus gnavus	Adrenaline	0.000010	lagC	8.643037	9.009948	0.366911	0.631874	0.531560	3.000000	-1.829779	2.563601	0.240196
Ruminococcus gnavus	Adrenaline	0.000001	lagC	8.643037	7.987844	-0.655194	0.219887	-1.545742	3.000000	-2.004137	0.693749	-0.428919
Ruminococcus gnavus	Levodopa	0.100000	t_k	17.583333	20.916667	3.333333	0.228098	1.510420	3.000000	-3.689981	10.356647	0.261438
Ruminococcus gnavus	Levodopa	0.010000	t_k	17.583333	20.416667	2.833333	0.232292	1.492914	3.000000	-3.206487	8.873154	0.222222
Ruminococcus gnavus	Levodopa	0.001000	t_k	17.583333	20.833333	3.250000	0.304423	1.235969	3.000000	-5.118292	11.618292	0.254902
Ruminococcus gnavus	Levodopa	0.000100	t_k	17.583333	19.500000	1.916667	0.396148	0.987631	3.000000	-4.259414	8.092748	0.150327
Ruminococcus gnavus	Levodopa	0.000010	t_k	17.583333	19.833333	2.250000	0.483701	0.796996	3.000000	-6.734372	11.234372	0.176471
Ruminococcus gnavus	Levodopa	0.000001	t_k	17.583333	20.583333	3.000000	0.197961	1.647751	3.000000	-2.794163	8.794163	0.235294
Ruminococcus gnavus	Dopamine	0.100000	t_k	30.833333	30.250000	-0.583333	0.573717	-0.629465	3.000000	-3.532547	2.365880	-0.045752
Ruminococcus gnavus	Dopamine	0.010000	t_k	30.833333	19.916667	-10.916667	0.232729	-1.491109	3.000000	-34.215899	12.382565	-0.856209
Ruminococcus gnavus	Dopamine	0.001000	t_k	30.833333	18.083333	-12.750000	0.186615	-1.705660	3.000000	-36.539146	11.039146	-1.000000
Ruminococcus gnavus	Dopamine	0.000100	t_k	30.833333	20.916667	-9.916667	0.305975	-1.231174	3.000000	-35.550144	15.716810	-0.777778
Ruminococcus gnavus	Dopamine	0.000010	t_k	30.833333	19.250000	-11.583333	0.202481	-1.625722	3.000000	-34.258392	11.091726	-0.908497
Ruminococcus gnavus	Dopamine	0.000001	t_k	30.833333	27.833333	-3.000000	0.808099	-0.265108	3.000000	-39.013083	33.013083	-0.235294
Ruminococcus gnavus	Noradrenaline	0.100000	t_k	23.416667	19.916667	-3.500000	0.142679	-1.975514	3.000000	-9.138311	2.138311	-0.274510
Ruminococcus gnavus	Noradrenaline	0.010000	t_k	23.416667	21.916667	-1.500000	0.218668	-1.551109	3.000000	-4.577584	1.577584	-0.117647
Ruminococcus gnavus	Noradrenaline	0.001000	t_k	23.416667	21.500000	-1.916667	0.095679	-2.402272	3.000000	-4.455800	0.622467	-0.150327
Ruminococcus gnavus	Noradrenaline	0.000100	t_k	23.416667	22.000000	-1.416667	0.334562	-1.147009	3.000000	-5.347294	2.513961	-0.111111
Ruminococcus gnavus	Noradrenaline	0.000010	t_k	23.416667	20.666667	-2.750000	0.068286	-2.792335	3.000000	-5.884197	0.384197	-0.215686
Ruminococcus gnavus	Noradrenaline	0.000001	t_k	23.416667	20.750000	-2.666667	0.023485	-4.276180	3.000000	-4.651271	-0.682063	-0.209150
Ruminococcus gnavus	Adrenaline	0.100000	t_k	21.833333	18.333333	-3.500000	0.012308	-5.422177	3.000000	-5.554260	-1.445740	-0.274510
Ruminococcus gnavus	Adrenaline	0.010000	t_k	21.833333	20.250000	-1.583333	0.329513	-1.161332	3.000000	-5.922207	2.755540	-0.124183
Ruminococcus gnavus	Adrenaline	0.001000	t_k	21.833333	20.333333	-1.500000	0.544375	-0.681636	3.000000	-8.503257	5.503257	-0.117647
Ruminococcus gnavus	Adrenaline	0.000100	t_k	21.833333	20.583333	-1.250000	0.293878	-1.269243	3.000000	-4.384197	1.884197	-0.098039

Species	Hormon	Concentration	Parameter	Mean_Baseline	Mean_Treatment	Diff	P_Value	T_Statistic	DF	Lower_CI	Upper_CI	Diff_Norm
Ruminococcus gnavus	Adrenaline	0.000010	t_k	21.833333	19.583333	-2.250000	0.175073	-1.768829	3.000000	-6.298161	1.798161	-0.176471
Ruminococcus gnavus	Adrenaline	0.000001	t_k	21.833333	20.750000	-1.083333	0.041424	-3.433759	3.000000	-2.087379	-0.079288	-0.084967
Ruminococcus gnavus	Levodopa	0.100000	t_gr	10.666667	12.250000	1.583333	0.333526	1.149932	3.000000	-2.798554	5.965220	0.904762
Ruminococcus gnavus	Levodopa	0.010000	t_gr	10.666667	12.416667	1.750000	0.275354	1.330821	3.000000	-2.434846	5.934846	1.000000
Ruminococcus gnavus	Levodopa	0.001000	t_gr	10.666667	11.916667	1.250000	0.318932	1.192079	3.000000	-2.087075	4.587075	0.714286
Ruminococcus gnavus	Levodopa	0.000100	t_gr	10.666667	12.250000	1.583333	0.368450	1.056099	3.000000	-3.187880	6.354547	0.904762
Ruminococcus gnavus	Levodopa	0.000010	t_gr	10.666667	11.916667	1.250000	0.462231	0.840718	3.000000	-3.481740	5.981740	0.714286
Ruminococcus gnavus	Levodopa	0.000001	t_gr	10.666667	12.333333	1.666667	0.418064	0.936586	3.000000	-3.996538	7.329871	0.952381
Ruminococcus gnavus	Dopamine	0.100000	t_gr	11.333333	12.250000	0.916667	0.022152	4.370957	3.000000	0.249252	1.584082	0.523810
Ruminococcus gnavus	Dopamine	0.010000	t_gr	11.333333	11.583333	0.250000	0.215170	1.566699	3.000000	-0.257827	0.757827	0.142857
Ruminococcus gnavus	Dopamine	0.001000	t_gr	11.333333	10.416667	-0.916667	0.551360	-0.669026	3.000000	-5.277100	3.443767	-0.523810
Ruminococcus gnavus	Dopamine	0.000100	t_gr	11.333333	11.833333	0.500000	0.102728	2.323790	3.000000	-0.184753	1.184753	0.285714
Ruminococcus gnavus	Dopamine	0.000010	t_gr	11.333333	10.750000	-0.583333	0.523167	-0.720718	3.000000	-3.159135	1.992468	-0.333333
Ruminococcus gnavus	Dopamine	0.000001	t_gr	11.333333	10.500000	-0.833333	0.334538	-1.147079	3.000000	-3.145327	1.478660	-0.476190
Ruminococcus gnavus	Noradrenaline	0.100000	t_gr	10.666667	11.583333	0.916667	0.001609	11.000000	3.000000	0.651463	1.181871	0.523810
Ruminococcus gnavus	Noradrenaline	0.010000	t_gr	10.666667	11.083333	0.416667	0.015392	5.000000	3.000000	0.151463	0.681871	0.238095
Ruminococcus gnavus	Noradrenaline	0.001000	t_gr	10.666667	11.250000	0.583333	0.005986	7.000000	3.000000	0.318129	0.848537	0.333333
Ruminococcus gnavus	Noradrenaline	0.000100	t_gr	10.666667	11.250000	0.583333	0.005986	7.000000	3.000000	0.318129	0.848537	0.333333
Ruminococcus gnavus	Noradrenaline	0.000010	t_gr	10.666667	11.166667	0.500000	0.102728	2.323790	3.000000	-0.184753	1.184753	0.285714
Ruminococcus gnavus	Noradrenaline	0.000001	t_gr	10.666667	11.250000	0.583333	0.035353	3.655631	3.000000	0.075507	1.091160	0.333333
Ruminococcus gnavus	Adrenaline	0.100000	t_gr	10.916667	9.416667	-1.500000	0.062772	-2.894704	3.000000	-3.149105	0.149105	-0.857143
Ruminococcus gnavus	Adrenaline	0.010000	t_gr	10.916667	10.666667	-0.250000	0.318932	-1.192079	3.000000	-0.917415	0.417415	-0.142857
Ruminococcus gnavus	Adrenaline	0.001000	t_gr	10.916667	11.083333	0.166667	0.391002	1.000000	3.000000	-0.363741	0.697074	0.095238
Ruminococcus gnavus	Adrenaline	0.000100	t_gr	10.916667	10.916667	-0.000000	1.000000	-0.000000	3.000000	-0.968388	0.968388	-0.000000
Ruminococcus gnavus	Adrenaline	0.000010	t_gr	10.916667	11.166667	0.250000	0.570656	0.634811	3.000000	-1.003305	1.503305	0.142857
Ruminococcus gnavus	Adrenaline	0.000001	t_gr	10.916667	10.166667	-0.750000	0.410465	-0.953998	3.000000	-3.251928	1.751928	-0.428571
Ruminococcus gnavus	Levodopa	0.100000	t_dr	45.250000	48.000000	2.750000	0.391002	1.000000	3.000000	-6.001727	11.501727	0.172775

Species	Hormon	Concentration	Parameter	Mean_Baseline	Mean_Treatment	Diff	P_Value	T_Statistic	DF	Lower_CI	Upper_CI	Diff_Norm
Ruminococcus gnavus	Levodopa	0.010000	t_dr	45.250000	48.000000	2.750000	0.391002	1.000000	3.000000	-6.001727	11.501727	0.172775
Ruminococcus gnavus	Levodopa	0.001000	t_dr	45.250000	48.000000	2.750000	0.391002	1.000000	3.000000	-6.001727	11.501727	0.172775
Ruminococcus gnavus	Levodopa	0.000100	t_dr	45.250000	48.000000	2.750000	0.391002	1.000000	3.000000	-6.001727	11.501727	0.172775
Ruminococcus gnavus	Levodopa	0.000010	t_dr	45.250000	40.833333	-4.416667	0.638823	-0.520283	3.000000	-31.432337	22.599004	-0.277487
Ruminococcus gnavus	Levodopa	0.000001	t_dr	45.250000	48.000000	2.750000	0.391002	1.000000	3.000000	-6.001727	11.501727	0.172775
Ruminococcus gnavus	Dopamine	0.100000	t_dr	32.083333	48.000000	15.916667	0.075124	2.678828	3.000000	-2.992325	34.825658	1.000000
Ruminococcus gnavus	Dopamine	0.010000	t_dr	32.083333	48.000000	15.916667	0.075124	2.678828	3.000000	-2.992325	34.825658	1.000000
Ruminococcus gnavus	Dopamine	0.001000	t_dr	32.083333	34.333333	2.250000	0.816289	0.253461	3.000000	-26.000954	30.500954	0.141361
Ruminococcus gnavus	Dopamine	0.000100	t_dr	32.083333	46.666667	14.583333	0.134369	2.037503	3.000000	-8.194884	37.361551	0.916230
Ruminococcus gnavus	Dopamine	0.000010	t_dr	32.083333	39.583333	7.500000	0.379888	1.027247	3.000000	-15.735249	30.735249	0.471204
Ruminococcus gnavus	Dopamine	0.000001	t_dr	32.083333	48.000000	15.916667	0.075124	2.678828	3.000000	-2.992325	34.825658	1.000000
Ruminococcus gnavus	Noradrenaline	0.100000	t_dr	46.333333	48.000000	1.666667	0.391002	1.000000	3.000000	-3.637411	6.970744	0.104712
Ruminococcus gnavus	Noradrenaline	0.010000	t_dr	46.333333	48.000000	1.666667	0.391002	1.000000	3.000000	-3.637411	6.970744	0.104712
Ruminococcus gnavus	Noradrenaline	0.001000	t_dr	46.333333	48.000000	1.666667	0.391002	1.000000	3.000000	-3.637411	6.970744	0.104712
Ruminococcus gnavus	Noradrenaline	0.000100	t_dr	46.333333	48.000000	1.666667	0.391002	1.000000	3.000000	-3.637411	6.970744	0.104712
Ruminococcus gnavus	Noradrenaline	0.000010	t_dr	46.333333	48.000000	1.666667	0.391002	1.000000	3.000000	-3.637411	6.970744	0.104712
Ruminococcus gnavus	Noradrenaline	0.000001	t_dr	46.333333	48.000000	1.666667	0.391002	1.000000	3.000000	-3.637411	6.970744	0.104712
Ruminococcus gnavus	Adrenaline	0.100000	t_dr	46.083333	48.000000	1.916667	0.187060	1.703314	3.000000	-1.664404	5.497737	0.120419
Ruminococcus gnavus	Adrenaline	0.010000	t_dr	46.083333	48.000000	1.916667	0.187060	1.703314	3.000000	-1.664404	5.497737	0.120419
Ruminococcus gnavus	Adrenaline	0.001000	t_dr	46.083333	42.083333	-4.000000	0.495574	-0.773523	3.000000	-20.456891	12.456891	-0.251309
Ruminococcus gnavus	Adrenaline	0.000100	t_dr	46.083333	48.000000	1.916667	0.187060	1.703314	3.000000	-1.664404	5.497737	0.120419
Ruminococcus gnavus	Adrenaline	0.000010	t_dr	46.083333	48.000000	1.916667	0.187060	1.703314	3.000000	-1.664404	5.497737	0.120419
Ruminococcus gnavus	Adrenaline	0.000001	t_dr	46.083333	46.250000	0.166667	0.900678	0.135665	3.000000	-3.743030	4.076363	0.010471
Ruminococcus lactaris	Levodopa	0.100000	auc_lin	16.470533	16.116328	-0.354205	0.548131	-0.674840	3.000000	-2.024583	1.316173	-0.256008
Ruminococcus lactaris	Levodopa	0.010000	auc_lin	16.470533	16.526677	0.056145	0.937816	0.084727	3.000000	-2.052715	2.165005	0.040580
Ruminococcus lactaris	Levodopa	0.001000	auc_lin	16.470533	15.791207	-0.679325	0.275592	-1.330004	3.000000	-2.304822	0.946171	-0.490996
Ruminococcus lactaris	Levodopa	0.000100	auc_lin	16.470533	16.168723	-0.301810	0.675461	-0.462098	3.000000	-2.380356	1.776736	-0.218139

Species	Hormon	Concentration	Parameter	Mean_Baseline	Mean_Treatment	Diff	P_Value	T_Statistic	DF	Lower_CI	Upper_CI	Diff_Norm
Ruminococcus lactaris	Levodopa	0.000010	auc_lin	16.470533	16.511654	0.041122	0.954223	0.062327	3.000000	-2.058590	2.140834	0.029722
Ruminococcus lactaris	Levodopa	0.000001	auc_lin	16.470533	16.805910	0.335377	0.595917	0.591297	3.000000	-1.469673	2.140427	0.242401
Ruminococcus lactaris	Dopamine	0.100000	auc_lin	15.961270	16.616693	0.655422	0.503064	0.758954	3.000000	-2.092895	3.403739	0.473719
Ruminococcus lactaris	Dopamine	0.010000	auc_lin	15.961270	16.601344	0.640074	0.513039	0.739830	3.000000	-2.113264	3.393412	0.462626
Ruminococcus lactaris	Dopamine	0.001000	auc_lin	15.961270	16.414447	0.453177	0.595039	0.592787	3.000000	-1.979757	2.886111	0.327542
Ruminococcus lactaris	Dopamine	0.000100	auc_lin	15.961270	16.611447	0.650177	0.374856	1.039836	3.000000	-1.339707	2.640060	0.469928
Ruminococcus lactaris	Dopamine	0.000010	auc_lin	15.961270	17.276447	1.315177	0.239760	1.462584	3.000000	-1.546526	4.176879	0.950570
Ruminococcus lactaris	Dopamine	0.000001	auc_lin	15.961270	17.344837	1.383567	0.171381	1.790048	3.000000	-1.076215	3.843349	1.000000
Ruminococcus lactaris	Noradrenaline	0.100000	auc_lin	16.166641	16.226639	0.059998	0.842655	0.216261	3.000000	-0.822925	0.942922	0.043365
Ruminococcus lactaris	Noradrenaline	0.010000	auc_lin	16.166641	16.237705	0.071064	0.805519	0.268786	3.000000	-0.770341	0.912470	0.051363
Ruminococcus lactaris	Noradrenaline	0.001000	auc_lin	16.166641	15.509533	-0.657108	0.005044	-7.430452	3.000000	-0.938546	-0.375670	-0.474938
Ruminococcus lactaris	Noradrenaline	0.000100	auc_lin	16.166641	16.163955	-0.002686	0.993216	-0.009229	3.000000	-0.928780	0.923409	-0.001941
Ruminococcus lactaris	Noradrenaline	0.000010	auc_lin	16.166641	15.582614	-0.584027	0.095479	-2.404595	3.000000	-1.356977	0.188924	-0.422117
Ruminococcus lactaris	Noradrenaline	0.000001	auc_lin	16.166641	16.413100	0.246459	0.045781	3.298600	3.000000	0.008679	0.484239	0.178133
Ruminococcus lactaris	Adrenaline	0.100000	auc_lin	16.086978	16.740942	0.653964	0.399008	0.980824	3.000000	-1.467931	2.775859	0.472665
Ruminococcus lactaris	Adrenaline	0.010000	auc_lin	16.086978	16.185329	0.098351	0.905905	0.128471	3.000000	-2.337982	2.534684	0.071085
Ruminococcus lactaris	Adrenaline	0.001000	auc_lin	16.086978	15.609932	-0.477046	0.504449	-0.756282	3.000000	-2.484463	1.530371	-0.344794
Ruminococcus lactaris	Adrenaline	0.000100	auc_lin	16.086978	15.822297	-0.264681	0.728118	-0.381696	3.000000	-2.471491	1.942130	-0.191303
Ruminococcus lactaris	Adrenaline	0.000010	auc_lin	16.086978	16.255656	0.168678	0.861179	0.190362	3.000000	-2.651265	2.988622	0.121916
Ruminococcus lactaris	Adrenaline	0.000001	auc_lin	16.086978	16.792649	0.705671	0.385982	1.012216	3.000000	-1.512985	2.924326	0.510037
Ruminococcus lactaris	Levodopa	0.100000	k_lin	0.456445	0.417344	-0.039101	0.047188	-3.258426	3.000000	-0.077290	-0.000912	-0.654840
Ruminococcus lactaris	Levodopa	0.010000	k_lin	0.456445	0.421525	-0.034920	0.138941	-2.002854	3.000000	-0.090407	0.020566	-0.584820
Ruminococcus lactaris	Levodopa	0.001000	k_lin	0.456445	0.405397	-0.051048	0.035356	-3.655501	3.000000	-0.095489	-0.006606	-0.854912
Ruminococcus lactaris	Levodopa	0.000100	k_lin	0.456445	0.415683	-0.040761	0.060906	-2.931920	3.000000	-0.085006	0.003483	-0.682645
Ruminococcus lactaris	Levodopa	0.000010	k_lin	0.456445	0.425663	-0.030781	0.116564	-2.187165	3.000000	-0.075570	0.014007	-0.515506
Ruminococcus lactaris	Levodopa	0.000001	k_lin	0.456445	0.442763	-0.013682	0.354054	-1.093671	3.000000	-0.053496	0.026131	-0.229141
Ruminococcus lactaris	Dopamine	0.100000	k_lin	0.472896	0.438323	-0.034572	0.075380	-2.674833	3.000000	-0.075706	0.006561	-0.578995

Species	Hormon	Concentration	Parameter	Mean_Baseline	Mean_Treatment	Diff	P_Value	T_Statistic	DF	Lower_CI	Upper_CI	Diff_Norm
Ruminococcus lactaris	Dopamine	0.010000	k_lin	0.472896	0.424018	-0.048878	0.112860	-2.221745	3.000000	-0.118891	0.021135	-0.818576
Ruminococcus lactaris	Dopamine	0.001000	k_lin	0.472896	0.418399	-0.054496	0.010063	-5.827828	3.000000	-0.084255	-0.024737	-0.912667
Ruminococcus lactaris	Dopamine	0.000100	k_lin	0.472896	0.426733	-0.046162	0.014475	-5.113187	3.000000	-0.074894	-0.017431	-0.773099
Ruminococcus lactaris	Dopamine	0.000010	k_lin	0.472896	0.447545	-0.025351	0.204146	-1.617743	3.000000	-0.075222	0.024520	-0.424560
Ruminococcus lactaris	Dopamine	0.000001	k_lin	0.472896	0.461332	-0.011563	0.559919	-0.653739	3.000000	-0.067854	0.044728	-0.193655
Ruminococcus lactaris	Noradrenaline	0.100000	k_lin	0.456512	0.428005	-0.028507	0.006876	-6.668376	3.000000	-0.042112	-0.014902	-0.477419
Ruminococcus lactaris	Noradrenaline	0.010000	k_lin	0.456512	0.421105	-0.035407	0.044137	-3.347620	3.000000	-0.069068	-0.001747	-0.592979
Ruminococcus lactaris	Noradrenaline	0.001000	k_lin	0.456512	0.396801	-0.059711	0.000361	-18.208138	3.000000	-0.070147	-0.049275	-1.000000
Ruminococcus lactaris	Noradrenaline	0.000100	k_lin	0.456512	0.415218	-0.041295	0.014192	-5.149960	3.000000	-0.066813	-0.015776	-0.691574
Ruminococcus lactaris	Noradrenaline	0.000010	k_lin	0.456512	0.406845	-0.049667	0.004086	-7.993568	3.000000	-0.069441	-0.029894	-0.831798
Ruminococcus lactaris	Noradrenaline	0.000001	k_lin	0.456512	0.435977	-0.020536	0.000284	-19.733381	3.000000	-0.023847	-0.017224	-0.343915
Ruminococcus lactaris	Adrenaline	0.100000	k_lin	0.454824	0.443173	-0.011651	0.514222	-0.737582	3.000000	-0.061921	0.038619	-0.195122
Ruminococcus lactaris	Adrenaline	0.010000	k_lin	0.454824	0.419650	-0.035174	0.105854	-2.291051	3.000000	-0.084033	0.013685	-0.589066
Ruminococcus lactaris	Adrenaline	0.001000	k_lin	0.454824	0.403483	-0.051341	0.023505	-4.274825	3.000000	-0.089563	-0.013120	-0.859827
Ruminococcus lactaris	Adrenaline	0.000100	k_lin	0.454824	0.406339	-0.048485	0.053004	-3.107027	3.000000	-0.098146	0.001177	-0.811988
Ruminococcus lactaris	Adrenaline	0.000010	k_lin	0.454824	0.418449	-0.036375	0.163147	-1.839330	3.000000	-0.099312	0.026562	-0.609188
Ruminococcus lactaris	Adrenaline	0.000001	k_lin	0.454824	0.447182	-0.007642	0.653062	-0.497425	3.000000	-0.056535	0.041251	-0.127986
Ruminococcus lactaris	Levodopa	0.100000	death_lin	0.001284	0.010950	0.009666	0.243179	1.449037	3.000000	-0.011564	0.030896	0.282616
Ruminococcus lactaris	Levodopa	0.010000	death_lin	0.001284	0.019965	0.018681	0.005814	7.071726	3.000000	0.010274	0.027088	0.546169
Ruminococcus lactaris	Levodopa	0.001000	death_lin	0.001284	0.026866	0.025583	0.010682	5.704898	3.000000	0.011311	0.039854	0.747953
Ruminococcus lactaris	Levodopa	0.000100	death_lin	0.001284	0.024685	0.023402	0.009727	5.898814	3.000000	0.010776	0.036027	0.684185
Ruminococcus lactaris	Levodopa	0.000010	death_lin	0.001284	0.013051	0.011767	0.099845	2.355068	3.000000	-0.004134	0.027669	0.344041
Ruminococcus lactaris	Levodopa	0.000001	death_lin	0.001284	0.010942	0.009659	0.239019	1.465544	3.000000	-0.011315	0.030632	0.282386
Ruminococcus lactaris	Dopamine	0.100000	death_lin	0.000659	0.034863	0.034204	0.000040	38.070284	3.000000	0.031344	0.037063	1.000000
Ruminococcus lactaris	Dopamine	0.010000	death_lin	0.000659	0.024213	0.023554	0.035111	3.665449	3.000000	0.003104	0.044004	0.688634
Ruminococcus lactaris	Dopamine	0.001000	death_lin	0.000659	0.016000	0.015341	0.032819	3.763179	3.000000	0.002367	0.028314	0.448508
Ruminococcus lactaris	Dopamine	0.000100	death_lin	0.000659	0.019084	0.018425	0.008605	6.161184	3.000000	0.008908	0.027942	0.538693

Species	Hormon	Concentration	Parameter	Mean_Baseline	Mean_Treatment	Diff	P_Value	T_Statistic	DF	Lower_CI	Upper_CI	Diff_Norm
Ruminococcus lactaris	Dopamine	0.000010	death_lin	0.000659	0.007385	0.006726	0.064002	2.870914	3.000000	-0.000730	0.014181	0.196637
Ruminococcus lactaris	Dopamine	0.000001	death_lin	0.000659	0.004234	0.003575	0.265311	1.366063	3.000000	-0.004754	0.011904	0.104529
Ruminococcus lactaris	Noradrenaline	0.100000	death_lin	0.004423	0.022114	0.017690	0.033556	3.730804	3.000000	0.002600	0.032780	0.517205
Ruminococcus lactaris	Noradrenaline	0.010000	death_lin	0.004423	0.031773	0.027350	0.035647	3.643780	3.000000	0.003463	0.051237	0.799621
Ruminococcus lactaris	Noradrenaline	0.001000	death_lin	0.004423	0.016977	0.012554	0.050222	3.176673	3.000000	-0.000023	0.025131	0.367039
Ruminococcus lactaris	Noradrenaline	0.000100	death_lin	0.004423	0.022906	0.018483	0.030743	3.859516	3.000000	0.003242	0.033723	0.540375
Ruminococcus lactaris	Noradrenaline	0.000010	death_lin	0.004423	0.016074	0.011651	0.055481	3.048798	3.000000	-0.000511	0.023813	0.340641
Ruminococcus lactaris	Noradrenaline	0.000001	death_lin	0.004423	0.012957	0.008534	0.081740	2.580562	3.000000	-0.001990	0.019059	0.249509
Ruminococcus lactaris	Adrenaline	0.100000	death_lin	0.000728	0.027459	0.026732	0.021323	4.433679	3.000000	0.007544	0.045920	0.781551
Ruminococcus lactaris	Adrenaline	0.010000	death_lin	0.000728	0.023852	0.023124	0.033491	3.733645	3.000000	0.003414	0.042834	0.676074
Ruminococcus lactaris	Adrenaline	0.001000	death_lin	0.000728	0.019550	0.018822	0.030633	3.864870	3.000000	0.003323	0.034321	0.550307
Ruminococcus lactaris	Adrenaline	0.000100	death_lin	0.000728	0.018459	0.017731	0.002908	8.987454	3.000000	0.011452	0.024009	0.518394
Ruminococcus lactaris	Adrenaline	0.000010	death_lin	0.000728	0.016620	0.015893	0.033899	3.716064	3.000000	0.002282	0.029503	0.464652
Ruminococcus lactaris	Adrenaline	0.000001	death_lin	0.000728	0.015894	0.015167	0.074772	2.684343	3.000000	-0.002814	0.033148	0.443424
Ruminococcus lactaris	Levodopa	0.100000	gr	0.487104	0.422697	-0.064407	0.000818	-13.833547	3.000000	-0.079224	-0.049590	-0.766831
Ruminococcus lactaris	Levodopa	0.010000	gr	0.487104	0.460398	-0.026706	0.082844	-2.565113	3.000000	-0.059840	0.006427	-0.317966
Ruminococcus lactaris	Levodopa	0.001000	gr	0.487104	0.495160	0.008056	0.124194	2.119899	3.000000	-0.004038	0.020150	0.095913
Ruminococcus lactaris	Levodopa	0.000100	gr	0.487104	0.532496	0.045392	0.044596	3.333712	3.000000	0.002060	0.088724	0.540437
Ruminococcus lactaris	Levodopa	0.000010	gr	0.487104	0.513603	0.026499	0.046154	3.287816	3.000000	0.000849	0.052149	0.315497
Ruminococcus lactaris	Levodopa	0.000001	gr	0.487104	0.512208	0.025104	0.016849	4.837299	3.000000	0.008588	0.041620	0.298886
Ruminococcus lactaris	Dopamine	0.100000	gr	0.478172	0.454554	-0.023617	0.086022	-2.522044	3.000000	-0.053419	0.006184	-0.281190
Ruminococcus lactaris	Dopamine	0.010000	gr	0.478172	0.480941	0.002769	0.756928	0.339035	3.000000	-0.023225	0.028764	0.032971
Ruminococcus lactaris	Dopamine	0.001000	gr	0.478172	0.538714	0.060542	0.228444	1.508959	3.000000	-0.067143	0.188228	0.720816
Ruminococcus lactaris	Dopamine	0.000100	gr	0.478172	0.556170	0.077999	0.066135	2.831004	3.000000	-0.009683	0.165680	0.928655
Ruminococcus lactaris	Dopamine	0.000010	gr	0.478172	0.562163	0.083991	0.098267	2.372661	3.000000	-0.028666	0.196648	1.000000
Ruminococcus lactaris	Dopamine	0.000001	gr	0.478172	0.505966	0.027794	0.001004	12.907983	3.000000	0.020942	0.034647	0.330921
Ruminococcus lactaris	Noradrenaline	0.100000	gr	0.490760	0.480621	-0.010139	0.148611	-1.933802	3.000000	-0.026824	0.006547	-0.120713

Species	Hormon	Concentration	Parameter	Mean_Baseline	Mean_Treatment	Diff	P_Value	T_Statistic	DF	Lower_CI	Upper_CI	Diff_Norm
Ruminococcus lactaris	Noradrenaline	0.010000	gr	0.490760	0.486394	-0.004366	0.903266	-0.132101	3.000000	-0.109537	0.100806	-0.051977
Ruminococcus lactaris	Noradrenaline	0.001000	gr	0.490760	0.502181	0.011421	0.523809	0.719517	3.000000	-0.039095	0.061937	0.135979
Ruminococcus lactaris	Noradrenaline	0.000100	gr	0.490760	0.528818	0.038059	0.203326	1.621664	3.000000	-0.036630	0.112747	0.453128
Ruminococcus lactaris	Noradrenaline	0.000010	gr	0.490760	0.520842	0.030082	0.093614	2.426603	3.000000	-0.009370	0.069534	0.358155
Ruminococcus lactaris	Noradrenaline	0.000001	gr	0.490760	0.516970	0.026210	0.056039	3.036110	3.000000	-0.001263	0.053683	0.312054
Ruminococcus lactaris	Adrenaline	0.100000	gr	0.472375	0.468807	-0.003568	0.797128	-0.280782	3.000000	-0.044013	0.036876	-0.042485
Ruminococcus lactaris	Adrenaline	0.010000	gr	0.472375	0.517622	0.045246	0.402669	0.972174	3.000000	-0.102869	0.193362	0.538705
Ruminococcus lactaris	Adrenaline	0.001000	gr	0.472375	0.529800	0.057424	0.055248	3.054138	3.000000	-0.002412	0.117261	0.683697
Ruminococcus lactaris	Adrenaline	0.000100	gr	0.472375	0.507429	0.035054	0.058415	2.983899	3.000000	-0.002332	0.072441	0.417355
Ruminococcus lactaris	Adrenaline	0.000010	gr	0.472375	0.512372	0.039997	0.055049	3.058707	3.000000	-0.001618	0.081611	0.476199
Ruminococcus lactaris	Adrenaline	0.000001	gr	0.472375	0.516574	0.044198	0.027409	4.033167	3.000000	0.009323	0.079074	0.526225
Ruminococcus lactaris	Levodopa	0.100000	dr	-0.012525	-0.010088	0.002437	0.290936	1.278747	3.000000	-0.003629	0.008503	0.175638
Ruminococcus lactaris	Levodopa	0.010000	dr	-0.012525	-0.017856	-0.005330	0.268684	-1.354069	3.000000	-0.017858	0.007198	-0.384106
Ruminococcus lactaris	Levodopa	0.001000	dr	-0.012525	-0.017753	-0.005227	0.315107	-1.203449	3.000000	-0.019050	0.008596	-0.376674
Ruminococcus lactaris	Levodopa	0.000100	dr	-0.012525	-0.010778	0.001747	0.732443	0.375238	3.000000	-0.013070	0.016564	0.125894
Ruminococcus lactaris	Levodopa	0.000010	dr	-0.012525	-0.009165	0.003361	0.080177	2.602873	3.000000	-0.000748	0.007470	0.242183
Ruminococcus lactaris	Levodopa	0.000001	dr	-0.012525	-0.010918	0.001607	0.701928	0.421257	3.000000	-0.010533	0.013747	0.115799
Ruminococcus lactaris	Dopamine	0.100000	dr	-0.004932	-0.018236	-0.013305	0.244684	-1.443143	3.000000	-0.042645	0.016035	-0.958747
Ruminococcus lactaris	Dopamine	0.010000	dr	-0.004932	-0.018809	-0.013877	0.166992	-1.815968	3.000000	-0.038197	0.010442	-1.000000
Ruminococcus lactaris	Dopamine	0.001000	dr	-0.004932	-0.012935	-0.008004	0.418266	-0.936127	3.000000	-0.035213	0.019205	-0.576744
Ruminococcus lactaris	Dopamine	0.000100	dr	-0.004932	-0.011877	-0.006946	0.507752	-0.749928	3.000000	-0.036420	0.022529	-0.500500
Ruminococcus lactaris	Dopamine	0.000010	dr	-0.004932	-0.013938	-0.009006	0.467146	-0.830556	3.000000	-0.043514	0.025502	-0.648971
Ruminococcus lactaris	Dopamine	0.000001	dr	-0.004932	-0.009254	-0.004323	0.400101	-0.978232	3.000000	-0.018385	0.009740	-0.311490
Ruminococcus lactaris	Noradrenaline	0.100000	dr	-0.009067	-0.015032	-0.005965	0.371832	-1.047478	3.000000	-0.024087	0.012158	-0.429829
Ruminococcus lactaris	Noradrenaline	0.010000	dr	-0.009067	-0.014961	-0.005894	0.215501	-1.565210	3.000000	-0.017878	0.006090	-0.424722
Ruminococcus lactaris	Noradrenaline	0.001000	dr	-0.009067	-0.011334	-0.002266	0.659194	-0.487679	3.000000	-0.017057	0.012524	-0.163322
Ruminococcus lactaris	Noradrenaline	0.000100	dr	-0.009067	-0.013504	-0.004436	0.166227	-1.820565	3.000000	-0.012191	0.003319	-0.319678

Species	Hormon	Concentration	Parameter	Mean_Baseline	Mean_Treatment	Diff	P_Value	T_Statistic	DF	Lower_CI	Upper_CI	Diff_Norm
Ruminococcus lactaris	Noradrenaline	0.000010	dr	-0.009067	-0.009194	-0.000127	0.979811	-0.027468	3.000000	-0.014798	0.014545	-0.009125
Ruminococcus lactaris	Noradrenaline	0.000001	dr	-0.009067	-0.012447	-0.003380	0.621287	-0.548900	3.000000	-0.022977	0.016217	-0.243561
Ruminococcus lactaris	Adrenaline	0.100000	dr	-0.007510	-0.012729	-0.005219	0.216949	-1.558733	3.000000	-0.015874	0.005436	-0.376069
Ruminococcus lactaris	Adrenaline	0.010000	dr	-0.007510	-0.015911	-0.008402	0.057430	-3.005217	3.000000	-0.017299	0.000495	-0.605417
Ruminococcus lactaris	Adrenaline	0.001000	dr	-0.007510	-0.013164	-0.005655	0.270527	-1.347584	3.000000	-0.019009	0.007699	-0.407472
Ruminococcus lactaris	Adrenaline	0.000100	dr	-0.007510	-0.014116	-0.006606	0.150086	-1.923739	3.000000	-0.017534	0.004322	-0.476019
Ruminococcus lactaris	Adrenaline	0.000010	dr	-0.007510	-0.012428	-0.004918	0.136298	-2.022722	3.000000	-0.012656	0.002820	-0.354407
Ruminococcus lactaris	Adrenaline	0.000001	dr	-0.007510	-0.010257	-0.002747	0.486751	-0.790920	3.000000	-0.013801	0.008307	-0.197965
Ruminococcus lactaris	Levodopa	0.100000	td	1.424413	1.641732	0.217319	0.001120	12.439002	3.000000	0.161719	0.272919	1.000000
Ruminococcus lactaris	Levodopa	0.010000	td	1.424413	1.507065	0.082652	0.083934	2.550117	3.000000	-0.020494	0.185798	0.380326
Ruminococcus lactaris	Levodopa	0.001000	td	1.424413	1.402181	-0.022232	0.126202	-2.103022	3.000000	-0.055874	0.011411	-0.102299
Ruminococcus lactaris	Levodopa	0.000100	td	1.424413	1.307902	-0.116511	0.034894	-3.674359	3.000000	-0.217425	-0.015598	-0.536131
Ruminococcus lactaris	Levodopa	0.000010	td	1.424413	1.350520	-0.073893	0.045132	-3.317681	3.000000	-0.144774	-0.003012	-0.340021
Ruminococcus lactaris	Levodopa	0.000001	td	1.424413	1.354002	-0.070411	0.017168	-4.804089	3.000000	-0.117055	-0.023768	-0.324000
Ruminococcus lactaris	Dopamine	0.100000	td	1.450071	1.527105	0.077033	0.095835	2.400454	3.000000	-0.025095	0.179162	0.354472
Ruminococcus lactaris	Dopamine	0.010000	td	1.450071	1.442632	-0.007439	0.781442	-0.303352	3.000000	-0.085481	0.070603	-0.034231
Ruminococcus lactaris	Dopamine	0.001000	td	1.450071	1.310396	-0.139675	0.175711	-1.765210	3.000000	-0.391490	0.112141	-0.642718
Ruminococcus lactaris	Dopamine	0.000100	td	1.450071	1.258316	-0.191755	0.038448	-3.536943	3.000000	-0.364290	-0.019219	-0.882365
Ruminococcus lactaris	Dopamine	0.000010	td	1.450071	1.248154	-0.201917	0.070810	-2.748865	3.000000	-0.435682	0.031849	-0.929127
Ruminococcus lactaris	Dopamine	0.000001	td	1.450071	1.370481	-0.079590	0.000941	-13.190186	3.000000	-0.098793	-0.060387	-0.366237
Ruminococcus lactaris	Noradrenaline	0.100000	td	1.412961	1.442218	0.029257	0.151700	1.912847	3.000000	-0.019418	0.077932	0.134626
Ruminococcus lactaris	Noradrenaline	0.010000	td	1.412961	1.447816	0.034855	0.775127	0.312494	3.000000	-0.320107	0.389816	0.160385
Ruminococcus lactaris	Noradrenaline	0.001000	td	1.412961	1.382921	-0.030040	0.545860	-0.678944	3.000000	-0.170849	0.110769	-0.138231
Ruminococcus lactaris	Noradrenaline	0.000100	td	1.412961	1.319402	-0.093559	0.190589	-1.684932	3.000000	-0.270271	0.083152	-0.430515
Ruminococcus lactaris	Noradrenaline	0.000010	td	1.412961	1.331850	-0.081111	0.094932	-2.411000	3.000000	-0.188176	0.025953	-0.373237
Ruminococcus lactaris	Noradrenaline	0.000001	td	1.412961	1.341302	-0.071660	0.056318	-3.029844	3.000000	-0.146928	0.003609	-0.329744
Ruminococcus lactaris	Adrenaline	0.100000	td	1.470165	1.480717	0.010552	0.819771	0.248524	3.000000	-0.124572	0.145676	0.048556

Species	Hormon	Concentration	Parameter	Mean_Baseline	Mean_Treatment	Diff	P_Value	T_Statistic	DF	Lower_CI	Upper_CI	Diff_Norm
Ruminococcus lactaris	Adrenaline	0.010000	td	1.470165	1.356203	-0.113962	0.408226	-0.959185	3.000000	-0.492074	0.264149	-0.524402
Ruminococcus lactaris	Adrenaline	0.001000	td	1.470165	1.309130	-0.161035	0.060642	-2.937294	3.000000	-0.335510	0.013440	-0.741008
Ruminococcus lactaris	Adrenaline	0.000100	td	1.470165	1.374614	-0.095551	0.056963	-3.015485	3.000000	-0.196392	0.005290	-0.439681
Ruminococcus lactaris	Adrenaline	0.000010	td	1.470165	1.352955	-0.117210	0.066028	-2.832972	3.000000	-0.248880	0.014459	-0.539348
Ruminococcus lactaris	Adrenaline	0.000001	td	1.470165	1.341859	-0.128306	0.037133	-3.585811	3.000000	-0.242180	-0.014433	-0.590406
Ruminococcus lactaris	Levodopa	0.100000	lagC	2.318504	2.530509	0.212005	0.006376	6.847104	3.000000	0.113468	0.310543	0.884850
Ruminococcus lactaris	Levodopa	0.010000	lagC	2.318504	2.444355	0.125852	0.094361	2.417723	3.000000	-0.039807	0.291510	0.525269
Ruminococcus lactaris	Levodopa	0.001000	lagC	2.318504	2.514896	0.196392	0.017807	4.739961	3.000000	0.064533	0.328251	0.819684
Ruminococcus lactaris	Levodopa	0.000100	lagC	2.318504	2.490756	0.172252	0.018772	4.648545	3.000000	0.054326	0.290178	0.718932
Ruminococcus lactaris	Levodopa	0.000010	lagC	2.318504	2.496849	0.178345	0.013435	5.253320	3.000000	0.070304	0.286387	0.744363
Ruminococcus lactaris	Levodopa	0.000001	lagC	2.318504	2.459219	0.140715	0.019734	4.563325	3.000000	0.042581	0.238849	0.587305
Ruminococcus lactaris	Dopamine	0.100000	lagC	2.176237	2.312241	0.136004	0.009291	5.995693	3.000000	0.063815	0.208193	0.567642
Ruminococcus lactaris	Dopamine	0.010000	lagC	2.176237	2.223166	0.046929	0.359948	1.078109	3.000000	-0.091599	0.185457	0.195868
Ruminococcus lactaris	Dopamine	0.001000	lagC	2.176237	2.257041	0.080805	0.286480	1.293333	3.000000	-0.118027	0.279637	0.337255
Ruminococcus lactaris	Dopamine	0.000100	lagC	2.176237	2.280044	0.103807	0.009902	5.861424	3.000000	0.047445	0.160169	0.433262
Ruminococcus lactaris	Dopamine	0.000010	lagC	2.176237	2.236660	0.060423	0.371909	1.047283	3.000000	-0.123189	0.244035	0.252190
Ruminococcus lactaris	Dopamine	0.000001	lagC	2.176237	2.234141	0.057904	0.003278	8.624702	3.000000	0.036538	0.079271	0.241677
Ruminococcus lactaris	Noradrenaline	0.100000	lagC	2.292810	2.532405	0.239595	0.000648	14.960367	3.000000	0.188627	0.290562	1.000000
Ruminococcus lactaris	Noradrenaline	0.010000	lagC	2.292810	2.414991	0.122181	0.093520	2.427723	3.000000	-0.037983	0.282346	0.509950
Ruminococcus lactaris	Noradrenaline	0.001000	lagC	2.292810	2.407340	0.114529	0.084092	2.547961	3.000000	-0.028520	0.257578	0.478013
Ruminococcus lactaris	Noradrenaline	0.000100	lagC	2.292810	2.444062	0.151252	0.027335	4.037320	3.000000	0.032027	0.270477	0.631283
Ruminococcus lactaris	Noradrenaline	0.000010	lagC	2.292810	2.440957	0.148146	0.010061	5.828343	3.000000	0.067254	0.229039	0.618321
Ruminococcus lactaris	Noradrenaline	0.000001	lagC	2.292810	2.382254	0.089444	0.043189	3.376936	3.000000	0.005151	0.173736	0.373313
Ruminococcus lactaris	Adrenaline	0.100000	lagC	2.336914	2.527131	0.190217	0.005774	7.088886	3.000000	0.104822	0.275612	0.793913
Ruminococcus lactaris	Adrenaline	0.010000	lagC	2.336914	2.546396	0.209482	0.018391	4.683930	3.000000	0.067152	0.351812	0.874318
Ruminococcus lactaris	Adrenaline	0.001000	lagC	2.336914	2.515320	0.178406	0.006079	6.962396	3.000000	0.096858	0.259954	0.744617
Ruminococcus lactaris	Adrenaline	0.000100	lagC	2.336914	2.484962	0.148048	0.005105	7.399800	3.000000	0.084377	0.211719	0.617909

Species	Hormon	Concentration	Parameter	Mean_Baseline	Mean_Treatment	Diff	P_Value	T_Statistic	DF	Lower_CI	Upper_CI	Diff_Norm
Ruminococcus lactaris	Adrenaline	0.000010	lagC	2.336914	2.441443	0.104529	0.017485	4.771896	3.000000	0.034817	0.174240	0.436273
Ruminococcus lactaris	Adrenaline	0.000001	lagC	2.336914	2.410645	0.073731	0.040553	3.462971	3.000000	0.005973	0.141489	0.307733
Ruminococcus lactaris	Levodopa	0.100000	t_k	47.583333	35.916667	-11.666667	0.069156	-2.777136	3.000000	-25.036028	1.702694	-0.595745
Ruminococcus lactaris	Levodopa	0.010000	t_k	47.583333	33.083333	-14.500000	0.032123	-3.794583	3.000000	-26.660881	-2.339119	-0.740426
Ruminococcus lactaris	Levodopa	0.001000	t_k	47.583333	29.166667	-18.416667	0.001455	-11.382079	3.000000	-23.565994	-13.267339	-0.940426
Ruminococcus lactaris	Levodopa	0.000100	t_k	47.583333	30.500000	-17.083333	0.004591	-7.677310	3.000000	-24.164823	-10.001844	-0.872340
Ruminococcus lactaris	Levodopa	0.000010	t_k	47.583333	28.250000	-19.333333	0.000417	-17.356662	3.000000	-22.878214	-15.788453	-0.987234
Ruminococcus lactaris	Levodopa	0.000001	t_k	47.583333	38.916667	-8.666667	0.189251	-1.691859	3.000000	-24.968976	7.635643	-0.442553
Ruminococcus lactaris	Dopamine	0.100000	t_k	47.833333	32.833333	-15.000000	0.002591	-9.349335	3.000000	-20.105892	-9.894108	-0.765957
Ruminococcus lactaris	Dopamine	0.010000	t_k	47.833333	28.250000	-19.583333	0.000657	-14.892520	3.000000	-23.768180	-15.398487	-1.000000
Ruminococcus lactaris	Dopamine	0.001000	t_k	47.833333	32.416667	-15.416667	0.024310	-4.220929	3.000000	-27.040343	-3.792990	-0.787234
Ruminococcus lactaris	Dopamine	0.000100	t_k	47.833333	30.000000	-17.833333	0.003250	-8.650437	3.000000	-24.394114	-11.272552	-0.910638
Ruminococcus lactaris	Dopamine	0.000010	t_k	47.833333	37.250000	-10.583333	0.142375	-1.977707	3.000000	-27.613607	6.446940	-0.540426
Ruminococcus lactaris	Dopamine	0.000001	t_k	47.833333	39.000000	-8.833333	0.188860	-1.693887	3.000000	-25.429248	7.762581	-0.451064
Ruminococcus lactaris	Noradrenaline	0.100000	t_k	39.583333	34.666667	-4.916667	0.544233	-0.681895	3.000000	-27.863059	18.029726	-0.251064
Ruminococcus lactaris	Noradrenaline	0.010000	t_k	39.583333	31.333333	-8.250000	0.177012	-1.757889	3.000000	-23.185632	6.685632	-0.421277
Ruminococcus lactaris	Noradrenaline	0.001000	t_k	39.583333	33.250000	-6.333333	0.177474	-1.755301	3.000000	-17.815976	5.149310	-0.323404
Ruminococcus lactaris	Noradrenaline	0.000100	t_k	39.583333	29.083333	-10.500000	0.182731	-1.726405	3.000000	-29.855643	8.855643	-0.536170
Ruminococcus lactaris	Noradrenaline	0.000010	t_k	39.583333	33.750000	-5.833333	0.328627	-1.163868	3.000000	-21.783826	10.117159	-0.297872
Ruminococcus lactaris	Noradrenaline	0.000001	t_k	39.583333	30.000000	-9.583333	0.178132	-1.751630	3.000000	-26.994796	7.828130	-0.489362
Ruminococcus lactaris	Adrenaline	0.100000	t_k	47.750000	32.250000	-15.500000	0.036224	-3.620935	3.000000	-29.122980	-1.877020	-0.791489
Ruminococcus lactaris	Adrenaline	0.010000	t_k	47.750000	33.416667	-14.333333	0.005671	-7.133716	3.000000	-20.727625	-7.939042	-0.731915
Ruminococcus lactaris	Adrenaline	0.001000	t_k	47.750000	30.500000	-17.250000	0.000017	-50.204875	3.000000	-18.343464	-16.156536	-0.880851
Ruminococcus lactaris	Adrenaline	0.000100	t_k	47.750000	30.083333	-17.666667	0.000322	-18.936624	3.000000	-20.635687	-14.697647	-0.902128
Ruminococcus lactaris	Adrenaline	0.000010	t_k	47.750000	29.250000	-18.500000	0.000258	-20.379269	3.000000	-21.388978	-15.611022	-0.944681
Ruminococcus lactaris	Adrenaline	0.000001	t_k	47.750000	33.916667	-13.833333	0.059129	-2.968707	3.000000	-28.662633	0.995966	-0.706383
Ruminococcus lactaris	Levodopa	0.100000	t_gr	3.583333	3.583333	0.000000	NA	NA	3.000000	NA	NA	0.000000

Species	Hormon	Concentration	Parameter	Mean_Baseline	Mean_Treatment	Diff	P_Value	T_Statistic	DF	Lower_CI	Upper_CI	Diff_Norm
Ruminococcus lactaris	Levodopa	0.010000	t_gr	3.583333	3.583333	0.000000	NA	NA	3.000000	NA	NA	0.000000
Ruminococcus lactaris	Levodopa	0.001000	t_gr	3.583333	3.666667	0.083333	0.391002	1.000000	3.000000	-0.181871	0.348537	0.250000
Ruminococcus lactaris	Levodopa	0.000100	t_gr	3.583333	3.583333	0.000000	NA	NA	3.000000	NA	NA	0.000000
Ruminococcus lactaris	Levodopa	0.000010	t_gr	3.583333	3.583333	0.000000	NA	NA	3.000000	NA	NA	0.000000
Ruminococcus lactaris	Levodopa	0.000001	t_gr	3.583333	3.583333	0.000000	NA	NA	3.000000	NA	NA	0.000000
Ruminococcus lactaris	Dopamine	0.100000	t_gr	3.333333	3.416667	0.083333	0.391002	1.000000	3.000000	-0.181871	0.348537	0.250000
Ruminococcus lactaris	Dopamine	0.010000	t_gr	3.333333	3.416667	0.083333	0.391002	1.000000	3.000000	-0.181871	0.348537	0.250000
Ruminococcus lactaris	Dopamine	0.001000	t_gr	3.333333	3.416667	0.083333	0.391002	1.000000	3.000000	-0.181871	0.348537	0.250000
Ruminococcus lactaris	Dopamine	0.000100	t_gr	3.333333	3.333333	0.000000	NA	NA	3.000000	NA	NA	0.000000
Ruminococcus lactaris	Dopamine	0.000010	t_gr	3.333333	3.416667	0.083333	0.391002	1.000000	3.000000	-0.181871	0.348537	0.250000
Ruminococcus lactaris	Dopamine	0.000001	t_gr	3.333333	3.333333	0.000000	NA	NA	3.000000	NA	NA	0.000000
Ruminococcus lactaris	Noradrenaline	0.100000	t_gr	3.416667	3.750000	0.333333	0.000000	1333333815.000000	3.000000	0.333333	0.333333	1.000000
Ruminococcus lactaris	Noradrenaline	0.010000	t_gr	3.416667	3.583333	0.166667	0.181690	1.732051	3.000000	-0.139564	0.472898	0.500000
Ruminococcus lactaris	Noradrenaline	0.001000	t_gr	3.416667	3.583333	0.166667	0.181690	1.732051	3.000000	-0.139564	0.472898	0.500000
Ruminococcus lactaris	Noradrenaline	0.000100	t_gr	3.416667	3.583333	0.166667	0.181690	1.732051	3.000000	-0.139564	0.472898	0.500000
Ruminococcus lactaris	Noradrenaline	0.000010	t_gr	3.416667	3.583333	0.166667	0.181690	1.732051	3.000000	-0.139564	0.472898	0.500000
Ruminococcus lactaris	Noradrenaline	0.000001	t_gr	3.416667	3.583333	0.166667	0.181690	1.732051	3.000000	-0.139564	0.472898	0.500000
Ruminococcus lactaris	Adrenaline	0.100000	t_gr	3.500000	3.583333	0.083333	0.391002	1.000000	3.000000	-0.181871	0.348537	0.250000
Ruminococcus lactaris	Adrenaline	0.010000	t_gr	3.500000	3.583333	0.083333	0.391002	1.000000	3.000000	-0.181871	0.348537	0.250000
Ruminococcus lactaris	Adrenaline	0.001000	t_gr	3.500000	3.666667	0.166667	0.181690	1.732051	3.000000	-0.139564	0.472898	0.500000
Ruminococcus lactaris	Adrenaline	0.000100	t_gr	3.500000	3.583333	0.083333	0.391002	1.000000	3.000000	-0.181871	0.348537	0.250000
Ruminococcus lactaris	Adrenaline	0.000010	t_gr	3.500000	3.583333	0.083333	0.391002	1.000000	3.000000	-0.181871	0.348537	0.250000
Ruminococcus lactaris	Adrenaline	0.000001	t_gr	3.500000	3.583333	0.083333	0.391002	1.000000	3.000000	-0.181871	0.348537	0.250000
Ruminococcus lactaris	Levodopa	0.100000	t_dr	48.000000	48.000000	0.000000	NA	NA	3.000000	NA	NA	0.000000
Ruminococcus lactaris	Levodopa	0.010000	t_dr	48.000000	48.000000	0.000000	NA	NA	3.000000	NA	NA	0.000000
Ruminococcus lactaris	Levodopa	0.001000	t_dr	48.000000	48.000000	0.000000	NA	NA	3.000000	NA	NA	0.000000
Ruminococcus lactaris	Levodopa	0.000100	t_dr	48.000000	44.083333	-3.916667	0.212520	-1.578699	3.000000	-11.812144	3.978810	-0.528090

Species	Hormon	Concentration	Parameter	Mean_Baseline	Mean_Treatment	Diff	P_Value	T_Statistic	DF	Lower_CI	Upper_CI	Diff_Norm
Ruminococcus lactaris	Levodopa	0.000010	t_dr	48.000000	44.916667	-3.083333	0.391002	-1.000000	3.000000	-12.895876	6.729209	-0.415730
Ruminococcus lactaris	Levodopa	0.000001	t_dr	48.000000	48.000000	0.000000	NA	NA	3.000000	NA	NA	0.000000
Ruminococcus lactaris	Dopamine	0.100000	t_dr	48.000000	48.000000	0.000000	NA	NA	3.000000	NA	NA	0.000000
Ruminococcus lactaris	Dopamine	0.010000	t_dr	48.000000	48.000000	0.000000	NA	NA	3.000000	NA	NA	0.000000
Ruminococcus lactaris	Dopamine	0.001000	t_dr	48.000000	48.000000	0.000000	NA	NA	3.000000	NA	NA	0.000000
Ruminococcus lactaris	Dopamine	0.000100	t_dr	48.000000	48.000000	0.000000	NA	NA	3.000000	NA	NA	0.000000
Ruminococcus lactaris	Dopamine	0.000010	t_dr	48.000000	43.083333	-4.916667	0.391002	-1.000000	3.000000	-20.563694	10.730361	-0.662921
Ruminococcus lactaris	Dopamine	0.000001	t_dr	48.000000	48.000000	0.000000	NA	NA	3.000000	NA	NA	0.000000
Ruminococcus lactaris	Noradrenaline	0.100000	t_dr	48.000000	48.000000	0.000000	NA	NA	3.000000	NA	NA	0.000000
Ruminococcus lactaris	Noradrenaline	0.010000	t_dr	48.000000	40.583333	-7.416667	0.183653	-1.721435	3.000000	-21.127988	6.294655	-1.000000
Ruminococcus lactaris	Noradrenaline	0.001000	t_dr	48.000000	47.583333	-0.416667	0.391002	-1.000000	3.000000	-1.742686	0.909353	-0.056180
Ruminococcus lactaris	Noradrenaline	0.000100	t_dr	48.000000	48.000000	0.000000	NA	NA	3.000000	NA	NA	0.000000
Ruminococcus lactaris	Noradrenaline	0.000010	t_dr	48.000000	44.083333	-3.916667	0.391002	-1.000000	3.000000	-16.381248	8.547915	-0.528090
Ruminococcus lactaris	Noradrenaline	0.000001	t_dr	48.000000	46.166667	-1.833333	0.391002	-1.000000	3.000000	-7.667818	4.001152	-0.247191
Ruminococcus lactaris	Adrenaline	0.100000	t_dr	48.000000	45.000000	-3.000000	0.391002	-1.000000	3.000000	-12.547339	6.547339	-0.404494
Ruminococcus lactaris	Adrenaline	0.010000	t_dr	48.000000	46.000000	-2.000000	0.391002	-1.000000	3.000000	-8.364893	4.364893	-0.269663
Ruminococcus lactaris	Adrenaline	0.001000	t_dr	48.000000	48.000000	0.000000	NA	NA	3.000000	NA	NA	0.000000
Ruminococcus lactaris	Adrenaline	0.000100	t_dr	48.000000	48.000000	0.000000	NA	NA	3.000000	NA	NA	0.000000
Ruminococcus lactaris	Adrenaline	0.000010	t_dr	48.000000	48.000000	0.000000	NA	NA	3.000000	NA	NA	0.000000
Ruminococcus lactaris	Adrenaline	0.000001	t_dr	48.000000	43.833333	-4.166667	0.391002	-1.000000	3.000000	-17.426860	9.093526	-0.561798

Supplementary Table 3

Species	Condition	Parameter	contrast	estimate	SE	df	t.ratio	p.value
Bacteroides caccae	Adrenaline	auc_lin	Adrenaline - NK	-1.797074	0.764796	103.102698	-2.349743	0.137859
Bacteroides caccae	Dopamine	auc_lin	Dopamine - NK	-2.337663	0.770565	103.165669	-3.033699	0.024907
Bacteroides caccae	Levodopa	auc_lin	Levodopa - NK	-2.486228	0.764796	103.102698	-3.250839	0.013205
Bacteroides caccae	Noradrenaline	auc_lin	NK - Noradrenaline	-1.810664	0.770565	103.165669	2.349787	0.137841
Bacteroides caccae	Adrenaline	k_lin	Adrenaline - NK	-0.058769	0.012283	103.253069	-4.784523	0.000056
Bacteroides caccae	Dopamine	k_lin	Dopamine - NK	-0.057041	0.012376	103.410643	-4.609029	0.000112
Bacteroides caccae	Levodopa	k_lin	Levodopa - NK	-0.072557	0.012283	103.253069	-5.907011	0.000000
Bacteroides caccae	Noradrenaline	k_lin	NK - Noradrenaline	-0.047468	0.012376	103.410643	3.835508	0.001977
Bacteroides caccae	Adrenaline	death_lin	Adrenaline - NK	0.005378	0.002684	104.117827	2.003650	0.271540
Bacteroides caccae	Dopamine	death_lin	Dopamine - NK	0.002263	0.002704	104.880732	0.836988	0.918477
Bacteroides caccae	Levodopa	death_lin	Levodopa - NK	0.001539	0.002684	104.117827	0.573240	0.978688
Bacteroides caccae	Noradrenaline	death_lin	NK - Noradrenaline	0.005782	0.002704	104.880732	-2.138001	0.212017
Bacteroides caccae	Adrenaline	gr	Adrenaline - NK	-0.018544	0.032266	103.053500	-0.574733	0.978480
Bacteroides caccae	Dopamine	gr	Dopamine - NK	-0.053722	0.032509	103.086141	-1.652511	0.467800
Bacteroides caccae	Levodopa	gr	Levodopa - NK	-0.020027	0.032266	103.053500	-0.620686	0.971473
Bacteroides caccae	Noradrenaline	gr	NK - Noradrenaline	-0.010951	0.032509	103.086141	0.336862	0.997184
Bacteroides caccae	Adrenaline	dr	Adrenaline - NK	0.000061	0.003045	103.730755	0.019884	1.000000
Bacteroides caccae	Dopamine	dr	Dopamine - NK	0.005427	0.003068	104.209142	1.768596	0.397374
Bacteroides caccae	Levodopa	dr	Levodopa - NK	0.005390	0.003045	103.730755	1.769958	0.396599
Bacteroides caccae	Noradrenaline	dr	NK - Noradrenaline	-0.001732	0.003068	104.209142	0.564318	0.979889
Bacteroides caccae	Adrenaline	td	Adrenaline - NK	0.864698	0.438766	103.085002	1.970751	0.287624
Bacteroides caccae	Dopamine	td	Dopamine - NK	1.534211	0.442075	103.137028	3.470476	0.006676
Bacteroides caccae	Levodopa	td	Levodopa - NK	1.193686	0.438766	103.085002	2.720555	0.057730
Bacteroides caccae	Noradrenaline	td	NK - Noradrenaline	0.754737	0.442075	103.137028	-1.707258	0.434085
Bacteroides caccae	Adrenaline	lagC	Adrenaline - NK	3.547794	1.386400	103.066834	2.558996	0.085837

Species	Condition	Parameter	contrast	estimate	SE	df	t.ratio	p.value
Bacteroides caccae	Dopamine	lagC	Dopamine - NK	4.016651	1.396856	103.107665	2.875493	0.038527
Bacteroides caccae	Levodopa	lagC	Levodopa - NK	5.763212	1.386400	103.066834	4.156961	0.000628
Bacteroides caccae	Noradrenaline	lagC	NK - Noradrenaline	3.907936	1.396856	103.107665	-2.797665	0.047340
Bacteroides caccae	Adrenaline	t_k	Adrenaline - NK	-3.522042	2.582618	103.836799	-1.363749	0.652060
Bacteroides caccae	Dopamine	t_k	Dopamine - NK	-2.312570	2.602206	104.390866	-0.888696	0.900495
Bacteroides caccae	Levodopa	t_k	Levodopa - NK	-1.424820	2.582618	103.836799	-0.551696	0.981505
Bacteroides caccae	Noradrenaline	t_k	NK - Noradrenaline	-2.712790	2.602206	104.390866	1.042496	0.834962
Bacteroides caccae	Adrenaline	t_gr	Adrenaline - NK	3.525469	1.666612	103.052607	2.115351	0.221505
Bacteroides caccae	Dopamine	t_gr	Dopamine - NK	4.704010	1.679180	103.084701	2.801374	0.046885
Bacteroides caccae	Levodopa	t_gr	Levodopa - NK	6.039357	1.666612	103.052607	3.623734	0.004057
Bacteroides caccae	Noradrenaline	t_gr	NK - Noradrenaline	4.008942	1.679180	103.084701	-2.387441	0.127016
Bacteroides caccae	Adrenaline	t_dr	Adrenaline - NK	0.395833	1.875307	105.049685	0.211077	0.999551
Bacteroides caccae	Dopamine	t_dr	Dopamine - NK	-1.423611	1.887079	106.563229	-0.754399	0.942865
Bacteroides caccae	Levodopa	t_dr	Levodopa - NK	-1.215278	1.875307	105.049685	-0.648042	0.966655
Bacteroides caccae	Noradrenaline	t_dr	NK - Noradrenaline	-2.368056	1.887079	106.563229	1.254879	0.719164
Bacteroides finegoldii	Adrenaline	auc_lin	Adrenaline - NK	4.757625	0.545015	102.631552	8.729355	0.000000
Bacteroides finegoldii	Dopamine	auc_lin	Dopamine - NK	5.936571	0.555183	103.484683	10.693001	0.000000
Bacteroides finegoldii	Levodopa	auc_lin	Levodopa - NK	4.542629	0.539114	102.362920	8.426102	0.000000
Bacteroides finegoldii	Noradrenaline	auc_lin	NK - Noradrenaline	4.142026	0.539114	102.362920	-7.683024	0.000000
Bacteroides finegoldii	Adrenaline	k_lin	Adrenaline - NK	0.066257	0.012719	102.676630	5.209354	0.000010
Bacteroides finegoldii	Dopamine	k_lin	Dopamine - NK	0.104827	0.012955	103.579831	8.091451	0.000000
Bacteroides finegoldii	Levodopa	k_lin	Levodopa - NK	0.066186	0.012581	102.389881	5.260609	0.000008
Bacteroides finegoldii	Noradrenaline	k_lin	NK - Noradrenaline	0.046388	0.012581	102.389881	-3.687005	0.003294
Bacteroides finegoldii	Adrenaline	death_lin	Adrenaline - NK	-0.012761	0.004471	104.828252	-2.854437	0.040662
Bacteroides finegoldii	Dopamine	death_lin	Dopamine - NK	-0.017800	0.004522	106.444935	-3.936643	0.001367
Bacteroides finegoldii	Levodopa	death_lin	Levodopa - NK	-0.016299	0.004429	103.864503	-3.680368	0.003349
Bacteroides finegoldii	Noradrenaline	death_lin	NK - Noradrenaline	-0.014663	0.004429	103.864503	3.310989	0.010977

Species	Condition	Parameter	contrast	estimate	SE	df	t.ratio	p.value
Bacteroides finegoldii	Adrenaline	gr	Adrenaline - NK	0.039396	0.014457	106.904482	2.725137	0.056822
Bacteroides finegoldii	Dopamine	gr	Dopamine - NK	0.041782	0.014386	106.904482	2.904482	0.035433
Bacteroides finegoldii	Levodopa	gr	Levodopa - NK	0.005475	0.014373	105.816824	0.380949	0.995466
Bacteroides finegoldii	Noradrenaline	gr	NK - Noradrenaline	0.012420	0.014373	105.816824	-0.864149	0.909300
Bacteroides finegoldii	Adrenaline	dr	Adrenaline - NK	-0.001331	0.006593	106.291257	-0.201877	0.999624
Bacteroides finegoldii	Dopamine	dr	Dopamine - NK	0.004098	0.006602	106.918077	0.620689	0.971485
Bacteroides finegoldii	Levodopa	dr	Levodopa - NK	-0.000980	0.006542	105.119257	-0.149735	0.999885
Bacteroides finegoldii	Noradrenaline	dr	NK - Noradrenaline	-0.001706	0.006542	105.119257	0.260757	0.998967
Bacteroides finegoldii	Adrenaline	td	Adrenaline - NK	-0.121488	0.058287	106.904482	-2.084308	0.234482
Bacteroides finegoldii	Dopamine	td	Dopamine - NK	-0.117277	0.058000	106.904482	-2.022013	0.262654
Bacteroides finegoldii	Levodopa	td	Levodopa - NK	-0.023883	0.057949	105.816824	-0.412139	0.993862
Bacteroides finegoldii	Noradrenaline	td	NK - Noradrenaline	-0.014571	0.057949	105.816824	0.251441	0.999105
Bacteroides finegoldii	Adrenaline	lagC	Adrenaline - NK	0.137753	0.038743	102.170811	3.555597	0.005089
Bacteroides finegoldii	Dopamine	lagC	Dopamine - NK	-0.031102	0.039486	102.429010	-0.787676	0.933656
Bacteroides finegoldii	Levodopa	lagC	Levodopa - NK	0.180025	0.038318	102.095506	4.698153	0.000079
Bacteroides finegoldii	Noradrenaline	lagC	NK - Noradrenaline	0.188593	0.038318	102.095506	-4.921753	0.000032
Bacteroides finegoldii	Adrenaline	t_k	Adrenaline - NK	5.564819	2.476580	104.425747	2.246977	0.170900
Bacteroides finegoldii	Dopamine	t_k	Dopamine - NK	8.314151	2.509674	106.143418	3.312840	0.010853
Bacteroides finegoldii	Levodopa	t_k	Levodopa - NK	7.161341	2.452332	103.558698	2.920217	0.034114
Bacteroides finegoldii	Noradrenaline	t_k	NK - Noradrenaline	3.681910	2.452332	103.558698	-1.501392	0.563942
Bacteroides finegoldii	Adrenaline	t_gr	Adrenaline - NK	0.123530	0.043389	102.209735	2.847062	0.041618
Bacteroides finegoldii	Dopamine	t_gr	Dopamine - NK	0.014345	0.044220	102.523969	0.324390	0.997569
Bacteroides finegoldii	Levodopa	t_gr	Levodopa - NK	0.210300	0.042914	102.117538	4.900529	0.000035
Bacteroides finegoldii	Noradrenaline	t_gr	NK - Noradrenaline	0.110159	0.042914	102.117538	-2.566998	0.084290
Bacteroides finegoldii	Adrenaline	t_dr	Adrenaline - NK	6.088166	2.336660	104.946865	2.605499	0.076648
Bacteroides finegoldii	Dopamine	t_dr	Dopamine - NK	8.123826	2.361759	106.516576	3.439735	0.007290
Bacteroides finegoldii	Levodopa	t_dr	Levodopa - NK	8.266438	2.314853	103.957455	3.571042	0.004810

Species	Condition	Parameter	contrast	estimate	SE	df	t.ratio	p.value
Bacteroides finegoldii	Noradrenaline	t_dr	NK - Noradrenaline	4.604903	2.314853	103.957455	-1.989285	0.278475
Bacteroides ovatus	Adrenaline	auc_lin	Adrenaline - NK	-1.839062	0.481491	103.335594	-3.819514	0.002090
Bacteroides ovatus	Dopamine	auc_lin	Dopamine - NK	-2.135284	0.481491	103.335594	-4.434731	0.000220
Bacteroides ovatus	Levodopa	auc_lin	Levodopa - NK	-2.625821	0.485131	103.546338	-5.412607	0.000004
Bacteroides ovatus	Noradrenaline	auc_lin	NK - Noradrenaline	-1.709347	0.485131	103.546338	3.523479	0.005623
Bacteroides ovatus	Adrenaline	k_lin	Adrenaline - NK	-0.036782	0.012487	103.443055	-2.945729	0.031818
Bacteroides ovatus	Dopamine	k_lin	Dopamine - NK	-0.032717	0.012487	103.443055	-2.620152	0.074071
Bacteroides ovatus	Levodopa	k_lin	Levodopa - NK	-0.052299	0.012581	103.724410	-4.157002	0.000625
Bacteroides ovatus	Noradrenaline	k_lin	NK - Noradrenaline	-0.028309	0.012581	103.724410	2.250171	0.169844
Bacteroides ovatus	Adrenaline	death_lin	Adrenaline - NK	0.019856	0.005522	103.472339	3.595737	0.004443
Bacteroides ovatus	Dopamine	death_lin	Dopamine - NK	0.020046	0.005522	103.472339	3.630178	0.003965
Bacteroides ovatus	Levodopa	death_lin	Levodopa - NK	0.018258	0.005564	103.773212	3.281485	0.012015
Bacteroides ovatus	Noradrenaline	death_lin	NK - Noradrenaline	0.022803	0.005564	103.773212	-4.098402	0.000775
Bacteroides ovatus	Adrenaline	gr	Adrenaline - NK	-0.004731	0.022149	103.595344	-0.213589	0.999530
Bacteroides ovatus	Dopamine	gr	Dopamine - NK	0.003052	0.022149	103.595344	0.137802	0.999917
Bacteroides ovatus	Levodopa	gr	Levodopa - NK	-0.019246	0.022316	103.979514	-0.862415	0.909892
Bacteroides ovatus	Noradrenaline	gr	NK - Noradrenaline	0.013737	0.022316	103.979514	-0.615562	0.972324
Bacteroides ovatus	Adrenaline	dr	Adrenaline - NK	-0.002280	0.002278	103.161634	-1.000856	0.854413
Bacteroides ovatus	Dopamine	dr	Dopamine - NK	-0.011620	0.002278	103.161634	-5.100357	0.000015
Bacteroides ovatus	Levodopa	dr	Levodopa - NK	-0.004810	0.002296	103.261338	-2.095362	0.229968
Bacteroides ovatus	Noradrenaline	dr	NK - Noradrenaline	-0.002644	0.002296	103.261338	1.151730	0.778505
Bacteroides ovatus	Adrenaline	td	Adrenaline - NK	-0.058248	0.308677	103.523674	-0.188701	0.999712
Bacteroides ovatus	Dopamine	td	Dopamine - NK	-0.353800	0.308677	103.523674	-1.146182	0.781546
Bacteroides ovatus	Levodopa	td	Levodopa - NK	-0.105300	0.311014	103.859049	-0.338571	0.997129
Bacteroides ovatus	Noradrenaline	td	NK - Noradrenaline	-0.305700	0.311014	103.859049	0.982913	0.862421
Bacteroides ovatus	Adrenaline	lagC	Adrenaline - NK	3.068824	0.754124	103.105189	4.069389	0.000864
Bacteroides ovatus	Dopamine	lagC	Dopamine - NK	5.055748	0.754124	103.105189	6.704133	0.000000

Species	Condition	Parameter	contrast	estimate	SE	df	t.ratio	p.value
Bacteroides ovatus	Levodopa	lagC	Levodopa - NK	4.669021	0.759813	103.169704	6.144959	0.000000
Bacteroides ovatus	Noradrenaline	lagC	NK - Noradrenaline	3.483945	0.759813	103.169704	-4.585264	0.000123
Bacteroides ovatus	Adrenaline	t_k	Adrenaline - NK	-5.190004	1.400395	104.184872	-3.706102	0.003067
Bacteroides ovatus	Dopamine	t_k	Dopamine - NK	-3.342782	1.400395	104.184872	-2.387029	0.127044
Bacteroides ovatus	Levodopa	t_k	Levodopa - NK	-4.592790	1.410997	104.999392	-3.254996	0.012982
Bacteroides ovatus	Noradrenaline	t_k	NK - Noradrenaline	-4.721645	1.410997	104.999392	3.346318	0.009815
Bacteroides ovatus	Adrenaline	t_gr	Adrenaline - NK	3.835992	0.817544	103.114949	4.692093	0.000081
Bacteroides ovatus	Dopamine	t_gr	Dopamine - NK	5.335992	0.817544	103.114949	6.526857	0.000000
Bacteroides ovatus	Levodopa	t_gr	Levodopa - NK	5.015211	0.823712	103.185519	6.088550	0.000000
Bacteroides ovatus	Noradrenaline	t_gr	NK - Noradrenaline	3.535028	0.823712	103.185519	-4.291583	0.000380
Bacteroides ovatus	Adrenaline	t_dr	Adrenaline - NK	-7.320206	2.631881	103.877929	-2.781360	0.049345
Bacteroides ovatus	Dopamine	t_dr	Dopamine - NK	-5.667429	2.631881	103.877929	-2.153376	0.205904
Bacteroides ovatus	Levodopa	t_dr	Levodopa - NK	-5.344728	2.651845	104.461805	-2.015476	0.265898
Bacteroides ovatus	Noradrenaline	t_dr	NK - Noradrenaline	-4.889859	2.651845	104.461805	1.843946	0.354273
Bacteroides thetaiotaomicron	Adrenaline	auc_lin	Adrenaline - NK	-2.313798	0.878865	104.000000	-2.632712	0.071794
Bacteroides thetaiotaomicron	Dopamine	auc_lin	Dopamine - NK	-2.734197	0.878865	104.000000	-3.111055	0.019927
Bacteroides thetaiotaomicron	Levodopa	auc_lin	Levodopa - NK	-4.224670	0.878865	104.000000	-4.806961	0.000050
Bacteroides thetaiotaomicron	Noradrenaline	auc_lin	NK - Noradrenaline	-3.763573	0.878865	104.000000	4.282311	0.000392
Bacteroides thetaiotaomicron	Adrenaline	k_lin	Adrenaline - NK	-0.062430	0.019071	104.000000	-3.273558	0.012301
Bacteroides thetaiotaomicron	Dopamine	k_lin	Dopamine - NK	-0.066860	0.019071	104.000000	-3.505868	0.005943
Bacteroides thetaiotaomicron	Levodopa	k_lin	Levodopa - NK	-0.103239	0.019071	104.000000	-5.413435	0.000004
Bacteroides thetaiotaomicron	Noradrenaline	k_lin	NK - Noradrenaline	-0.089845	0.019071	104.000000	4.711083	0.000074
Bacteroides thetaiotaomicron	Adrenaline	death_lin	Adrenaline - NK	0.011225	0.005450	104.000000	2.059403	0.245705
Bacteroides thetaiotaomicron	Dopamine	death_lin	Dopamine - NK	0.008344	0.005450	104.000000	1.530964	0.544906
Bacteroides thetaiotaomicron	Levodopa	death_lin	Levodopa - NK	0.016443	0.005450	104.000000	3.016845	0.026082
Bacteroides thetaiotaomicron	Noradrenaline	death_lin	NK - Noradrenaline	0.004167	0.005450	104.000000	-0.764461	0.940164
Bacteroides thetaiotaomicron	Adrenaline	gr	Adrenaline - NK	-0.013044	0.033031	104.000000	-0.394918	0.994789

Species	Condition	Parameter	contrast	estimate	SE	df	t.ratio	p.value
Bacteroides thetaiotaomicron	Dopamine	gr	Dopamine - NK	-0.023903	0.033031	104.000000	-0.723661	0.950585
Bacteroides thetaiotaomicron	Levodopa	gr	Levodopa - NK	-0.074660	0.033031	104.000000	-2.260321	0.166325
Bacteroides thetaiotaomicron	Noradrenaline	gr	NK - Noradrenaline	-0.061386	0.033031	104.000000	1.858461	0.346277
Bacteroides thetaiotaomicron	Adrenaline	dr	Adrenaline - NK	-0.009734	0.006085	104.000000	-1.599521	0.501097
Bacteroides thetaiotaomicron	Dopamine	dr	Dopamine - NK	0.000593	0.006085	104.000000	0.097387	0.999979
Bacteroides thetaiotaomicron	Levodopa	dr	Levodopa - NK	-0.003579	0.006085	104.000000	-0.588057	0.976588
Bacteroides thetaiotaomicron	Noradrenaline	dr	NK - Noradrenaline	-0.004226	0.006085	104.000000	0.694419	0.957288
Bacteroides thetaiotaomicron	Adrenaline	td	Adrenaline - NK	1.744180	0.500677	104.000000	3.483642	0.006383
Bacteroides thetaiotaomicron	Dopamine	td	Dopamine - NK	2.415064	0.500677	104.000000	4.823595	0.000047
Bacteroides thetaiotaomicron	Levodopa	td	Levodopa - NK	3.340855	0.500677	104.000000	6.672672	0.000000
Bacteroides thetaiotaomicron	Noradrenaline	td	NK - Noradrenaline	2.181137	0.500677	104.000000	-4.356373	0.000296
Bacteroides thetaiotaomicron	Adrenaline	lagC	Adrenaline - NK	2.839404	0.889860	104.000000	3.190845	0.015769
Bacteroides thetaiotaomicron	Dopamine	lagC	Dopamine - NK	4.228799	0.889860	104.000000	4.752209	0.000063
Bacteroides thetaiotaomicron	Levodopa	lagC	Levodopa - NK	4.346504	0.889860	104.000000	4.884483	0.000037
Bacteroides thetaiotaomicron	Noradrenaline	lagC	NK - Noradrenaline	4.467488	0.889860	104.000000	-5.020442	0.000021
Bacteroides thetaiotaomicron	Adrenaline	t_k	Adrenaline - NK	-6.826389	2.419826	104.000000	-2.821025	0.044476
Bacteroides thetaiotaomicron	Dopamine	t_k	Dopamine - NK	-5.659722	2.419826	104.000000	-2.338896	0.141035
Bacteroides thetaiotaomicron	Levodopa	t_k	Levodopa - NK	-5.604167	2.419826	104.000000	-2.315938	0.148102
Bacteroides thetaiotaomicron	Noradrenaline	t_k	NK - Noradrenaline	-6.798611	2.419826	104.000000	2.809546	0.045839
Bacteroides thetaiotaomicron	Adrenaline	t_gr	Adrenaline - NK	4.250000	0.949336	104.000000	4.476814	0.000186
Bacteroides thetaiotaomicron	Dopamine	t_gr	Dopamine - NK	5.527778	0.949336	104.000000	5.822784	0.000001
Bacteroides thetaiotaomicron	Levodopa	t_gr	Levodopa - NK	6.652778	0.949336	104.000000	7.007823	0.000000
Bacteroides thetaiotaomicron	Noradrenaline	t_gr	NK - Noradrenaline	5.861111	0.949336	104.000000	-6.173907	0.000000
Bacteroides thetaiotaomicron	Adrenaline	t_dr	Adrenaline - NK	-2.826389	1.771182	104.000000	-1.595764	0.503481
Bacteroides thetaiotaomicron	Dopamine	t_dr	Dopamine - NK	-2.881944	1.771182	104.000000	-1.627131	0.483656
Bacteroides thetaiotaomicron	Levodopa	t_dr	Levodopa - NK	-3.312500	1.771182	104.000000	-1.870220	0.339847
Bacteroides thetaiotaomicron	Noradrenaline	t_dr	NK - Noradrenaline	-4.215278	1.771182	104.000000	2.379924	0.129051

Species	Condition	Parameter	contrast	estimate	SE	df	t.ratio	p.value
Bifidobacterium stercoris	Adrenaline	auc_lin	Adrenaline - NK	-2.615411	0.716475	104.927786	-3.650387	0.003687
Bifidobacterium stercoris	Dopamine	auc_lin	Dopamine - NK	-3.148926	0.711088	104.144460	-4.428322	0.000225
Bifidobacterium stercoris	Levodopa	auc_lin	Levodopa - NK	-3.461687	0.716475	104.927786	-4.831554	0.000045
Bifidobacterium stercoris	Noradrenaline	auc_lin	NK - Noradrenaline	-3.919848	0.711088	104.144460	5.512468	0.000003
Bifidobacterium stercoris	Adrenaline	k_lin	Adrenaline - NK	-0.074540	0.021377	103.807902	-3.486972	0.006319
Bifidobacterium stercoris	Dopamine	k_lin	Dopamine - NK	-0.086342	0.021216	103.493112	-4.069641	0.000862
Bifidobacterium stercoris	Levodopa	k_lin	Levodopa - NK	-0.099670	0.021377	103.807902	-4.662541	0.000090
Bifidobacterium stercoris	Noradrenaline	k_lin	NK - Noradrenaline	-0.106720	0.021216	103.493112	5.030116	0.000020
Bifidobacterium stercoris	Adrenaline	death_lin	Adrenaline - NK	0.004256	0.009256	103.420035	0.459768	0.990677
Bifidobacterium stercoris	Dopamine	death_lin	Dopamine - NK	-0.010071	0.009187	103.258799	-1.096247	0.808104
Bifidobacterium stercoris	Levodopa	death_lin	Levodopa - NK	-0.003650	0.009256	103.420035	-0.394368	0.994817
Bifidobacterium stercoris	Noradrenaline	death_lin	NK - Noradrenaline	-0.006935	0.009187	103.258799	0.754880	0.942721
Bifidobacterium stercoris	Adrenaline	gr	Adrenaline - NK	-0.073698	0.029793	103.971402	-2.473687	0.104638
Bifidobacterium stercoris	Dopamine	gr	Dopamine - NK	-0.081674	0.029569	103.590531	-2.762195	0.051871
Bifidobacterium stercoris	Levodopa	gr	Levodopa - NK	-0.084373	0.029793	103.971402	-2.832012	0.043207
Bifidobacterium stercoris	Noradrenaline	gr	NK - Noradrenaline	-0.092102	0.029569	103.590531	3.114848	0.019725
Bifidobacterium stercoris	Adrenaline	dr	Adrenaline - NK	-0.004999	0.003435	103.956338	-1.455194	0.593679
Bifidobacterium stercoris	Dopamine	dr	Dopamine - NK	-0.004652	0.003409	103.581590	-1.364486	0.651597
Bifidobacterium stercoris	Levodopa	dr	Levodopa - NK	-0.002183	0.003435	103.956338	-0.635540	0.968919
Bifidobacterium stercoris	Noradrenaline	dr	NK - Noradrenaline	-0.003056	0.003409	103.581590	0.896206	0.897704
Bifidobacterium stercoris	Adrenaline	td	Adrenaline - NK	0.494071	0.279554	103.348044	1.767354	0.398147
Bifidobacterium stercoris	Dopamine	td	Dopamine - NK	1.021382	0.277459	103.214814	3.681198	0.003348
Bifidobacterium stercoris	Levodopa	td	Levodopa - NK	0.683690	0.279554	103.348044	2.445644	0.111569
Bifidobacterium stercoris	Noradrenaline	td	NK - Noradrenaline	0.812895	0.277459	103.214814	-2.929784	0.033252
Bifidobacterium stercoris	Adrenaline	lagC	Adrenaline - NK	1.840846	0.619634	103.077454	2.970859	0.029704
Bifidobacterium stercoris	Dopamine	lagC	Dopamine - NK	2.491076	0.614997	103.048114	4.050551	0.000926
Bifidobacterium stercoris	Levodopa	lagC	Levodopa - NK	2.865062	0.619634	103.077454	4.623796	0.000106

Species	Condition	Parameter	contrast	estimate	SE	df	t.ratio	p.value
Bifidobacterium stercoris	Noradrenaline	lagC	NK - Noradrenaline	3.042877	0.614997	103.048114	-4.947793	0.000029
Bifidobacterium stercoris	Adrenaline	t_k	Adrenaline - NK	-10.275950	2.477750	103.291151	-4.147290	0.000650
Bifidobacterium stercoris	Dopamine	t_k	Dopamine - NK	-6.017831	2.459187	103.179945	-2.447082	0.111221
Bifidobacterium stercoris	Levodopa	t_k	Levodopa - NK	-9.174499	2.477750	103.291151	-3.702754	0.003113
Bifidobacterium stercoris	Noradrenaline	t_k	NK - Noradrenaline	-9.212276	2.459187	103.179945	3.746066	0.002690
Bifidobacterium stercoris	Adrenaline	t_gr	Adrenaline - NK	2.435660	0.723362	103.055122	3.367137	0.009248
Bifidobacterium stercoris	Dopamine	t_gr	Dopamine - NK	2.649278	0.717949	103.034260	3.690064	0.003252
Bifidobacterium stercoris	Levodopa	t_gr	Levodopa - NK	3.071340	0.723362	103.055122	4.245924	0.000452
Bifidobacterium stercoris	Noradrenaline	t_gr	NK - Noradrenaline	3.649278	0.717949	103.034260	-5.082921	0.000016
Bifidobacterium stercoris	Adrenaline	t_dr	Adrenaline - NK	-11.127057	2.610466	103.259857	-4.262479	0.000424
Bifidobacterium stercoris	Dopamine	t_dr	Dopamine - NK	-8.136070	2.590912	103.160724	-3.140235	0.018336
Bifidobacterium stercoris	Levodopa	t_dr	Levodopa - NK	-11.517469	2.610466	103.259857	-4.412035	0.000241
Bifidobacterium stercoris	Noradrenaline	t_dr	NK - Noradrenaline	-7.136070	2.590912	103.160724	2.754270	0.052966
Enterococcus faecium	Adrenaline	auc_lin	Adrenaline - NK	5.133693	0.673459	104.262118	7.622873	0.000000
Enterococcus faecium	Dopamine	auc_lin	Dopamine - NK	5.674267	0.673459	104.262118	8.425556	0.000000
Enterococcus faecium	Levodopa	auc_lin	Levodopa - NK	3.606010	0.696566	103.790575	5.176836	0.000011
Enterococcus faecium	Noradrenaline	auc_lin	NK - Noradrenaline	4.790493	0.673459	104.262118	-7.113265	0.000000
Enterococcus faecium	Adrenaline	k_lin	Adrenaline - NK	0.047738	0.015108	104.180523	3.159832	0.017275
Enterococcus faecium	Dopamine	k_lin	Dopamine - NK	0.074242	0.015108	104.180523	4.914108	0.000032
Enterococcus faecium	Levodopa	k_lin	Levodopa - NK	0.031025	0.015759	103.832440	1.968664	0.288607
Enterococcus faecium	Noradrenaline	k_lin	NK - Noradrenaline	0.040754	0.015108	104.180523	-2.697537	0.061111
Enterococcus faecium	Adrenaline	death_lin	Adrenaline - NK	-0.005116	0.003169	103.568891	-1.614169	0.491841
Enterococcus faecium	Dopamine	death_lin	Dopamine - NK	-0.003671	0.003169	103.568891	-1.158444	0.774804
Enterococcus faecium	Levodopa	death_lin	Levodopa - NK	-0.005512	0.003405	106.403651	-1.618908	0.488753
Enterococcus faecium	Noradrenaline	death_lin	NK - Noradrenaline	-0.003637	0.003169	103.568891	1.147555	0.780795
Enterococcus faecium	Adrenaline	gr	Adrenaline - NK	0.047956	0.014306	102.770033	3.352076	0.009699
Enterococcus faecium	Dopamine	gr	Dopamine - NK	0.050295	0.014306	102.770033	3.515534	0.005783

Species	Condition	Parameter	contrast	estimate	SE	df	t.ratio	p.value
Enterococcus faecium	Levodopa	gr	Levodopa - NK	0.000939	0.015573	106.303248	0.060267	0.999997
Enterococcus faecium	Noradrenaline	gr	NK - Noradrenaline	0.047553	0.014306	102.770033	-3.323886	0.010580
Enterococcus faecium	Adrenaline	dr	Adrenaline - NK	0.008650	0.007047	103.637912	1.227528	0.735383
Enterococcus faecium	Dopamine	dr	Dopamine - NK	0.006491	0.007047	103.637912	0.921119	0.888153
Enterococcus faecium	Levodopa	dr	Levodopa - NK	0.010048	0.007556	106.172038	1.329731	0.673382
Enterococcus faecium	Noradrenaline	dr	NK - Noradrenaline	0.002822	0.007047	103.637912	-0.400386	0.994506
Enterococcus faecium	Adrenaline	td	Adrenaline - NK	-0.112276	0.042882	102.425253	-2.618248	0.074488
Enterococcus faecium	Dopamine	td	Dopamine - NK	-0.114609	0.042882	102.425253	-2.672644	0.065159
Enterococcus faecium	Levodopa	td	Levodopa - NK	0.016780	0.046799	104.788441	0.358555	0.996413
Enterococcus faecium	Noradrenaline	td	NK - Noradrenaline	-0.112172	0.042882	102.425253	2.615820	0.074929
Enterococcus faecium	Adrenaline	lagC	Adrenaline - NK	-0.243134	0.078205	102.175917	-3.108926	0.020124
Enterococcus faecium	Dopamine	lagC	Dopamine - NK	-0.541280	0.078205	102.175917	-6.921295	0.000000
Enterococcus faecium	Levodopa	lagC	Levodopa - NK	-0.089610	0.085448	103.259059	-1.048715	0.831947
Enterococcus faecium	Noradrenaline	lagC	NK - Noradrenaline	-0.293071	0.078205	102.175917	3.747474	0.002688
Enterococcus faecium	Adrenaline	t_k	Adrenaline - NK	-1.360526	3.772582	103.772925	-0.360635	0.996331
Enterococcus faecium	Dopamine	t_k	Dopamine - NK	0.667252	3.772582	103.772925	0.176869	0.999777
Enterococcus faecium	Levodopa	t_k	Levodopa - NK	0.776022	4.027277	105.625876	0.192691	0.999687
Enterococcus faecium	Noradrenaline	t_k	NK - Noradrenaline	-9.013303	3.772582	103.772925	2.389161	0.126483
Enterococcus faecium	Adrenaline	t_gr	Adrenaline - NK	-0.263226	0.077041	102.199271	-3.416673	0.007940
Enterococcus faecium	Dopamine	t_gr	Dopamine - NK	-0.554892	0.077041	102.199271	-7.202513	0.000000
Enterococcus faecium	Levodopa	t_gr	Levodopa - NK	-0.071435	0.084169	103.415714	-0.848704	0.914577
Enterococcus faecium	Noradrenaline	t_gr	NK - Noradrenaline	-0.277114	0.077041	102.199271	3.596951	0.004445
Enterococcus faecium	Adrenaline	t_dr	Adrenaline - NK	3.808403	3.489795	104.180475	1.091297	0.810659
Enterococcus faecium	Dopamine	t_dr	Dopamine - NK	1.447292	3.489795	104.180475	0.414721	0.993712
Enterococcus faecium	Levodopa	t_dr	Levodopa - NK	2.338681	3.640304	103.832547	0.642441	0.967680
Enterococcus faecium	Noradrenaline	t_dr	NK - Noradrenaline	-6.316597	3.489795	104.180475	1.810020	0.373400
Enterococcus mundtii	Adrenaline	auc_lin	Adrenaline - NK	5.574557	0.515674	102.702971	10.810230	0.000000

Species	Condition	Parameter	contrast	estimate	SE	df	t.ratio	p.value
Enterococcus mundtii	Dopamine	auc_lin	Dopamine - NK	6.278781	0.515674	102.702971	12.175868	0.000000
Enterococcus mundtii	Levodopa	auc_lin	Levodopa - NK	4.640487	0.561670	106.069768	8.261947	0.000000
Enterococcus mundtii	Noradrenaline	auc_lin	NK - Noradrenaline	5.192712	0.515674	102.702971	-10.069753	0.000000
Enterococcus mundtii	Adrenaline	k_lin	Adrenaline - NK	0.069747	0.011380	102.604772	6.129169	0.000000
Enterococcus mundtii	Dopamine	k_lin	Dopamine - NK	0.098207	0.011380	102.604772	8.630135	0.000000
Enterococcus mundtii	Levodopa	k_lin	Levodopa - NK	0.047760	0.012404	105.672673	3.850324	0.001859
Enterococcus mundtii	Noradrenaline	k_lin	NK - Noradrenaline	0.057190	0.011380	102.604772	-5.025731	0.000021
Enterococcus mundtii	Adrenaline	death_lin	Adrenaline - NK	-0.008669	0.003713	103.704784	-2.334945	0.142257
Enterococcus mundtii	Dopamine	death_lin	Dopamine - NK	-0.010877	0.003713	103.704784	-2.929627	0.033241
Enterococcus mundtii	Levodopa	death_lin	Levodopa - NK	-0.011997	0.003973	105.915729	-3.019943	0.025771
Enterococcus mundtii	Noradrenaline	death_lin	NK - Noradrenaline	-0.008942	0.003713	103.704784	2.408412	0.121228
Enterococcus mundtii	Adrenaline	gr	Adrenaline - NK	0.050692	0.013646	102.470220	3.714706	0.003000
Enterococcus mundtii	Dopamine	gr	Dopamine - NK	0.058316	0.013646	102.470220	4.273390	0.000409
Enterococcus mundtii	Levodopa	gr	Levodopa - NK	-0.000607	0.014889	105.028190	-0.040763	0.999999
Enterococcus mundtii	Noradrenaline	gr	NK - Noradrenaline	0.049414	0.013646	102.470220	-3.621055	0.004102
Enterococcus mundtii	Adrenaline	dr	Adrenaline - NK	0.020508	0.006327	103.941595	3.241411	0.013559
Enterococcus mundtii	Dopamine	dr	Dopamine - NK	0.025618	0.006327	103.941595	4.049131	0.000926
Enterococcus mundtii	Levodopa	dr	Levodopa - NK	0.023276	0.006705	104.823187	3.471193	0.006626
Enterococcus mundtii	Noradrenaline	dr	NK - Noradrenaline	0.019383	0.006327	103.941595	-3.063658	0.022842
Enterococcus mundtii	Adrenaline	td	Adrenaline - NK	-0.084162	0.028231	102.283028	-2.981158	0.028905
Enterococcus mundtii	Dopamine	td	Dopamine - NK	-0.091288	0.028231	102.283028	-3.233553	0.013937
Enterococcus mundtii	Levodopa	td	Levodopa - NK	0.017889	0.030832	103.955629	0.580197	0.977719
Enterococcus mundtii	Noradrenaline	td	NK - Noradrenaline	-0.080117	0.028231	102.283028	2.837885	0.042638
Enterococcus mundtii	Adrenaline	lagC	Adrenaline - NK	-0.300418	0.068587	102.157049	-4.380067	0.000274
Enterococcus mundtii	Dopamine	lagC	Dopamine - NK	-0.545770	0.068587	102.157049	-7.957285	0.000000
Enterococcus mundtii	Levodopa	lagC	Levodopa - NK	-0.149654	0.074945	103.130612	-1.996866	0.274866
Enterococcus mundtii	Noradrenaline	lagC	NK - Noradrenaline	-0.350864	0.068587	102.157049	5.115571	0.000014

Species	Condition	Parameter	contrast	estimate	SE	df	t.ratio	p.value
Enterococcus mundtii	Adrenaline	t_k	Adrenaline - NK	8.924298	3.923336	104.239138	2.274671	0.161453
Enterococcus mundtii	Dopamine	t_k	Dopamine - NK	12.160409	3.923336	104.239138	3.099508	0.020595
Enterococcus mundtii	Levodopa	t_k	Levodopa - NK	11.532662	4.068695	103.769081	2.834487	0.042936
Enterococcus mundtii	Noradrenaline	t_k	NK - Noradrenaline	7.452076	3.923336	104.239138	-1.899423	0.324140
Enterococcus mundtii	Adrenaline	t_gr	Adrenaline - NK	-0.389541	0.078294	102.153898	-4.975385	0.000026
Enterococcus mundtii	Dopamine	t_gr	Dopamine - NK	-0.611763	0.078294	102.153898	-7.813705	0.000000
Enterococcus mundtii	Levodopa	t_gr	Levodopa - NK	-0.192489	0.085551	103.109002	-2.249979	0.169960
Enterococcus mundtii	Noradrenaline	t_gr	NK - Noradrenaline	-0.403430	0.078294	102.153898	5.152780	0.000012
Enterococcus mundtii	Adrenaline	t_dr	Adrenaline - NK	11.721521	3.715366	104.020655	3.154877	0.017535
Enterococcus mundtii	Dopamine	t_dr	Dopamine - NK	12.040966	3.715366	104.020655	3.240856	0.013579
Enterococcus mundtii	Levodopa	t_dr	Levodopa - NK	13.765992	3.920855	104.439322	3.510967	0.005838
Enterococcus mundtii	Noradrenaline	t_dr	NK - Noradrenaline	10.221521	3.715366	104.020655	-2.751148	0.053338
Escherichia coli	Adrenaline	auc_lin	Adrenaline - NK	5.521983	0.460037	106.839433	12.003348	0.000000
Escherichia coli	Dopamine	auc_lin	Dopamine - NK	5.205590	0.449558	103.841150	11.579348	0.000000
Escherichia coli	Levodopa	auc_lin	Levodopa - NK	5.416613	0.449558	103.841150	12.048750	0.000000
Escherichia coli	Noradrenaline	auc_lin	NK - Noradrenaline	4.868559	0.449558	103.841150	-10.829655	0.000000
Escherichia coli	Adrenaline	k_lin	Adrenaline - NK	0.084471	0.009404	106.306204	8.982695	0.000000
Escherichia coli	Dopamine	k_lin	Dopamine - NK	0.097839	0.009123	103.570719	10.724878	0.000000
Escherichia coli	Levodopa	k_lin	Levodopa - NK	0.080063	0.009123	103.570719	8.776305	0.000000
Escherichia coli	Noradrenaline	k_lin	NK - Noradrenaline	0.069381	0.009123	103.570719	-7.605316	0.000000
Escherichia coli	Adrenaline	death_lin	Adrenaline - NK	-0.010711	0.004329	106.942061	-2.474445	0.104236
Escherichia coli	Dopamine	death_lin	Dopamine - NK	-0.015786	0.004277	104.084408	-3.690827	0.003230
Escherichia coli	Levodopa	death_lin	Levodopa - NK	-0.010239	0.004277	104.084408	-2.393781	0.125180
Escherichia coli	Noradrenaline	death_lin	NK - Noradrenaline	-0.010774	0.004277	104.084408	2.518926	0.094247
Escherichia coli	Adrenaline	gr	Adrenaline - NK	0.041503	0.013056	106.934280	3.178734	0.016245
Escherichia coli	Dopamine	gr	Dopamine - NK	0.023517	0.012951	104.156238	1.815755	0.370136
Escherichia coli	Levodopa	gr	Levodopa - NK	0.022494	0.012951	104.156238	1.736781	0.416231

Species	Condition	Parameter	contrast	estimate	SE	df	t.ratio	p.value
Escherichia coli	Noradrenaline	gr	NK - Noradrenaline	0.051759	0.012951	104.156238	-3.996422	0.001118
Escherichia coli	Adrenaline	dr	Adrenaline - NK	0.005251	0.004588	106.936377	1.144531	0.782456
Escherichia coli	Dopamine	dr	Dopamine - NK	0.010673	0.004518	104.018398	2.362289	0.134101
Escherichia coli	Levodopa	dr	Levodopa - NK	0.008059	0.004518	104.018398	1.783741	0.388540
Escherichia coli	Noradrenaline	dr	NK - Noradrenaline	0.007803	0.004518	104.018398	-1.727070	0.422064
Escherichia coli	Adrenaline	td	Adrenaline - NK	-0.153078	0.047602	106.930185	-3.215830	0.014545
Escherichia coli	Dopamine	td	Dopamine - NK	-0.077644	0.047265	104.174472	-1.642718	0.473872
Escherichia coli	Levodopa	td	Levodopa - NK	-0.064707	0.047265	104.174472	-1.369013	0.648731
Escherichia coli	Noradrenaline	td	NK - Noradrenaline	-0.177192	0.047265	104.174472	3.748887	0.002653
Escherichia coli	Adrenaline	lagC	Adrenaline - NK	0.167930	0.050420	103.364103	3.330658	0.010346
Escherichia coli	Dopamine	lagC	Dopamine - NK	0.016825	0.048695	103.044357	0.345509	0.996893
Escherichia coli	Levodopa	lagC	Levodopa - NK	0.204262	0.048695	103.044357	4.194720	0.000546
Escherichia coli	Noradrenaline	lagC	NK - Noradrenaline	0.154484	0.048695	103.044357	-3.172488	0.016684
Escherichia coli	Adrenaline	t_k	Adrenaline - NK	0.955028	5.206833	106.918028	0.183418	0.999743
Escherichia coli	Dopamine	t_k	Dopamine - NK	15.959435	5.112246	103.956444	3.121805	0.019317
Escherichia coli	Levodopa	t_k	Levodopa - NK	4.181657	5.112246	103.956444	0.817969	0.924556
Escherichia coli	Noradrenaline	t_k	NK - Noradrenaline	4.306657	5.112246	103.956444	-0.842420	0.916681
Escherichia coli	Adrenaline	t_gr	Adrenaline - NK	0.078164	0.050171	103.381830	1.557959	0.527607
Escherichia coli	Dopamine	t_gr	Dopamine - NK	0.024871	0.048455	103.046570	0.513285	0.985870
Escherichia coli	Levodopa	t_gr	Levodopa - NK	0.177649	0.048455	103.046570	3.666276	0.003522
Escherichia coli	Noradrenaline	t_gr	NK - Noradrenaline	0.122093	0.048455	103.046570	-2.519734	0.094147
Escherichia coli	Adrenaline	t_dr	Adrenaline - NK	6.687500	4.969597	106.926892	1.345683	0.663401
Escherichia coli	Dopamine	t_dr	Dopamine - NK	14.604167	4.937817	104.187077	2.957616	0.030757
Escherichia coli	Levodopa	t_dr	Levodopa - NK	4.895833	4.937817	104.187077	0.991498	0.858623
Escherichia coli	Noradrenaline	t_dr	NK - Noradrenaline	6.395833	4.937817	104.187077	-1.295276	0.694697
Eubacterium ventriosum	Adrenaline	auc_lin	Adrenaline - NK	3.324279	0.436394	103.365821	7.617609	0.000000
Eubacterium ventriosum	Dopamine	auc_lin	Dopamine - NK	3.670346	0.436394	103.365821	8.410626	0.000000

Species	Condition	Parameter	contrast	estimate	SE	df	t.ratio	p.value
Eubacterium ventriosum	Levodopa	auc_lin	Levodopa - NK	3.285213	0.436394	103.365821	7.528090	0.000000
Eubacterium ventriosum	Noradrenaline	auc_lin	NK - Noradrenaline	2.980152	0.451153	105.473672	-6.605634	0.000000
Eubacterium ventriosum	Adrenaline	k_lin	Adrenaline - NK	0.046417	0.014227	103.705161	3.262571	0.012727
Eubacterium ventriosum	Dopamine	k_lin	Dopamine - NK	0.071090	0.014227	103.705161	4.996811	0.000023
Eubacterium ventriosum	Levodopa	k_lin	Levodopa - NK	0.057638	0.014227	103.705161	4.051266	0.000920
Eubacterium ventriosum	Noradrenaline	k_lin	NK - Noradrenaline	0.047557	0.014621	106.643248	-3.252707	0.013024
Eubacterium ventriosum	Adrenaline	death_lin	Adrenaline - NK	0.001733	0.004687	103.664266	0.369626	0.995964
Eubacterium ventriosum	Dopamine	death_lin	Dopamine - NK	0.011621	0.004687	103.664266	2.479129	0.103366
Eubacterium ventriosum	Levodopa	death_lin	Levodopa - NK	0.006207	0.004687	103.664266	1.324240	0.676818
Eubacterium ventriosum	Noradrenaline	death_lin	NK - Noradrenaline	0.003261	0.004822	106.557055	-0.676348	0.961128
Eubacterium ventriosum	Adrenaline	gr	Adrenaline - NK	0.040014	0.011832	104.049749	3.382010	0.008805
Eubacterium ventriosum	Dopamine	gr	Dopamine - NK	0.041562	0.011832	104.049749	3.512810	0.005810
Eubacterium ventriosum	Levodopa	gr	Levodopa - NK	0.016682	0.011832	104.049749	1.409977	0.622682
Eubacterium ventriosum	Noradrenaline	gr	NK - Noradrenaline	0.046498	0.011996	106.940709	-3.876273	0.001688
Eubacterium ventriosum	Adrenaline	dr	Adrenaline - NK	-0.004613	0.003377	104.037486	-1.365845	0.650734
Eubacterium ventriosum	Dopamine	dr	Dopamine - NK	-0.007378	0.003377	104.037486	-2.184595	0.193715
Eubacterium ventriosum	Levodopa	dr	Levodopa - NK	-0.008299	0.003377	104.037486	-2.457220	0.108632
Eubacterium ventriosum	Noradrenaline	dr	NK - Noradrenaline	-0.003847	0.003426	106.939379	1.122707	0.794227
Eubacterium ventriosum	Adrenaline	td	Adrenaline - NK	-0.114433	0.035151	104.131560	-3.255496	0.012989
Eubacterium ventriosum	Dopamine	td	Dopamine - NK	-0.118719	0.035151	104.131560	-3.377439	0.008930
Eubacterium ventriosum	Levodopa	td	Levodopa - NK	-0.038743	0.035151	104.131560	-1.102204	0.805018
Eubacterium ventriosum	Noradrenaline	td	NK - Noradrenaline	-0.134801	0.035483	106.938497	3.799056	0.002209
Eubacterium ventriosum	Adrenaline	lagC	Adrenaline - NK	0.185406	0.028041	103.022817	6.611891	0.000000
Eubacterium ventriosum	Dopamine	lagC	Dopamine - NK	-0.013073	0.028041	103.022817	-0.466215	0.990170
Eubacterium ventriosum	Levodopa	lagC	Levodopa - NK	0.233102	0.028041	103.022817	8.312808	0.000000
Eubacterium ventriosum	Noradrenaline	lagC	NK - Noradrenaline	0.137900	0.029035	103.189372	-4.749521	0.000064
Eubacterium ventriosum	Adrenaline	t_k	Adrenaline - NK	-4.789394	3.217962	103.463148	-1.488332	0.572355

Species	Condition	Parameter	contrast	estimate	SE	df	t.ratio	p.value
Eubacterium ventriosum	Dopamine	t_k	Dopamine - NK	-6.081061	3.217962	103.463148	-1.889725	0.329355
Eubacterium ventriosum	Levodopa	t_k	Levodopa - NK	-5.650506	3.217962	103.463148	-1.755927	0.404875
Eubacterium ventriosum	Noradrenaline	t_k	NK - Noradrenaline	-3.173483	3.322952	105.919036	0.955019	0.874406
Eubacterium ventriosum	Adrenaline	t_gr	Adrenaline - NK	0.130840	0.045449	103.056471	2.878838	0.038185
Eubacterium ventriosum	Dopamine	t_gr	Dopamine - NK	-0.035827	0.045449	103.056471	-0.788291	0.933482
Eubacterium ventriosum	Levodopa	t_gr	Levodopa - NK	0.186395	0.045449	103.056471	4.101214	0.000770
Eubacterium ventriosum	Noradrenaline	t_gr	NK - Noradrenaline	0.107481	0.047058	103.460624	-2.283994	0.158419
Eubacterium ventriosum	Adrenaline	t_dr	Adrenaline - NK	-0.000000	1.963690	104.187077	-0.000000	1.000000
Eubacterium ventriosum	Dopamine	t_dr	Dopamine - NK	-3.513889	1.963690	104.187077	-1.789432	0.385231
Eubacterium ventriosum	Levodopa	t_dr	Levodopa - NK	-3.972222	1.963690	104.187077	-2.022836	0.262457
Eubacterium ventriosum	Noradrenaline	t_dr	NK - Noradrenaline	-1.777778	1.976328	106.926892	0.899536	0.896478
Klebsiella pneumoniae	Adrenaline	auc_lin	Adrenaline - NK	1.243679	0.555070	105.487854	2.240583	0.173059
Klebsiella pneumoniae	Dopamine	auc_lin	Dopamine - NK	2.523262	0.540586	103.346130	4.667645	0.000089
Klebsiella pneumoniae	Levodopa	auc_lin	Levodopa - NK	0.003543	0.540586	103.346130	0.006554	1.000000
Klebsiella pneumoniae	Noradrenaline	auc_lin	NK - Noradrenaline	1.400871	0.535324	102.715828	-2.616866	0.074718
Klebsiella pneumoniae	Adrenaline	k_lin	Adrenaline - NK	0.013929	0.012655	104.921075	1.100634	0.805838
Klebsiella pneumoniae	Dopamine	k_lin	Dopamine - NK	0.049505	0.012311	103.046857	4.021314	0.001028
Klebsiella pneumoniae	Levodopa	k_lin	Levodopa - NK	-0.011046	0.012311	103.046857	-0.897293	0.897294
Klebsiella pneumoniae	Noradrenaline	k_lin	NK - Noradrenaline	0.022141	0.012188	102.539941	-1.816541	0.369780
Klebsiella pneumoniae	Adrenaline	death_lin	Adrenaline - NK	0.006887	0.002924	105.645978	2.355407	0.135986
Klebsiella pneumoniae	Dopamine	death_lin	Dopamine - NK	0.002292	0.002849	103.442227	0.804534	0.928681
Klebsiella pneumoniae	Levodopa	death_lin	Levodopa - NK	0.009435	0.002849	103.442227	3.312147	0.010950
Klebsiella pneumoniae	Noradrenaline	death_lin	NK - Noradrenaline	0.010820	0.002821	102.774400	-3.835250	0.001984
Klebsiella pneumoniae	Adrenaline	gr	Adrenaline - NK	-0.011707	0.019601	106.021029	-0.597238	0.975225
Klebsiella pneumoniae	Dopamine	gr	Dopamine - NK	0.008245	0.019123	103.703981	0.431169	0.992701
Klebsiella pneumoniae	Levodopa	gr	Levodopa - NK	-0.064475	0.019123	103.703981	-3.371641	0.009103
Klebsiella pneumoniae	Noradrenaline	gr	NK - Noradrenaline	-0.001108	0.018942	102.939076	0.058484	0.999997

Species	Condition	Parameter	contrast	estimate	SE	df	t.ratio	p.value
Klebsiella pneumoniae	Adrenaline	dr	Adrenaline - NK	-0.005662	0.003316	106.631516	-1.707271	0.433926
Klebsiella pneumoniae	Dopamine	dr	Dopamine - NK	-0.003035	0.003248	104.340041	-0.934355	0.882889
Klebsiella pneumoniae	Levodopa	dr	Levodopa - NK	0.000327	0.003248	104.340041	0.100544	0.999977
Klebsiella pneumoniae	Noradrenaline	dr	NK - Noradrenaline	-0.008039	0.003219	103.369310	2.497599	0.099087
Klebsiella pneumoniae	Adrenaline	td	Adrenaline - NK	0.027189	0.065968	106.231445	0.412158	0.993861
Klebsiella pneumoniae	Dopamine	td	Dopamine - NK	-0.025826	0.064420	103.881291	-0.400900	0.994479
Klebsiella pneumoniae	Levodopa	td	Levodopa - NK	0.204796	0.064420	103.881291	3.179067	0.016333
Klebsiella pneumoniae	Noradrenaline	td	NK - Noradrenaline	-0.009293	0.063821	103.054830	0.145611	0.999897
Klebsiella pneumoniae	Adrenaline	lagC	Adrenaline - NK	0.095448	0.072727	102.732126	1.312416	0.684154
Klebsiella pneumoniae	Dopamine	lagC	Dopamine - NK	-0.020401	0.070596	102.218626	-0.288986	0.998453
Klebsiella pneumoniae	Levodopa	lagC	Levodopa - NK	0.290159	0.070596	102.218626	4.110132	0.000749
Klebsiella pneumoniae	Noradrenaline	lagC	NK - Noradrenaline	0.058430	0.069868	102.103505	-0.836290	0.918687
Klebsiella pneumoniae	Adrenaline	t_k	Adrenaline - NK	-4.402037	1.335882	106.763448	-3.295228	0.011439
Klebsiella pneumoniae	Dopamine	t_k	Dopamine - NK	-2.219012	1.310335	104.567022	-1.693469	0.442435
Klebsiella pneumoniae	Levodopa	t_k	Levodopa - NK	-5.228195	1.310335	104.567022	-3.989967	0.001141
Klebsiella pneumoniae	Noradrenaline	t_k	NK - Noradrenaline	-4.886867	1.298982	103.532307	3.762075	0.002543
Klebsiella pneumoniae	Adrenaline	t_gr	Adrenaline - NK	0.068840	0.038997	102.352516	1.765283	0.399414
Klebsiella pneumoniae	Dopamine	t_gr	Dopamine - NK	0.045320	0.037848	102.102907	1.197422	0.752848
Klebsiella pneumoniae	Levodopa	t_gr	Levodopa - NK	0.170662	0.037848	102.102907	4.509136	0.000167
Klebsiella pneumoniae	Noradrenaline	t_gr	NK - Noradrenaline	0.076290	0.037457	102.048142	-2.036746	0.256153
Klebsiella pneumoniae	Adrenaline	t_dr	Adrenaline - NK	0.634567	1.263580	106.912343	0.502198	0.986990
Klebsiella pneumoniae	Dopamine	t_dr	Dopamine - NK	-1.794950	1.243146	104.965458	-1.443876	0.600938
Klebsiella pneumoniae	Levodopa	t_dr	Levodopa - NK	-1.285933	1.243146	104.965458	-1.034418	0.838835
Klebsiella pneumoniae	Noradrenaline	t_dr	NK - Noradrenaline	-1.484240	1.232863	103.828642	1.203896	0.749129
Ruminococcus gnavus	Adrenaline	auc_lin	Adrenaline - NK	-0.160061	0.425887	103.577437	-0.375829	0.995696
Ruminococcus gnavus	Dopamine	auc_lin	Dopamine - NK	0.232108	0.427272	104.184504	0.543234	0.982539
Ruminococcus gnavus	Levodopa	auc_lin	Levodopa - NK	-0.628708	0.427272	104.184504	-1.471447	0.583214

Species	Condition	Parameter	contrast	estimate	SE	df	t.ratio	p.value
Ruminococcus gnavus	Noradrenaline	auc_lin	NK - Noradrenaline	-0.451869	0.441078	106.688006	1.024466	0.843550
Ruminococcus gnavus	Adrenaline	k_lin	Adrenaline - NK	0.000746	0.010830	103.117442	0.068870	0.999995
Ruminococcus gnavus	Dopamine	k_lin	Dopamine - NK	0.013479	0.010873	103.665549	1.239709	0.728202
Ruminococcus gnavus	Levodopa	k_lin	Levodopa - NK	-0.004660	0.010873	103.665549	-0.428602	0.992866
Ruminococcus gnavus	Noradrenaline	k_lin	NK - Noradrenaline	-0.006157	0.011257	106.069006	0.546896	0.982101
Ruminococcus gnavus	Adrenaline	death_lin	Adrenaline - NK	0.016676	0.004456	105.823341	3.742236	0.002696
Ruminococcus gnavus	Dopamine	death_lin	Dopamine - NK	0.007659	0.004451	105.823341	1.720633	0.425862
Ruminococcus gnavus	Levodopa	death_lin	Levodopa - NK	0.019680	0.004451	105.823341	4.421056	0.000228
Ruminococcus gnavus	Noradrenaline	death_lin	NK - Noradrenaline	0.012297	0.004481	106.775718	-2.744407	0.054099
Ruminococcus gnavus	Adrenaline	gr	Adrenaline - NK	-0.035232	0.027059	103.788163	-1.302037	0.690548
Ruminococcus gnavus	Dopamine	gr	Dopamine - NK	-0.005599	0.027138	104.397907	-0.206302	0.999590
Ruminococcus gnavus	Levodopa	gr	Levodopa - NK	-0.027064	0.027138	104.397907	-0.997283	0.856033
Ruminococcus gnavus	Noradrenaline	gr	NK - Noradrenaline	-0.013039	0.027971	106.845464	0.466164	0.990180
Ruminococcus gnavus	Adrenaline	dr	Adrenaline - NK	0.014045	0.004426	105.823341	3.173400	0.016541
Ruminococcus gnavus	Dopamine	dr	Dopamine - NK	0.009910	0.004421	105.823341	2.241410	0.172742
Ruminococcus gnavus	Levodopa	dr	Levodopa - NK	0.010106	0.004421	105.823341	2.285841	0.157625
Ruminococcus gnavus	Noradrenaline	dr	NK - Noradrenaline	0.012302	0.004450	106.775718	-2.764275	0.051401
Ruminococcus gnavus	Adrenaline	td	Adrenaline - NK	0.173619	0.177921	103.640660	0.975821	0.865517
Ruminococcus gnavus	Dopamine	td	Dopamine - NK	-0.138288	0.178481	104.250022	-0.774805	0.937321
Ruminococcus gnavus	Levodopa	td	Levodopa - NK	-0.056996	0.178481	104.250022	-0.319336	0.997714
Ruminococcus gnavus	Noradrenaline	td	NK - Noradrenaline	-0.095616	0.184166	106.742352	0.519186	0.985259
Ruminococcus gnavus	Adrenaline	lagC	Adrenaline - NK	-0.078363	0.321311	102.150773	-0.243886	0.999206
Ruminococcus gnavus	Dopamine	lagC	Dopamine - NK	0.079688	0.322870	102.267241	0.246812	0.999168
Ruminococcus gnavus	Levodopa	lagC	Levodopa - NK	0.925877	0.322870	102.267241	2.867647	0.039393
Ruminococcus gnavus	Noradrenaline	lagC	NK - Noradrenaline	0.588988	0.335638	102.842183	-1.754834	0.405553
Ruminococcus gnavus	Adrenaline	t_k	Adrenaline - NK	-3.422116	1.758781	105.434533	-1.945731	0.300016
Ruminococcus gnavus	Dopamine	t_k	Dopamine - NK	-0.707277	1.758204	105.649366	-0.402272	0.994407

Species	Condition	Parameter	contrast	estimate	SE	df	t.ratio	p.value
Ruminococcus gnavus	Levodopa	t_k	Levodopa - NK	-3.068388	1.758204	105.649366	-1.745183	0.411147
Ruminococcus gnavus	Noradrenaline	t_k	NK - Noradrenaline	-2.316109	1.781767	106.796359	1.299894	0.691849
Ruminococcus gnavus	Adrenaline	t_gr	Adrenaline - NK	0.123465	0.326870	102.140081	0.377720	0.995610
Ruminococcus gnavus	Dopamine	t_gr	Dopamine - NK	-0.004807	0.328457	102.248810	-0.014635	1.000000
Ruminococcus gnavus	Levodopa	t_gr	Levodopa - NK	0.953526	0.328457	102.248810	2.903044	0.035815
Ruminococcus gnavus	Noradrenaline	t_gr	NK - Noradrenaline	0.580593	0.341455	102.786153	-1.700351	0.438307
Ruminococcus gnavus	Adrenaline	t_dr	Adrenaline - NK	4.284722	2.181358	105.823341	1.964246	0.290673
Ruminococcus gnavus	Dopamine	t_dr	Dopamine - NK	1.659722	2.179049	105.823341	0.761673	0.940926
Ruminococcus gnavus	Levodopa	t_dr	Levodopa - NK	4.368056	2.179049	105.823341	2.004570	0.270983
Ruminococcus gnavus	Noradrenaline	t_dr	NK - Noradrenaline	5.562500	2.193440	106.775718	-2.535971	0.090363
Ruminococcus lactaris	Adrenaline	auc_lin	Adrenaline - NK	0.334407	0.210366	102.176862	1.589643	0.507430
Ruminococcus lactaris	Dopamine	auc_lin	Dopamine - NK	0.411386	0.211384	102.311893	1.946157	0.300006
Ruminococcus lactaris	Levodopa	auc_lin	Levodopa - NK	-0.079400	0.211384	102.311893	-0.375618	0.995704
Ruminococcus lactaris	Noradrenaline	auc_lin	NK - Noradrenaline	0.035862	0.219729	102.976626	-0.163210	0.999838
Ruminococcus lactaris	Adrenaline	k_lin	Adrenaline - NK	-0.031708	0.006125	102.223981	-5.177051	0.000011
Ruminococcus lactaris	Dopamine	k_lin	Dopamine - NK	-0.028893	0.006154	102.391401	-4.694840	0.000080
Ruminococcus lactaris	Levodopa	k_lin	Levodopa - NK	-0.043555	0.006154	102.391401	-7.077383	0.000000
Ruminococcus lactaris	Noradrenaline	k_lin	NK - Noradrenaline	-0.038695	0.006396	103.211399	6.049531	0.000000
Ruminococcus lactaris	Adrenaline	death_lin	Adrenaline - NK	0.017402	0.003109	103.159288	5.596466	0.000002
Ruminococcus lactaris	Dopamine	death_lin	Dopamine - NK	0.017173	0.003121	103.716051	5.501477	0.000003
Ruminococcus lactaris	Levodopa	death_lin	Levodopa - NK	0.017286	0.003121	103.716051	5.537844	0.000002
Ruminococcus lactaris	Noradrenaline	death_lin	NK - Noradrenaline	0.017191	0.003231	106.143858	-5.320510	0.000006
Ruminococcus lactaris	Adrenaline	gr	Adrenaline - NK	0.028302	0.013925	104.876412	2.032469	0.257922
Ruminococcus lactaris	Dopamine	gr	Dopamine - NK	0.032683	0.013937	105.302125	2.345065	0.139079
Ruminococcus lactaris	Levodopa	gr	Levodopa - NK	0.005691	0.013937	105.302125	0.408379	0.994074
Ruminococcus lactaris	Noradrenaline	gr	NK - Noradrenaline	0.025496	0.014222	106.931385	-1.792697	0.383202
Ruminococcus lactaris	Adrenaline	dr	Adrenaline - NK	-0.004422	0.002016	104.320964	-2.194073	0.190098

Species	Condition	Parameter	contrast	estimate	SE	df	t.ratio	p.value
Ruminococcus lactaris	Dopamine	dr	Dopamine - NK	-0.006092	0.002020	104.879056	-3.016682	0.026055
Ruminococcus lactaris	Levodopa	dr	Levodopa - NK	-0.004677	0.002020	104.879056	-2.315898	0.148046
Ruminococcus lactaris	Noradrenaline	dr	NK - Noradrenaline	-0.003555	0.002072	106.999072	1.715332	0.429018
Ruminococcus lactaris	Adrenaline	td	Adrenaline - NK	-0.073509	0.037059	105.076439	-1.983579	0.281178
Ruminococcus lactaris	Dopamine	td	Dopamine - NK	-0.076441	0.037074	105.436057	-2.061817	0.244518
Ruminococcus lactaris	Levodopa	td	Levodopa - NK	-0.008721	0.037074	105.436057	-0.235241	0.999312
Ruminococcus lactaris	Noradrenaline	td	NK - Noradrenaline	-0.065360	0.037747	106.881809	1.731536	0.419251
Ruminococcus lactaris	Adrenaline	lagC	Adrenaline - NK	0.188057	0.025049	102.048011	7.507693	0.000000
Ruminococcus lactaris	Dopamine	lagC	Dopamine - NK	-0.013837	0.025171	102.086841	-0.549733	0.981744
Ruminococcus lactaris	Levodopa	lagC	Levodopa - NK	0.218378	0.025171	102.086841	8.675698	0.000000
Ruminococcus lactaris	Noradrenaline	lagC	NK - Noradrenaline	0.154235	0.026172	102.280573	-5.893084	0.000000
Ruminococcus lactaris	Adrenaline	t_k	Adrenaline - NK	-13.970190	1.858350	104.152523	-7.517522	0.000000
Ruminococcus lactaris	Dopamine	t_k	Dopamine - NK	-12.423548	1.862547	104.735413	-6.670193	0.000000
Ruminococcus lactaris	Levodopa	t_k	Levodopa - NK	-13.076326	1.862547	104.735413	-7.020669	0.000000
Ruminococcus lactaris	Noradrenaline	t_k	NK - Noradrenaline	-13.766046	1.914097	106.981351	7.191929	0.000000
Ruminococcus lactaris	Adrenaline	t_gr	Adrenaline - NK	0.126775	0.036105	102.091426	3.511313	0.005876
Ruminococcus lactaris	Dopamine	t_gr	Dopamine - NK	-0.059839	0.036281	102.163951	-1.649310	0.469831
Ruminococcus lactaris	Levodopa	t_gr	Levodopa - NK	0.148495	0.036281	102.163951	4.092906	0.000798
Ruminococcus lactaris	Noradrenaline	t_gr	NK - Noradrenaline	0.145680	0.037721	102.524192	-3.862067	0.001810
Ruminococcus lactaris	Adrenaline	t_dr	Adrenaline - NK	-1.527778	1.284590	105.823341	-1.189312	0.757483
Ruminococcus lactaris	Dopamine	t_dr	Dopamine - NK	-0.819444	1.283230	105.823341	-0.638580	0.968384
Ruminococcus lactaris	Levodopa	t_dr	Levodopa - NK	-1.166667	1.283230	105.823341	-0.909164	0.892811
Ruminococcus lactaris	Noradrenaline	t_dr	NK - Noradrenaline	-2.263889	1.291705	106.775718	1.752637	0.406665
